# Analysis of Antibiotic Resistance Genes in Water Reservoirs and Related Wastewater from Animal Farms in Central China

**DOI:** 10.3390/microorganisms12020396

**Published:** 2024-02-16

**Authors:** Yapei Rui, Gang Qiu

**Affiliations:** College of Animal Science and Technology, Xinyang Agriculture and Forestry University, Xinyang 464000, China; 2008200001@xyafu.edu.cn

**Keywords:** antibiotic resistance genes, animal farms, wastewater, drinking water, fluoroquinolone resistance genes

## Abstract

This study aimed to explore the phenotype and relationship of drug resistance genes in livestock and poultry farm wastewater and drinking water reservoirs to provide evidence for the transmission mechanisms of drug resistance genes, in order to reveal the spread of drug resistance genes in wastewater from intensive farms in Central China to urban reservoirs that serve as drinking water sources and provide preliminary data for the treatment of wastewater from animal farms to reduce the threat to human beings. DNA extraction and metagenomic sequencing were performed on eight groups of samples collected from four water reservoirs and four related wastewaters from animal farms in Central China. Metagenomic sequencing showed that the top 20 AROs with the highest abundance were *vanT*_gene, *vanY*_gene, *adeF*, *qacG*, *Mtub_rpsL_STR*, *vanY*_gene_, *vanW*_gene, *Mtub_murA_FOF*, *vanY*_gene, *vanH*_gene, *FosG*, *rsmA*, *qacJ*, *RbpA*, *vanW*_gene, *aadA6*, *vanY*_gene, *sul4*, *sul1*, and *InuF*. The resistance genes mentioned above belong to the following categories of drug resistance mechanisms: antibiotic target replacement, antibiotic target protection, antibiotic inactivation, and antibiotic efflux. The resistomes that match the top 20 genes are *Streptococcus agalactiae* and *Streptococcus anginosus*; *Enterococcus faecalis*; *Enterococcus faecium*; *Actinomyces viscosus* and *Bacillus cereus*. *Enterococcus faecium*; *Clostridium tetani*; *Streptococcus agalactiae* and *Streptococcus anginosus*; *Streptococcus agalactiae* and *Streptococcus anginosus*; *Acinetobacter baumannii*, *Bifidobacterium bifidum*, *Bifidobacterium breve*, *Bifidobacterium longum*, *Corynebacterium jeikeium*, *Corynebacterium urealyticum*, *Mycobacterium kansasii*, *Mycobacterium tuberculosis*, *Schaalia odontolytica*, and *Trueperella pyogenes*; *Mycobacterium avium* and *Mycobacterium tuberculosis*; *Aeromonas caviae*, *Enterobacter hormaechei*, *Vibrio cholerae*, *Vibrio metoecus*, *Vibrio parahaemolyticus*, and *Vibrio vulnificus*; *Pseudomonas aeruginosa* and *Pseudomonas fluorescens*; *Staphylococcus aureus* and *Staphylococcus equorum*; *M. avium*, *Achromobacter xylosoxidans*, and *Acinetobacter baumannii*; *Sphingobium yanoikuyae*, *Acinetobacter indicus*, *Morganella morganii*, *Proteus mirabilis*, *Proteus vulgaris*, *Providencia rettgeri*, and *Providencia stuartii*. Unreported drug resistance genes and drug-resistant bacteria in Central China were identified in 2023. In the transmission path of drug resistance genes, the transmission path from aquaculture wastewater to human drinking water sources cannot be ignored. For the sake of human health and ecological balance, the treatment of aquaculture wastewater needs to be further strengthened, and the effective blocking of drug resistance gene transmission needs to be considered.

## 1. Introduction

Antibiotic resistance (ABR) is an escalating global public health concern, and it is acknowledged as a pivotal issue within the One Health Framework (Centers for Disease Control and Prevention, USA). The interconnected domains of the One Health Framework have identified that the emergence, evolution, and dissemination of antibiotic-resistant microorganisms at both the local and global scales pose a substantial risk to global health. Multiple environmental reservoirs of microorganisms, including water and farm waste, contribute to the dissemination of ABR [1,2].

Inadequate waste treatment steps in the livestock sector exacerbate the persistence of resistant superbugs and antibiotic resistance genes (ARGs) in water and soil [3]. Research has demonstrated that the patterns of ABR genes in wastewater resemble those found in clinical settings. Metagenomic tools and genetic relatedness have been employed to predict and continuously monitor ABR microbes.

To ensure drinking water safety, sewage discharge that is up to standards, and the recycling of drinking water for livestock water purposes, it is imperative to establish guidelines that define the threshold levels of antibiotic-resistant bacteria and ARGs. Metagenomics proves to be an invaluable tool for delineating microbial diversity, identifying genes, and reconstructing the complete genomes of microbial communities. One of the key advantages of this tool lies in its sensitivity in detecting species abundances and identifying ARGs.

Metagenomics is emerging as a valuable alternative to DNA sequencing for studying microbial diversity in veterinary clinical and water samples [4,5]. Traditional methods, such as culturing bacterial pathogens or sequencing isolates, can be logistically challenging and impractical in certain situations. To overcome these limitations, a pathogen-independent approach, such as metagenome sequencing, can detect the entire genetic material in a sample.

Metagenomics offers a comprehensive assessment of pathogenic microorganism variation and the spread of ARGs across different health niches, and it provides valuable insights into the association between pathogens and various ARGs, facilitated by innovative techniques such as metagenome high-throughput chromosome conformation capture (Hi-C).

The consequences of administering fluoroquinolones as antibiotics in animal drinking water can be studied using metagenomic sequencing. This approach allows for the identification of microbial taxonomy and the presence of genes responsible for resistance in drinking water [6].

This study aimed to analyze antibiotic resistance genes in wastewater from large- and medium-sized animal farms and related drinking water reservoirs in Central China.

## 2. Methods

### 2.1. Sample Collection and Preparation

The corresponding numbers of samples were as follows: Medium Reservoir 21 Water Sample (A21), Small Reservoir 22 Water Sample (A22), Small Reservoir 23 Water Sample (A23), Small Reservoir 24 Water Sample (A24), Large Animal Farm 1 Sewage Sample (B21), Large Animal Farm 2 Sewage Sample (B22), Medium Animal Farm 3 Sewage Sample (B23), and Medium Animal Farm 4 Sewage Sample (B24). The water samples were collected in July 2023 by the author using standard water sample collectors from eight locations.

#### 2.1.1. The Sampling Location

The Sampling Location was 10 cm below the Water Surface of the Sampling Point.

#### 2.1.2. Collection of Sewage Samples

1. When collecting sewage samples from the measuring or regulating tank, three diagonal positions were sampled. After stirring evenly, instantaneous water samples were collected, and multiple sewage samples were combined to make a mixed sample.
2. When collecting sewage samples from drainage channels or pipes, sewage samples collected at the same sampling point were mixed to form a mixed sample.
3. When sampling, the sampler and water sample container were first rinsed with sampling water three times. Then, the water sample was collected into the container, and the corresponding fixative was immediately added according to the requirements of HJ493, after which the sample was properly labelled.
4. The sampling amount for testing a single project was executed following the HJ/T91 regulations, and the number of sewage samples for testing multiple projects was increased. In contrast, each sample had a duplicate sample.
5. On-site measurements: The pH meter and thermometer were immersed 5 cm below the surface of the drainage channel or regulating pool, and the pH value and temperature were recorded after the readings became stable.

### 2.2. Experimental Procedure

#### 2.2.1. DNA Extraction and Sample Quality Control

DNA was extracted using a DNA Kit (TianGen, Beijing, China) and the Cetyltrimethylammonium bromide (CTAB) extraction method. After DNA extraction, each sample was divided into two parts for preservation: one for 16s rDNA testing and the other for metagenomic sequencing.

#### 2.2.2. Construction of the Library and Quality Control

The genomic DNA underwent random shearing, resulting in the generation of short fragments. Subsequently, sequencing libraries were created. The resulting fragments were processed, utilizing an Illumina adapter provided by ABclonal Technology Co., Ltd. Wuhan, China. in Wuhan, China. Fragments containing adapters were then subjected to polymerase chain reaction (PCR) amplification, size selection, and purification.

To ensure the quality of the library, it underwent quantification via qPCR. The sequencing platform was Illumina PE150.

### 2.3. Bioinformatics Analysis Pipeline

#### 2.3.1. Pre-Processing of Sequencing Results

Readfq was employed to preprocess raw data from the sequencing platform and obtain clean data for analyses. 

Bowtie2 was utilized with default settings, employing the end-to-end sensitive mode and employing parameter settings of −I 200 and ×400.

#### 2.3.2. Metagenome Assembly

The clean data underwent assembly analysis using MEGAHIT 1.2.9 software. The assembly parameters were set as follows: pre-set meta-large (end-to-end, sensitive, I 200, ×400) [7,8,9,10,11,12,13,14,15,16,17,18].

#### 2.3.3. Abundance Analysis

For each sample, MetaGeneMark was employed to predict Open Reading Frames (ORFs) in the scaftigs (≥500 bp) using default parameters. Predictions with a length of <100 nt were filtered out from the results.

To eliminate redundancy in the ORF predictions, CD-HIT was employed. 

The abundance of genes was calculated based on the following formula, where *r* represents the number of gene reads on alignment, and *L* denotes the length of the gene.

$$Gk=\frac{rk}{Lk}\cdot \frac{1}{\sum_{i=1}^{n}\frac{ri}{li}}$$

#### 2.3.4. Taxon Annotation

DIAMOND 3.0 software was employed to align UniGene sequences with those of bacteria, fungi, archaea, and viruses extracted from NCBI’s Non-Redundant Protein Sequence (NR) database. The alignment was performed using blastp with the parameter setting e 1 × 10^−5^.

The sequences were filtered based on their alignment results, selecting those with an evalue ≤ min. evalue*10. Since each sequence could have multiple alignment results, the species annotation information was determined using the LCA algorithm, which was applied to the systematic taxonomy of the MEGAN 6 software.

From the LCA annotation results and the gene abundance table, the abundance of each sample at different taxonomic levels (kingdom, phylum, class, order, family, genus, or species) was obtained. Additionally, the corresponding gene abundance tables were acquired. The abundance of a taxon in a sample was calculated as the sum of the abundances of the genes annotated relative to that taxon. The number of genes belonging to a taxon in a sample was determined by counting the non-zero abundances among the annotated genes.

Using the abundance tables at each taxonomic level, various analyses were conducted, including a Krona analysis, a relative abundance overview, and an abundance clustering heatmap. These analyses were combined with dimension reduction techniques, such as principal component analysis (PCA), using the R ade4 package, and non-metric multidimensional scaling (NMDS) analysis was carried out via the R vegan package. To test for differences between groups, an analysis of similarities (ANOSIM) was performed via the R vegan package. Taxon differences between groups were explored using Metastats and LEfSe analyses. Permutation tests between groups at each taxonomic level were conducted using Metastats to obtain *p*-values. The *p*-values were corrected via the White and Nagarajani method to obtain q-values. The LEfSe 1.12.1 software, with a default linear discriminant analysis (LDA) score of 4, was used for the LEfSe analysis.

Finally, we employed the random forest algorithm (utilizing the R pROC and randomForest packages) to identify species at the species level via gradient selection and model construction. Significant species were identified using mean decrease accuracy and mean decrease Gini, followed by cross-validation (using the default 10-fold method) for each model and the generation of receiver operator curves (ROCs). 

To visually represent the data, we conducted a principal coordinates analysis (PCoA) based on the Bray–Curtis distance and selected the principal coordinate combination with the highest contribution rate.

#### 2.3.5. Annotations of the Common Functional Database

Annotations of the common functional database were performed using the DIAMOND software. Unigenes were aligned with the functional database using the following parameter settings: blastp, −e 1 × 10^−5^. The functional databases included the Kyoto Encyclopaedia of Genes and Genomes (KEGG) database, the eggNOG database, and the CAZy database. The best BLAST hits were selected for subsequent analyses based on the alignment results of each sequence.

From these alignment results, we calculated the relative abundances at different functional levels. The relative abundance at each functional level was determined by summing the relative abundances of genes annotated at that specific level.

To derive the gene number table for each sample at each taxonomic level, we utilized the functional annotation results and the gene abundance table. The number of genes with a specific function in a sample was equivalent to the number of genes with non-zero abundances among the genes annotated with that particular function.

Using the abundance table at each taxonomic level, we conducted annotated gene statistics, generated a relative abundance overview, and constructed an abundance clustering heat map. These analyses were combined with dimension reduction techniques, such as PCA and NMDS; ANOSIM analysis to assess inter-/intra-group differences based on functional abundance; comparative analysis of metabolic pathways; and Metastat and LEfSe analyses to identify inter-group functional differences.

#### 2.3.6. Annotations of Resistance Genes

Unigenes were aligned relative to the CARD database using the Resistance Gene Identifier (RGI) 6.0.3 software provided by the CARD database (RGI built-in blastp, default evalue < 1 × 10^−30^). The relative abundance of each antibiotic resistance ontology (ARO) was calculated based on the RGI alignment results and unigene abundance information. Several analyses were conducted, including an abundance histogram, abundance clustering heat map, abundance distribution circle map, ARO difference analysis between groups, resistance gene annotation (unigenes annotated as ARO), and the species attribution analysis of resistance mechanisms. For abbreviation purposes, certain AROs with long names were shortened to the first three words followed by an underline [19,20].

## 3. Results

### 3.1. Taxon Annotations

#### 3.1.1. Overview of Taxon Relative Abundances

**Figure 1:**
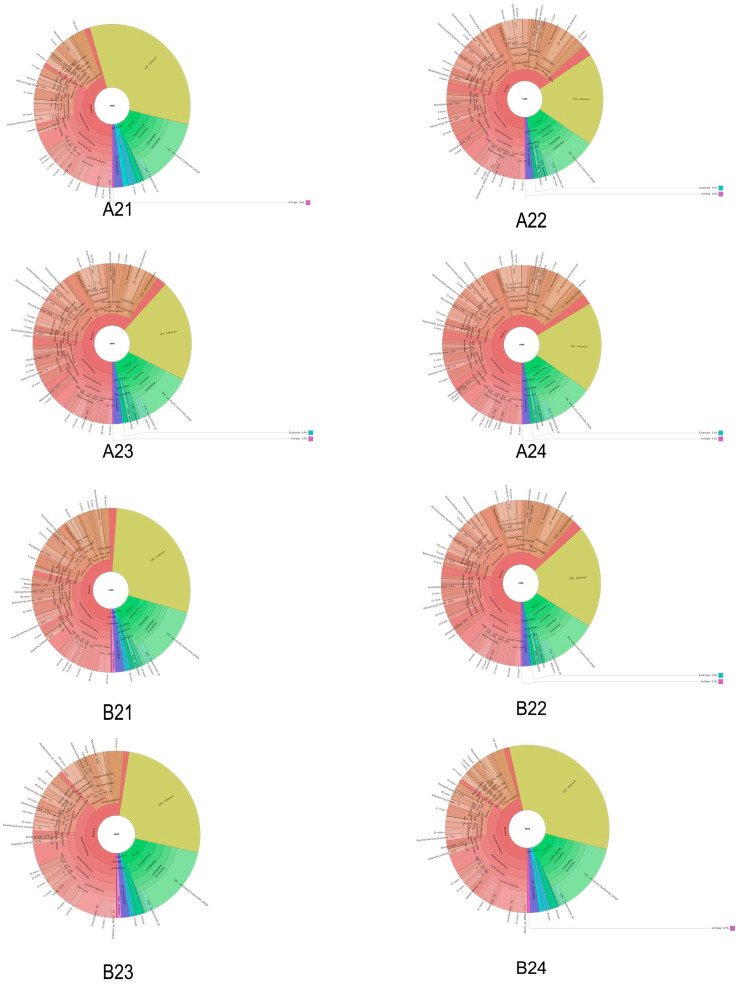
Taxon annotation results using Krona.

In order to comprehensively and intuitively display the relative abundance of species at different taxonomic levels in each sample, Krona was adopted to visually display the species annotation results. A Krona example diagram is shown in Figure 1.

In Figure 1, the different classification levels (phylum, order, family, genus, and species) are represented by the circles, with the fan size indicating the relative proportion of each taxon. Separate figures are included in Supplement Material S1.

Based on the relative abundance table of different taxonomic levels, the taxonomic groups with the highest relative abundance (top 10) were selected, and the rest were set to others. The relative abundance histogram of each sample (group) corresponding to the classification results at different classification levels was constructed.

**Figure 2:**
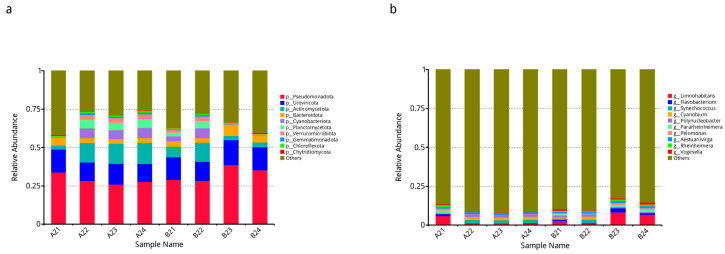
Relative abundances of taxa at the phylum and genus levels. (**a**). Horizontal relative abundance column chart of phylum. (**b**). Horizontal relative abundance column chart of genera. The horizontal axis represents the sample name. The vertical axis shows the relative proportion of annotated genera of a certain type. The types of objects corresponding to each color block are shown in the legend on the right.

The histogram of the relative abundance at different taxonomic levels is shown in Figure 2.

The top 10 taxa with the highest relative abundances at the phylum level were Pseudomonadota, Uroviricota, Actinomycetota, Bacteroidetes, Cyanobacteria, Planctomycetota, Verrucomicrobiota, Gemmatimonadota, Chloroflexota, and Chytridiomycota. The top ten genera with the highest relative abundances were *Limnohabitans*, *Flavobacterium*, *Synechococcus*, *Cyanobium*, *Polynucleobacter*, *Pararheinheimera*, *Pelomonas*, *Aestuariivirga*, *Rheinheimera*, and *Vogesella*.

#### 3.1.2. Cluster Analysis of Gene Numbers and Abundances at the Genus Level

**Figure 3:**
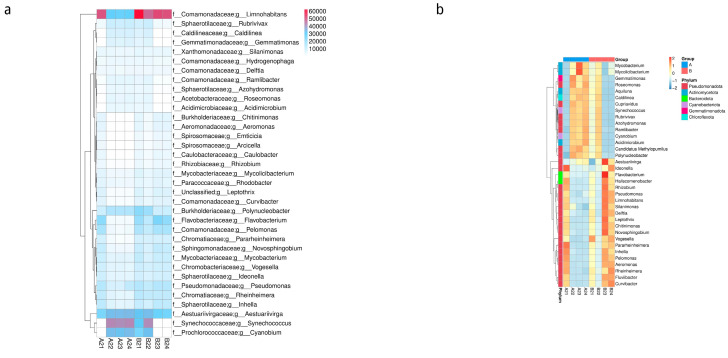
Cluster heatmap of gene number and abundance at the genus level. (**a**). Heatmap showing the statistical gene annotation numbers. The horizontal axis represents the sample name, and the vertical axis represents genus information. Different colors represent the number of unigenes. (**b**). Cluster heatmap of relative abundances at the genus level. The horizontal axis represents sample information, and the vertical axis represents genus information. The cluster tree on the left represents the genus clustering. The middle heatmap displays the standardized relative abundances, with the Z value indicating the difference between the relative abundance of the sample in that classification and the average relative abundance of all samples in that classification divided by the standard deviation of all samples in that classification.

The top 35 genera with the highest abundance and their abundance information in each sample were thermologically mapped, and the clustering results from the species level are shown in Figure 3.

#### 3.1.3. Sample Clustering Analysis Based on Taxon Abundances

**Figure 4:**
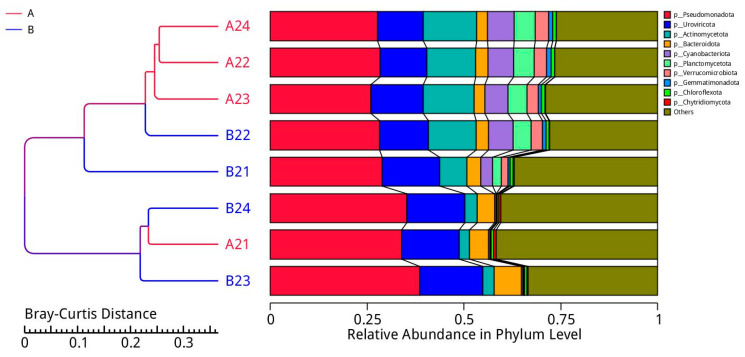
Clustering tree based on the Bray–Curtis distance.

A cluster analysis was conducted to construct a cluster tree of the samples, revealing similarities between the different samples. The clustering tree is shown in Figure 4.

The left side shows the Bray–Curtis distance clustering tree structure, and the right shows the relative abundance of the taxa (phylum level) for each sample. 

### 3.2. Overview of Resistance Gene Abundances

Beginning with the relative abundance table of the resistance genes, we computed the content and percentage of the antibiotic resistance ontologies (AROs) in each sample and subsequently identified the top 20 AROs with the highest abundance. 

The AROs with the highest abundance in the top 20 AROs were *vanT*_gene, *vanY*_gene, *adeF*, *qacG*, *Mtub_rpsL_STR*, *vanY*_gene, *vanW*_gene, *Mtub_murA_FOF*, *vanY*_gene, *vanH*_gene, *FosG*, *rsmA*, *qacJ*, *RbpA*, *vanW*_gene, *aadA6*, *vanY*_gene, *sul4*, *sul1*, and *InuF*.

The relative abundance ranking of the top 20 AROs among all the AROs was as follows: *vanT*_gene_in_*vanG*_cluster, *vanY*_gene_in_*vanB*_cluster, *adeF*, *qacG*, *Mtub_rpsL_STR*, *vanY*_in_*vanM*_cluster, *vanW*_in_*vanl*_cluster, *Mtub_murA_FOF*, *vanY*_in_*vanA*_cluster, *vanH*_in_*vanO*_cluster, *FosG*, *rsmA*, *qacJ*, *RbpA*, *vanW*_in_*vanG*_cluster, *aadA6*, *vanY*_in_*vanG*_cluster, *sul4*, *sul1*, and *InuF*.

The resistance genes mentioned above belong to the following categories of drug resistance mechanisms: antibiotic target replacement, antibiotic inactivation, antibiotic target protection, and antibiotic efflux.

In the glycopeptide resistance gene cluster, the drug class was identified as glycopeptide antibiotics. The resistance mechanism in this cluster is antibiotic target alteration, which has been extensively detected in wastewater from small- and medium-sized reservoirs and aquaculture farms in Central China. Within this cluster, the gene *vanTG* is a variant of the *vanT* gene and is commonly found. The resistomes that match with this gene are *Streptococcus agalactiae* and *Streptococcus anginosus*. Similarly, the gene *vanYB* is a variant of *vanY* in the *vanB* gene cluster, and its matching resistome is *Enterococcus faecalis*. Additionally, the gene *vanYM* is a variant of *vanY* in the *vanM* gene cluster, and its matching resistome is *Enterococcus faecium*. Moreover, the gene *vanWI* is a variant of *vanW* found in the *vanI* gene cluster, and the resistomes that match with this gene are *Actinomyces viscosus* and *Bacillus cereus*. Furthermore, the gene *vanYA* is a variant of *vanY* in the *vanA* gene cluster, and it matches with the resistome of *Enterococcus faecium*. Within the *vanO* gene cluster, the *vanHO* gene is a variant of *vanH* and has been found in resistomes with sequence variants, including *Clostridium tetani*. Lastly, the gene *vanWG* is a *vanW* variant observed in the *vanG* gene cluster, and its matching resistomes are *Streptococcus agalactiae* and *Streptococcus anginosus*. A similar observation is found for the gene *vanYG*, which is a *vanY* variant observed in the *vanG* gene cluster, and the resistomes that match with this gene are *Streptococcus agalactiae* and *Streptococcus anginosus*.

The gene *AdeF* encodes a membrane fusion protein called AdeFGH, which is part of a multidrug efflux complex. This gene is resistant to the tetracycline and fluoroquinolone antibiotic drug classes. The resistance mechanism in this case is antibiotic efflux, and the matching resistome is *Acinetobacter baumannii*.

The gene *Mtub_rpsL_STR* codes for the ribosomal protein S12, resulting in streptomycin resistance by disrupting interactions between the rRNA and streptomycin. The resistomes that have sequence variants of this gene include *Bifidobacterium bifidum*, *Bifidobacterium breve*, *Bifidobacterium longum*, *Corynebacterium jeikeium*, *Corynebacterium urealyticum*, *Mycobacterium kansasii*, *Mycobacterium tuberculosis*, *Schaalia odontolytica*, and *Trueperella pyogenes*.

The gene *Mtub_murA_FOF* is responsible for the *Mycobacterium tuberculosis* murA gene. The resistomes that have sequence variants of this gene include *Mycobacterium avium* and *Mycobacterium tuberculosis*.

The gene *FosG* encodes a glutathione transferase that provides resistance to fosfomycin. The AMR gene family associated with *FosG* is fosfomycin thiol-transferase, providing resistance against phosphonic acid antibiotics. The resistance mechanism in this case is antibiotic inactivation. The resistomes that have sequence variants of this gene include *Aeromonas caviae*, *Enterobacter hormaechei*, *Vibrio cholerae*, *Vibrio metoecus*, *Vibrio parahaemolyticus*, and *Vibrio vulnificus*.

The gene *rsmA* regulates the virulence of *Pseudomonas aeruginosa*. However, the overexpression of the Multidrug Efflux Operon of *Pseudomonas aeruginosa* (MexEF-OprN) results in a negative effect, conferring resistance to various antibiotics. In *Escherichia coli*, the homologue of this gene is csrA. The AMR gene family related to *rsmA* is resistance-nodulation cell division (RND) antibiotic efflux pumps, which provide resistance against the diaminopyrimidine, phenicol, and fluoroquinolone drug classes. The resistance mechanism in this case is antibiotic efflux. The resistomes that match with this gene are *Pseudomonas aeruginosa* and *Pseudomonas fluorescens*.

The gene *qacG* encodes a small multidrug resistance efflux pump that provides resistance to benzalkonium chloride and ethidium bromide. The AMR gene family associated with *qacG* is a small multidrug resistance (SMR) antibiotic efflux pump, conferring resistance to disinfecting agents and antiseptics via the mechanism of antibiotic efflux. The efflux component in this case is the efflux pump complex or subunit that confers antibiotic resistance. The resistomes that match this gene are *Staphylococcus aureus* and *Staphylococcus equorum*.

The gene *RbpA* codes for an RNA polymerase-binding protein that confers resistance to rifampin. The AMR gene family associated with *RbpA* is the bacterial *RbpA* RNA polymerase-binding protein, which provides resistance against rifamycin antibiotics via the mechanism of antibiotic target protection. The resistomes that have sequence variants of this gene include *M. avium* and other *Mycobacterium* species.

The gene *Sul1* is involved in sulfonamide resistance in Gram-negative bacteria and is linked to other resistance genes found in class 1 integrons. The AMR gene family related to *Sul1* is sulfonamide-resistant sul, providing resistance against sulfonamide antibiotics. The resistance mechanism in this case is antibiotic target replacement. The resistomes that match with this gene include *Achromobacter xylosoxidans* and *Acinetobacter baumannii*.

The gene *Sul4* is a dihydropteroate synthase gene, and the mobile sulfonamide resistance gene has been shown to confer resistance when expressed in *E. coli*. The AMR gene family related to *Sul4* is the sulfonamide-resistant sul, which confers resistance to sulfonamide antibiotics via the mechanism of antibiotic target replacement. The matching resistome is *Sphingobium yanoikuyae*.

The gene lnuF codes for an integron-mediated nucleotidyltransferase observed in *E. coli*. The AMR gene family related to lnuF is lincosamide nucleotidyltransferase, which provides resistance against lincosamide antibiotics via the mechanism of antibiotic inactivation. The resistomes that have sequence variants of this gene include *Acinetobacter indicus*, *Morganella morganii*, *Proteus mirabilis*, *Proteus vulgaris*, *Providencia rettgeri*, and *Providencia stuartii*.

## 4. Discussion

When compared with previous data [21,22,23,24,25,26,27,28], the ARGs in the reservoirs and sewage around livestock and poultry farms in Central China were low. Fluoroquinolone and sulphonamide ARGs were detected in these samples; their abundances decreased significantly over time. This indicates that the Chinese government’s policy of reducing and replacing antibiotics has significantly reduced the presence of ARGs. Although a significant reduction in the ARGs was observed under the monitoring and intervention of the local government, none of the detected ARGs were completely removed from the drinking water source [29]. The results are consistent with this observation.

The discovery of highly abundant glycopeptide ARGs may indicate an increase in the use of glycopeptide antibiotics in animal and human clinical settings, adding uncertainty to the already controllable form of drug resistance. Glycopeptides have strong antibacterial activity against Gram-positive bacteria and are sensitive to various drug-resistant *Staphylococcus* species, including methicillin-resistant *S. aureus* [30]. With the emergence of drug-resistant bacteria, the clinical use of glycopeptide antibiotics has gradually decreased, and the development of safe and effective glycopeptide antibiotics has become increasingly urgent.

Generally, if a bacterium carries genes that render it resistant to multiple antibiotics, it is called a multidrug-resistant bacterium or a super-bacterium. The detection of multidrug resistance genes indicates that antibiotics continue to be used at high levels in hospitals and farms, the risk of horizontal transfer and the spread of resistance genes continues to increase, and the detection of multiple resistance genes in water sources poses a huge threat to public health.

## 5. Conclusions

The metagenomic sequencing showed that the abundance of sulphonamide and fluoroquinolone resistance genes decreased compared with that in measurements by [1,4,27]. Compared with [2,3,28]’s measurements, the abundance of glycopeptide resistance genes and *AdeF* and *qacG* multidrug resistance genes was high, indicating that the Chinese government’s policy of reducing and replacing antibiotics in animal husbandry has played a role in altering the abundances of antibiotic resistance genes. The existence of multiple drug-resistant gene phenotypes in wastewater from livestock and poultry farms and drinking water reservoirs has raised public health concerns.

## Data Availability

All data generated or analyzed during this study are included in this published article. The raw sequence data reported in this paper have been deposited in the Genome Sequence Archive (Genomics, Proteomics & Bioinformatics 2021) in National Genomics Data Center (Nucleic Acids Res 2022), China National Center for Bioinformation/Beijing Institute of Genomics, Chinese Academy of Sciences (GSA: CRA012181), which are publicly accessible at https://ngdc.cncb.ac.cn/gsa (accessed on 30 November 2023).

## Supplementary Materials

The following supporting information can be downloaded at https://www.mdpi.com/article/10.3390/microorganisms12020396/s1, Supplement Material S1.
