# Supplementary material for: Analysis of Antibiotic Resistance Genes in Water Reservoirs and Related Wastewater from Animal Farms in Central China
Source: Microorganisms. 2024 Feb 16;12(2):396. doi: 10.3390/microorganisms12020396 (PMC10893252; doi:10.3390/microorganisms12020396)
Supplement: Supplementary file 1 [file microorganisms-12-00396-s001.zip › Krona_geneset_origin_group.list.result/taxonomy.krona.html]

Javascript must be enabled to view this page.

members
magnitude

A21
A22
A23
A24
B21
B22
B23
B24

24805111.999968224805111.999946724805112.000013124805111.999990124805111.99994724805111.999994424805112.000015924805112.0000044

24805111.999968224805111.999946724805112.000013124805111.999990124805111.99994724805111.999994424805112.000015924805112.0000044

730846.66621461634631.328895597592483.803970962530503.677883474321840.48341748787222.5245393744281493.381403612557237.864703674

163179.1168254528438.4865756089321388.84077323647477.3169807250876570.734520449320546.762405754264742.764344856127927.500301904

12350.0988414926707.432250539331748.00339564152533.2722868405765015.665136132961484.52309452223639.41670039299097.30909345175

12350.0988414926707.432250539331748.00339564152533.2722868405765015.665136132961484.52309452223639.41670039299097.30909345175

12350.0988414926707.432250539331748.00339564152533.2722868405765015.665136132961484.52309452223639.41670039299097.30909345175

2146.60386565418140.153287305641356.033071082425109.910815170591938.538104253689286.728540991491611.6017001361221539.49407708975

90.13992606444340005.24807622397732004.17937306727849

1051.7403258587575.9263798451805240.56813485826562.6135967825263511.818023937996156.193647569394306.437894219523790.181950985865

848.13547717853350.832365662418764.099133840394342.2565259006703320.522684011977105.777418821757276.476119446661675.194698839835

49.537936684534501.365722489295940.40913893686330.98495187588078.8569685570068230.936959570543164.1556898888504

49.537936684534501.365722489295940.40913893686330.98495187588078.8569685570068230.936959570543164.1556898888504

2781.46827152551167.075924100086390.533991390365115.8738732515711015.79071141869274.104423283916711.6722252462641876.19360749734

207.5340154885463.9502609421537430.30593352533840.60095746281773962.255924598458316.205970073253752.1462614373193111.020306894671

422.75119138534980.154133227094494.217493455834471.0814606135638170.624531924087125.07262603710999.4306437645019311.960769004429

1587.7172805703953.8250469347864177.70564445989151.0337968055128612.144694922014154.247823479684463.2090302059481164.77248712613

1587.7172805703953.8250469347864177.70564445989151.0337968055128612.144694922014154.247823479684463.2090302059481164.77248712613

7920.15749758991824.8446786249881383.45875390987886.0009548625964828.986774155051533.356969454564229.358767242067248.39483076313

7920.15749758991824.8446786249881383.45875390987886.0009548625964828.986774155051533.356969454564229.358767242067248.39483076313

3521.41353467869658.048262748236835.98816639098729.626309731183064.401007205431031.405029665032761.716578698773690.66499921992

3521.41353467869658.048262748236835.98816639098729.626309731183064.401007205431031.405029665032761.716578698773690.66499921992

3521.41353467869658.048262748236835.98816639098729.626309731183064.401007205431031.405029665032761.716578698773690.66499921992

3894.33788383787165.542748023973523.965079590759149.4385652269841564.09706054971453.6928294552991268.700969198383027.6214290055

3894.33788383787165.542748023973523.965079590759149.4385652269841564.09706054971453.6928294552991268.700969198383027.6214290055

1219.4079882808139.0253236319896160.6415546365860.8621224393414505.954650893837147.036422117154410.6668292466551167.00882303445

1792.05167824025120.309813559509273.75578340322882.9758738273261761.746933800518227.698061608073616.0575251127841261.22540560193

128738.0548117566576.1578931593716833.1461716435776.224431834661182.202227659516236.557695958952623.3197327623101585.80890468

23726.33915984691500.818061609473428.241667704761384.5168317504112017.65977691823398.3884136940111235.222675345520216.0014833762

1526.6635744922481.1843693989949194.08671576127285.1221581333885623.219653437443167.856609137703693.2494456799891176.40381315722

1526.6635744922481.1843693989949194.08671576127285.1221581333885623.219653437443167.856609137703693.2494456799891176.40381315722

1526.6635744922481.1843693989949194.08671576127285.1221581333885623.219653437443167.856609137703693.2494456799891176.40381315722

10976.3375771532432.9932268910171194.82081532118413.2488396722694884.186008789151259.132259636174815.225296994519337.21354167604

873.30079288026563.415351367717177.03357828470759.4793460800215292.719458239042111.406211626225172.598153855723406.954908485769

873.30079288026563.415351367717177.03357828470759.4793460800215292.719458239042111.406211626225172.598153855723406.954908485769

1485.6997649145324.502721152578112.45047731463631.9240614796383663.506709203857103.218625716648665.754260495561177.21078923564

1485.6997649145324.502721152578112.45047731463631.9240614796383663.506709203857103.218625716648665.754260495561177.21078923564

1467.63722609276134.691568761961191.556490386224102.729026759274691.995981817959237.21900080944656.4919115860671278.27916245214

1467.63722609276134.691568761961191.556490386224102.729026759274691.995981817959237.21900080944656.4919115860671278.27916245214

1157.0201305185941.04195939710691.882831697275631.8165871275187616.509066557398143.943696675983436.4803750227881075.8404669015

1157.0201305185941.04195939710691.882831697275631.8165871275187616.509066557398143.943696675983436.4803750227881075.8404669015

4834.14038016198153.266676823013498.554192472022175.7394692914652260.86353426475581.8362091980642597.74971539894810.24225310651

2322.7949304376568.9833605315746259.19176818018568.2777298006734988.337915351934184.9401480975911439.333611151642156.06892186107

759.21860941464943.156266131500987.367762969863837.7638825227985434.652092162968133.093177399265456.715818959914810.356013630737

1752.1268403096841.1270501599372151.99466132197369.6978569679929837.873526749847263.802883701209701.7002852873431843.8173176147

9609.78783191122954.8360729649191856.67499322019853.3018612821925833.298932459771824.673995359235184.758782516288679.06302528245

2864.07827742238344.867419113242611.815032645063275.408807959461700.19437563794613.0187507064981468.681162402232424.53387789918

646.041618866544.4730067232249740.45972151193611.0792539336339295.65358550595378.3272448011032321.313136784625372.446422104969

22.11749636349211.722195251861120.0378069153395019.66007605778317.3640647666095222.485947853563616.0685692790004

57.297266048673235.576062639608644.888688547614837.4258219228332113.7598500650640.2437132641402117.639278682651107.337729781633

433.677703177943144.137071108188169.89427569625196.7780935111357273.410929018059134.980332717727218.088684653904302.497419532806

959.02305615393224.967264843789161.707346931242834.5115573725111386.8055333983178.9385570835383623.145891909739765.601690831918

959.02305615393224.967264843789161.707346931242834.5115573725111386.8055333983178.9385570835383623.145891909739765.601690831918

588.080676576892285.486318174393296.988037670245226.034548387761544.075448056058325.071263656203339.916350922746648.865015254001

588.080676576892285.486318174393296.988037670245226.034548387761544.075448056058325.071263656203339.916350922746648.865015254001

549.0713683615116.907419743010773.542774336207212.7714734461791365.79777568916688.529410388001406.670208734924627.517027680388

549.0713683615116.907419743010773.542774336207212.7714734461791365.79777568916688.529410388001406.670208734924627.517027680388

1058.4311096712979.8382399396881153.707107483078100.459974355521925.426669494014217.724549391896635.7591968831451133.40235145291

286.61284400006101.707546918566082.94983464562824167.8571907314630.3062528987587157.306404172321224.726655317775

149.57918911480814.228139075049910.41099281572998.4210115359397298.745723713857221.357404171097692.8499219886978128.789914669899

50.54751091911685.4866404851004113.0627339270305025.112234452944212.528438014960555.01631067624959.8924592575421

1591.0899289159995.1859799499751389.13885766624683.8460909296367867.194350970446213.148852866848724.0937246262171485.59945282594

59.347326637172700.652752724160844010.6714694459962.399864959110850.8697910616886816.94611023319859

121.3715532787518.1818323023427422.50614302726075.1872117843215485.309575902964119.037793563041677.9707345212291159.194432547446

921.84301327050573.7650818646286299.40743590672366.681340732336482.749432247388137.841350621011427.54099613214905.225866254573

3862.62759839125144.371233476203488.904342309703110.4249271465751603.69049143829446.0766667991461511.551888587522715.57169518449

3862.62759839125144.371233476203488.904342309703110.4249271465751603.69049143829446.0766667991461511.551888587522715.57169518449

3862.62759839125144.371233476203488.904342309703110.4249271465751603.69049143829446.0766667991461511.551888587522715.57169518449

2141.4491102891162.1528009937655173.68446121655950.1359480591614771.224385222358247.041922028437756.9231325022461484.13766721214

1370.2791070084772.8158454719797240.31203706245751.5892606918611617.221984100171155.405601031395621.467191814434923.716979291978

451.63238618912222.03729591378442.85988879040069.82891820459086153.31321904145837.466961516409358.003330169044161.168713335218

451.63238618912222.03729591378442.85988879040069.82891820459086153.31321904145837.466961516409358.003330169044161.168713335218

451.63238618912222.03729591378442.85988879040069.82891820459086153.31321904145837.466961516409358.003330169044161.168713335218

451.63238618912222.03729591378442.85988879040069.82891820459086153.31321904145837.466961516409358.003330169044161.168713335218

6357.42030599917216.605142330587850.395464115399196.2096394128323280.41371417048810.9254532658522590.172870139465414.6052080123

3752.99131350184144.124932479668537.988471940793114.7980731182252052.60409595322513.8797403937311432.540426207223354.40920906813

3752.99131350184144.124932479668537.988471940793114.7980731182252052.60409595322513.8797403937311432.540426207223354.40920906813

3752.99131350184144.124932479668537.988471940793114.7980731182252052.60409595322513.8797403937311432.540426207223354.40920906813

2269.7549797246868.4512700821171269.47131774230678.51972884496031130.40667957925264.1723997380961062.178344728431966.20313052251

2269.7549797246868.4512700821171269.47131774230678.51972884496031130.40667957925264.1723997380961062.178344728431966.20313052251

2269.7549797246868.4512700821171269.47131774230678.51972884496031130.40667957925264.1723997380961062.178344728431966.20313052251

22466.62277307571170.924345701682516.269690301531118.5028543813511556.10187301192787.9153371150510397.1526108216324.3521653303

3002.1142223678415.053946004306675.532933479422420.6638527004971841.02764952367492.5579372085571491.311721416822000.83195900654

772.39050925168777.5612552482745102.5009658645571.2466189676213711.723656198804156.691453103302547.989453255883822.322940930809

772.39050925168777.5612552482745102.5009658645571.2466189676213711.723656198804156.691453103302547.989453255883822.322940930809

1594.24268690769300.679868500986470.590131070621309.556445465216774.392071817104267.78780843892682.228807999248842.551601765036

156.6237842175442.1559089593283616.04463901601681.8045701195384754.447199829297511.60251045062654.06591903753273.6666926127415

404.72999995384567.5723746823027126.16089581062561.7452900997304235.06463122028882.3326903046079242.030858606233308.17115957224

266.79290531746847.390187597274558.686335428878130.9187860423941170.43537581873248.4014169075027141.196806912812155.446012895703

22.5282363473070000.98209613548113314.11904987616974.614470474643127.8448190827769

22.5282363473070000.98209613548113314.11904987616974.614470474643127.8448190827769

22.5282363473070000.98209613548113314.11904987616974.614470474643127.8448190827769

2197.59340576581189.875509727876372.487864085495123.5870492364481260.513749678404.1261726271361176.058779643782139.52574518302

2197.59340576581189.875509727876372.487864085495123.5870492364481260.513749678404.1261726271361176.058779643782139.52574518302

1367.65050919826129.30452816819207.11304595217284.1245115090594824.347889730296264.977497471206717.1756530143831339.5752527981

245.70354949077836.261191087238948.193475322752922.322251293491590.10715296015667.0638246277696.5981765762975199.601710971521

12717.7841455736420.328642377248962.735255584761437.2846133479686628.673228933991437.390021655336174.824196821198868.62375670351

11983.0824606071407.322595633046911.512618995796420.877053923986315.090774563271355.757676607895936.493105634428251.7015374739

1075.1949363273984.4256705935224211.21762819473643.7735459278534530.673580402782177.316495928693422.363605255445785.448793026324

10742.4880339221319.801011364797676.597083200687375.3844901373695735.886890044261172.339531939615443.262213109177348.82111274387

586.3995291145949.9466686966925147.13301454919468.13838803520057217.0245796600666.560618944851147.469628894127399.566405501499

586.3995291145949.9466686966925147.13301454919468.13838803520057217.0245796600666.560618944851147.469628894127399.566405501499

1921.24730769154103.885560419178271.91139992110991.5159196799537715.830884365583204.480550121186643.9159148447221432.89392281919

1921.24730769154103.885560419178271.91139992110991.5159196799537715.830884365583204.480550121186643.9159148447221432.89392281919

1921.24730769154103.885560419178271.91139992110991.5159196799537715.830884365583204.480550121186643.9159148447221432.89392281919

925.19727028105417.8623790752923105.07181820859430.7042450743749512.031799978704111.086710451549422.545168323197814.995250463394

925.19727028105417.8623790752923105.07181820859430.7042450743749512.031799978704111.086710451549422.545168323197814.995250463394

203.0910322879464.9090993814056426.37536466341.0219046257131185.138157527071324.788093889661779.9228360758031115.272311575674

178.6573056619378.9225924087242526.660491254214916.2536182851077112.76880808704316.5382781439290.4232172412794175.737543162974

11204.3277894292620.5243378042561597.95663596286520.5718235280325573.092100597551496.864176764414689.100334108659043.77139862274

5164.96661537475287.593596142046819.684201957798250.7906463743152769.38376691086704.4914637885412330.400598909274551.58080583872

2541.76164795948179.2441104018479.815163520235159.1323817669531516.49521339485407.6840069288341212.280313288662460.93165887717

90.107282196647511.738424711320325.242163036505617.8554608238237120.08047155949326.88985794658175.0764065713599134.308385674985

1148.4170125744171.6684555423961221.95145946452774.4905646576265591.202032242929181.467737091999541.3739723677381103.95403221595

2406.7976869511104.876624585165315.90549910911287.55421377891351163.53288828327282.0269116393791065.987183860641937.21197833414

396.1103731946434.720908228854942.221632265396614.7586098273505234.35316962258348.0938728481319383.53610702214338.891924847302

647.85584890252542.46523679572394.453739472827345.6846756820078226.69614792027658.552894414954183.39252767057286.101153037583

4636.93361478163286.966469216323659.64400824774229.2506192765692273.04562267877658.5398460008621910.85983663683462.62436028356

868.02716993618391.4702723910858174.17893636269364.2630305129673521.384571392703135.166943266357486.034933754726718.996045411705

868.02716993618391.4702723910858174.17893636269364.2630305129673521.384571392703135.166943266357486.034933754726718.996045411705

904.56738296558472.6177200857765173.55965247533549.0232236569099520.67944137717142.133693386602414.849991145964732.197263181343

904.56738296558472.6177200857765173.55965247533549.0232236569099520.67944137717142.133693386602414.849991145964732.197263181343

1458.3541436391576.3977725956307135.37041340081868.2219123652763787.710381909918204.055390026632715.211753516771222.58536263738

59.3060484748866001.2744442745962453.21033057335645.9883614862955835.693486954005542.4237776711793

1013.3730807890556.671429505821598.095384722779543.3796764258826516.076777458204149.959118908019497.744130100121806.778218562361

424.33438097521416.564242955388371.427886184216514.3901631856072135.53856509515951.513925896744578.53355893043250.888553002091

424.33438097521416.564242955388371.427886184216514.3901631856072135.53856509515951.513925896744578.53355893043250.888553002091

6300.93251221287846.4249575695151353.80958354797679.4618143105863682.659959130121287.971857136883060.233531333386171.12890544184

1679.7223129784287.377215252571469.958990850329202.8956074813851128.17104491008368.812688187608913.3461349698221504.10427156351

1679.7223129784287.377215252571469.958990850329202.8956074813851128.17104491008368.812688187608913.3461349698221504.10427156351

716.098895763084239.076581221625310.908221199823173.455413322853576.225421591922251.629284222101403.765160872677585.683145817505

963.62341721531348.3006340309464159.05076965050629.4401941585315551.945623318163117.183403965507509.580974097146918.421125746007

4537.18356896456556.223601582796868.307882163289475.3133633050232513.84880390295902.1322949547782102.306918896674592.40983787494

4537.18356896456556.223601582796868.307882163289475.3133633050232513.84880390295902.1322949547782102.306918896674592.40983787494

4537.18356896456556.223601582796868.307882163289475.3133633050232513.84880390295902.1322949547782102.306918896674592.40983787494

3834.45548139936319.526346966567619.658524037296306.1227547043331769.95164667278624.955145223571313.501101375432478.7249711264

3834.45548139936319.526346966567619.658524037296306.1227547043331769.95164667278624.955145223571313.501101375432478.7249711264

929.89629565867756.8012865251525106.38765194586859.8432478850223383.427609386923106.384886105811409.147109486364469.782822836525

929.89629565867756.8012865251525106.38765194586859.8432478850223383.427609386923106.384886105811409.147109486364469.782822836525

207.543800999288194.948137346445248.445680885813176.857711696163167.170372509386208.63710261708432.4565880019368103.300938574723

207.543800999288194.948137346445248.445680885813176.857711696163167.170372509386208.63710261708432.4565880019368103.300938574723

2907.5877828735195.2640343680949446.11845320093185.54082250859721501.60766803665337.6423786655591178.709563278392444.38967065683

2907.5877828735195.2640343680949446.11845320093185.54082250859721501.60766803665337.6423786655591178.709563278392444.38967065683

2907.5877828735195.2640343680949446.11845320093185.54082250859721501.60766803665337.6423786655591178.709563278392444.38967065683

2907.5877828735195.2640343680949446.11845320093185.54082250859721501.60766803665337.6423786655591178.709563278392444.38967065683

8171.46872659952473.3186651342631158.21165030295462.1034945275784104.817808404571093.553288399643432.750178776627261.9873039013

3226.59956313149104.030509978665334.03548643291998.60718511841631287.17585389902299.512540950642996.8236078682122205.65250724622

3226.59956313149104.030509978665334.03548643291998.60718511841631287.17585389902299.512540950642996.8236078682122205.65250724622

2372.5293505371981.1121713937979246.83605215868274.262426486162930.076255082067215.615987936153708.6608599104191625.81595293723

818.18216236640421.538562131513971.139399794587321.7204591792396343.85001781756379.6357746494914288.162747957793574.021774389301

4850.76046696799367.073273028305791.869748533021360.4755382743652807.65254960636790.1554429659392435.92657090845054.26665328158

4850.76046696799367.073273028305791.869748533021360.4755382743652807.65254960636790.1554429659392435.92657090845054.26665328158

2554.39640354274212.878090174155457.681694253913199.1778162954061449.92164587002445.9701111717191246.349949934512422.22646383037

1938.38508694876151.740633163446331.787734060408158.0366439821981171.55823864798316.3294740928471029.601167161672192.83063280021

121383.1565219776538.9999772517217812.18538517575830.197347318158530.809061815816316.324844179752024.5085039836107680.971147243

43332.13653612262856.330431707587250.669944383112738.4343820014422447.07789701546625.4711807610920839.429598750441607.3731145662

2246.18051003848144.774177001149284.208869458791122.8855212646991282.28860084969310.5763229805741072.101520931651830.27651688306

2246.18051003848144.774177001149284.208869458791122.8855212646991282.28860084969310.5763229805741072.101520931651830.27651688306

2246.18051003848144.774177001149284.208869458791122.8855212646991282.28860084969310.5763229805741072.101520931651830.27651688306

716.37286116776771.3319046905253113.70869087896863.2822598900176364.261309320286127.064045777472401.304744891712567.666384906936

643.19682177653745.437929578336982.52371358303842.5655239145841479.771408110468104.122528531238384.652287478249679.45588893037

11738.74210482961082.104287950192659.186428799551054.953072903026559.989922500832070.810089904436133.5806861873911023.8795412281

78.616280377395934.185580900058895.411532815756728.987860214085286.58540353958038.77023314553453102.904594404724125.102251100525

78.616280377395934.185580900058895.411532815756728.987860214085286.58540353958038.77023314553453102.904594404724125.102251100525

78.616280377395934.185580900058895.411532815756728.987860214085286.58540353958038.77023314553453102.904594404724125.102251100525

11660.12582445221042.267189568582536.364654545891022.003133408596465.88024788312055.289553540296030.6760917826710898.7772901276

7871.84314319221804.1503187583681872.75279721642754.5614221798834487.486456483081489.081991688894382.986478751287706.33483990319

4282.1184699706619.2535778146671189.21423947913578.2011920543352395.28306406665967.0723339522579.915437519233632.56884453387

1316.1011649924686.0374734943925323.61572284602656.4273134930418762.097250987874186.821225692186579.5647668488631459.12623808404

215.9913899121960.8448145523950371.574372527694922.16974274272131120.0454251862823.4157771279619121.465231457469235.669751273684

666.54024961367518.926396641270263.432069352007614.5661689827816275.57861804544677.558333381852259.322704140737548.657366208295

666.54024961367518.926396641270263.432069352007614.5661689827816275.57861804544677.558333381852259.322704140737548.657366208295

1156.52279976417157.437435901739340.418417186136168.121343795657868.458355301545268.356947756736683.3368798840341170.20419329413

391.43548506703581.3159350146131145.78242537359983.5769248740236266.00181408348994.4201226262007116.570735699387264.248994124842

40.69607548522026.0230328268977538.31902836763693.5518927445240744.227772068480410.9237201676117.661790401397455.053069548316

128.0125312118474.663644412335345.362437162650433.19069501841361245.18285376351835.0666853078404334.81555379285286.272674770914

14921.4434951675888.27524917962216.20861813677902.3750423318917520.372979275842244.260471021336956.1685601059714513.4430189447

8421.68451938434325.235475208211006.92597315058328.3049326337274141.064696909221075.976132034013645.528999598498359.52607219547

298.7177228472318.156991486152669.46787265622329.16052468214939153.23564497751832.599472324977994.4194194422589301.666936337844

298.7177228472318.156991486152669.46787265622329.16052468214939153.23564497751832.599472324977994.4194194422589301.666936337844

761.7345994926268.1722002579339197.08829125767449.2852227416497400.40488272552184.3935876192862339.222957571996701.6726900844

761.7345994926268.1722002579339197.08829125767449.2852227416497400.40488272552184.3935876192862339.222957571996701.6726900844

769.74619555339537.253221401805980.458431789496436.50499436141349.56237144672394.9151840150109372.238669014236593.516854057296

573.86266957362336.059318367733668.876869293271231.8316731657959230.22184583477477.1706825459577258.332631936371394.346902429186

195.8835259797721.1939030340723611.58156249622524.67332119561414119.34052561194917.7445014690532113.906037077865199.169951628109

2845.37030299391100.000587447143326.91905303406791.52540371008541539.0837725164371.1593598843791272.222650044723210.62795242159

1349.7093924607158.2781029366753216.86813137279439.9466108277866823.966277413707204.731217618755651.2576518276851528.15296325631

673.60530015907738.206663844377285.235531819292537.4571278223675288.12946038922293.3250429479854297.502970242276781.26130528802

1384.5981276201159.8706859872135181.8761693242851.2338602630054576.518743242874188.335830499109610.0559537379111427.72872915127

817.38121243178348.7040652037123121.59321187449339.097655859695401.071948684968137.201589878548459.200975447671968.219739205212

439.68194730166711.166620783501248.94858682601838.55006821710293130.15673367606632.1886694195168121.548591988885365.905624061989

1501.99672982314182.093184644773327.210741581738159.850096565513723.856247473441283.7022503199981216.838123002831686.60000389565

1501.99672982314182.093184644773327.210741581738159.850096565513723.856247473441283.7022503199981216.838123002831686.60000389565

1501.99672982314182.093184644773327.210741581738159.850096565513723.856247473441283.7022503199981216.838123002831686.60000389565

1689.7419281187359.9668541019591166.15401518508481.5430342212748823.625084626686215.149561421363673.1303683847911486.08744180453

1689.7419281187359.9668541019591166.15401518508481.5430342212748823.625084626686215.149561421363673.1303683847911486.08744180453

1346.6052345925145.190724195896297.702501244060758.0954093817538610.670896096542156.994168591379506.9144801940081140.23653996194

267.72121191481612.996112954789956.698377257568117.0004573514377166.35737817783852.0906681236326124.029915094478230.178648294537

2272.0263808772294.749379072719504.046171477182291.6079278186121349.82642829993525.5682874446051074.302643636272082.95394601591

2272.0263808772294.749379072719504.046171477182291.6079278186121349.82642829993525.5682874446051074.302643636272082.95394601591

1367.75594355768103.147762838138238.22919292864776.8033097594486832.845626093337225.825329606608692.1432530009991306.42866556607

409.6501467254925.631582509030155.185190920029645.1622011231095237.82551677635694.6299958202755161.467560723129368.121051005427

4064.76938127251201.990174626139531.524288126749187.7861883057032097.44032191155617.1386730819162198.347905803644235.50344794194

1396.97385942699125.873265922829225.275884768453100.805186624886841.155767847632226.997420545154856.6147132666131603.41668411026

1396.97385942699125.873265922829225.275884768453100.805186624886841.155767847632226.997420545154856.6147132666131603.41668411026

1396.97385942699125.873265922829225.275884768453100.805186624886841.155767847632226.997420545154856.6147132666131603.41668411026

2222.9845200100559.6508127490922248.27446837230862.1838751419421063.05271439036319.8623256088561106.447900834052198.42231432813

2222.9845200100559.6508127490922248.27446837230862.1838751419421063.05271439036319.8623256088561106.447900834052198.42231432813

1003.2419342906117.86935200605387.695362288637429.9452140905273442.028988141044119.524814386408416.367947141007945.732982610751

751.29391644186628.473099021016118.59813504453529.6997325095809445.393183487517139.955084825492488.02569505087875.447222363302

17049.2469400805766.0415538415452258.94706409327611.6784320394587538.521105599362116.856519665766554.7215462069413741.8192364102

17049.2469400805766.0415538415452258.94706409327611.6784320394587538.521105599362116.856519665766554.7215462069413741.8192364102

17049.2469400805766.0415538415452258.94706409327611.6784320394587538.521105599362116.856519665766554.7215462069413741.8192364102

361.5919348711777.231549914883077.027449758820136.47442174698142119.60499185184945.5498558761787136.441088525947262.59054354562

361.5919348711777.231549914883077.027449758820136.47442174698142119.60499185184945.5498558761787136.441088525947262.59054354562

1322.1814714736632.6370810917077130.07707289141925.9004214747264588.165501364779157.859629705185548.324693432466975.957316804104

548.27698883070217.649751514222260.845594578097411.077994661244180.84671976988445.4060306464433156.075796995661258.886018223063

429.7076803374588.9014089635531925.66500441409169.84528424381187276.01412582748566.608545603698233.439379313854421.201643486048

195.82146804329512.843668499723234.81382698066717.0732184305548182.681706333228132.5835587437806197.99990524089399.492237644315

99.28932412217071.2469637466137311.8752126609369029.339052704771813.645711575923676.946006939814727.4540947693114

83.04764601747086.6141786715545913.44859394539176.1255547391883851.222757108545617.9121042281352121.05389830107864.3138890212875

700.70856780226593.8809083887345208.39844801700170.1195762972543465.592415640639125.041883057039335.221189314838813.661408790906

6.187485408592180003.9561469768498106.970622360105765.90724108449255

40.2880994365293001.1199661807057932.0421264404456.8693693841970432.103197367782269.2340588922901

270.40188245326354.714144099057887.751712743259942.602714473849174.14575833306257.7066476319485142.516463686754318.230593764923

56.589693356256812.16602890174445.7215195300359311.975239204213187.774860340456719.690947716153148.165651684190599.0753710515871

40.8806089458673.8027325313399236.41202665140246.5985129417387721.28455863820352.74345765886552.884044046651937.70248551522792

10.79753263346921.9540409548891211.02750970872851.2388452725125518.42997318879579.6892264163783514.319433747758415.9101581772646

113.4040136657578.7569766200924926.52748846731062.7386200053880142.68272324760535.7132127650067725.4731241742123103.948687574081

679.16183852439658.918150021821662.100461510930933.7583647115893207.22311393467184.0836873016676166.123157550207471.893885544165

218.4508221637298.890533590587665.3326066421391310.339372504178238.279676679603810.769483614381942.528060255979278.5474074625337

162.04516041907336.762488991314130.439064127135616.482624630308256.427303452895145.427765460691231.585032818166593.7683199125942

46.62994140184610.720114275959626.32879074165624.0688679592613810.46273101621642.202053650411793.2076197201787513.0097159108739

48.19426708160350.5348216069191930025.67866042313074.9546207134265222.622547338416655.2137359346881

186.5176300926882.010191557041102.8674996178415476.374742362824619.633764557300166.1798974174663229.827994700725

682.32005434153182.6728523152444169.1743629102258.5170407034514368.645568897707148.479976794111329.494411757205647.621850867424

468.01913734678763.5420206852295128.24392330632442.8305738817182281.772887911761112.130314113153278.012439994681441.276553701634

81.406711791333600.688258102462482.7835142733859237.085429383754211.047122706459231.181463667567596.9940974051798

132.8942052034119.130831630014940.242181501433412.902952548347249.787251602192425.302539974498720.3005080949568109.351199760611

333.59680284714216.575642299554226.478280078304815.3742599211185130.74697158333843.2759597228858154.173139065847227.765033837873

333.59680284714216.575642299554226.478280078304815.3742599211185130.74697158333843.2759597228858154.173139065847227.765033837873

1728.3478218291572.4044311105766164.38432020102996.8504620469703923.445015139528295.041157718305748.3760662126651643.73034453633

2.0995903724599435.228968582389442.602681392713643.074382748583852.35490909291556.98407899662757.095993265863062.40539457109553

86.3422216493260.66057286293701918.17352343641022.5127853119518149.06004969829289.5708608746717729.733811972416972.8561763694871

33.42336740963462.62002495075029.703279259155033.7234956224393921.908986006485315.465725934599214.694123857802153.2344725887985

58.8591048951794006.6692722941276928.247455692027913.395243491540432.136490805550257.0939565493361

199.0091498757142.7759788168662822.21869849547493.5060358615345495.148535705644830.4416664522405112.855297268893206.184801889638

98.8401956442893001.3002582865886759.998577416203114.466551739491747.4179585149761143.888901266844

296.24562816903112.88709014797362.624899252476599.8963798941168572.640373633132224.982840373481374.7910210199697169.996767430766

70.522375749299700022.39179188896991.9201907831590821.041985364372610.6991950522329

58.6198402411345.3361520295445119.85926915564914.0806164263583530.267783854059413.310419390872124.528856683719855.6450244825599

113.9261128156014.7924174989379311.31152870306531.7583105865208883.958035813932714.300994381428385.7535108978307178.354583139114

62.52659146027670000008.35874613455696

97.140715308099602.03917793167603051.585519880527.2939302859887828.362804853230759.629855093203

30.69315012814820002.73764061413592005.25450510261809

183.3730433875873.008802846667979.5512463122374078.66731360201711.037319832207470.671080606675184.4169143870911

183.3730433875873.008802846667979.5512463122374078.66731360201711.037319832207470.671080606675184.4169143870911

469.5944856264312.9342767432657434.57498752278955.60312019507947126.12850750329833.900947012267494.0643229975516204.74437488739

469.5944856264312.9342767432657434.57498752278955.60312019507947126.12850750329833.900947012267494.0643229975516204.74437488739

261.67289994008112.177956326395859.60677764755127.70438493053046164.58882064761332.2018953346136142.529122446952324.127162814042

261.67289994008112.177956326395859.60677764755127.70438493053046164.58882064761332.2018953346136142.529122446952324.127162814042

3779.40413855246119.564827487109420.105430845075100.2449141057021910.56315270423421.4451824087031646.096918396783236.11082493275

3779.40413855246119.564827487109420.105430845075100.2449141057021910.56315270423421.4451824087031646.096918396783236.11082493275

2728.7474896425779.4755960950309336.64164034664463.00227927418641448.90119755444326.8060846085521269.109008704272437.56796844318

1296.2284102804330.1932226874041129.18644966827124.247630313119655.06392248299156.476457489607600.24997729131318.14422397823

1296.2284102804330.1932226874041129.18644966827124.247630313119655.06392248299156.476457489607600.24997729131318.14422397823

1318.5892215503341.9946977696197186.92700681065635.0608178002674.65853025801153.280329817756566.872222670624934.980126133575

1318.5892215503341.9946977696197186.92700681065635.0608178002674.65853025801153.280329817756566.872222670624934.980126133575

1050.6566489098940.089231392078383.463790498430237.2426348315156461.66195514979394.6390978001513376.987909692505798.542856489562

1050.6566489098940.089231392078383.463790498430237.2426348315156461.66195514979394.6390978001513376.987909692505798.542856489562

1050.6566489098940.089231392078383.463790498430237.2426348315156461.66195514979394.6390978001513376.987909692505798.542856489562

3125.1845077618879.9475302475231279.45301618273990.99051759665051350.50441682993358.894285861431188.643411290752818.95735089754

3125.1845077618879.9475302475231279.45301618273990.99051759665051350.50441682993358.894285861431188.643411290752818.95735089754

3125.1845077618879.9475302475231279.45301618273990.99051759665051350.50441682993358.894285861431188.643411290752818.95735089754

3125.1845077618879.9475302475231279.45301618273990.99051759665051350.50441682993358.894285861431188.643411290752818.95735089754

947.81658451590321.720222532859580.6130978439830.3039613921116336.67512087281898.3893040683285299.06450950307704.717227694592

439.64961011531319.151388890765448.599619946448915.4290684896026191.71230932113268.0189840880954177.727875238722432.040205465817

91.786680409014601.058972230612871.7819817351560449.256367870285313.943609261748146.9549211246385140.262955527501

21.652341443868700.5194537468058930.46684906058893913.389828561425808.80663449959795.97053295325497

34050.79410034891957.85994063175203.720078845861582.7176470147416361.55951771464479.6119084393114374.147029659429226.1502316693

34050.79410034891957.85994063175203.720078845861582.7176470147416361.55951771464479.6119084393114374.147029659429226.1502316693

3346.5156930296141.714413031684236.882351450353100.3174327659751787.26425262072414.8816521676821620.216121219353251.42440348886

296.183151824727.8972909115288747.554426619938210.4834009861964226.774997330840.351525477597165.848193096138399.69092205665

296.183151824727.8972909115288747.554426619938210.4834009861964226.774997330840.351525477597165.848193096138399.69092205665

353.8191537693173.8995368146703918.05252411689076.90021713564966201.12329946389448.1075337343509198.109207399466417.849833579016

353.8191537693173.8995368146703918.05252411689076.90021713564966201.12329946389448.1075337343509198.109207399466417.849833579016

442.16926004037430.509695794100617.88062498962367.70646510635663261.38394036837164.6108436083339280.218307042706482.60347407381

442.16926004037430.509695794100617.88062498962367.70646510635663261.38394036837164.6108436083339280.218307042706482.60347407381

266.24501431161512.46191325537396.604056871337649.92117685797353129.49452942327443.4176362977398173.832219251756333.416523844101

266.24501431161512.46191325537396.604056871337649.92117685797353129.49452942327443.4176362977398173.832219251756333.416523844101

415.9098853909542.823029305287754.173384402962041.88908723027392232.34048793221938.8461795115554198.393502937328475.859079756792

415.9098853909542.823029305287754.173384402962041.88908723027392232.34048793221938.8461795115554198.393502937328475.859079756792

773.93400638838238.203439136809394.711835628144230.7710354485715455.555584999113118.44579159974440.719507242482738.153451689141

263.26885751219124.132536078076164.476275086702319.8396534289616187.27822444108352.6574927608471158.46036440343255.128700161439

434.46168274685314.070903058733330.235560541441810.93138201961268.27736055802965.7882988388926282.259142839053483.024751527703

364.2549239838136.6376408809548750.626331431737215.7983695313253129.31436408273439.254609084716186.0177508086607200.892546948033

364.2549239838136.6376408809548750.626331431737215.7983695313253129.31436408273439.254609084716186.0177508086607200.892546948033

364.2549239838136.6376408809548750.626331431737215.7983695313253129.31436408273439.254609084716186.0177508086607200.892546948033

3980.42019676298289.463836934542821.980611677102227.028060618881884.27756146146588.1239110955871636.860092869563369.70427820156

185.13350220123913.31263872270637.1948037495145.30944383295082119.87683272116914.914766106658465.4488543881109157.486294206975

185.13350220123913.31263872270637.1948037495145.30944383295082119.87683272116914.914766106658465.4488543881109157.486294206975

1189.16163633414103.728513663248375.68751929474696.5604595475492579.896858513277187.846492203501551.3152023513431222.22856317917

112.74548764943001.4214955370496542.64679404596337.9339457197737530.208512642495374.347001388614

726.09482834872175.1535223631436304.44556884196874.6165036728572377.879408578238149.396725638402417.916522385836799.618990234083

73.647511846826439.181679191514456.787706767291232.305424482458378.699107106967258.03653552945566.3227119484292549.5853244336895

73.647511846826439.181679191514456.787706767291232.305424482458378.699107106967258.03653552945566.3227119484292549.5853244336895

1188.4402662036971.9178186566508198.58342546971570.2496151831274562.185856721028195.514219151693575.641036737575955.180832547734

132.6481922596278.8936173222727176.52349749681382.9597003133393875.592208655599217.6826583140875115.44459801433265.0416542316056

244.60651073843420.613108318578334.168725318390131.0661931630707151.78930098406465.2453742907456145.781638886239227.174239310136

254.48476692548321.263747641099419.21820550205347.46139312364859102.55331132573234.410034295573598.2349850756712134.671588108408

352.16563991676525.128656969887466.44424527179197.09579685448807157.90368753970652.5218599938051135.735107509094237.615310725133

352.16563991676525.128656969887466.44424527179197.09579685448807157.90368753970652.5218599938051135.735107509094237.615310725133

126.227103456672.9529864549080412.67425549391484.954299834645890.210235443152517.894812837622588.7095604063807112.809854573609

126.227103456672.9529864549080412.67425549391484.954299834645890.210235443152517.894812837622588.7095604063807112.809854573609

570.13064768868731.731846410446123.76559561994615.7559268501007248.21054136792441.3169218957725200.680822396351476.253649613286

570.13064768868731.731846410446123.76559561994615.7559268501007248.21054136792441.3169218957725200.680822396351476.253649613286

570.13064768868731.731846410446123.76559561994615.7559268501007248.21054136792441.3169218957725200.680822396351476.253649613286

301.53457135285217.693254214224477.39481116650857.7775318560244679.456290537409130.335101373352346.5714965241239163.702059532491

301.53457135285217.693254214224477.39481116650857.7775318560244679.456290537409130.335101373352346.5714965241239163.702059532491

301.53457135285217.693254214224477.39481116650857.7775318560244679.456290537409130.335101373352346.5714965241239163.702059532491

430.00122028340814.018289210836271.247637648267312.3279353979584196.1314516747446.7643381753044157.604867454643349.014222625136

430.00122028340814.018289210836271.247637648267312.3279353979584196.1314516747446.7643381753044157.604867454643349.014222625136

430.00122028340814.018289210836271.247637648267312.3279353979584196.1314516747446.7643381753044157.604867454643349.014222625136

628.7077065600086.1401583697447948.341048217858313.7850169204759221.05636012766653.6088192690934169.754140303685523.77292908764

628.7077065600086.1401583697447948.341048217858313.7850169204759221.05636012766653.6088192690934169.754140303685523.77292908764

628.7077065600086.1401583697447948.341048217858313.7850169204759221.05636012766653.6088192690934169.754140303685523.77292908764

1170.48575020071134.040681786961480.26449556276349.9115224817175776.256553155839205.534269623732579.9182029313081166.37091084951

426.134868990273015.20634723867631.85567618225729145.23920378986325.2546311382699135.700196097744341.426820338212

426.134868990273015.20634723867631.85567618225729145.23920378986325.2546311382699135.700196097744341.426820338212

490.838443016204115.873923021126377.28306211995439.0810566814492379.466147083367141.042228797649287.093448878525488.267596598429

490.838443016204115.873923021126377.28306211995439.0810566814492379.466147083367141.042228797649287.093448878525488.267596598429

60.07092662314682.8927024588064215.0206342997156.0715888754189949.24412427934894.486427063455839.945357730263342.4147043965443

60.07092662314682.8927024588064215.0206342997156.0715888754189949.24412427934894.486427063455839.945357730263342.4147043965443

4074.93462039616413.714629102233621.646821119733366.4760022669351881.2949273887669.6447921077292118.637468323113275.79949900441

313.2881124487059.87863650603569.30634138210125.0965451251339253.31397400614127.7342996059816125.1343064777342109.118176759163

313.2881124487059.87863650603569.30634138210125.0965451251339253.31397400614127.7342996059816125.1343064777342109.118176759163

3551.04183937697398.969486751068537.995305969756355.6156657567011735.88650281126646.5786440980932039.183214132063006.57211435975

11.5581410602745001.09778863257313.85630686198634.752947483067039.7657729104451826.4831560698834

380.314340809209249.093430829357217.253113936553219.936002811423326.891464003396266.671900912995443.540302334235352.139634610068

393.5830750591354.3843571165828410.2103140619435.17296033736612254.59322269085250.6311845483542266.364717583238529.168242813019

350.55042820164827.312368192778864.783964258961639.5142984248015148.37078828273351.0818278857931190.933064261211299.826571418488

6642.59452550686486.643445120278988.213948454654461.3441978477343564.17769259957986.1818136095643046.449257258035947.87845359588

218.2644637540275.8200960246457116.14995929120739.0573546777245992.500282385167117.654257959103598.9774907235077145.028600073613

218.2644637540275.8200960246457116.14995929120739.0573546777245992.500282385167117.654257959103598.9774907235077145.028600073613

669.02294361387517.578938585746657.953911162317810.5686096810735208.9783035544141.3013391411792170.664744655558376.557147572512

669.02294361387517.578938585746657.953911162317810.5686096810735208.9783035544141.3013391411792170.664744655558376.557147572512

159.0975533963865.4622380533719410.6124959024864.5275583943889590.858850460492614.225621358199974.9346650513957172.536618087192

159.0975533963865.4622380533719410.6124959024864.5275583943889590.858850460492614.225621358199974.9346650513957172.536618087192

594.08581232035756.8288213823362218.61988939645439.2175308099587352.54825835111697.3437347743854417.618187798496542.814572111124

594.08581232035756.8288213823362218.61988939645439.2175308099587352.54825835111697.3437347743854417.618187798496542.814572111124

1764.96668369753273.790961384326303.020605037535262.1850115540921038.89468127306395.009694881321645.8699845236351567.51732214674

74.272159321697735.497215233794327.189353562960432.844093479482163.369188568713131.206125014163826.867677406345375.2475830646718

34.8004686829302191.195098813308189.355819129355182.739308326527136.225794508128196.40442808692724.929452733074630.2016731537025

597.87044040071213.742800673955747.30435256992559.2283233808545467.525456167299100.191517159795210.148230158967641.233542837589

239.39668392530310.731652872558936.809142326580114.3167608253621115.64306691248337.93873892503192.6291626901448308.54614565073

239.39668392530310.731652872558936.809142326580114.3167608253621115.64306691248337.93873892503192.6291626901448308.54614565073

13.969580098272518.018626138568416.823217936327227.21517819115054.8272426311345717.456279846900702.41407830600923

13.969580098272518.018626138568416.823217936327227.21517819115054.8272426311345717.456279846900702.41407830600923

1580.6499124201261.61332655968173.1502655230859.1484961033022927.969855178329199.180333481424925.7994956832061506.00648296807

53.038207494370615.373015251790238.571285121555614.822570221688349.597746847461817.604806994762264.34171755384657.335258505662

123.77479404605400068.86423305356449.6783809635034355.680656836167272.846185244579

232.1235815820235.530304813229799.624976218199935.9885839327321192.88236383722259.2175466303968231.437927414864215.846122535989

98.23263702894791.46125657884131.725458249879720.38768060101354370.570416319095415.415420057107653.800021532391189.4265587220298

131.15514949814700.785797865391084.23732427604609111.65956349819116.2677715959321128.029667803789105.746789569715

234.6789801260994.179571206758622.00602056693333093.920427919107921.296350276504189.1865035213748215.045449688486

997.23823241222537.6800901705819108.91167775755324.1676846149717240.0919291431391.428246144006207.423090595637438.123307326876

390.6162090678083.2938504953068802.2627888140790464.219952829148818.5356806962118118.028233262405203.809727640008

390.6162090678083.2938504953068802.2627888140790464.219952829148818.5356806962118118.028233262405203.809727640008

563.82362562167831.357899147784698.23026954401215.185098716658172.28354812664767.710565401667689.3948573332324227.169352221434

178.45490238954713.056459960949732.76410913543527.0726529090749468.461845431885315.569768392209325.1552346042669121.057485165753

149.13109138974515.005577411332843.66929382440735.7617040714019957.876215839959349.596374593611449.714782099736680.497800037366

66641.73807002813762.62195980577996.306475445293439.5954206413729926.29848764788462.7622810182828016.193094295748520.8930244864

1259.43798450983148.156525084816235.551847510446124.707013645377814.56925785438300.839239735188803.7766566442381295.18173397527

1259.43798450983148.156525084816235.551847510446124.707013645377814.56925785438300.839239735188803.7766566442381295.18173397527

149.5692370075427.951997581867722.17284760338585.4463712655189543.848788433660718.904867251055933.2167006176269202.395329622794

149.5692370075427.951997581867722.17284760338585.4463712655189543.848788433660718.904867251055933.2167006176269202.395329622794

149.5692370075427.951997581867722.17284760338585.4463712655189543.848788433660718.904867251055933.2167006176269202.395329622794

33.0836123915566000.4949850530797910.04096216322191.8751863116787914.310781061846635.9298143794093

33.0836123915566000.4949850530797910.04096216322191.8751863116787914.310781061846635.9298143794093

33.0836123915566000.4949850530797910.04096216322191.8751863116787914.310781061846635.9298143794093

129.58496838904232.670839187868670.49030726917936.3741568149491145.19048607025862.4293670181085143.09913832713796.8764224274307

129.58496838904232.670839187868670.49030726917936.3741568149491145.19048607025862.4293670181085143.09913832713796.8764224274307

35.676053343809400034.0818750212635.1146634254617721.518392567256914.2493084790012

14.653662288325321.112089168425130.903695195188821.741639447898637.238567165241431.660121279287624.357816091788121.8687609187906

05.910148448812087.9593719268644913.7957124471275.804323934352787.7427047708027300

137.05767954147540.688088228582452.270093655936122.2700608849482119.03667175572965.247191114444793.360466021273191.27314526109

2.833427784193517.77274068722569.399639227916155.2798405661844117.48226470295784.6162700288614019.4766900222686

2.833427784193517.77274068722569.399639227916155.2798405661844117.48226470295784.6162700288614019.4766900222686

37.177287570076410.52406728445499.490020374338432.8429910740993223.14301033154928.2421218954781230.239057144661623.7920163294861

35.908404692807100018.2752294861210.73853491659964520.232678234973619.4309313897172

12.16012757383058.428273034341011.486388733330125.3434531031264113.849090035495710.550747078963823.117415561444520.8968653363924

12.16012757383058.428273034341011.486388733330125.3434531031264113.849090035495710.550747078963823.117415561444520.8968653363924

31.7848879157552.792601444512194.432464605978422.655728188979011.498148402150200

31.7848879157552.792601444512194.432464605978422.655728188979011.498148402150200

13.8625454341667023.02911610839462.9567107170632730.6332152925495.000496831143426.849604610798369.10174579096205

13.8625454341667023.02911610839462.9567107170632730.6332152925495.000496831143426.849604610798369.10174579096205

371.51070922357951.383632520021253.184223113854537.193361122287236.77732375472875.9544386367104251.474711833875326.189810852806

60.9471498200971001.7101797206664258.23222992531996.9858725923510860.666896379235373.8882477730534

11.596413050540300033.36525281469024.7686857197659434.837724113199519.9281364797385

49.3507367695568001.7101797206664224.86697711062972.2171868725851425.829172266035853.9601112933149

158.8943358194846.209276217235836.616185323805330.2225280401615132.59780476211660.4802143670203125.72427255849179.885630291598

158.8943358194846.209276217235836.616185323805330.2225280401615132.59780476211660.4802143670203125.72427255849179.885630291598

12.507559790225607.2570744039058603.99853426588747014.090615199356614.3292790878119

12.507559790225607.2570744039058603.99853426588747014.090615199356614.3292790878119

27.19684446620140005.51769065364400.5892132998536231.79757981388322

27.19684446620140005.51769065364400.5892132998536231.79757981388322

270.75852721573415.461967566475935.438639070297822.9280785045943162.97792209099752.7357090663292179.67957823896322.017361191391

129.26669961976611.052541220648216.80782946686715.900149132215885.68502543676724.450510746567101.90322392097163.183362446444

8.2308679790633500023.0353378820184040.446335687292812.4471900537158

01.480522035662027.049729420937137.039787421579226.21994219138053.8099023475378500

9.1200956803728600013.1201905599433020.548813832395136.5695143386866

02.3794104144568116.8567136415265010.553622670417310.005971757688700

02.3794104144568116.8567136415265010.553622670417310.005971757688700

01.5340935566892633.25291030689246.056659214079982.901647673012443.6367249681043100

01.5340935566892633.25291030689246.056659214079982.901647673012443.6367249681043100

01.5340935566892633.25291030689246.056659214079982.901647673012443.6367249681043100

01.5340935566892633.25291030689246.056659214079982.901647673012443.6367249681043100

01.5340935566892633.25291030689246.056659214079982.901647673012443.6367249681043100

16836.9719753344703.038900490031736.6911202796672.3394968042997668.632747926672009.898070319755726.9597610316912276.9862722055

16836.9719753344703.038900490031736.6911202796672.3394968042997668.632747926672009.898070319755726.9597610316912276.9862722055

790.01948433778335.548161066726871.248619362955215.0870649871139325.98520127554378.4919010562836395.355658233739505.458180030507

404.11497802691225.889861439535161.54534010380016.00085826833706190.85217745741446.9876331734537237.369277239834308.773602329528

180.29237884735119.70553977043113.475218728630973.12328596872881119.79860226003221.8970003989909151.782843619264176.433364667663

34.04835720672540004.664956643535388.000794929829527.398418443193427.8624871151898

65.93132292522022.232080641701219.32527570913861.3859581486234135.93450198543945.4872561572314933.861168308531551.0966541115774

153.358246758768.384079842400639.703279259155036.4781864025543753.191483455257813.180300923728860.52103148085459.6240884912396

153.358246758768.384079842400639.703279259155036.4781864025543753.191483455257813.180300923728860.52103148085459.6240884912396

155.517421954165000.18448694158048757.90897688349746.5195154232870681.065991161616472.8452884392488

18.623008961020300023.07850360498971.8238894216936625.430693267157431.3915339975395

39.8895534542698000.18448694158048712.81977585460250.5990611844298327.713342793115325.7816063573568

22.44946629014860000000

867.7003061900772.7512723375786853.81355622723199.70418951410219730.600676826369126.975464773692217.6942658209241041.56116859424

38.245338380778900019.459533427319015.781489023279416.5582780570271

38.245338380778900019.459533427319015.781489023279416.5582780570271

202.0461534396350.728694439427418.50553972995992.28611653381181117.75697719407323.3863041644091152.772792694156284.399345960656

202.0461534396350.728694439427418.50553972995992.28611653381181117.75697719407323.3863041644091152.772792694156284.399345960656

22.242558766737100018.0578966846531007.34834825015998

12.82826645151350000007.34834825015998

9.414292315223600018.0578966846531000

215.756584544083000264.66218435847732.39076148062461.86807398476319308.008006746009

215.756584544083000264.66218435847732.39076148062461.86807398476319308.008006746009

389.4096710588432.0225778981512822.49032664102277.41807298029038310.66408516184771.198399128658438.3859365695809419.222868041966

389.4096710588432.0225778981512822.49032664102277.41807298029038310.66408516184771.198399128658438.3859365695809419.222868041966

0012.81768985624930008.885973549143836.02432153841943

0012.81768985624930008.885973549143836.02432153841943

1049.3387217525637.312067304046361.423923802468226.1686181177961314.736370846828145.968008239318260.646044607136485.869649900845

332.13597074018925.051084759816654.66611937995522.8979204219297182.336898143182104.2078908227146.503184514927303.04680454505

34.39547022683500035.92374540584413.6525368157917229.668773321862230.5703503130856

103.9365699002921.676452364233235.184635648205121.541795238263562.970827585806367.370934166319331.2932916532554127.495643206023

44.77715399909611.387989408433144.4060808880857013.16798857270913.612895949397419.798275703111612.5527836687382

51.96301101811911.9866429871503114.45232069787430.2361590029603267.322633043138171.6122049897459315.872187236056914.9739327517642

404.582199573300035.3721469767113.993211151859626.302481705465731.8510082521693

245.2830510975311.94318517180640.207170889784046089.42803096179215.911978984590987.8403783867429146.572497032807

139.85786102882200.207170889784046040.2878394968763.9107865898467143.284261836500662.5282736033817

86.28794011645051.94318517180640038.816107089878712.001192394744233.776410933248259.3790709012244

19.137249952257800010.3240843750373010.779705616994124.6651525282009

1760.6179433459691.1520515951249270.73823354445579.1100563460979770.770387431848226.043461435582611.8836183617021333.52901979923

666.88450274484349.3691963213062164.53787747014535.4899246545325333.07447886606182.1925820610342257.221372723274543.161830073153

46.95465938351355.11364518896422.164390611691228.6874807531910552.036670425585110.919907442140254.39308318523768.6341237765433

354.9061119738844.0304370289011740.21415488246752.66115857981862132.45090675012736.0644525176988116.382338941116299.543662893838

0019.40655851831012.491610154828619.43474377344236000

14.66197200289610002.678444005857640.57421973180594502.39964003862879

75.83924974789934.9613238429099539.03294130789220.89416654749897453.996229548993112.887332301518845.06230474726591.3538655754963

164.8588320945341.421842808638832.073449829687391.8634731410062763.5268774229228.7859711282717928.319499307206679.0163004020096

49.67541476969040000000

49.67541476969040000000

422.10184378252913.630271182969833.175133055374117.8381054156965148.82060555689871.2369828218295191.891895818019356.04998484002

81.74145136583452.7114211699624213.5925513305488029.476427139045314.832090011899739.423181432107566.4119555896306

107.8518352048738.327936450598845.874774517447615.2798405661844122.803660477922912.709858193036356.439593212330973.1083624179699

232.5085572118212.5909135624085413.707807207377712.558264849512184.371065826359743.695034616893685.3080009132005212.895429382613

490.43466505360128.15258409084971.712773392697924.6024874259766260.09395966783872.6138965527187140.811580847124385.907170359501

24.62410394229397.28694439427421.8564801948462024.92120157713396.776520348338285.5102964466757915.5769557218898

31.98280129007481.143050101062599.67609920520781001.1765874896808100

55.033263076992914.21578752117226.8000515039456117.237359488450635.721905497872114.084256503260625.550982228166862.2845997683559

84.154391654759300035.85498832266282.036228334653728.7545242362866723.3378729750147

208.02583492508520.779976838508320.451619275576331.515653210828981.014152236514734.880544039878951.5090477581441127.285048611804

208.02583492508520.779976838508320.451619275576331.515653210828981.014152236514734.880544039878951.5090477581441127.285048611804

208.02583492508520.779976838508320.451619275576331.515653210828981.014152236514734.880544039878951.5090477581441127.285048611804

561.66218633025448.860938599835491.903424704669943.1794920187265124.5460095181168.1580789956007216.087929693791219.4117390504

146.0817054512617.99481899257494.229837652562281.267161735884261.33922200292882028.308172877986812.7980802060202

146.0817054512617.99481899257494.229837652562281.267161735884261.33922200292882028.308172877986812.7980802060202

334.22491906743740.331298000341387.673587052107641.912330282842287.898629433376756.5972973309388165.911294388668131.601443165769

17.9595730321188012.986343670147302.870742549867943.1582085249327100

61.278075448869101.2214877709544504.15689205859589008.27598627183858

01.160110550332187.365388947247758.826002140487391.392524371204593.5824454909684400

29.005756684790600031.57655792448181.27189893866905112.73345495574327.8830039869688

88.98331816719881.3439897441888618.50143741567717.029643635669185.145414045027710.1753684685109.16026973650079

2342.17653143061142.223127215022310.036335067459108.9656980714141402.61669298901317.219299296265865.7937446593491983.81271697373

64.33711996887243.9856682198987517.36736193780654.9046576142098217.880435328943.38061757598432.283201536932792.32187392626582

32.42700686354790009.7186596740320402.283201536932792.32187392626582

31.91011310532453.9856682198987517.36736193780654.904657614209828.161775654907963.380617575984300

02.3759469349597917.62824711097265.540509807597378.181948102250082.7950375300714500

02.3759469349597917.62824711097265.540509807597378.181948102250082.7950375300714500

117.5967945208049.5125784006490214.48209062054617.142949006003369.344816110024318.676603113975737.3681217360986112.810639367568

117.5967945208041.0332249236756512.238994895702413.111070755462468.072555207241913.221515661819337.3681217360986112.810639367568

77.1954175237658001.3688475541959626.78030665733285.1857004174820928.413174681830184.7506901342494

77.1954175237658001.3688475541959626.78030665733285.1857004174820928.413174681830184.7506901342494

114.40580577126316.13595713616595.85512644213849.7473979683404746.898612335877914.915619542860827.551745795652159.7897783720584

60.680240566441200030.48387509636987.1294212246005522.786803457705449.6559176310316

53.725565204821516.13595713616595.85512644213849.7473979683404716.41473723950817.786198318260254.7649423379466910.1338607410268

008.02408226773332011.995602797662409.393743466237760

008.02408226773332011.995602797662409.393743466237760

185.5608758072477.2134545792284935.074283812153412.357373643417561.113255571100315.882887348995832.8893835629778118.024209994859

70.55735199309641.3976956280725313.06418389194642.5963523679306913.26506752263653.11719282980375.6362460797426438.4747267131292

115.0035238141515.8157589511559622.0100999202079.7610212754868247.848188048463812.765694519192127.253137483235179.5494832817301

1027.4137182009142.1610098655272129.29028844398128.0788470137733863.38728549624156.357057700496383.9891284084761051.12704278004

131.702419233763000.93963264313451756.03322573528318.9696472812027158.36669242781109.793134584278

44.877786256227701.442927074460820118.5765736731927.01824116651711117.91675390740785.2310626695671

2.1148047954487800013.183573123034808.3386490914066910.9027123494221

37.415777150247706.96445043600644015.279758733168000

21.48216433150660002.512543973178840.86184505527786303.00134511115152

82.54147201630380004.62640328284502005.52644372532692

451.63147982684121.95366183186424.719592790370815.6637554068124537.630935910291102.11728954903514.5918057806345619.33199671065

000010.04116228204933.22902037526754.423063067062610

22.416930831757105.33493037260107001.600158985965900

30.809912033082300015.549855478451313.334658216382518.265612295462325.5467073462109

194.15487976227714.71294118761321.989842981716122.19557726514692.719725922531825.5804297717908106.409069955522252.464085436276

73.12202645617002.19557726514624.696119069441713.291034779515732.261656958066541.6800522503512

17.120336783096800011.54782980521531.8862384903330921.961797101813212.4806425584741

17.989255164567314.71294118761321.98984298171612014.8062679870194018.9205020152356111.457007610885

85.923261358442500041.669509060855510.40315650194233.26511388040786.8463830165654

65.67306556521085.9803198821972825.95029709258750.7646665647577454.288014701674845.48330342173659013.307456532628

65.67306556521085.9803198821972825.95029709258750.7646665647577454.288014701674845.48330342173659013.307456532628

3598.6808345425118.704570091157347.757355665861140.9425526352681475.04502513301351.5634489402691069.023766849652322.97070855056

328.5380393652067.8336649626134233.5572609892818.22718870496149142.58020224848748.8420603857647116.355889955419277.518248315689

142.211398988872003.3598985421173673.622309779202915.321308365625482.8106208775024162.640277791512

01.1806694714773113.11785094781970.5614007690626471.417202018289235.4688978001366100

80.302993132759703.33433148287567022.69438367125329.7306965362791313.328960323715733.4061447010759

22.21642684769030001.937006218769021.6610646913140803.4707596406465

9.371107488089530006.41966510578264007.66857443537336

19.586782669257110.599191846216716.823217936327202.12043483797063006.73187608152238

19.586782669257110.599191846216716.823217936327202.12043483797063006.73187608152238

46.14739415554270003.86694498241554.3730895021866304.6192371325469

46.14739415554270003.86694498241554.3730895021866304.6192371325469

37.32258450450144.1639682252994328.171410378315613.199601415461131.357268275318312.858420422940327.819932573088822.9281646298713

14.395846096373300012.5514528525616007.4966333045354

04.1639682252994311.749549034895213.19960141546114.9981678323593512.858420422940300

2.2449466290148605.69401222460305010.7652845620047027.819932573088815.4315313253359

216.0571455619122.520370948532256.4931718350736612.122862571301487.670191133060218.899786651887851.8904557899488139.982435097239

92.48547732209100051.248819464191613.522470303937227.784311660703275.3464440298092

55.21355222712212.045458075585686.4931718350736611.67122651472352.45524033870284000

57.06096523443050.47491287294657300.45163605657789333.06323649593315.3773163479505924.106144129245659.2432516257805

629.99654630099519.58100878882129.864898517054528.2105538746076255.04862963709886.9485434567005273.366750263637600.463433889634

03.480331650996524.603368092029834.137188503353473.1331798352103410.747336472905300

48.768977894778900024.72763599763651.9836681644205463.05560958582745.3000653738209

369.2847692570577.2233135103182439.79032701315173.622956411300768.59997966683298.5197186095460763.426556535654592.1597841962566

38.4463913711526000.44262136482983217.87767815486624.3118056508063118.374987219387238.7071677288866

05.6188486895606614.86388733330122.671726551563193.3722578146039000

8.877355491161410004.2569946556976901.2501179517806922.8832593797757

0011.34439217162300000

20.184267887333700011.44919433967782.5089600825249216.038098600893715.5756502836669

37.95931715614651.6044648207575813.58204750822750.5086084949076748.206761067662660.5505134126029488.885973549143834.51824115381459

265.1527047803633.3777466402382930.08746020059677.04551697554229101.06620401012310.6950180737329203.281367085165202.134869059144

200.1728806479313.3777466402382930.08746020059677.0455169755422983.39888657899969.734612992991185.58493817568180.815359388585

46.010025117255900016.26783043806280.96040508074185317.696428909484716.3042619898249

220.3384845571617.8380578358577515.89644869427011.75056793409034.07143792700077019.8667934375224

25.54043364762260007.62324118283221.38209509344464015.4547141776959

194.7980509095387.8380578358577515.8964486942704.127326751258062.6893428335561304.41207925982647

226.3346615804038.5131516216777725.14376669988668.41026227605634109.44081340975627.97159497429263.4273386959929138.131881408799

226.3346615804038.5131516216777725.14376669988668.41026227605634109.44081340975627.97159497429263.4273386959929138.131881408799

336.9607863300021.21449073237911.55930374611788.51205779746664383.10764791901963.614646579865584.4857202311138410.704409859462

89.90618116650671.21449073237911.55930374611788.5120577974666418.23085143345310.463635109665812.558785361139136.1449254905956

119.58793630065700087.097059865557219.073274799152971.926934869974788.9442675798116

206.8569852939314.6949440392637712.479577059065714.273736512479790.148611065026810.67386588794674.9662175912683161.202123911135

2.0995903724599400022.15015384700250.86339513631253620.105314253278618.0404592832165

38.71387554117450002.85609590420533000

46.2235905290947001.3773497129176848.67780845428238.1995724436140731.652831244931779.9532238957623

64.304403441273010.6124959024861.1922220633319715.68533081901583.0002980986860513.609378341455110.4484326681962

64.304403441273010.6124959024861.1922220633319715.68533081901583.0002980986860513.609378341455110.4484326681962

64.304403441273010.6124959024861.1922220633319715.68533081901583.0002980986860513.609378341455110.4484326681962

555.36800670470522.965814142827338.068279640047816.8117693192452137.74183938289438.8744660193855192.993966256234215.735485709431

145.2494860867149.7926326617086318.19505499179236.9906440538893355.673503697775312.379075889844558.423142552752672.231313225522

145.2494860867149.7926326617086318.19505499179236.9906440538893355.673503697775312.379075889844558.423142552752672.231313225522

81.2959032991126002.9567107170632748.75896801883475.9912929474537368.260197243095379.9819398914194

5.980390610080570007.32882532795859014.69733011205911.54791595084388

267.704615216451011.51455805419731.7392415982725229.073271952454312.328534795441866.310626460385654.510051019021

38.268636776466500012.00540312674553.5297624690424240.291791828225620.0773804212398

5.2248118475793300012.16945211357063.0693586687325513.453954836555411.9716057170125

165.9405764737451.246963746613733.958404220312288.1920215354626378.957601885485416.390893456731265.3873865211394158.497807088771

165.9405764737451.246963746613733.958404220312288.1920215354626378.957601885485416.390893456731265.3873865211394158.497807088771

165.9405764737451.246963746613733.958404220312288.1920215354626378.957601885485416.390893456731265.3873865211394158.497807088771

854.05362918532618.541277150543429.352782496919320.2088943135575307.30449570269297.8533743242458209.451467435468548.192944311717

280.8990746384475.1818271248170614.11585356145784.4156140314037107.79099455516628.390436611649772.2424474889231192.433110562513

280.8990746384475.1818271248170614.11585356145784.4156140314037107.79099455516628.390436611649772.2424474889231192.433110562513

380.8223670726481.3636387170571202.42795588080014115.02642423643719.953067084600477.1527006912764205.690335566711

380.8223670726481.3636387170571202.42795588080014115.02642423643719.953067084600477.1527006912764205.690335566711

192.33218747423111.995811308669215.236928935461513.365324401353684.487076911088549.509870627995760.0563192552684150.069498182492

192.33218747423111.995811308669215.236928935461513.365324401353684.487076911088549.509870627995760.0563192552684150.069498182492

724.85560086976924.603476768060447.343853431802730.4405572878417223.26115932079643.3446635085232180.563531493069268.064213339475

724.85560086976924.603476768060447.343853431802730.4405572878417223.26115932079643.3446635085232180.563531493069268.064213339475

724.85560086976924.603476768060447.343853431802730.4405572878417223.26115932079643.3446635085232180.563531493069268.064213339475

85.14812755071971.8506525445775216.6317817388928038.03263322420574.1294425444281225.41242302662842.61256721488

26.15081198673231.8506525445775215.666065379860308.02573185984582002.39677308517762

45.263524421778900.965716359032481023.42108022054524.1294425444281217.676398995608736.2822665369697

13.73379114220850006.5858211438146707.736024031019343.93352759273269

13.146083863600500015.1295891141688006.02432153841943

13.146083863600500015.1295891141688006.02432153841943

1.5482390544930006.681900762623641.2733360631028416.56986579588367.98182654758754

1.5482390544930006.681900762623641.2733360631028416.56986579588367.98182654758754

133.8145772767616.3754445290018114.148358699129312.125932102638168.38167783122588.3245265165856139.284887693201150.9275544626446

21.401824529941600013.9948699306061016.439051065916142.3509804150888

112.412752746826.3754445290018114.148358699129312.125932102638154.38680790061978.3245265165856122.8458366272858.57657404755584

38.348300880547116.37737969448116.563712993780717.784114889079816.412527975451118.169136956257222.171874721434242.1245161360324

38.348300880547116.37737969448116.563712993780717.784114889079816.412527975451118.169136956257222.171874721434242.1245161360324

6325.45315240149441.962671034499613.571337590882437.0959288335362770.2560159704980.2269429941682455.585815461974949.41378598501

14.26300677832750005.261229297220362.7070358785137500

14.26300677832750005.261229297220362.7070358785137500

14.26300677832750005.261229297220362.7070358785137500

14.26300677832750005.261229297220362.7070358785137500

276.129943420903114.465770269213113.121784024752121.880566399377134.623072795944156.707023737039236.374113104856241.011197324656

276.129943420903114.465770269213113.121784024752121.880566399377134.623072795944156.707023737039236.374113104856241.011197324656

276.129943420903114.465770269213113.121784024752121.880566399377134.623072795944156.707023737039236.374113104856241.011197324656

49.725311949704700013.6013144930291.0213780761484524.12124018160549.93707702368249

106.215258705797114.465770269213113.121784024752121.88056639937759.486616183023140.71497749899492.8433808301841110.712255191419

1.6395677627636600011.5026328196763015.358633210729715.8503543755182

1.6395677627636600011.5026328196763015.358633210729715.8503543755182

1.6395677627636600011.5026328196763015.358633210729715.8503543755182

1.6395677627636600011.5026328196763015.358633210729715.8503543755182

532.36028715330832.049937249274331.109601421938418.9594274876622187.06912516285780.632299324147623.655642180403652.2743058352563

532.36028715330832.049937249274331.109601421938418.9594274876622187.06912516285780.632299324147623.655642180403652.2743058352563

532.36028715330832.049937249274331.109601421938418.9594274876622187.06912516285780.632299324147623.655642180403652.2743058352563

532.36028715330832.049937249274331.109601421938418.9594274876622187.06912516285780.632299324147623.655642180403652.2743058352563

1018.1498426146119.048139820467646.221709796851950.2681800109289660.547706154133169.353290531174455.100471818122980.61929406055

548.4112689578632.020679639220247.7152352818545922.3071974924954461.87369283465195.6293366165629223.206752084778699.645720299781

110.743859939403006.61950160536554116.25758899868819.997969836135985.5492851015155182.105833266887

1.0849184452488200029.13430171427312.676845887303548.5556399599563117.4011071946167

109.658941494154006.6195016053655487.123287284415417.321123948832376.9936451415592164.70472607227

126.131512730081000240.38802352007453.03309241540020314.979791369976

126.131512730081000240.38802352007453.03309241540020314.979791369976

120.0187347465980000000

120.0187347465980000000

102.59003827156700064.65240387755641.5485409541605573.6833604333332127.176438134722

102.59003827156700064.65240387755641.5485409541605573.6833604333332127.176438134722

88.9271232702132.020679639220247.7152352818545915.687695887129840.57567643833221.049733410866263.974106549929775.3836575281954

88.9271232702132.020679639220247.7152352818545915.687695887129840.57567643833221.049733410866263.974106549929775.3836575281954

373.721917894714.112682423537729.90040361907518.840483733944133.89840799320739.5069517835195145.201869260882146.912229795657

11.367571742890700036.47696235073773.0690707363996421.117053320867413.6471411928508

11.367571742890700036.47696235073773.0690707363996421.117053320867413.6471411928508

51.92937042205183.179757553865019.363527705486058.4152903699010741.223969821471613.760021302806126.910902100787888.0494254743366

51.92937042205183.179757553865019.363527705486058.4152903699010741.223969821471613.760021302806126.910902100787888.0494254743366

010.932924869672720.53687591358910.42519336404294.2422011891833115.84081680427763.407057215733910

010.93292486967279.4587296806877610.42519336404291.9573244658190412.90174927903413.407057215733910

290.79189702873700020.0432474995855015.058672732136913.1249972590372

000019.2295922710619015.058672732136910.2091555841917

19.633078701020800028.49864422232526.8370429400361178.708183891355619.8583544530081

17.510583706315900011.19589594448491.600158985965967.94807773911968.91599587686076

2.1224949947049500017.30274827784035.2368839540702110.76010615223610.9423585761473

17.33721159041180005.542522744794520011.586380780574

17.33721159041180005.542522744794520011.586380780574

17.33721159041180005.542522744794520011.586380780574

397.90902412614417.561628514435311.17747538886388.77717049654181127.86430276792132.6438655500272222.849036499586399.154866030335

52.9553942731326001.0219046257131115.96189081947016.4983415040665851.32468708367896.1063207636851

52.9553942731326001.0219046257131115.96189081947016.4983415040665851.32468708367896.1063207636851

52.9553942731326001.0219046257131115.96189081947016.4983415040665851.32468708367896.1063207636851

344.95362985301217.561628514435311.17747538886387.7552658708287111.90241194845126.1455240459607171.524349415908303.04854526665

11.88501156537280006.33252033059103006.05158091189645

11.88501156537280006.33252033059103006.05158091189645

012.519850771370104.4648316197264206.4435932320774500

012.519850771370104.4648316197264206.4435932320774500

05.041777743065249.33612815205186003.8922786145116400

05.041777743065249.33612815205186003.8922786145116400

226.3821656897801.841347236811943.2904342511022882.196515101812514.742879542061141.854145443466257.501888868972

226.3821656897801.841347236811943.2904342511022882.196515101812514.742879542061141.854145443466257.501888868972

38.760406641584600010.9334921332861017.980212103345726.1210816704905

38.760406641584600010.9334921332861017.980212103345726.1210816704905

99.931410405586516.09387299176388.5977531244134820.799158436456472.839541524287320.505268775000248.2207380939193103.548497670097

3.8912408236257513.21508014464324.510795790361975.2701882068312520.2654131142210.53121087655816.073738820006810.4019951896709

3.8912408236257513.21508014464324.510795790361975.2701882068312520.2654131142210.53121087655816.073738820006810.4019951896709

3.8912408236257513.21508014464324.510795790361975.2701882068312520.2654131142210.53121087655816.073738820006810.4019951896709

77.82481647251502.056171081106661.8479441981645530.322218182982.0001987324573724.658576598874172.4424664994937

77.82481647251502.056171081106661.8479441981645530.322218182982.0001987324573724.658576598874172.4424664994937

77.82481647251502.056171081106661.8479441981645530.322218182982.0001987324573724.658576598874172.4424664994937

18.21535310944582.878792847120582.0307862529448513.681026031460622.25191022708737.973859165984887.4884226750383920.7040359809325

18.21535310944582.878792847120582.0307862529448513.681026031460622.25191022708737.973859165984887.4884226750383920.7040359809325

18.21535310944582.878792847120582.0307862529448513.681026031460622.25191022708737.973859165984887.4884226750383920.7040359809325

27.503534617250100013.92159836028880.62833468035310261.969197735390510.5031365041554

27.503534617250100013.92159836028880.62833468035310261.969197735390510.5031365041554

27.503534617250100013.92159836028880.62833468035310261.969197735390510.5031365041554

27.503534617250100013.92159836028880.62833468035310261.969197735390510.5031365041554

43.38207674988161.959514458964449.5546809719378410.652015387103423.870423161220117.008276298706431.100907422003574.5509790379405

43.382076749881603.334331482875675.9933325345877222.694383671253212.974262048372231.100907422003574.5509790379405

43.382076749881603.334331482875675.9933325345877222.694383671253212.974262048372231.100907422003574.5509790379405

43.382076749881603.334331482875675.9933325345877222.694383671253212.974262048372231.100907422003574.5509790379405

01.959514458964446.220349489062174.658682852515661.17603948996694.0340142503341900

96.511435049239100029.74751136027566.8578242255681346.0974135893929117.570819930679

96.511435049239100029.74751136027566.8578242255681346.0974135893929117.570819930679

96.511435049239100029.74751136027566.8578242255681346.0974135893929117.570819930679

96.511435049239100029.74751136027566.8578242255681346.0974135893929117.570819930679

146.6380983913964.8987861474110812.44069897812439.5964365618970254.047103012543513.461330161441334.766837946279799.7167059807612

132.657592438254.8987861474110812.44069897812439.5964365618970254.047103012543513.461330161441332.798089315631195.712516215704

132.657592438254.8987861474110812.44069897812439.5964365618970254.047103012543513.461330161441332.798089315631195.712516215704

132.657592438254.8987861474110812.44069897812439.5964365618970254.047103012543513.461330161441332.798089315631195.712516215704

13.9805059531464000001.968748630648634.00418976505722

13.9805059531464000001.968748630648634.00418976505722

13.9805059531464000001.968748630648634.00418976505722

15.882615606635732.854776981772838.750916528548544.947688881510120.355175809586531.38773395548491.686056519581141.13724437204857

15.882615606635732.854776981772838.750916528548544.947688881510120.355175809586531.38773395548491.686056519581141.13724437204857

15.882615606635732.854776981772838.750916528548544.947688881510120.355175809586531.38773395548491.686056519581141.13724437204857

15.882615606635732.854776981772838.750916528548544.947688881510120.355175809586531.38773395548491.686056519581141.13724437204857

42.78627623088645.6676234177686612.51603206958551.5399534984704625.16208491342212.720072013672114.694123857802164.9067451882804

42.78627623088645.6676234177686612.51603206958551.5399534984704625.16208491342212.720072013672114.694123857802164.9067451882804

42.78627623088645.6676234177686612.51603206958551.5399534984704625.16208491342212.720072013672114.694123857802164.9067451882804

8.547704738090035.6676234177686611.13759335599441.5399534984704611.08903135191863.3336645540956301.39895332795933

128.2650960062326.163406712337482.173925372095151.7235309212623660.712938968319116.953847398979156.8813071483448148.300403056148

116.2165292358316.163406712337482.173925372095151.7235309212623659.429005947162616.953847398979156.8813071483448140.631828620775

116.2165292358316.163406712337482.173925372095151.7235309212623659.429005947162616.953847398979156.8813071483448140.631828620775

116.2165292358316.163406712337482.173925372095151.7235309212623659.429005947162616.953847398979156.8813071483448140.631828620775

12.04856677040080001.28393302115653007.66857443537336

12.04856677040080001.28393302115653007.66857443537336

12.04856677040080001.28393302115653007.66857443537336

307.6628723463421.485237073992152.134106771034790158.22527694639628.1653871765313137.268956229394329.429108378983

38.943627136712800023.098587907327510.01112353602617.478495652694435.847507365812

1.0829056095433500014.02083443882622.2265663070026410.369716813379310.5453569044503

1.0829056095433500014.02083443882622.2265663070026410.369716813379310.5453569044503

166.48122993920701.348308905643710105.7107203951199.7457848925953468.4838680779804197.748842782582

166.48122993920701.348308905643710105.7107203951199.7457848925953468.4838680779804197.748842782582

166.48122993920701.348308905643710105.7107203951199.7457848925953468.4838680779804197.748842782582

1909.96678874133117.150212789237235.38575323924881.5502045515594786.933163438383243.940228719405736.2426539543861429.71389111455

7.977486882148461.564981346421261.6559767096161024.865600332284211.305643933958223.965814765800218.2788070368444

7.977486882148461.564981346421261.6559767096161024.865600332284211.305643933958223.965814765800218.2788070368444

7.977486882148461.564981346421261.6559767096161024.865600332284211.305643933958223.965814765800218.2788070368444

008.91833239998069002.8918535890950100

008.91833239998069002.8918535890950100

008.91833239998069002.8918535890950100

014.292201618191625.1346370363752.367472994089810.9960761516445665.1631689356531200

014.292201618191625.1346370363752.367472994089810.9960761516445665.1631689356531200

014.292201618191625.1346370363752.367472994089810.9960761516445665.1631689356531200

020.886923640584626.097185499195911.72717167719993.9274878551996114.8300994692800

020.886923640584626.097185499195911.72717167719993.9274878551996114.8300994692800

020.886923640584626.097185499195911.72717167719993.9274878551996114.8300994692800

14.32358585383710006.86864781870242000

14.32358585383710006.86864781870242000

14.32358585383710006.86864781870242000

251.5463255339076.818193585285594.328781223382449.9207890779989925.19091066424678.6835162233450837.092230315399676.509975438046

242.146534999312000.84316845543629617.02797862279081.8252764098470317.501651324929764.8359015760311

242.146534999312000.84316845543629617.02797862279081.8252764098470317.501651324929764.8359015760311

06.818193585285594.328781223382449.07762062256271.636826892468565.6145929332136700

06.818193585285594.328781223382449.07762062256271.636826892468565.6145929332136700

9.399790534595190006.526105148987331.2436468802843819.590578990469911.6740738620149

9.399790534595190006.526105148987331.2436468802843819.590578990469911.6740738620149

86.857031336892115.143820040254243.323792652559710.069138752067878.154528751422620.857689657924752.022313499734497.5802205503131

1.84710798589839.8512996791259424.21704936216948.3695710032700320.476015647129313.499300242817316.64714031991511.6387604405223

1.84710798589839.8512996791259424.21704936216948.3695710032700320.476015647129313.499300242817316.64714031991511.6387604405223

20.36114384455335.2925203611282319.10674329039030.2960658261411838.076442464170321.2818363038445101.29592963326465

20.36114384455335.2925203611282319.10674329039030.2960658261411838.076442464170321.2818363038445101.29592963326465

72.04725832228860.34699735210828500.32999003538652537.9745854849374.6433184860617629.35544833199384.8055873216647

72.04725832228860.34699735210828500.32999003538652537.9745854849374.6433184860617629.35544833199384.8055873216647

72.04725832228860.34699735210828500.32999003538652537.9745854849374.6433184860617629.35544833199384.8055873216647

783.95244334034516.582651299458528.808674149566411.1899956448903356.47042607467887.9581160287113288.319356628618661.143417446183

403.4889142024979.1449425384064226.75250306845985.25798542297157179.76564735962154.1679086084556120.633682581832335.060117699682

16.28350105591085.82955551541922.056171081106661.8479441981645525.00531329717148.8431034728965216.893251925698622.3260898954291

71.40840873143002.3590776997845311.910527600515910.213780761484510.493011318669853.3536987312146

315.7970044151573.3153870229872224.69633198735311.05096352502249142.84980646193435.111024374074693.2474193374635259.380329073039

122.98235665998700093.279157546910211.923923962586174.2176704539161125.850394876556

64.512676812742700038.3017492837642044.991087127770473.9089131897668

58.469679847244500054.97740826314611.923923962586129.226583326145751.9414816867893

101.587715909757.437708761052082.056171081106664.6198604954113755.270261544441714.76498618267341.1456423678121114.932564372542

86.15370783527271.608153245632880038.80834723504299.0336475069778138.774625387151190.8207966767041

15.434008074477100014.12943598763121.730941210780422.3710169806609724.1117676958374

74.71182381361430000000

74.71182381361430000000

27.64829006260410000017.304264279911714.0778882266222

27.64829006260410000017.304264279911714.0778882266222

36.10605761280905.874774517447613.1679043397106516.70244618923994.0408055201159213.284081669427120.9006644625235

36.10605761280905.874774517447613.1679043397106516.70244618923994.0408055201159213.284081669427120.9006644625235

005.874774517447613.167904339710655.3313790211833000

18.90991760941240002.41812007440279002.8885515799765

39.921928261254801.163870423267922.092012299808927.921624489022351.132187961768331.5508538741430212.6169752974445

39.921928261254801.163870423267922.092012299808927.921624489022351.132187961768331.5508538741430212.6169752974445

39.921928261254801.163870423267922.092012299808927.921624489022351.132187961768331.5508538741430212.6169752974445

171.915529441724001.5399534984704644.231832120506613.571568387739572.517725972530198.0014564798781

119.943477345353001.5399534984704644.231832120506613.571568387739572.517725972530198.0014564798781

119.943477345353001.5399534984704644.231832120506613.571568387739572.517725972530198.0014564798781

51.97205209637130000000

51.97205209637130000000

0011.74954903489523.959880424638320000

0011.74954903489523.959880424638320000

0011.74954903489523.959880424638320000

11.163173444799200044.43806997857463.8542591303068848.462958222302343.4331575498916

11.163173444799200044.43806997857463.8542591303068848.462958222302343.4331575498916

11.163173444799200044.43806997857463.8542591303068848.462958222302343.4331575498916

56.82709058884290.58884399145648500.55998309035289439.29786817996347.9152867228960260.243857159308875.2276909086662

49.74597643839740.58884399145648500.55998309035289428.62587031260346.6673291081912429.474056204041462.4795165613349

49.74597643839740.58884399145648500.55998309035289428.62587031260346.6673291081912429.474056204041462.4795165613349

7.0811141504454600010.671997867361.2479576147047830.769800955267412.7481743473313

7.0811141504454600010.671997867361.2479576147047830.769800955267412.7481743473313

52.11483245927330007.4972517485392.1430700704900417.613268999195862.6905960091771

52.11483245927330007.4972517485392.1430700704900417.613268999195862.6905960091771

52.11483245927330007.4972517485392.1430700704900417.613268999195862.6905960091771

10.709837129245200012.839330211565309.049018935366643.06742977414934

10.709837129245200012.839330211565309.049018935366643.06742977414934

10.709837129245200012.839330211565309.049018935366643.06742977414934

127.64316743039825.7760977004713.809867902888431.451824919042891.547604263285535.733284409618673.3294112964503283.391888669266

5.583392921843800023.7781042398243033.6468886074322166.973348812829

5.583392921843800023.7781042398243033.6468886074322166.973348812829

5.583392921843800023.7781042398243033.6468886074322166.973348812829

103.8921809243686.818193585285595.0502447606128411.620228646057438.226048264743321.975819957106939.6825226890181116.418539856437

103.8921809243686.818193585285595.0502447606128411.620228646057438.226048264743321.975819957106939.6825226890181116.418539856437

103.8921809243686.818193585285595.0502447606128411.620228646057438.226048264743321.975819957106939.6825226890181116.418539856437

14.519555312036418.95790411518448.7596231422755219.831596272985412.049864345460213.757464452511700

14.519555312036418.95790411518448.7596231422755219.831596272985412.049864345460213.757464452511700

018.95790411518448.7596231422755219.831596272985410.657339974255613.757464452511700

9157.85239457153594.4357297538861240.29389417097523.2809117687574386.643910849651222.827983228494681.013369380927092.66752830107

281.515429126855000129.46477939310724.900273202822147.571761420307149.939201009028

277.424171251548000126.84891585467624.900273202822147.571761420307140.564906278684

277.424171251548000126.84891585467624.900273202822147.571761420307140.564906278684

35.021167412631800011.1958959444849013.151240852732917.8319917537215

4.091257875307460002.61586353843107009.37429473034426

264.6334116255481.3884114179826830.26394245277825.66473183534527174.1042970245837.3179035409964150.873155257798327.083597974984

66.0336339458162001.9452044191205843.944222704903715.328865767356329.1797397661256103.997790387664

58.8806177259159001.9452044191205841.200130561647615.328865767356325.9563964198675100.719850727054

58.8806177259159001.9452044191205841.200130561647615.328865767356325.9563964198675100.719850727054

7.153016219900280002.7440921432561203.223343346258053.27793966061057

198.5997776797321.3884114179826830.26394245277823.71952741622469130.16007431967621.9890377736402121.693415491672223.085807587319

135.7903607684630.84486311817669519.66772338449852.3590776997845374.784137464248412.125131547259446.8606022048199121.267861053794

135.7903607684630.84486311817669519.66772338449852.3590776997845374.784137464248412.125131547259446.8606022048199121.267861053794

24.9602618620730005.52429076208137015.141231244922726.3960404249167

24.9602618620730005.52429076208137015.141231244922726.3960404249167

26.880282005309600018.41430254027134.7373127873990523.793363384878550.5924108144237

26.880282005309600018.41430254027134.7373127873990523.793363384878550.5924108144237

6.887151900222570009.082216474139681.4160698990848721.33682144248696.90397910818865

6.887151900222570009.082216474139681.4160698990848721.33682144248696.90397910818865

76.663912328828532.607000111842234.606429881223819.493738276311251.058095721364222.93742294217757.920725900353832.0353296514621

31.514880016231300012.17236082878921.008503562583551.9687486306486314.927383903559

31.514880016231300012.17236082878921.008503562583551.9687486306486314.927383903559

31.514880016231300012.17236082878921.008503562583551.9687486306486314.927383903559

17.206611504646432.607000111842234.606429881223819.493738276311231.937927125607421.928919379593539.6295152184415.25156825207768

17.206611504646400014.30416673628194.248209697254639.6295152184415.25156825207768

17.206611504646400014.30416673628194.248209697254639.6295152184415.25156825207768

032.607000111842234.606429881223819.493738276311217.633760389325517.680709682338900

010.01954854212683.855320777074994.331119214448163.280047639985824.6879657791969800

022.587451569715430.751109104148815.16261906186314.353712749339712.992743903141900

33.5768705479663010.9177225545486028.02493553213280.71648909819368626.897814242017223.6792296598439

33.5768705479663010.9177225545486028.02493553213280.71648909819368626.897814242017223.6792296598439

33.5768705479663010.9177225545486028.02493553213280.71648909819368626.897814242017223.6792296598439

0010.917722554548600000

12.19642646211060007.51963160450480.7164890981936868.832922960790738.98253315952391

6.4141332257567200012.3031823565768018.06489128122657.34834825015998

3293.85374614765217.218980278468433.234654497985200.5436385142911745.04332419473440.8949883440641866.582313390992779.19721696218

660.25152983139930.233994977423179.250806824322933.8920353375835277.01806435649772.2414147889814408.291462621479573.202706052614

231.0239147768464.7588208289136413.42805603988027.7047486757415382.001604251315814.797593725363877.9466315156796132.16033865217

42.80364905988320000000

37.92310972177650006.32536494038697001.88898217730101

1.241885369242260004.7642110402063602.7981363516452915.6504182944896

66.1319253276298002.1847616135935634.47012298178862.3647669743338432.392218849095657.64652506591

10.94411481644740000000

20.466673682157414.278540937487236.89900304879036.4352117483252717.029806599472616.712078826971619.36872617450127.181019987721

11.1027251761061001.205180998802973.042363028392646.522387171056661.786853376730011.81711872490369

9.3639485060512814.278540937487236.89900304879035.230030749522313.987443571079910.189691655914917.58187279777125.36390126281731

48.6405102953220000000

48.6405102953220000000

331.9424802853018.6620438564921112.831974057426412.5209889206989153.4169493246938.1227873682234308.708649611861394.661710367905

106.87655279685900020.28242018928435.2179097368453442.8844810415202111.44994846076

225.0659274884428.6620438564921112.831974057426412.5209889206989124.65278978352332.904877631378265.824168570341281.185399207858

173.23583030830812.576712678475435.12604054573461.80287238845322104.55040783085310.0437329514419254.465784663141149.654813695918

173.23583030830812.576712678475435.12604054573461.80287238845322104.55040783085310.0437329514419254.465784663141149.654813695918

1.370155219586530003.94221688186088015.43572870039071.56971758395436

011.374742469110616.04816453546661.802872388453220000

01.2019702093647819.07787601026802.88554019187755000

59.38666954661400017.9004150275195086.018290461188840.8217834478364

125.8405862686316.599496809908539.310963386143372.092012299808920000

125.8405862686316.599496809908539.310963386143372.092012299808920000

125.8405862686310000000

84.04796270883981.7532497790734422.26230343453831.6673180735319241.872778156400816.775863337864444.425607535697282.7187515709914

84.04796270883981.7532497790734422.26230343453831.6673180735319241.872778156400816.775863337864444.425607535697282.7187515709914

57.411749856773400028.678012152881411.80445153581428.296727244609772.6251713330358

20.02844541572080000000

11.67372247087720004.4783583777939707.890744511639715.34959752611646

11.67372247087720004.4783583777939707.890744511639715.34959752611646

11.67372247087720004.4783583777939707.890744511639715.34959752611646

255.13068494010727.88031706351849.941633746068522.2639812005952182.78211713423146.4862377040382175.728043464304210.216960327802

48.64051029532212.361409161501818.25199534049986.5432935204831552.478128477982412.120787707090333.354596365626841.1880244311503

48.64051029532212.361409161501818.25199534049986.5432935204831552.478128477982412.120787707090333.354596365626841.1880244311503

5.40450114392467000.6844237770979811.727761719827921.481628690709174.0590249545471720.6388793445851

5.40450114392467000.6844237770979811.727761719827921.481628690709174.0590249545471720.6388793445851

66.43143826326360003.251333418221632.9093799744834519.760071483272815.8731744777445

12.97080274541920001.5549855478451301.826561229546233.71499828202533

7.074983315683170001.69634787037652.9093799744834517.933510253726612.1581761957192

121.2361634319918.609805068926824.37880789496726.8231785778383366.485670259507919.18409721183285.8022182689937114.582407609372

008.4355736660786102.39228545822327000

4.771276214255020000.76266321147717603.5834443740416515.4875950177077

9.872069742813768.609805068926815.94323422888866.823178577838334.628389574057695.169744416197526.950972966560717.6717677263361

57.302518978050400033.229828145731613.151991665473227.023097642601856.4883300417549

9.216096687534670007.3657210161085024.225969991876312.3181521982945

1217.05880343188110.607144156452172.80814894753299.6922009540503740.602326824538188.932932524617680.7768383331751057.41846101083

50.251123219008101.634043243263571.4685649256936838.926128283805211.126933346120521.7735775707498117.354581491793

50.251123219008101.634043243263571.4685649256936838.926128283805211.126933346120521.7735775707498117.354581491793

69.08937788886550.95176416578272501.8102310512632331.98827412709999.7968917508116333.54908380799250.4936501189563

69.08937788886550.95176416578272501.8102310512632331.98827412709999.7968917508116333.54908380799250.4936501189563

39.9245807048306001.281811004507219.41484845864449.2494739073173310.769320544145439.9416000263994

39.9245807048306001.281811004507219.41484845864449.2494739073173310.769320544145439.9416000263994

9.1679052491511800043.4212950245462.017007125167138.231912314069729.0676767530588

9.1679052491511800043.4212950245462.017007125167138.231912314069729.0676767530588

24.32025514766090000010.95936737727745.57249742303798

24.32025514766090000010.95936737727745.57249742303798

3.9796781150717801.12154786242181021.62843534730041.091017490431313.45013269029518.2372642935788

3.9796781150717801.12154786242181021.62843534730041.091017490431313.45013269029518.2372642935788

1020.32588310729109.655379990669170.05255784184695.1315939725862585.223345583142155.651608904769552.043444028646796.751190904003

116.47133784588554.021902152959649.179571122689638.0827150950129112.92186275892439.1204168911659123.401213606556119.741577798299

228.22861960261912.648803295621222.748324722034414.6756922453704185.72560677890241.6016678780072152.611733566567213.243183211402

63.735841076628600019.30326886980162.7588948033894834.011829791550449.9603217237887

18.950848167008501.6022112320311603.6350311508067802.134941696872226.51330867627815

122.8483745361021.1659111030838418.50553972995991.10876651889873001.643905106591610

31.16770562612860000003.24611500371144

11.53529888426600013.27576594207709.096708099716419.8231133625857

00001.5048247237210901.7676398995608712.5830586971825

9.5076997467886800022.30432395190361.8751863116787922.141346904405724.2664848718857

38.54531004534930002.640541496340790012.6169752974445

93.70871712095827.805262614787212.38864584432474.6391904556432259.88604812917117.718426867818292.470983142344970.3943193089335

2.933096098210365.757585694241179.138538138251829.5819328793717313.62240270557795.926514762836671.6521659362729712.1447877952307

31.55060127264130000000

20.12710770840900021.7161774785268014.171595746479417.293957519773

289.57939328984314.532169761701127.862999572820816.297597771320770.790381433302339.018338526492569.516634279026588.5785454415569

013.447601293716115.607946684098715.26618705606611.980012609358523.228564888803200

013.447601293716115.607946684098715.26618705606611.980012609358523.228564888803200

23.43672771399831.084568467984973.44289111255071.031410715254634.209226742434984.9244965724887807.67150696861824

94.879020082232708.8121617761714050.681010448285810.865277065200569.516634279026572.4780507742257

94.879020082232700050.681010448285810.865277065200558.17935768184370.1721897715893

00000011.33727659718352.3058610026364

130.884368314289010.2808554055333060.432911264736414.028182898211473.4125677888405141.605077331906

13.289756911290100011.21628919757140.87440381746770421.559411233988332.8868700376013

13.289756911290100011.21628919757140.87440381746770421.559411233988332.8868700376013

13.35486419952310008.664387843982280.3230469016081923.89525592353887.64999646231325

13.35486419952310008.664387843982280.3230469016081923.89525592353887.64999646231325

13.35486419952310008.664387843982280.3230469016081923.89525592353887.64999646231325

18.43219337506930000010.3825585679473.51947205665557

18.43219337506930000010.3825585679473.51947205665557

18.43219337506930000010.3825585679473.51947205665557

2.02668792897174010.280855405533307.774927739225651.666832277047812.283201536932799.28749570506329

2.02668792897174010.280855405533307.774927739225651.666832277047812.283201536932799.28749570506329

83.780865899434400032.777306483957111.163899902087715.292140526433688.2612430702726

83.780865899434400032.777306483957111.163899902087715.292140526433688.2612430702726

13.81505617855300013.2495809993904007.91360580786458

54.296383585475700019.527725484566711.163899902087715.292140526433680.347637262408

15.66942613540570000000

26.03804268597160000066.00065111966329.8304694764858

26.03804268597160000066.00065111966329.8304694764858

26.03804268597160000066.00065111966329.8304694764858

26.03804268597160000066.00065111966329.8304694764858

8.305866819603393.1850840167484817.12451707317362.1011359947251813.6028899991144.000466917465448.534917532652139.5281088119259

8.305866819603393.1850840167484817.12451707317362.1011359947251813.6028899991144.000466917465448.534917532652139.5281088119259

8.305866819603391.233768363051687.833032689930141.1732979035965413.6028899991141.9918991526546548.534917532652139.5281088119259

5.883932697014750004.51447417116327019.885948870059824.2673274874235

01.233768363051687.833032689930141.173297903596544.44281585098607000

289.09143087369332.963256715302374.808001704257519.4461174489171142.04010938548544.2452868700968181.838854134831189.225789480304

33.49018741645120000000

33.49018741645120000000

33.49018741645120000000

181.8306796857725.269654703203837.5577404365191.83267193206402124.76306420185521.0318486639165138.323718960347140.773483074017

18.74221497617910005.13573208462611000

18.74221497617910005.13573208462611000

121.93771142701200075.52641545564439.95803496766624121.01069302763679.686158883195

45.710359072712100047.21160940445465.7837071781899927.728519870219824.1698683408876

5.075531509077080006.084726056785284.17432778947625014.5369497992295

71.151820845222800022.2300799944044093.28217315741640.9793407430779

9.07956192179341030.58766897514051.8326719320640220.27392727504523.9673363288410713.151240852732920.8039903793418

9.07956192179341030.58766897514051.8326719320640220.27392727504523.9673363288410713.151240852732920.8039903793418

17.97703725624839.9613912125758222.8303601794588.97370640855137.971133578739198.19082215893357028.7846684426233

15.89244395787740005.542522744794523.5647106123002603.31039450873542

15.89244395787740005.542522744794523.5647106123002603.31039450873542

875.7891712158643.778407183999389.163097615501161.7525279073178408.030167712309136.567163112979347.896383330046769.982359092362

240.05867657021910.420894349644724.966921926201417.3371042307645108.67945318142333.577225009278769.3073627580536145.035300968194

167.7435816178820.96756108139737614.963927477574413.341549207706104.89705590288126.008905481061669.3073627580536145.035300968194

46.42957800917090.96756108139737610.2381962544730.7199782590251519.33655574827524.675789244705568.5397667874888642.336506395808

28.509878933602504.278737509817348.972677031550419.35649967855455.1280171515568319.224940672324810.8874424441708

72.31509495233699.4533332682473510.0029944486273.995555023058493.782397278542217.5683195282170800

09.4533332682473510.0029944486273.995555023058493.782397278542217.5683195282170800

590.7216503576833.357512834354664.196175689299844.4154236765533293.826423768805102.9899381037274.262954502014612.260589082735

590.7216503576833.357512834354664.196175689299844.4154236765533293.826423768805102.9899381037274.262954502014612.260589082735

03.3311745802395312.3370264866407.997068531774941.7144560563920400

64.76945829850760004.78457091644654050.557881229812819.9554014627696

13.28887003552330007.25674831820492.347421495304520.11240151503822.2419025577665

45.010621467312800027.8504874240924.776593987957919.8143588453230236.595505464727

3.4743221639515700013.32844755295822.857426760653399.785149443997667.96071060433997

44.89893258029720000000

33.60617074949520008.481739351882515.8187599489669111.955673502484444.5799793843039

02.454549690702821.298634367014731.167122651472354.419432609665091.2632834099730800

47.26203429504951.5702506438839619.10785247089024.0355056713690324.72791396291078.4186174925167822.628653289115246.0238653562651

010.696432138383912.450210215875211.18938688796888.9875311480957120.91950967891200

273.3581990043093.939921919150711.95272272794256.40712003007043142.8309544019128.3334171843667126.120180999529362.026970528521

05.87854337689334.1468996593747811.180838846037608.0680285006684100

1619.16907533702138.768926772262218.758325201082111.344886799947857.934667372576244.089407648454951.6697245627291331.69413335353

59.08042470017161.7062113703665901.622585149607932.08579935309718.1959362695814320.047623251117162.7925319376475

59.08042470017161.7062113703665901.622585149607932.08579935309718.1959362695814320.047623251117162.7925319376475

59.08042470017161.7062113703665901.622585149607932.08579935309718.1959362695814320.047623251117162.7925319376475

21.32514983390960002.71164967456736025.31991773370986.47837138835943

21.32514983390960002.71164967456736025.31991773370986.47837138835943

21.32514983390960002.71164967456736025.31991773370986.47837138835943

494.92861741962576.772889482838379.260551904355570.1524415425646285.32536413824101.534548537796335.147197190039513.685598359168

021.99832269969519.310963386143378.368049199235692.6405414963407913.586255541219900

021.99832269969519.310963386143378.368049199235692.6405414963407913.586255541219900

19.880770242148623.626392397293725.364593973827822.468396418212124.862480894030830.240488806825348.225375612725414.1480105351514

023.626392397293723.913179093046622.46839641821219.2477114078895324.319596923270300

19.880770242148601.45141488078117015.61476948614125.9208918835550348.225375612725414.1480105351514

009.62889872127997001.1708480385116300

009.62889872127997001.1708480385116300

02.252968315137857.151899412544915.356359994679842.704322691904571.1595354970767400

02.252968315137857.151899412544915.356359994679842.704322691904571.1595354970767400

39.697508182330300022.30143616234530.82766844101684539.72871182034353.6235335293956

39.697508182330300022.30143616234530.82766844101684539.72871182034353.6235335293956

222.06522723701826.674423017218627.804196410559433.959635930437120.43156764611240.366138089048374.889569856134250.331419378655

63.9072398040726002.4279558808001431.66722393056869.6359938935902826.398476164244868.3342749686408

24.60503565290299.245110068506217.608738802333012.930660402375495.01007940443042.676864298345910.769978972642445.48114500626685

09.0681974684298611.03030093976849.268505168364241.307931769215536.4554700524169500

08.361115480282527.8642399914836518.55306525647273.345387234407843.8250812413527500

12.75042502887080008.152351415887082.3303286203386915.960243753316634.0842075389701

70.107551151916500034.4007975947866.7045767568403520.204420304477861.6399156291352

000022.29271316379742.83213979816973044.3827228383556

27.183975876823101.300916676974340.77944922242441411.3139546011935.9056834279936511.556450661452429.9680880746857

155.82481892372900045.4465897593169.61173130705145141.15478705636281.2085561547888

9.41429231522360009.02894834232656015.908759096047821.5709577665986

46.161610115838700017.31463569344432.1100997617132765.756204263664318.7594482030262

31.38097438407880006.01929889488437000

6.948644327903120002.665689510591645.714853521306781.5656239110396314.3292790878119

32.22806817113360000000

3.233718135977070006.202712434617690.66488600524898817.304264279911718.5235371402924

3.233718135977070006.202712434617690.66488600524898817.304264279911718.5235371402924

155.0475082736555.3837164933164232.7563185215266.4751593102003755.08304857355999.8013966979766239.82005469704289.8674263811589

155.0475082736555.3837164933164232.7563185215266.4751593102003755.08304857355999.8013966979766239.82005469704289.8674263811589

2.288965190368090008.781094858419540.94126999174464711.6040360465292.62235172848845

03.0283405274904812.81768985624935.759826072201190000

38.5985984924168000.71533323799917914.44631734772252.322811431240826.3635036384191437.7491760915477

365.26468441758518.260701257657218.19611498514058.40098127990323115.63862144309130.3259616725953389.034037989879171.702993334208

175.91644849991213.45282042019811.019402313395761.542631678467865.89683534041915.6707531017359280.151805108382111.412578463307

6.981891429950500.59028834864306108.704943019037321.7226591954178318.877379214449110.3984401673914

41.04043056167790008.746793706628867.500745246715155.1372034580987831.3452980045886

35.021167412631800.4291139647526951.54263167846785.354558929971061.669731115790509.30364787150689

41.69186596741870002.221407925493040010.6142808057866

14.93684961824840002.2039165245049109.060894288300216.58168986972991

4.6694889883509100015.67425432227891.9201907831590828.932729876012324.0731888675241

31.574754521634200022.99096091250492.85742676065339218.14359827152119.0960328767793

04.8078808374591216.53415920889896.8583496014354304.9489453174203100

04.8078808374591216.53415920889896.8583496014354304.9489453174203100

34.200358801398200.642553462845832016.03578846215294.375434727250527.7058051871481529.6038925598893

34.200358801398200.642553462845832016.03578846215294.375434727250527.7058051871481529.6038925598893

14.72602605271210001.283933021156531.1010268252058904.60114466122402

14.72602605271210001.283933021156531.1010268252058904.60114466122402

131.3876808002066.6208586421470833.345893598949113.149581873255139.709648040991221.740458003030534.092636560675183.0867565648545

66.25532366655632.331822206167681.644936864885331.478355358531648.4302430772460805.871089666398615.642796337528

66.25532366655632.331822206167681.644936864885331.478355358531648.4302430772460805.871089666398615.642796337528

65.13235713364984.289036435979431.700956734063811.671226514723531.279404963745221.740458003030528.221546894276567.4439602273265

03.068187113378529.739757752610511.67122651472355.524290762081377.8955213123317300

1294.8686289337352.8387755831798172.78536913350136.5181524473787405.975427778179126.302482772542284.610263731561778.150011919386

1294.8686289337352.8387755831798172.78536913350136.5181524473787405.975427778179126.302482772542284.610263731561778.150011919386

1294.8686289337352.8387755831798172.78536913350136.5181524473787405.975427778179126.302482772542284.610263731561778.150011919386

1294.8686289337352.8387755831798172.78536913350136.5181524473787405.975427778179126.302482772542284.610263731561778.150011919386

105.3738721417682.5484395695821602.4235333746420355.456728012313720.950708420564843.596858835789114.79914447869

180.2186924972197.2869443942742.570213851383335.9217023721882657.901188638773219.619533004856443.1215946694981105.725264596474

52.8774885440433022.84634534562960.7726595950513830.76258671888748.3632002750830935.512932616264676.8888146175274

56.902415154096812.341120311193825.493858468718916.238196069516243.065873016188818.063929083984530.675741223479882.7875813326933

38.82357719910760002.463247389689720.44697178378935706.33687283168826

14.83947771721690004.744023705290220028.334732659515

93.218614706919900000015.1403703569334

1952.09917974376346.958031514111498.808155270923320.8884274838591317.87854875706577.0572881828141551.091804802581442.05177240363

1952.09917974376346.958031514111498.808155270923320.8884274838591317.87854875706577.0572881828141551.091804802581442.05177240363

1952.09917974376343.005790486708477.897940886787319.6355839596791311.55318381667564.8526857474811551.091804802581442.05177240363

1952.09917974376323.214558594969434.172466511097289.8481586741111300.39329678866544.3564588770081551.091804802581442.05177240363

1952.09917974376323.214558594969434.172466511097289.8481586741111300.39329678866544.3564588770081551.091804802581442.05177240363

016.460057311499820.813853757644628.20347311571335.1620856291791320.496226870472400

014.349810971076619.697380772428323.18643004377335.1620856291791311.807580792829500

02.110246340423241.116472985216295.0170430719399608.688646077642900

03.9522410274028320.91021438413551.252843524179356.3253649403869712.204602435333100

03.9522410274028320.91021438413551.252843524179356.3253649403869712.204602435333100

03.9522410274028320.91021438413551.252843524179356.3253649403869712.204602435333100

2273.80437827872226.345550844707380.463727803337181.6773748113311561.97733402922344.3454183505141529.190968117952018.48345547187

0011.033113118133300000

0011.033113118133300000

0011.033113118133300000

0011.033113118133300000

152.8319568006941.207485708332830.979582846555727.5598859035439130.54328311009343.326406939538798.5694751332928175.912721925712

100.8912068909441.207485708332830.382148149866127.022952964609995.194747782396534.958610722784262.218428726791197.4221165952931

100.8912068909441.207485708332830.382148149866127.022952964609995.194747782396534.958610722784262.218428726791197.4221165952931

3.0086913584735100015.87047105532663.7117089880652410.1684851954121.72345281124886

015.483140018600722.596118461494311.735678755025711.132596552516917.152450375500600

06.568513256810354.633624971507971.0410953229096102.253745050656200

08.86365166087130.7214635372304063.890408838241162.455240338702842.8072964666068400

1.9989250806296700013.419738289622304.503849607100286.87020230237559

32.42700686354797.994818992574903.8014852076527921.03673305441915.4862593804545222.832015369327955.7249742303798

51.940749909749700.5974346966895860.53693293893400935.34853532769688.3677962167545836.351046406501778.4906053304194

20.32333299247430002.338975475867320.6685901055567827.3265965753386444.704157599859

20.32333299247430002.338975475867320.6685901055567827.3265965753386444.704157599859

2.6451032788996800016.06661804722160.72514757672170817.87933046444047.07084265158897

2.6451032788996800016.06661804722160.72514757672170817.87933046444047.07084265158897

28.972313638375700.5974346966895860.53693293893400916.94294180460796.9740585344760911.145119366722726.7156050789714

28.972313638375700.5974346966895860.53693293893400916.94294180460796.9740585344760911.145119366722726.7156050789714

857.46193817951725.130279906943279.130690183521730.4484690542742749.19441959508115.243017658212913.9759193770711068.85965384918

857.46193817951725.130279906943279.130690183521730.4484690542742749.19441959508115.243017658212913.9759193770711068.85965384918

586.55169916617424.3829009947146.873252691893523.3224988956979548.84110781219780.1768178858528688.354531084837709.395483628094

586.55169916617424.3829009947146.873252691893523.3224988956979548.84110781219780.1768178858528688.354531084837709.395483628094

241.5754982682270.7473789122332327.51242730445896.27307283634652180.57626323701434.1430311266093210.368660499116334.474104457979

9.0493972642459400034.71595641700765.5819499510438343.327731491561720.7348741322343

161.5186835031310.7473789122332327.51242730445893.7531489297585103.62659740159117.6509062712525120.671584466668209.064317346216

17.94504263322560007.24653459189962020.21630875420124.8868816951211

004.74501018716920.8528973222297911.84181301820520.92316864574955900

004.74501018716920.8528973222297911.84181301820520.92316864574955900

1112.05908364805150.612820348815240.366657520191103.719037077678545.36045428476167.617039026713387.759262287429629.263968321166

0.93840212788402400017.99983270817513.8589043069917114.800431827834410.75079888689

0.93840212788402400017.99983270817513.8589043069917114.800431827834410.75079888689

0.93840212788402400017.99983270817513.8589043069917114.800431827834410.75079888689

0017.016588257434400000

0017.016588257434400000

0017.016588257434400000

41.866964431914917.299164673274927.255550026904612.905073497509836.9500892814248.2319484318701527.107719043265719.3633199580365

41.866964431914917.299164673274927.255550026904612.905073497509836.9500892814248.2319484318701527.107719043265719.3633199580365

41.866964431914917.299164673274927.255550026904612.905073497509836.9500892814248.2319484318701527.107719043265719.3633199580365

3.725656107726790003.573158280154760014.2276529949906

3.725656107726790003.573158280154760014.2276529949906

3.725656107726790003.573158280154760014.2276529949906

2.4252886019828100014.7314420322171.9946580157469711.839759770465911.1141222841755

2.4252886019828100014.7314420322171.9946580157469711.839759770465911.1141222841755

2.4252886019828100014.7314420322171.9946580157469711.839759770465911.1141222841755

50.155168531676111.92423497877077.328926625726712.07567495830337.17222881176066.774600492357712.657430258004620.9911818702205

1.4447676325343111.92423497877077.328926625726712.07567495830327.08831857964135.941184353833793.255257636815063.31039450873542

1.4447676325343111.92423497877077.328926625726712.07567495830327.08831857964135.941184353833793.255257636815063.31039450873542

17.88880413663330002.45094044318847002.92775696481854

17.88880413663330002.45094044318847002.92775696481854

30.82159676250850007.632969788930810.8334161385239069.4021726211895114.7530303966665

30.82159676250850007.632969788930810.8334161385239069.4021726211895114.7530303966665

17.32315841878399.026408540003923.18374877074579015.8449419719914015.50853874143023.15424382436111

8.259709295432029.026408540003923.18374877074579012.3679556538284015.50853874143023.15424382436111

8.259709295432029.026408540003923.18374877074579012.3679556538284015.50853874143023.15424382436111

9.063449123351910003.47698631816302000

9.063449123351910003.47698631816302000

116.2158421239086.5962216253874714.286052176423411.533585481348450.45200367620718.9850765115340826.511805524683350.691691595242

116.2158421239086.5962216253874714.286052176423411.533585481348450.45200367620718.9850765115340826.511805524683350.691691595242

33.8368767271804002.1425439978719422.37047631600951.2869911415275321.870191106071736.0673979379751

63.67484984114880000000

13.897288655806200001.428713380326705.97053295325497

4.8068268997733.58741877871956.326680249558967.9603750074780526.27573769173234.72083103551934.641614418611618.65376070401193

93.530088931331300015.306888986600507.7058051871481513.0605408352453

93.530088931331300015.306888986600507.7058051871481513.0605408352453

93.530088931331300015.306888986600507.7058051871481513.0605408352453

105.86793258212800032.92910571907325.1341999549707945.712869274205164.3668151538077

105.86793258212800032.92910571907325.1341999549707945.712869274205164.3668151538077

87.396852723145300032.92910571907325.1341999549707945.712869274205164.3668151538077

60.86566528933339.327288824670714.386498306360872.9567107170632719.26285285985426.3466221384301520.423978437871214.1307738829739

7.80329042170949.327288824670714.386498306360872.9567107170632714.86655864081566.3466221384301515.259878626588710.6297283815887

7.80329042170949.327288824670714.386498306360872.9567107170632714.86655864081566.3466221384301515.259878626588710.6297283815887

53.06237486762390004.3962942190385905.164099811282533.50104550138513

00004.3962942190385905.164099811282533.50104550138513

53.06237486762390000000

64.0006714412131.0227290377928401.9452044191205838.0562252498945.2636808748878227.398418443193458.657867610926

64.0006714412131.0227290377928401.9452044191205838.0562252498945.2636808748878227.398418443193458.657867610926

295.31025263087120.292299619764584.504490576103414.7363726255556173.03007759099348.0723256175969131.160402472399236.317211935851

4.214340242193951.1515171388482417.05860452473670.54753902167838614.19883313724298.4361905089765320.177896615203813.2774306830508

01.1515171388482417.05860452473670.5475390216783862.07331406379352.3706059051346700

02.2421367366996916.60753565509231.066121652787244.036981710751793.4618824215608500

02.2421367366996916.60753565509231.066121652787244.036981710751793.4618824215608500

41.34443375102370.32386419530106601.2319627987763727.98973986121235.0004968311434227.855058750579954.3318498746202

41.34443375102370.32386419530106601.2319627987763727.98973986121235.0004968311434227.855058750579954.3318498746202

6.242632337367528.3427425483538828.40739314783818.5308506101962837.076397759488814.069323968615512.30731095843985.36390126281731

6.242632337367525.0814527495179513.74099802386056.9801282061421128.268437663303114.069323968615512.30731095843985.36390126281731

03.2612897988359314.66639512397761.550722404054178.8079600961857000

208.033640009928.2320390005616422.43095724843633.3598985421173675.982460935535317.104431887300570.8201361481752145.106765821784

16.676746386967500013.32844755295822.2859414085227115.656239110396331.84284241736

191.3568936229538.2320390005616422.43095724843633.3598985421173662.654013382577114.818490478777855.1638970377789113.263923404424

8.386294878503782.0635594744846721.835445109097302.476968129310821.379447401694745.668638298591743.84310167106067

8.386294878503782.0635594744846721.835445109097302.476968129310821.379447401694745.668638298591743.84310167106067

8.3862948785037800001.379447401694745.668638298591743.84310167106067

67.103886979625201.634043243263572.9371298513873542.391128025451513.421411105557543.991579118347585.5944188006387

67.103886979625201.634043243263572.9371298513873542.391128025451513.421411105557543.991579118347585.5944188006387

67.103886979625201.634043243263572.9371298513873542.391128025451513.421411105557543.991579118347585.5944188006387

44.654420689476601.634043243263572.9371298513873542.391128025451513.421411105557543.991579118347585.5944188006387

44.654420689476601.634043243263572.9371298513873542.391128025451513.421411105557543.991579118347585.5944188006387

22.44946629014860000000

22.44946629014860000000

389.73918102782018.6644475652680123.32395555512210.082810629966200.245673143795275.974570503964

389.73918102782018.6644475652680123.32395555512210.082810629966200.245673143795275.974570503964

389.73918102782018.6644475652680123.32395555512210.082810629966200.245673143795275.974570503964

389.73918102782018.6644475652680123.32395555512210.082810629966200.245673143795275.974570503964

389.73918102782018.6644475652680123.32395555512210.082810629966200.245673143795275.974570503964

765.44230945564826.9981411370622142.66999084271721.1263734809084245.72171402803283.9382254718543132.76994583684455.209173036993

765.44230945564826.9981411370622142.66999084271721.1263734809084245.72171402803283.9382254718543132.76994583684455.209173036993

765.44230945564826.9981411370622142.66999084271721.1263734809084245.72171402803283.9382254718543132.76994583684455.209173036993

765.44230945564826.9981411370622142.66999084271721.1263734809084245.72171402803283.9382254718543132.76994583684455.209173036993

156.809783831765.4943380373965531.66779395739721.9236060355633892.277326380428725.189209062771863.9087918185853221.853262189068

162.4923621797120.115813691232578.858256566754114.144461023587779.574926219460629.130060385950827.9669723858429112.938131391267

63.50288844111480001.29582128987094000

41.277907829255100020.43400383948833.4723160635787913.674436691358813.178070380115

2.1618004575698648.163362082738551.651712643127443.010040368709328.78235317309144.08687835492517.44830354190262.47666552135021

2.1618004575698648.163362082738551.651712643127443.010040368709328.78235317309144.08687835492517.44830354190262.47666552135021

2.1618004575698648.163362082738551.651712643127443.010040368709328.78235317309144.08687835492517.44830354190262.47666552135021

014.509115949487812.062870342492410.8412726292328.707919067932716.93402227251890.7306244918184910

014.509115949487812.062870342492410.8412726292328.707919067932716.93402227251890.7306244918184910

2.1618004575698633.654246133250739.58884230063532.168767739477320.074434105158337.152856082406102.47666552135021

2.1618004575698633.654246133250739.58884230063532.168767739477320.074434105158337.152856082406102.47666552135021

8.3862948785037823.684963365975639.632091283724917.217980401005819.897796588527932.37463631832719.785149443997667.89208378878531

8.3862948785037823.684963365975639.632091283724917.217980401005819.897796588527932.37463631832719.785149443997667.89208378878531

8.3862948785037811.984063406711327.416666312634310.499312314937116.43356467246177.439363141523099.785149443997667.89208378878531

8.3862948785037811.984063406711327.416666312634310.499312314937116.43356467246177.439363141523099.785149443997667.89208378878531

8.3862948785037811.984063406711327.416666312634310.499312314937116.43356467246177.439363141523099.785149443997667.89208378878531

011.700899959264312.21542497109066.718668086068683.4642319160662724.93527317680400

011.700899959264312.21542497109066.718668086068683.4642319160662724.93527317680400

011.700899959264312.21542497109066.718668086068683.4642319160662724.93527317680400

36.42168540415990002.209568160805680.53102621215682405.59827526711077

36.42168540415990002.209568160805680.53102621215682405.59827526711077

36.42168540415990002.209568160805680.53102621215682405.59827526711077

36.42168540415990002.209568160805680.53102621215682405.59827526711077

36.42168540415990002.209568160805680.53102621215682405.59827526711077

369.86709949851826.748699722585273.455863825736918.31244099941170.93095789774237.1214892588142106.876228795431234.471162420575

6.9902529765731900010.056193962112201.9687486306486310.0104744126431

6.9902529765731900010.056193962112201.9687486306486310.0104744126431

6.9902529765731900010.056193962112201.9687486306486310.0104744126431

6.9902529765731900010.056193962112201.9687486306486310.0104744126431

183.10152047043526.748699722585273.455863825736918.31244099941160.8747639356337.1214892588142104.907480164782224.460688007932

183.10152047043526.748699722585273.455863825736918.31244099941160.8747639356337.1214892588142104.907480164782224.460688007932

183.10152047043526.748699722585273.455863825736918.31244099941160.8747639356337.1214892588142104.907480164782224.460688007932

183.10152047043526.748699722585273.455863825736918.31244099941160.8747639356337.1214892588142104.907480164782224.460688007932

179.775326051510000000

179.775326051510000000

179.775326051510000000

179.775326051510000000

108.3449370390391.7665319743694516.82321793632720.839974635529349.615925428006363.63672496810431022.0841284861224

108.3449370390391.7665319743694516.82321793632720.839974635529349.615925428006363.63672496810431022.0841284861224

42.12844403196250003.25462091409446004.61809178704806

42.12844403196250003.25462091409446004.61809178704806

42.12844403196250003.25462091409446004.61809178704806

66.21649300707681.7665319743694516.82321793632720.839974635529346.36130451391193.63672496810431017.4660366990743

66.21649300707680000000

66.21649300707680000000

000000017.4660366990743

000000017.4660366990743

01.7665319743694516.82321793632720.839974635529346.36130451391193.6367249681043100

01.7665319743694516.82321793632720.839974635529346.36130451391193.6367249681043100

4590.26329957519323.081927568558693.612249127512307.0019207808962316.06033827403643.316652848192522.513518999973643.83129478694

3415.87531998829180.966584259203505.49824875554188.3852471744991758.98737698334459.6570605610292096.656231333592836.05600933095

613.37799738652619.046506358189138.908946942227225.9097720855689300.39739938793357.2701044384797527.081073587844546.561184122111

613.37799738652613.605587877131225.749452023144619.2571729721766292.64951487562746.138563666543512.786246574004543.653794162266

343.72422617913103.123297844718972.80700384531325147.11224875834321.3599759288865285.028154981027295.840805278731

04.3450103220515710.72784911881744.132049138753043.4769863181630210.435819473690600

59.38666954661400013.0184836563778047.787939145104929.1584167484546

82.087016418527908.995748479841631.5914873753877735.05801500051747.087318343352935.282012861381365.1587297103258

05.4409184810579113.15949491908266.652599113392387.747884512306611.131540771936714.29482701384012.9073899598459

05.4409184810579113.15949491908266.652599113392387.747884512306611.131540771936714.29482701384012.9073899598459

2543.66105846777148.184772383884444.868088391847156.600500181771341.47726151938393.7108873604411330.772747207572025.9860815547

12.82826645151354.0553429672481403.856579196169513.841080151148913.073444175832214.45191302498125.51126118761997

12.82826645151354.0553429672481403.856579196169513.841080151148913.073444175832214.45191302498125.51126118761997

30.70686096899220004.0546019697910402.6949264042485410.324546725641

30.70686096899220004.0546019697910402.6949264042485410.324546725641

1145.998846642856.3849786154508160.56131477545844.3158111433505638.066598128465144.577201676993744.772832998541921.934450026356

03.2163064912657715.31492943169107.72130754792063000

23.2147890045854015.943234228888609.80619557375342063.514515481948322.7965803669736

018.957904115184419.391532147022212.01914925635489.8608839619447221.46554737271332.67301643348230

2.7403104391730700010.5125783516293.380617575984315.43572870039073.13943516790872

17.78255215097790.835778568518881001.0032164824807304.713706398828994.79354617035524

48.17130624090472.9895156489329119.94012063090571.4214955370496521.95315689646999.1478708865455523.799132906735950.1881176908685

56.226644928537100028.24652646544372.2020536504117933.179736096344367.4834550312856

25.82681962583480002.47696812931082002.95884818922372

6.709035902803020006.43442295660052011.33727659718350

35.483538783576900.656224813119147082.72402806176496.3720064068806663.228342445984538.5263678000716

1.5004784666937300015.82967292922032.4681115464769721.975081116391612.892667559728

12.34720645958170005.3826422810023701.2645423896858512.8596094377799

188.11570849154600001.445926794547500

14.59215308859660004.43739778287513014.835241205826710.6013365609015

02.6369556451007614.35001465328070.3582444326005582.260883672149620.77552131791562300

4.6620297407656800029.81030509326491.5336987085935121.593719737829923.8594163253554

27.57572237215100011.01958262252453.7799031164548915.53296163708652.6535189578391

6.238925311038910009.30775197284971.1402558094768913.27619325988475.55926108711626

27.25382678025820006.534569617403663.7357797337725210.23442867916959.10680512714377

11.91196170497680002.8560959042053303.354908380799220.4703986968742

50.29760182842900031.41133130717226.2531262978627228.990711298609557.6234202462443

02.634827351601898.364085753654217.517061145076131.581341235096744.0682008117777200

30.07853282886191.948597943872710.6872995257181030.6176972250132213.25419436324826.017310950011037.3265965753386426.0774252665845

03.429150303187767.2570744039058606.1742073223262414.119049876169700

7.3573881118974400014.11247387960284.0340142503341916.57719435218435.61932513247528

926.11165017423183.5429693486306244.31905007761291.4616748830563495.131382044527170.252988859346409.827276925709765.94407269541

02.8906886853318220.39177931676032.737695108391936.9253259017896112.355068999384700

13.2655937169060001.413623225313751.2122416560347703.37727116547756

01.8076141133082816.25809692037822.578526788136580000

59.72828052903070.3879903837217440.4105499662775373.3207649484487727.47744844944314.7924894754386526.258717176837875.6598651780198

89.63751182995031.6655872901197701.5839521698553337.9860755259311025.832794532153890.7521008894756

16.75653943188120001.3392220029288204.719344803612261.59976002575253

46.52570549987320000000

14.23624691570400015.018884803577304.8114295802681116.3097485552331

03.5153098585442437.1970647838394.457352839793891.406519591015691.2061499894215300

5.55891546232250.3172547219275740013.70926034018562.2859414085227126.391945928953814.1018302134022

12.3163126986320001.540719625387830.44041073008235807.97531741278828

5.4245922262440800024.97225861223411.7845639248690312.222342799937616.1581709664298

1.6395677627636600014.15211566016355.3937943347165216.62375950485899.39184958938985

12.57944231775570002.4129086087252002.88232625329551

82.867261277565210.465987552215534.32696139957212.9045684639158852.50618228545069.7402545407348944.178113192258360.4138260070534

08.8550210360797917.17813814595442.105252883984933.543005045723082.2787074167235900

5.2426897324299100018.4363555972057011.81249178389184.00418976505722

53.87871909635651.3689758548929615.38460445812520.433959498590518.94353009423617.920100475471735.058169558743430.8630626506718

5.120053715297042.520888871532626.6686629657513410.787998562257910.96231632307323.89227861451164023.4631470443704

2.833427784193510002.717450471962372.3303286203386912.76819500265333.24611500371144

035.454606643485236.073176861520423.342453029446921.278749602091244.916743465709500

17.04663407780650002.64054149634079010.855977119001220.1334712193262

71.159134224258500051.77054977960824.6413210474263375.951066421544943.6361879694935

02.139286427676772.2636745847045810.17216989815351.283933021156534.4041073008235800

152.26594527231200075.7210353733285.79767748538369173.12623827873171.212964302036

152.26594527231200075.7210353733285.79767748538369173.12623827873171.212964302036

152.26594527231200075.7210353733285.79767748538369173.12623827873171.212964302036

013.735305517129616.8831638188615.874974907160572.30368229310389000

013.735305517129616.8831638188615.874974907160572.30368229310389000

013.735305517129616.8831638188615.874974907160572.30368229310389000

12.23858000979070003.6115793369306303.181751819209571.07854788832993

12.23858000979070003.6115793369306303.181751819209571.07854788832993

12.23858000979070003.6115793369306303.181751819209571.07854788832993

12.23858000979070003.6115793369306303.181751819209571.07854788832993

714.45825341085730.019800103927844.922862350665618.0983326616324289.16876118887748.4572331524051254.492237813151425.181353336762

424.32334367865730.019800103927844.09208615627913.6184679388093127.76909751055331.2337608597586116.730228071675217.790514373375

259.00518709737115.731099062504424.51210884450390.73917767926582182.604751337223110.895575596669589.9788707927944160.505879476554

2.68361436112120006.434422956600521.1035579213557918.89546099530589.99206434475776

46.694889883509113.99093323700610016.7938439167274032.87810213183240.1219814458734

78.9692990676991.7401658254982716.5721251313074001.7912227454842200

7.004233482526360008.9567167555879503.945372255819861.33739938152911

31.150434331037600016.8896774912384.1600058527339318.661005430890422.2765866847677

25.92027193369120001.841430254027131.579104262466352.163033034988965.49917508852432

5.2301623973464300015.04824723721092.5809015902675816.497972395901416.7774115962434

012.978688566048319.579977311775111.45779472249389.0271471157653715.551485290525100

012.978688566048319.579977311775111.45779472249389.0271471157653715.551485290525100

54.631825391877500000016.2109015942923

5.3062374867623900000016.2109015942923

49.32558790511510000000

6.7870479481844500049.63296893994047.6751811826852835.363074967377544.7095723476303

6.7870479481844500049.63296893994047.6751811826852835.363074967377544.7095723476303

6.7870479481844500049.63296893994047.6751811826852835.363074967377544.7095723476303

270.79120831103800.830776194386534.47986472282314108.7243317099919.5482911099612785.1260187990416160.864147890853

270.79120831103800.830776194386534.47986472282314108.7243317099919.5482911099612785.1260187990416160.864147890853

67.795328565644200.830776194386534.4798647228231453.62597098802084.848966624139138.70437553516878.7765499034717

184.16437904641690.1539390081113109.95053355673381.0984300885268179.23788847059382.8903127664307100.166193267821206.595634252469

1.5043456792367600018.75601124720411.8558544940326210.16848519541218.9579809237374

1.5043456792367600018.75601124720411.8558544940326210.16848519541218.9579809237374

142.4565241072177.0076034358842426.809301359982218.327202544451109.99551099222726.211274039930776.9564368580246110.403529261411

39.52237972929514.5269415144292717.44716069281578.8908917453128753.041202506468516.891666759030439.742760818698126.9079222689681

25.09057997134250000.633252033059103003.02579045594822

3.6079499394881600013.84108015114891.318812351070820.77462497341048.26689178142997

3.6079499394881600013.84108015114891.318812351070820.77462497341048.26689178142997

26.8361436112120000000

26.8361436112120000000

58.368612354386204.11234216221332041.98460979181858.000794929829516.439051065916175.2287152110127

58.368612354386204.11234216221332041.98460979181858.000794929829516.439051065916175.2287152110127

026.32702490834475.9695289451483621.459997139975409.6783809635034300

026.32702490834475.9695289451483621.459997139975409.6783809635034300

026.32702490834475.9695289451483621.459997139975409.6783809635034300

38.545310045349332.159841344649540.547186040720616.783843620890737.960812153816628.07410005783269.3051232448581656.7763888385002

38.545310045349332.159841344649540.547186040720616.783843620890737.960812153816628.07410005783269.3051232448581656.7763888385002

38.545310045349300026.405414963407911.32187961768339.3051232448581656.7763888385002

07.319621274965791.106459774586542.9832283019696603.229020375267500

06.6562854344311638.30888897378188.7145303698442501.4119049876169700

018.18393463525251.131837292352295.0860849490767411.555397190408712.111295077264800

1.658199214613257.0829200265117330.42500641357531.679949271058686.899475713282774.091315589117373.7361479695263717.0974352752302

1.658199214613250003.1806522569559503.7361479695263717.0974352752302

08.7170923595053414.604579641505313.12558495892583.4878180512414112.711543346458100

08.7170923595053414.604579641505313.12558495892583.4878180512414112.711543346458100

08.7170923595053414.604579641505313.12558495892583.4878180512414112.711543346458100

08.7170923595053414.604579641505313.12558495892583.4878180512414112.711543346458100

237.9372467670984.536619078147245.760479293372749.0004527563897852.933371009584813.348971548695820.7850070948365108.399899881388

237.9372467670984.536619078147245.760479293372749.0004527563897852.933371009584813.348971548695820.7850070948365108.399899881388

43.45378266186500012.65383816821390018.7301633285404

43.45378266186500012.65383816821390018.7301633285404

43.45378266186500012.65383816821390018.7301633285404

191.0700949617014.536619078147245.760479293372749.0004527563897832.095398379028113.348971548695820.785007094836585.7592120454523

191.0700949617014.536619078147245.760479293372749.0004527563897832.095398379028113.348971548695820.785007094836585.7592120454523

191.0700949617014.536619078147245.760479293372749.0004527563897832.095398379028113.348971548695820.785007094836585.7592120454523

2142.1072071586345.3908819584084139.85016504316847.7922549242331060.35746604379234.4103417890181844.734000945352139.40698418719

43.55046243400528.9062653707793120.56171081106665.3898372446465910.174112160606115.45506549643393.85879929175834.74645809323778

43.55046243400528.9062653707793120.56171081106665.3898372446465910.174112160606115.45506549643393.85879929175834.74645809323778

43.55046243400528.9062653707793120.56171081106665.3898372446465910.174112160606115.45506549643393.85879929175834.74645809323778

43.55046243400528.9062653707793120.56171081106665.3898372446465910.174112160606115.45506549643393.85879929175834.74645809323778

122.851752046926.0320604002859414.63483186330911.766005098509874.707652359933419.606673864262257.9966119331487132.016697292928

122.851752046926.0320604002859414.63483186330911.766005098509874.707652359933419.606673864262257.9966119331487132.016697292928

122.851752046926.0320604002859414.63483186330911.766005098509874.707652359933419.606673864262257.9966119331487132.016697292928

122.851752046926.0320604002859414.63483186330911.766005098509874.707652359933419.606673864262257.9966119331487132.016697292928

88.815523552949400.4139941774040262.2324158098632128.67312740005948.8599406941064957.921567944329492.5201175816661

88.815523552949400.4139941774040262.2324158098632128.67312740005948.8599406941064957.921567944329492.5201175816661

64.636382808548400.4139941774040262.2324158098632127.70796395656938.8599406941064929.788884481861372.9286575498259

64.636382808548400.4139941774040262.2324158098632127.70796395656938.8599406941064929.788884481861372.9286575498259

24.1791407444010000.965163443490079028.132683462468119.5914600318402

24.1791407444010000.965163443490079028.132683462468119.5914600318402

141.4371794493842.3553759658259401.6729669382612644.203046439424316.251898111827854.4034491810891127.38881549484

141.4371794493842.3553759658259401.6729669382612644.203046439424316.251898111827854.4034491810891127.38881549484

78.11960744400192.3553759658259401.1199661807057918.37710192907887.2734499362086523.247142921497552.3477030649021

78.11960744400192.3553759658259401.1199661807057918.37710192907887.2734499362086523.247142921497552.3477030649021

63.3175720053816000.55300075755547525.82594451034558.9784481756191331.156306259591675.0411124299379

63.3175720053816000.55300075755547525.82594451034558.9784481756191331.156306259591675.0411124299379

1487.049996070413.335109555792162.41395375975119.94758144339874744.078579135471143.5220461504841530.135172606971540.49952648535

1487.049996070413.335109555792162.41395375975119.94758144339874744.078579135471143.5220461504841530.135172606971540.49952648535

6.424218340891570007.041443990242092.2643759235366521.711954238002412.6169752974445

6.424218340891570007.041443990242092.2643759235366521.711954238002412.6169752974445

10.28521803601520003.699084563155821.057373779272624.3451236297575511.7832544628116

04.2494180286255522.19596414134445.205476614548022.967110232637346.4825034319227900

04.2494180286255522.19596414134445.205476614548022.967110232637346.4825034319227900

3.279135525527310003.14491459114745011.08250633657263.75673983575593

3.279135525527310003.14491459114745011.08250633657263.75673983575593

197.21841545934900.717268981781394054.122506947462930.0237283695665170.911837255718154.610952575657

197.21841545934900.717268981781394054.122506947462930.0237283695665170.911837255718154.610952575657

54.04209796154102.183544510909730008.728699681017374.77642636260397

54.04209796154102.183544510909730008.728699681017374.77642636260397

1123.409682075040.67151107449033200.638598427011508626.85339359600693.37772999607261284.791820408681290.37896099413

40.34115756674640.67151107449033200.63859842701150819.54647060142264.6656903478487319.883085522490344.7725494896357

4.993512463778300010.64248663924422.2815955123087913.751297469587612.0772757837705

1078.07501204452000596.66443635533986.43044413591511251.15743741661233.52913572072

4.081721143663380009.7866223290952302.2991679812472.33811080686908

4.081721143663380009.7866223290952302.2991679812472.33811080686908

4.081721143663380009.7866223290952302.2991679812472.33811080686908

4.081721143663380009.7866223290952302.2991679812472.33811080686908

21.4590486597008000001.611671673129030

21.4590486597008000001.611671673129030

21.4590486597008000001.611671673129030

21.4590486597008000001.611671673129030

173.71168719008211.081099440879222.277387468321413.9044582663242116.86797011726622.7953160846827103.189407121689175.32874144643

173.7116871900826.3027752479126414.187534034459211.1779832198519114.57372914503521.8116117900316103.189407121689175.32874144643

94.35795011048880.8799329079878030.7985130412064690.83680491992356965.378280346769710.470027139895946.7391727632658100.553724057104

94.35795011048880.8799329079878030.7985130412064690.83680491992356965.378280346769710.470027139895946.7391727632658100.553724057104

05.4228423399248413.38902099325278.59508929378862.169747276062973.7212999673625600

05.4228423399248413.38902099325278.59508929378862.169747276062973.7212999673625600

04.778324192966568.089853433862272.726475046472282.294240972230520.98370429465116800

04.778324192966568.089853433862272.726475046472282.294240972230520.98370429465116800

04.778324192966568.089853433862272.726475046472282.294240972230520.98370429465116800

03.0885062333346714.70638918937217.34282462846847.414500625486713.1791238131772800

03.0885062333346714.70638918937217.34282462846847.414500625486713.1791238131772800

03.0885062333346714.70638918937217.34282462846847.414500625486713.1791238131772800

03.0885062333346714.70638918937217.34282462846847.414500625486713.1791238131772800

03.0885062333346714.70638918937217.34282462846847.414500625486713.1791238131772800

03.0885062333346714.70638918937217.34282462846847.414500625486713.1791238131772800

803.5117336665268.4116540480154920.68483482789931.3439594168469438.117447050091726.136413498241353.634769626815550.2676110808965

803.5117336665268.4116540480154920.68483482789931.3439594168469438.117447050091726.136413498241353.634769626815550.2676110808965

803.5117336665268.4116540480154920.68483482789931.3439594168469438.117447050091726.136413498241353.634769626815550.2676110808965

803.5117336665268.4116540480154920.68483482789931.3439594168469438.117447050091726.136413498241353.634769626815550.2676110808965

803.5117336665268.4116540480154920.68483482789931.3439594168469438.117447050091726.136413498241353.634769626815550.2676110808965

803.5117336665268.4116540480154920.68483482789931.3439594168469438.117447050091726.136413498241353.634769626815550.2676110808965

6174.96257452009351.4236352208551104.07139167646229.6318401624372628.12020438566778.8362487742882103.122901408074660.17369596036

6174.96257452009351.4236352208551104.07139167646229.6318401624372628.12020438566778.8362487742882103.122901408074660.17369596036

6174.96257452009351.4236352208551104.07139167646229.6318401624372628.12020438566778.8362487742882103.122901408074660.17369596036

6174.96257452009351.4236352208551104.07139167646229.6318401624372628.12020438566778.8362487742882103.122901408074660.17369596036

4963.07259492875279.474837892358880.760147450292162.5835485546222075.0840281424589.4197754218951648.867230097843782.01195912718

4963.07259492875279.474837892358880.760147450292162.5835485546222075.0840281424589.4197754218951648.867230097843782.01195912718

1142.8505534460365.997150906805196.20793921813967.0482916078147500.552243540556181.654320657519424.334252661963822.242598929526

1142.8505534460365.997150906805196.20793921813967.0482916078147500.552243540556181.654320657519424.334252661963822.242598929526

41503.38776655873362.391499131865952.090548494772827.5024839895221919.40337201676193.7757740227223709.428032587734475.1615005062

1738.4961585796652.8653858610541109.48668307959731.870295672298726.298358948617167.090913975695855.0016813794761411.59238003012

1551.5930071621651.384863825392195.387224237722829.9676504232225713.402185370349166.328933506188849.6700972499891285.44648099959

54.68711670352012.6497979615541712.150101842903023.57688630354935.1768162576939821.833051127682212.2980593678588

54.68711670352012.6497979615541712.150101842903023.57688630354935.1768162576939821.833051127682212.2980593678588

54.68711670352012.6497979615541712.150101842903023.57688630354935.1768162576939821.833051127682212.2980593678588

1480.3850015145448.735065863837983.237122394819929.9676504232225686.62647165409161.152117248494827.8370461223071264.98832126646

78.75285138570250005.643092713954090.96783809635034305.39273944164966

78.75285138570250005.643092713954090.96783809635034305.39273944164966

549.9379042115893.3528030304712914.14389627483186.21915436380981232.88703985032154.7410650211933185.075731222975491.180738891381

549.9379042115891.183666094501361.519962195479266.21915436380981231.58519148468453.6246750309845185.075731222975491.180738891381

02.1691369359699312.623934079352501.301848365637781.1163899902087700

01.302693969926086.892193567955291.238845272512550000

555.28008146496131.273439026311327.718930536284815.6534202504262315.18189753209177.6554291147151528.848311787892544.76866871618

04.182640728551896.0855287602259910.938503773888804.8435305629012600

24.5696936619960003.58842818733491012.912382845792239.3361164246757

28.42627225051299.421503863303747.476985749478782.239932361411574.678419721871728.0527481436595809.76996301441723

72.27218399748815.9485260361420212.5888025373877020.563494993576211.180116142070550.909559531508678.8600598869806

148.6783718822470.3604052868883590.38136094240.342740809551386109.01722480719520.1158271651518101.95828580913467.6968002242326

85.503553694950800061.76607124479524.11847732608387138.016746704274137.563944872724

94.47844886587631.4805220356620214.09945884187421.902645249075485.33137902118330.7619804695075723.55438941965752120.72400964596

94.47844886587631.4805220356620214.09945884187421.902645249075485.33137902118330.7619804695075723.55438941965752120.72400964596

94.47844886587631.4805220356620214.09945884187421.902645249075485.33137902118330.7619804695075723.55438941965752120.72400964596

94.47844886587631.4805220356620214.09945884187421.902645249075485.33137902118330.7619804695075723.55438941965752120.72400964596

2511.49445774535156.342040698095342.843604062961112.474951319571147.04409861594354.6546863109941099.630819441592069.67454648736

1467.730033576573.7979397603937129.39492684549961.5195689836744650.106936997452198.749930607115713.6807644661031400.45876348012

133.11735696060631.154283587839823.121595714317112.526553673351780.540863716000834.111687756833979.8242754035865212.39388190356

133.11735696060631.154283587839823.121595714317112.526553673351780.540863716000834.111687756833979.8242754035865212.39388190356

020.322240818048610.36376552977158.6842538931339813.261267877792111.90734142782541.868073984763190

133.11735696060610.832042769791312.75783018454563.8422997802177567.279595838208622.204346329008577.9562014188233212.39388190356

130.7312191494663.2038590214037913.43902667389975.0269674609044954.960698523526515.117287623130777.9681588746189155.775925663214

130.7312191494663.2038590214037913.43902667389975.0269674609044954.960698523526515.117287623130777.9681588746189155.775925663214

130.7312191494663.2038590214037913.43902667389975.0269674609044954.960698523526515.117287623130777.9681588746189155.775925663214

61.28854165266935.7813773706636712.38118191429433.3608605746276330.082549850184214.551065054914824.568530444456281.4584277529266

61.28854165266935.7813773706636712.38118191429433.3608605746276330.082549850184214.551065054914824.568530444456281.4584277529266

61.28854165266935.7813773706636712.38118191429433.3608605746276330.082549850184214.551065054914824.568530444456281.4584277529266

41.69186596741870007.997068531774946.8578242255681321.066442447953325.860533264986

41.69186596741870007.997068531774946.8578242255681321.066442447953325.860533264986

41.69186596741870007.997068531774946.8578242255681321.066442447953325.860533264986

20.22674685548040008.31378411719177006.62078901747087

20.22674685548040008.31378411719177006.62078901747087

20.22674685548040008.31378411719177006.62078901747087

914.18856511993129.216853673500364.496504400655536.381314821843431.69039115906997.408639297064498.371963798235864.954726954459

490.9026357283178.8765741969013822.190224484254626.9943716913419190.66565823653558.4333402641982336.120553921239532.848498487384

21.07170121096980007.073219459512140.86651208626312320.177896615203820.5196656010785

72.208592603364402.543716801369084.5722330676236346.168643070040912.372363293550830.50545558623675.8319236949498

256.73111696830012.206603877784293.367568571818316.1526803907661193.786761287937315.554291873351

33.935239740922300013.018483656377813.954874877609634.407316184475585.5313557954667

339.2671005602129.3706857647886335.67346997734726.34677686900462197.11121143940134.3941987101408139.004266955499325.282051679099

112.8926805739391.6897262363533912.5158239719536050.446852645036716.191376093585436.549402947110480.2789698999058

10.195390804259600020.7784095039567015.792974823150824.8207308799072

12.49328175393540008.626974614757211.643998958184147.8817368124255250.3814835507543

135.5329144524324.8327921371350810.22758672467564.45951470917774.911072588235816.558823658371367.5512167882332138.40636541723

251.36609314265214.461690948665528.512953482394614.0380398484822219.40989932504165.0627440506588162.391103415288274.677468543186

251.36609314265214.461690948665528.512953482394614.0380398484822219.40989932504165.0627440506588162.391103415288274.677468543186

76.25040603011311.9113296771866213.65162766964261.5164696479409834.9889170123515.9079449789224111.469105394825246.3837018534599

76.25040603011311.9113296771866213.65162766964261.5164696479409834.9889170123515.9079449789224111.469105394825246.3837018534599

81.31288680703400.351983637279314021.049159326254419.474737767236819.059769351786890.0834663735421

81.31288680703400.351983637279314021.049159326254419.474737767236819.059769351786890.0834663735421

40.67467907176662.004431695846147.069929218704843.8123777154111177.791299278116213.690471983259558.503825036747943.8836188129871

40.67467907176662.004431695846147.069929218704843.8123777154111177.791299278116213.690471983259558.503825036747943.8836188129871

51.431359246692710.54592957563277.439412956767818.7091924851300667.680108680800323.198614345718248.508675276473690.4388926034032

51.431359246692710.54592957563277.439412956767818.7091924851300667.680108680800323.198614345718248.508675276473690.4388926034032

361.19838773566347.2583773974286122.19225376664523.8167994110419167.29594265259671.371272246375134.032661154019200.486611894559

361.19838773566347.2583773974286122.19225376664523.8167994110419167.29594265259671.371272246375134.032661154019200.486611894559

218.10997521572840.381821297704698.721138651967521.238272622905379.138489702067951.725488361095981.1399247841513117.513888358257

218.10997521572840.381821297704698.721138651967521.238272622905379.138489702067951.725488361095981.1399247841513117.513888358257

143.0884125199356.8765560997239723.47111511467792.5785267881365888.157452950528419.645783885279152.892736369867982.9727235363029

36.05500071979364.067131754943638.607227781376712.5785267881365828.795823445415413.233994169270631.383697592033813.6451380747952

14.76248950597320001.089094936233940019.3313468996715

283.639330658819.725455744944330.87281821126852.5459387137777288.588457794127712.820795772208175.032480524578176.711524858102

283.639330658819.725455744944330.87281821126852.5459387137777288.588457794127712.820795772208175.032480524578176.711524858102

283.639330658817.1200678050921422.60218592972212.5459387137777288.588457794127711.479880420840175.032480524578176.711524858102

283.639330658817.1200678050921422.60218592972212.5459387137777288.588457794127711.479880420840175.032480524578176.711524858102

02.605387939852168.27063228154635001.3409153513680700

02.605387939852168.27063228154635001.3409153513680700

415.45206721064312.016654441127652.67372625579867.03777414836334232.3106744007834.9865694831136270.756376906429293.523615323098

415.45206721064312.016654441127652.67372625579867.03777414836334232.3106744007834.9865694831136270.756376906429293.523615323098

415.45206721064312.016654441127652.67372625579867.03777414836334232.3106744007834.9865694831136270.756376906429293.523615323098

415.45206721064312.016654441127652.67372625579867.03777414836334232.3106744007834.9865694831136270.756376906429293.523615323098

10.65120663401214.70144497494825.91150547801883.2914750763660342.38849739507997.7338236704399719.19889175581449.76203928123441

211.892332537027000102.0239791309215.3245564156731182.564919056868146.758006188617

6.080063786915245.2484744190269212.69404654573892.852132524498346.299941447861280.77178086139834527.39841844319340

34.407472300142300.956358642375192017.38330922933431.1484394636118924.359658629834634.2111781817305

01.1264841575689313.111815589665702.70432269190457000

1472.9930316172719.722712582514551.498373128421824.0673237141714768.594912080867147.7505097047991105.829079533891324.3644769659

164.8206942351790.47636817286367404.1711578123871541.644265553182720.714465476943271.1895619801888129.441910480408

164.8206942351790.47636817286367404.1711578123871541.644265553182720.714465476943271.1895619801888129.441910480408

164.8206942351790.47636817286367404.1711578123871541.644265553182720.714465476943271.1895619801888129.441910480408

164.8206942351790.47636817286367404.1711578123871541.644265553182720.714465476943271.1895619801888129.441910480408

584.721550275978017.40414878862785.48648021940415256.11409039123756.4961250260033239.925573819466335.48265840146

584.721550275978017.40414878862785.48648021940415256.11409039123756.4961250260033239.925573819466335.48265840146

517.58193835031602.119337212259711.56164298436441242.07138793613545.6693770111554223.965330066149318.960064169346

12.331396976278800013.14072293953632.25374505065623.087145740078141.56971758395436

77.0252184264578001.5616429843644154.499677415278217.899613210155658.713365064097996.6957746026212

8.979786516059420004.3061138248019006.85845836681597

214.59737484832802.119337212259710124.70996731038421.8965963502409115.228651707758170.608553359055

59.8068716801361015.28481157636813.9248372350397414.042702455101810.826748014847915.96024375331669.8019943249326

59.8068716801361015.28481157636813.9248372350397414.042702455101810.826748014847915.96024375331669.8019943249326

66.049363448990300026.011937716388810.536140047145486.289803037258665.2499397452047

66.049363448990300026.011937716388810.536140047145486.289803037258665.2499397452047

66.049363448990300026.011937716388810.536140047145486.289803037258665.2499397452047

66.049363448990300026.011937716388810.536140047145486.289803037258665.2499397452047

481.74216710720113.77970054570379.080478522075910.3031430197922366.05259265049633.0964329567098629.520463443138624.911052985109

481.74216710720113.77970054570379.080478522075910.3031430197922366.05259265049633.0964329567098629.520463443138624.911052985109

481.74216710720113.77970054570379.080478522075910.3031430197922366.05259265049633.0964329567098629.520463443138624.911052985109

000015.1295891141688009.03648230762915

73.714609443435500081.54775788680943.88958401873683142.06364105006372.8894590905905

13.46299838531238.57861012302490.8252191629859513.708249227086048.738435566440960.802755344130052.4462873609994119.9017765108499

249.5567850195035.201090422678848.255259359089956.5948937927062170.34985569635521.9719178701186306.492325238588366.217622351157

2549.3167372958161.780373720824251.491233943723162.6172896699681372.9134953466374.2023792528891763.894222372142642.96595636118

424.77255842762626.465792386246835.328565048293227.9137006116205135.30646064592172.6860930533276224.319587856478305.034345208299

71.525605317452301.394014292275710.02274819343489.4657749988185316.903087879921544.05329651217732.9640692630415

71.525605317452301.394014292275710.02274819343489.4657749988185316.903087879921544.05329651217732.9640692630415

71.525605317452301.394014292275710.02274819343489.4657749988185316.903087879921544.05329651217732.9640692630415

353.2469531101733.068187113378521.434537963562793.62515369017926117.01133402670148.8970751108479180.266291344301272.070275945257

353.2469531101733.068187113378521.434537963562793.62515369017926117.01133402670148.8970751108479180.266291344301272.070275945257

353.2469531101733.068187113378521.434537963562793.62515369017926117.01133402670148.8970751108479180.266291344301272.070275945257

188.890062944191002.1569581227745778.68852741830917.800749113115763.7071599206896131.927283523916

188.890062944191002.1569581227745778.68852741830917.800749113115763.7071599206896131.927283523916

95.7902093715444000.57300595291924147.20581509494216.4695740277660441.494870682814970.9352011997163

95.7902093715444000.57300595291924147.20581509494216.4695740277660441.494870682814970.9352011997163

58.7160445707813001.5839521698553326.65689510591648.5722802819601822.212289237874755.2274298176086

58.7160445707813001.5839521698553326.65689510591648.5722802819601822.212289237874755.2274298176086

1628.88957163141117.818652053043163.599118053452110.597431000246972.361473811241255.7756913945961303.338930030631850.12920134376

1628.88957163141117.818652053043163.599118053452110.597431000246972.361473811241255.7756913945961303.338930030631850.12920134376

1628.88957163141117.818652053043163.599118053452110.597431000246972.361473811241255.7756913945961303.338930030631850.12920134376

25.015119580451225.82916865649115.016709881773117.981615247482519.480809978190822.595278629232505.73171163512478

2.21093228615110.402377142550617.265132175596711.047492489027217.280391519222616.686440512619919.49294592796379.25078623587332

626.33339679102610.717282833145428.748934904150616.0007413018868288.83481329504852.8911986621949456.495378358454692.882880741588

5.6311370293014900014.04167551565841.605510688260110.99602077987717.4615432499621

5.6311370293014900014.04167551565841.605510688260110.99602077987717.4615432499621

5.6311370293014900014.04167551565841.605510688260110.99602077987717.4615432499621

5.6311370293014900014.04167551565841.605510688260110.99602077987717.4615432499621

105.4352103987183.6434721971369907.3897326065982687.44517081509997.4343669701955669.127037980641214.985418320136

105.4352103987183.6434721971369907.3897326065982687.44517081509997.4343669701955669.127037980641214.985418320136

105.4352103987183.6434721971369907.3897326065982687.44517081509997.4343669701955669.127037980641214.985418320136

105.4352103987183.6434721971369907.3897326065982687.44517081509997.4343669701955669.127037980641214.985418320136

102.16610122057112.395068205542531.514294310649113.173509180104942.923265712759513.95102271597478.847505543277754.4900522651523

102.16610122057112.395068205542531.514294310649113.173509180104942.923265712759513.95102271597478.847505543277754.4900522651523

102.16610122057112.395068205542531.514294310649113.173509180104942.923265712759513.95102271597478.847505543277754.4900522651523

76.629833315526812.39506820554254.0986798958936711.787551031481530.677754523479113.95102271597466.362150303341550.3106791978738

1450.68867635912245.000988474342389.04761682086217.122543904867978.870095212126339.9315955433471047.627816898981550.96251986368

1450.68867635912245.000988474342389.04761682086217.122543904867978.870095212126339.9315955433471047.627816898981550.96251986368

60.94380925086012.576599122837216.81603673295025035.07318288987486.4128508979549327.607566675584272.8051222381129

60.94380925086012.576599122837216.81603673295025035.07318288987486.4128508979549327.607566675584272.8051222381129

60.94380925086012.576599122837216.81603673295025035.07318288987486.4128508979549327.607566675584272.8051222381129

142.61671331639119.798490429725639.861374374045312.5520737988535110.76899230579228.069122967198766.3532850554231144.755120937327

142.61671331639119.798490429725639.861374374045312.5520737988535110.76899230579228.069122967198766.3532850554231144.755120937327

71.969327012677400049.43697577265854.6352485747147533.726160802459954.2210997190226

28.31900329862419.798490429725626.769019735162112.552073798853510.750776092244615.850631464756506.30848764872224

863.165122406674218.744749124342295.677295298753197.043715810157697.149000847345272.707848755659842.8195549543571073.91220482956

863.165122406674218.744749124342295.677295298753197.043715810157697.149000847345272.707848755659842.8195549543571073.91220482956

053.760850151053582.753076739251150.416984304880917.79108797823856.399296479037513.54422554619258.19342427012647

172.78281022233228.854324191107320.678401518305324.128578559102498.855397538467829.6244070465091137.76510805798213.323998184632

16.875869571121627.929403896632638.876795657688428.389106113655344.312333904202142.027823995223517.079533574977716.6451221727109

329.79897408186753.413254090713871.48258874539937.2751612303984226.06469793576960.2778304740147246.008749968556333.475715765249

107.34195322932632.461557441510757.41977959530536.09073147903472.332521700361652.0002843691025145.174415203606176.988273530973

200.4040571366871.355710584981211.434537963562790.4297544646894318.44285184653524.6516249592032115.292140526433642.1177130811009

200.4040571366871.355710584981211.434537963562790.4297544646894318.44285184653524.6516249592032115.292140526433642.1177130811009

200.4040571366871.355710584981211.434537963562790.4297544646894318.44285184653524.6516249592032115.292140526433642.1177130811009

1182.0935212473314.343857679020648.15358809571864.16359781901201502.84510200132676.3436471345629591.372618683326710.268053420543

1182.0935212473314.343857679020648.15358809571864.16359781901201502.84510200132676.3436471345629591.372618683326710.268053420543

1182.0935212473314.343857679020648.15358809571864.16359781901201502.84510200132676.3436471345629591.372618683326710.268053420543

1182.0935212473314.343857679020648.15358809571864.16359781901201502.84510200132676.3436471345629591.372618683326710.268053420543

325.54038074005302.496448669358160.418402459961785129.72948631344315.1824496000929157.26154164927300.79269561193

314.48247988253612.47839991408658.520796179209012.85818214393125198.85851671536224.3705301415897311.732549830864192.242038828317

353.866140185586027.2667220578395036.578083639547816.868540846581730.71656600551868.0668340583295

187.9746993160828.5631888927061734.937483200159815.660544052241957.351621858164216.279831792671850.1941483746144149.148422776424

187.9746993160828.5631888927061734.937483200159815.660544052241957.351621858164216.279831792671850.1941483746144149.148422776424

187.9746993160828.5631888927061734.937483200159815.660544052241957.351621858164216.279831792671850.1941483746144149.148422776424

187.9746993160828.5631888927061734.937483200159815.660544052241957.351621858164216.279831792671850.1941483746144149.148422776424

187.9746993160828.5631888927061734.937483200159815.660544052241957.351621858164216.279831792671850.1941483746144149.148422776424

80.38934806981151.2272748453514108.1698585603064119.24294615458353.000298098686056.1646441497185318.4716087024607

80.38934806981151.2272748453514108.1698585603064119.24294615458353.000298098686056.1646441497185318.4716087024607

80.38934806981151.2272748453514108.1698585603064119.24294615458353.000298098686056.1646441497185318.4716087024607

80.38934806981151.2272748453514108.1698585603064119.24294615458353.000298098686056.1646441497185318.4716087024607

80.38934806981151.2272748453514108.1698585603064119.24294615458353.000298098686056.1646441497185318.4716087024607

23778.45926681662225.165725044933885.04992191291870.9666298128613513.81321514453939.9365150474714284.922879228720930.0923011064

157.4713588399656.6957614388003210.733751304344215.222286659888580.529843834663433.463543771096584.1648189095865232.16729163757

157.4713588399656.6957614388003210.733751304344215.222286659888580.529843834663433.463543771096584.1648189095865232.16729163757

157.4713588399656.6957614388003210.733751304344215.222286659888580.529843834663433.463543771096584.1648189095865232.16729163757

146.24662569489100.7795909312252756.2761765437962861.118696081018521.752879907641666.777361051406219.30768219979

11.22473314507431.355710584981214.303613890688373.8677901822048617.27452639019362.5494482949479117.387457858180512.8596094377799

71.50839712671096.984229854797680.90713430048823315.292060662777443.271908900584121.122709104866557.962611932616973.8763946117774

71.50839712671096.984229854797680.90713430048823315.292060662777443.271908900584121.122709104866557.962611932616973.8763946117774

4.2918097319401600024.69682928930506.4466866925161114.7507284727476

4.2918097319401600024.69682928930506.4466866925161114.7507284727476

01.714575151593880.9071343004882336.522155993521922.058069107442083.5297624690424200

01.714575151593880.9071343004882336.522155993521922.058069107442083.5297624690424200

57.29959015009355.269654703203808.7699046692554815.158285267855916.427782325654846.727852114105847.7642636260397

57.29959015009355.269654703203808.7699046692554815.158285267855916.427782325654846.727852114105847.7642636260397

3652.65465879444266.767116765909558.978349703029220.7543001521441948.549592904535.9504418673222418.013144356433499.60990898322

131.893444219281016.4716139224133044.618938249344313.17777988442539.453722558198693.1403140707775

131.893444219281016.4716139224133044.618938249344313.17777988442539.453722558198693.1403140707775

007.5532815224326200000

131.89344421928108.91833239998069044.618938249344313.17777988442539.453722558198693.1403140707775

2136.44789317239137.014733262196294.8515496562595.48157103656611155.90800791545308.7536961674541458.302017858512257.86272450661

963.2156833109463.0442265068727127.37763612399235.1684705610693490.516911293324124.529301425816653.686546735159856.957349437436

963.2156833109463.0442265068727127.37763612399235.1684705610693490.516911293324124.529301425816653.686546735159856.957349437436

1039.3716388307544.9725869829922113.21897573502245.8848434196079541.860296627908147.436087161877684.8162475441081204.92183415364

2.1779332968054600017.7546857328585023.309102257642211.2281664494049

03.886370343612826.730224054386702.332478321767694.0003974649147400

007.151899412544914.820723995211862.028242018928433.4786064912302100

40.6363756897626002.8070038453132526.572537842923212.153106222525824.9707104798726118.503742667137

465.97617369782614.109313821262520.47674506391347.97886354218169298.47844991190239.4075486560723496.529416205953668.479368081805

9.859562897700395.733989031559874.044926716931155.452950092944586.8827229166915511.80445153581402.25912057690729

71.1709475106012000.84638665564788430.493528196829614.324405507845322.588009098205367.4002504587414

41.132385682784112.24343371517739.401272101924328.449212419993215.1143658952745913.1134370125843013.7028625156671

136.2245766146623.7594301698793530.40382501108539.3192715868769993.599747414996232.296720727678646.5378644011889172.994211549782

38.18195575244250006.305399222022762.9788298571913524.926288944399922.732731983178

70.334419992656509.67609920520781025.33647780754648.8019833340986326.846065775026140.3116907929509

16.617398535056609.6760992052078100000

16.617398535056609.6760992052078100000

53.717021457599900025.33647780754648.8019833340986326.846065775026140.3116907929509

53.717021457599900025.33647780754648.8019833340986326.846065775026140.3116907929509

235.37633175037796.3422808991444107.31051181149295.1551023296847151.98810606029992.7501688736263115.649550499106200.171903934608

235.37633175037796.3422808991444107.31051181149295.1551023296847151.98810606029992.7501688736263115.649550499106200.171903934608

235.37633175037796.3422808991444107.31051181149295.1551023296847151.98810606029992.7501688736263115.649550499106200.171903934608

1025.5498575835529.5558510241258116.39432958593330.1176267858936502.49924923605110.22824660792727.11873727613882.874821357762

33.488271410696300023.40891383068142.7070358785137520.034742344858751.7627036598547

33.488271410696300023.40891383068142.7070358785137520.034742344858751.7627036598547

912.93340554207829.5558510241258116.39432958593330.1176267858936456.08506976601694.369219063933693.572446109971812.791578224905

297.101495317372000157.59988660592511.8930735443411485.766554019863500.018687688814

110.140608113040.95176416578272511.95185915072788.1460397306845434.455571103948214.06600087136430.483008599049793.7852546429174

03.886370343612812.3370264866400000

82.982414310691100.5532298872932730.49720471699494631.358387852609512.048065271159432.269336462386985.5809673265399

000024.1290860872524.1383422050842111.33727659718355.764652506591

55.40705222014900026.1972934661594014.57649848209316.58817429324687

3.78061723793694.7757639669056727.54312890040543.0942321457639148.058154986374714.212997009198141.243554748459221.9120117514463

3.1895416587096211.528161082271110.591032332116611.837421157249910.646673089373719.231899597707900

253.5205461456947.239538528679443.939746616199626.759312641182993.167666632066164.738847103614272.9689946494774132.343817693573

57.143439342046530.336727810339633.45634301461724.541779603385434.218692543960828.49305260864318.818105392274220.2781627749655

57.143439342046530.336727810339633.45634301461724.541779603385434.218692543960828.49305260864318.818105392274220.2781627749655

57.143439342046530.336727810339633.45634301461724.541779603385434.218692543960828.49305260864318.818105392274220.2781627749655

86.840465551855700027.23954200821778.5348702291542715.04593685232163.78509258923335

86.840465551855700027.23954200821778.5348702291542715.04593685232163.78509258923335

86.840465551855700027.23954200821778.5348702291542715.04593685232163.78509258923335

84.274944349268816.902810718339810.48340360158262.2175330377974631.709432079887625.919701520332736.651362693550979.3795420924028

84.274944349268816.902810718339810.48340360158262.2175330377974631.709432079887625.919701520332736.651362693550979.3795420924028

84.274944349268816.902810718339810.48340360158262.2175330377974631.709432079887625.919701520332736.651362693550979.3795420924028

06.9606633019930704.413001070243710000

06.9606633019930704.413001070243710000

06.9606633019930704.413001070243710000

06.9606633019930704.413001070243710000

591.53819589601440.343065188062957.949381148442635.9369228620795318.99498717328953.9284803731183460.058649689367536.03376631477

591.53819589601440.343065188062957.949381148442635.9369228620795318.99498717328953.9284803731183460.058649689367536.03376631477

440.42703161122129.239149920597857.949381148442628.8971354405003280.13421969482248.404166163197400.937187314444455.613174361259

29.38465610518310003.84298487796966004.59061572607248

2.7794577311612500025.32405035062072.857426760653399.393743466237767.96071060433997

243.17326774411229.239149920597855.296257172821128.8971354405003173.80255686892232.1080812148326327.581356388535327.746006380394

75.403035147251300030.03090016388892.1223973688454431.314547053868820.9867888217216

5177.37629768073634.7219315492841051.20153293436557.4185397879413499.783738322381138.795673453953448.901398823655226.20734319248

78.445148191671139.848694908598357.295207616304134.587793988831890.402991565637865.014670183261399.1327262031925141.094657228896

5.5691396256387700029.36888916863352.508151108111417.319981141201325.9811998380389

5.5691396256387700029.36888916863352.508151108111417.319981141201325.9811998380389

72.876008566032339.848694908598357.295207616304134.587793988831861.034102397004362.506519075149981.8127450619912115.113457390857

02.467536726103367.833032689930143.5198937107896105.0798697967171400

72.876008566032337.38115818249549.46217492637431.067900278042261.034102397004357.426649278432881.8127450619912115.113457390857

557.74280225761569.37993386282576.57167529822544.3210809682608192.95315124643492.6380340106625290.679189935386277.598659332277

44.234879096810700.497460745429031034.97032977363784.1385912627518345.61896031833141.6729977002106

44.234879096810700.497460745429031034.97032977363784.1385912627518345.61896031833141.6729977002106

23.88365387695560006.514190085236250.68774741517158916.659186888037322.2691347810312

23.88365387695560006.514190085236250.68774741517158916.659186888037322.2691347810312

2.334744494175450002.239179188896981.9201907831590813.15124085273292.67479876305823

2.334744494175450002.239179188896981.9201907831590813.15124085273292.67479876305823

284.86384571945421.747791503146442.80446823196616.6835366779690462.236381452150232.237508984902474.788153616055598.7043751831023

04.961323842909955.2497985049531801.985087933419313.4045935871614900

2.382392340995368.5658774920445212.08525043589225.430693153789693.42731508504646.8578242255681305.45877298583313

50.16052624205072.3794104144568115.1065630448653032.07157692430579.9499681844180620.548813832395152.242163340981

203.324995524755000005.9507877161687213.616057051767

31.793974504638700011.085045489589023.718339448114847.9278500572891

31.793974504638700011.085045489589023.718339448114847.9278500572891

32.42700686354790009.996335664718681.9049511737689319.57029888799537.96071060433997

32.42700686354790009.996335664718681.9049511737689319.57029888799537.96071060433997

56.188097415449618.770953688777418.927685454348519.425300717866158.289323244525225.442208545864997.173009924119356.3887922432452

56.188097415449618.770953688777418.927685454348519.425300717866158.289323244525225.442208545864997.173009924119356.3887922432452

026.298746686101512.986343670147313.33854458825544.2089834377762814.437524685406600

026.298746686101512.986343670147313.33854458825544.2089834377762814.437524685406600

560.864659350321127.752119410828179.553415979471114.220293540534460.799054370803196.184498434248415.884215259024613.025297652916

170.80345339404733.884505044891941.813578044449938.1526801696296214.02094792339850.502722371846148.866077715704201.115223994489

170.80345339404733.884505044891941.813578044449938.1526801696296214.02094792339850.502722371846148.866077715704201.115223994489

11.177318758581826.308965252775944.673958248520723.547043717438344.544215205863827.656827374145755.352099067249244.5310848756177

11.177318758581826.308965252775944.673958248520723.547043717438344.544215205863827.656827374145755.352099067249244.5310848756177

198.04626051556622.598074082436930.006711511010620.298593889873564.861321754894834.537310146136895.6284167678799154.120059736712

04.6022806700677814.60963662891581.458903314340433.682860508054263.1582085249327100

49.029555914702517.995793412369113.95414780763417.542887629452722.56044371605716.367202719264246.615962008958719.4389444989697

95.206006740085117.985080635169337.688854752974711.091879865781868.920012756986246.152098613848365.54233122095891.4852639212306

95.206006740085117.985080635169337.688854752974711.091879865781868.920012756986246.152098613848365.54233122095891.4852639212306

86.31977883395150000000

86.31977883395150000000

86.31977883395150000000

225.23690568324443.848717741492580.866639556757244.2108175395427219.13026822853673.6317755242138213.575007839737273.989777230735

2.59446135619763.7013050891550312.72867812113656.1598139938818210.79690800150359.5247558688446324.577245486612314.8617162256347

2.59446135619763.7013050891550312.72867812113656.1598139938818210.79690800150359.5247558688446324.577245486612314.8617162256347

171.45095960285115.917286682049637.232849158459916.862153977425193.696949916822519.089143140987185.0576054462249144.589440941514

46.83900991401378.229960727650648.7084892846870415.653174384452626.32973350294627.9020196837822217.589108136371181.1795920887014

15.15032287887070001.529493981487010.65580286310077800.913524167711144

29.470405674585600025.50221428916833.249984960330946.224950038409326.1744624397341

9.4996187567763816.738327717540321.986779877180118.113512437454573.526981713133928.155853370085976.406770885168540.4216592645591

9.4996187567763816.738327717540321.986779877180118.113512437454573.526981713133928.155853370085976.406770885168540.4216592645591

41.6918659674187002.1847616135935639.985342658874713.006218358836127.533386021731374.1169607990273

41.6918659674187002.1847616135935639.985342658874713.006218358836127.533386021731374.1169607990273

009.902629955831420.741649845417212.808335103131670.8027553441300500

009.902629955831420.741649845417212.808335103131670.8027553441300500

009.902629955831420.741649845417212.808335103131670.8027553441300500

154.9810720516079.137197792109920.37969416622753.69263775076749238.66610517709836.3561978844382262.847992698067220.160838975615

6.4242183408915700011.44234648414340.75479197451221616.54244132419231.05141460812037

6.4242183408915700011.44234648414340.75479197451221616.54244132419231.05141460812037

18.86027692560950009.755590134653590.70803494954243312.317444602703712.5942904192606

18.86027692560950009.755590134653590.70803494954243312.317444602703712.5942904192606

00004.680558505219441.60551068826019.896418701889288.9458152610643

00004.680558505219441.60551068826019.896418701889288.9458152610643

24.03711936091778.134263509887242.869075927125572.5785267881365834.605457257613015.728145190069525.3454609264146

24.03711936091778.134263509887242.869075927125572.5785267881365834.605457257613015.728145190069525.3454609264146

102.784156322987000.63722213729812168.53051836056828.0887247488361194.287651415696154.745231719126

51.1537495927203000123.14929079330819.1223166378203153.50364040323492.5719853456264

6.344414386346330003.04236302839264010.721120260381.81711872490369

45.2859923439203000.6372221372981229.75920617427758.9664081110158122.67455319436752.8426479770843

132.8068078899080.87993290798780319.78722525742370.6176972250132286.474608295830716.050079270454277.3049468639748171.536962470551

132.8068078899080.87993290798780319.78722525742370.6176972250132286.474608295830716.050079270454277.3049468639748171.536962470551

132.8068078899080.87993290798780319.78722525742370.6176972250132286.474608295830716.050079270454277.3049468639748171.536962470551

107.0778217195073.8322075263208124.36952206406171.464986602192775.978716713365714.377752084605844.9528993238188133.140162387008

1.380960860119552.8646464449234324.36952206406170.54484841223524816.21044342482233.8733027795685215.557461576576710.0199480477339

1.380960860119552.8646464449234324.36952206406170.54484841223524816.21044342482233.8733027795685215.557461576576710.0199480477339

105.6968608593870.96756108139737600.92013818995745159.768273288543410.504449305037229.3954377472421123.120214339274

105.6968608593870.96756108139737600.92013818995745159.768273288543410.504449305037229.3954377472421123.120214339274

132.53545102411915.868404507625839.926471685517227.6995227708741101.05038126261634.2023208457254108.237447559227150.633403336612

132.53545102411915.868404507625839.926471685517227.6995227708741101.05038126261634.2023208457254108.237447559227150.633403336612

111.29310357711315.868404507625839.926471685517226.893147120765976.089039484747930.7110648763453102.259610807985136.043591901748

21.2423474470061000.80637565010816724.9613417778683.491255969380155.9778367512422114.5898114348631

62.88239582465544.1502804678007213.158719475749410.159455384601160.97981111115998.3692363963777261.926910724239580.9074824702148

61.06216882815690.3761003558334970.7959371926864492.8613329519967344.09478147426963.4842171468612255.091546455667376.7367983282321

61.06216882815690.3761003558334970.7959371926864492.8613329519967344.09478147426963.4842171468612255.091546455667376.7367983282321

1.820226996498533.7741801119672212.3627822830637.2981224326043616.88502963689034.885019249516516.835364268572164.17068414198268

1.820226996498533.7741801119672212.3627822830637.2981224326043616.88502963689034.885019249516516.835364268572164.17068414198268

64.29588968469400081.97798163596617.1435669016334874.650079854690569.8240640958976

64.29588968469400081.97798163596617.1435669016334874.650079854690569.8240640958976

64.29588968469400081.97798163596617.1435669016334874.650079854690569.8240640958976

126.57154591791310.810649385055516.65585412387484.6731940489012851.834913175953525.975289729093258.1658330185268142.566424541338

09.5898000624546214.30594431689573.398749774305041.3587252359811810.604304395923200

09.5898000624546214.30594431689573.398749774305041.3587252359811810.604304395923200

126.5715459179131.220849322600882.349909806979041.2744442745962450.476187939972315.3709853331758.1658330185268142.566424541338

2.216529583077960001.41720201828923014.98242628792350

124.3550163348351.220849322600882.349909806979041.2744442745962449.058985921683115.3709853331743.1834067306033142.566424541338

71.41045311661430.43996645399390.4655481693071682.0920122998089229.63431872604754.0758766623659618.610246489716347.737045798217

71.41045311661430.43996645399390.4655481693071682.0920122998089229.63431872604754.0758766623659618.610246489716347.737045798217

71.41045311661430.43996645399390.4655481693071682.0920122998089229.63431872604754.0758766623659618.610246489716347.737045798217

80.949170418491800046.990074219553510.511993338462145.597367920059282.9773338904925

80.949170418491800046.990074219553510.511993338462145.597367920059282.9773338904925

80.949170418491800046.990074219553510.511993338462145.597367920059282.9773338904925

221.11706615996425.395763316949856.367089686722915.4375379542387140.99783431300239.5492317713676132.930332069402116.051594213182

216.98721151224817.820276935732.355754439199110.028920788879129.05099412833834.1867951577389117.421793327972112.897350388821

02.2102580153248113.4479435636360.52548176251124601.7063306722385200

216.98721151224815.610018920375218.90781087556319.50343902636777129.05099412833832.4804644855004117.421793327972112.897350388821

4.1298546477167.5754863812497824.01133524752395.4086171653596611.94684018466385.3624366136286815.50853874143023.15424382436111

4.1298546477167.5754863812497824.01133524752395.4086171653596611.94684018466385.3624366136286815.50853874143023.15424382436111

160.65672268600910.882631314766521.29928469912844.03287104453808173.34093472937137.4407589718661117.585718599548210.557582058424

53.699123366035500035.82686702235187.6807631326363313.151240852732958.845572787281

53.699123366035500035.82686702235187.6807631326363313.151240852732958.845572787281

106.95759931997310.882631314766521.29928469912844.03287104453808137.51406770701929.7599958392298104.434477746815151.712009271143

106.95759931997310.882631314766521.29928469912844.03287104453808137.51406770701929.7599958392298104.434477746815151.712009271143

332.5562660080858.573495954660285.321923087167641.4183371298105267.784647530715101.783002524792330.934135238064361.402856336663

332.5562660080858.573495954660285.321923087167641.4183371298105267.784647530715101.783002524792330.934135238064361.402856336663

8.66157020657687.9920050426446719.12676520829487.321029752406077.7436162236303914.62074200374938.502957447887620

34.846932748887416.711070491591110.42565618589294.1643812916384236.892089327265424.565128505385524.535897113307644.9126657976195

141.28030470526117.904483774880721.307593397940824.8537812136178147.38448938028836.0188830341736215.23854759526191.625819546091

9.8565468938174500014.30646378587785.5267343148456719.230649607661726.605983065752

87.854181859713715.965936645543834.46190829503915.0791448721481742.071156009350220.531982528337642.076666505224490.2976773228608

49.42503465492380000000

172.3861072684247.777360901622629.0280862209874415.6920625269114207.66458945609645.1850351653752198.412630917339236.651403502533

172.3861072684247.777360901622629.0280862209874415.6920625269114207.66458945609645.1850351653752198.412630917339236.651403502533

28.895224492705800029.84220490327864.3150354677732235.089742650753417.0272585362728

62.58766960419924.758820828913645.0355210149550811.19690326966738.377157095523113.769581733007143.152040859061253.2396327617998

12.953869582566100045.30976165522625.0135529586398858.373653915524431.4271395137389

17.6874582892083.018540072708983.992565206032362.8705929291876443.615561073734212.222411922792324.993782398009163.5818641824033

21.398285783839800026.93880521156192.8307872107851522.3514980690134.6337672575184

306.67403114729255.707674738085199.100579877643453.2616676024032208.41492738247175.8475255900717118.029362603792291.34089849616

36.33843570701090.90732381562944600.43142666105008822.87099366091282.3348623336078319.82920556589131.2233318644929

36.33843570701090.90732381562944600.43142666105008822.87099366091282.3348623336078319.82920556589131.2233318644929

68.951825636900415.162242102353548.80385627402744.9320711490297552.324230057249413.318063412279623.739470793813579.1723373159552

24.32025514766090003.33211188823955000

44.631570489239515.162242102353548.80385627402744.9320711490297548.992118169009813.318063412279623.739470793813579.1723373159552

121.90389356399114.771724382962720.842233425905624.752007970770986.703535775291520.278972071781739.9666947566458111.76257789652

84.714487730043914.771724382962720.842233425905624.752007970770980.91255511435120.278972071781735.4317841177724104.84499488861

37.18940583394680005.7909806609404704.53491063887346.9175830079092

022.216586475585223.112404050920414.59641999562886.601993008697129.330160454527500

017.119707336420923.112404050920414.59641999562883.5430050457230818.83731464491500

05.09687913916432003.0589879629740210.492845809612500

02.649797961554175.607739312109077.559771719764063.180652256955952.7275437260782300

02.649797961554175.607739312109077.559771719764063.180652256955952.7275437260782300

16.4240684114074000008.539766787488860

16.4240684114074000008.539766787488860

36.777661674608921.943326892945824.732246494443418.723510083289836.504588200072824.428791441813361.865653432571293.1122230598047

36.777661674608921.943326892945824.732246494443418.723510083289836.504588200072824.428791441813361.865653432571293.1122230598047

8.3511640469924213.1989936198179.310963386143372.092012299808927.813210468037839.839340332240729.594276841261631.5708720755253

26.2962563008140006.898879543256551.420259454999323.473038957587948.5252986093002

2.1302413268024200021.79249818877841.1679992598291228.798337633721713.0160523749792

08.744333273128815.421283108316.6314977834809012.001192394744200

100.13217053261311.14036213122788.71665621409617.3611055196596141.074029402210421.047001055421821.055511035386391.4831832141677

100.1321705326130.4971902358566470.526099210517696041.07402940221049.0956061394939121.055511035386391.4831832141677

100.1321705326130.4971902358566470.526099210517696041.07402940221049.0956061394939121.055511035386391.4831832141677

11.224733145074300000019.2894141566699

11.224733145074300000019.2894141566699

11.224733145074300000019.2894141566699

41.80897795047340.66244949038854415.42128310831.2599619532940111.7934297168032.0379961548786931.400434620289140.3849532343765

41.80897795047340.66244949038854415.42128310831.2599619532940111.7934297168032.0379961548786931.400434620289140.3849532343765

41.80897795047340.66244949038854415.42128310831.2599619532940111.7934297168032.0379961548786931.400434620289140.3849532343765

200.45998726781671.9066012244804105.77342792843953.3662109534321166.82389421468571.412480109337593.4154686507864290.233910484919

86.846866559894960.127585484250467.115496166341450.980538263812695.554945361072759.035831666815559.5541600835836122.100810151674

86.846866559894960.127585484250467.115496166341450.980538263812695.554945361072759.035831666815559.5541600835836122.100810151674

43.73183494441774.0705125793450720.30533037701340.55300075755547529.3927808833342.8006168621197315.081698225611169.5284082140518

43.731834944417700023.11079438081752.2020536504117915.081698225611169.5284082140518

69.8812857635037.708503160884918.35260138508421.8326719320640241.87616797027849.576031580402218.779610341591873.0367627664313

69.8812857635037.708503160884918.35260138508421.8326719320640241.87616797027849.576031580402218.779610341591873.0367627664313

559.8681618339695.9211388699953193.732374895862527.9061967183914249.63829039680761.8403505571792436.769256328490.867371394691

34.01001114039180006.421741407413771.2372363293550804.30863202812215

34.01001114039180006.421741407413771.2372363293550804.30863202812215

34.01001114039180006.421741407413771.2372363293550804.30863202812215

471.7097838057963.4467944237223146.702558577720814.7760668893275199.57027173836949.012093498366331.559824054812415.209260593569

3.1268899475564100018.4932209797296014.09061519935665.97053295325497

3.1268899475564100018.4932209797296014.09061519935665.97053295325497

128.43386280644500030.49862410647518.29295535583854118.20711697960678.2305780395595

128.43386280644500030.49862410647518.29295535583854118.20711697960678.2305780395595

90.802697633208802.61101089664338042.48433228779121.188404283675546.115465590197793.7081210278615

90.802697633208802.61101089664338042.48433228779121.188404283675546.115465590197793.7081210278615

16.03533306439180003.07579558914422012.64542389685853.67417412507998

34.13611755881650001.243988438276105.3029196986826126.8349214340107

34.13611755881650001.243988438276105.3029196986826126.8349214340107

02.441698645201769.0428466394219700000

02.441698645201769.0428466394219700000

130.620904864861.005095778520555.317683830448270.9558332059471848.17663658878315.7087724626730668.7594250531758110.651631539862

130.620904864861.005095778520555.317683830448270.9558332059471848.17663658878315.7087724626730668.7594250531758110.651631539862

22.23566184929014.51414880781174.603874818956663.5542526807888709.3937434662377628.6585581756239

0014.51414880781174.603874818956660000

22.235661849290003.5542526807888709.3937434662377628.6585581756239

42.026506349287805.861777228692199.2163588644236942.48461668210489.1283036346810840.122561602842845.1711838171459

42.026506349287805.861777228692199.2163588644236942.48461668210489.1283036346810840.122561602842845.1711838171459

45.40007169524980.94025088958374214.5639571427734014.64999618763885.1068903807422451.662312558041150.904676494372

45.40007169524980.94025088958374214.5639571427734014.64999618763885.1068903807422451.662312558041150.904676494372

45.40007169524980.94025088958374214.5639571427734014.64999618763885.1068903807422451.662312558041150.904676494372

98.20446377235216.4005442374573318.72623140611399.6765078012980131.299860886079912.113694252970364.622140367910142.3377185236786

98.20446377235216.4005442374573318.72623140611399.6765078012980131.299860886079912.113694252970364.622140367910142.3377185236786

98.20446377235216.4005442374573318.72623140611399.6765078012980131.299860886079912.113694252970364.622140367910142.3377185236786

98.20446377235216.4005442374573318.72623140611399.6765078012980131.299860886079912.113694252970364.622140367910142.3377185236786

1098.3320857379281.26070394479435.761913601663245.543300873085564.695816368309353.713190501118492.711588080678666.650501453779

15.451636050978300020.46317927949154.0751077741067126.850951024908423.9347388335997

15.451636050978300020.46317927949154.0751077741067126.850951024908423.9347388335997

15.451636050978300020.46317927949154.0751077741067126.850951024908423.9347388335997

58.832918348400290.6928618724946152.86618121848580.868405270515473.902063758909586.033645001881748.017416216473127.3497436633577

2.8895352650686390.6928618724946152.86618121848580.868405270515455.414472013845985.037695425554301.10346483624514

2.8895352650686390.6928618724946152.86618121848580.868405270515455.414472013845985.037695425554301.10346483624514

55.943383083331500018.48759174506360.99594957632732448.017416216473126.2462788271126

55.943383083331500018.48759174506360.99594957632732448.017416216473126.2462788271126

69.521506529119626.697674534383642.992333987788829.945203170257291.043375001429949.595527244158270.360143062785195.5223434322705

41.079690858977600039.12657352002541.2699674491792923.232170724631413.0113915597562

41.079690858977600039.12657352002541.2699674491792923.232170724631413.0113915597562

28.441815670141926.697674534383642.992333987788829.945203170257251.916801481404548.325559794978947.127972338153782.5109518725143

28.441815670141926.697674534383642.992333987788829.945203170257251.916801481404548.325559794978947.127972338153782.5109518725143

107.83039814607110.743760866373320.54182858386787.731625826817828.009450376996220.627396756247827.055548384672852.8912211014899

107.83039814607110.743760866373320.54182858386787.731625826817828.009450376996220.627396756247827.055548384672852.8912211014899

79.427987135795200.932856445114554020.26732011469436.8059692219721611.808770141867944.7926800394962

14.505122354469710.743760866373319.60897213875327.73162582681787.7421302623019213.821427534275615.24677824280498.09854106199369

137.1911828842410000000

137.1911828842410000000

137.1911828842410000000

510.033876108621128.720921049003185.3667205078103.978395622134305.777776823967158.767959697104278.329114215103431.625846781719

185.7491466558118.545945258206162.583670468406286.9660723700443612.18946655793578.8866427571306215.316869765517811.7685071506355

15.18258702859760008.08952019110182011.40280998791875.79797419738055

148.5272725089290000000

22.03928711828410004.099946366833896.373304035718223.914059777599065.97053295325497

274.818691513036111.945015063147171.17173099314595.707892053385265.3082342007138.605418203252215.786908106927402.966437631408

11.22473314507430000000

000011.27103618572311.61089830801936024.6835456322488

195.05661754591703.163340124779471.89532738273287139.09283856023235.0344269584267154.748980561556329.114851118814

011.9274793154357103.03783553656.2385839938035820.75965360550279.2080760061976300

13.68014352055930001.45779895110481010.27440691619761.74140544469937

19.816504194390400022.694383671253212.97426204837224.4429867745719118.0729646152583

5.73647295866205100.01753574771163.859111504240386.57509192108448.504607198841376.975920081906413.73286821231325.75048874121855

86.76415349976346.1802495227358720.00847827610695.9933325345877242.236425673808520.703215297188126.6579206474315189.766128460212

86.76415349976346.1802495227358720.00847827610695.9933325345877242.236425673808520.703215297188126.6579206474315189.766128460212

86.76415349976346.1802495227358720.00847827610695.9933325345877242.236425673808520.703215297188126.6579206474315189.766128460212

86.76415349976346.1802495227358720.00847827610695.9933325345877242.236425673808520.703215297188126.6579206474315189.766128460212

1321.6689722813676.5029509296716201.33163716155858.1178845149575363.168481816067108.794323512761330.774297429169499.789516854643

03.1942769947502411.83002539814791.518858245066751.91710546994605000

03.1942769947502411.83002539814791.518858245066751.91710546994605000

03.1942769947502411.83002539814791.518858245066751.91710546994605000

7.7948941941560300020.69353509698323.3457061320904240.549507040197343.2464359063461

7.7948941941560300020.69353509698323.3457061320904240.549507040197343.2464359063461

7.7948941941560300020.69353509698323.3457061320904240.549507040197343.2464359063461

214.91933955049413.852409145550503.2933658977190111.26436240192068.5180274500456252.862830878632132.7793966061057

178.78638904539813.852409145550503.2933658977190111.26436240192068.5180274500456252.862830878632132.7793966061057

013.852409145550503.293365897719011.385630686198634.7529474830670300

56.07964716401820009.8787317157223.7650799669785952.862830878632132.7793966061057

01.183666094501363.757469995930962.251302576444120000

01.183666094501363.757469995930962.251302576444120000

01.183666094501363.757469995930962.251302576444120000

1.0098375839859213.905480615460921.16441107705517.600257772567112.781640276623310.97097835232191.516867457062611.54255984028733

1.0098375839859213.905480615460921.16441107705517.600257772567112.781640276623310.97097835232191.516867457062611.54255984028733

1.0098375839859213.905480615460921.16441107705517.600257772567112.781640276623310.97097835232191.516867457062611.54255984028733

182.8027969340670000000

182.8027969340670000000

182.8027969340670000000

132.832713294626001.56716115745403114.15570161723224.993010229593177.5527968630356158.665003508161

5.3385925933889800010.240148729711802.004762325111720

5.3385925933889800010.240148729711802.004762325111720

9.9280611729861700018.6368019849352.2643759235366530.601119986815516.1054436329962

9.9280611729861700018.6368019849352.2643759235366530.601119986815516.1054436329962

44.279636958499800032.81555707866283.3106737640673722.67455319436753.0348030606374

44.279636958499800032.81555707866283.3106737640673722.67455319436753.0348030606374

46.4061405644413001.5671611574540323.73688186109886.785126442258249.2941631468076751.9837215435346

46.4061405644413001.5671611574540323.73688186109886.785126442258249.2941631468076751.9837215435346

26.880282005309600028.726311962823112.632834099730812.978198209933737.5410352709927

26.880282005309600028.726311962823112.632834099730812.978198209933737.5410352709927

28.87049643335239.4533999429069917.4139996386828.750765145664011.096448261594664.7550385419268606.47128732997958

28.87049643335239.4533999429069917.4139996386828.750765145664011.096448261594664.7550385419268606.47128732997958

28.87049643335239.4533999429069917.4139996386828.750765145664011.096448261594664.7550385419268606.47128732997958

1.523984656772490004.384815644054151.253388239659976.8674886959440614.8405936592656

1.523984656772490004.384815644054151.253388239659976.8674886959440614.8405936592656

1.523984656772490004.384815644054151.253388239659976.8674886959440614.8405936592656

255.69336057951815.882065831305989.504394960555410.9801104619275102.5359635925529.1276192963977.678600161440597.4355537823255

255.69336057951815.882065831305989.504394960555410.9801104619275102.5359635925529.1276192963977.678600161440597.4355537823255

39.11298766015590000000

2.415919385529230005.560875469115030.7947809532943229.7981099068373911.6247462798474

35.6449541095490000000

3.33534927739350003.19882741270999018.78748693247553.82114109008318

02.2207830534930328.1989176837486013.32844755295824.5718828170454400

01.3401277046940706.37222137298123.217211478300276.897237008473700

02.1998322699695113.966445079215004.528751847073300

20.507890827216800015.12958911416883.8922786145116417.771947098287719.8802610767841

21.8609035035155014.95397149895751.679949271058684.19321945486326008.76572628343053

31.456138394579401.47748820199281010.0561939621122004.00418976505722

87.98209950477330000000

87.98209950477330000000

87.98209950477330000000

130.03413180254400038.64002822944373.8713523854013829.712504543835452.6817120157286

130.03413180254400038.64002822944373.8713523854013829.712504543835452.6817120157286

16.78743275679250000010.1834829611870

54.88545026795531.6306448994179613.8036659990377033.53247822158335.7324239052943912.933300972515271.3445707065718

54.88545026795531.6306448994179613.8036659990377033.53247822158335.7324239052943912.933300972515271.3445707065718

47.80188080747151.6306448994179613.8036659990377026.73885204167745.7324239052943911.337276597183564.8523406991489

7.083569460483780006.793626179905901.596024375331666.49223000742288

201.511342858619001.2778630520839888.408296411902716.158468191616488.2477116225131183.52361316158

20.7320207816126000.6058833436605073.8237349537175300.8983088014161793.65409667084459

20.7320207816126000.6058833436605073.8237349537175300.8983088014161793.65409667084459

20.7320207816126000.6058833436605073.8237349537175300.8983088014161793.65409667084459

119.168009036265000.67197970842347262.814763788353410.824604905063450.8181782301722146.434531952507

119.168009036265000.67197970842347262.814763788353410.824604905063450.8181782301722146.434531952507

119.168009036265000.67197970842347262.814763788353410.824604905063450.8181782301722146.434531952507

118.8294447270383.4631022863876512.68045734346184.3756257698762397.950878936297214.909187175435479.1863925381994136.375748568243

118.8294447270383.4631022863876512.68045734346184.3756257698762397.950878936297214.909187175435479.1863925381994136.375748568243

82.611743620983502.908555175826182.1800485047302356.57294139321129.2783923204303445.3644214456448113.411146068387

82.611743620983502.908555175826182.1800485047302356.57294139321129.2783923204303445.3644214456448113.411146068387

17.221998860544600031.6949207359823.7324282567386433.821971092554619.9970003219066

17.221998860544600031.6949207359823.7324282567386433.821971092554619.9970003219066

03.463102286387659.771902167635632.1955772651465.542522744794521.1882368707667600

03.463102286387659.771902167635632.1955772651465.542522744794521.1882368707667600

92.164376495721300091.05416914495629.3635613688841948.26880337855652.9260152677112

92.164376495721300091.05416914495629.3635613688841948.26880337855652.9260152677112

92.164376495721300091.05416914495629.3635613688841948.26880337855652.9260152677112

2.4690614363107600017.04958773271823.2490537786109611.682574361564712.4461871377498

114.6084136844440.42864378789847110.66765986633554.3911545302919977.08162628981445.6829175751583182.9628974434181106.575350475547

114.6084136844440.42864378789847110.66765986633554.3911545302919977.08162628981445.6829175751583182.9628974434181106.575350475547

114.6084136844440.42864378789847110.66765986633554.3911545302919977.08162628981445.6829175751583182.9628974434181106.575350475547

114.6084136844440.42864378789847110.66765986633554.3911545302919977.08162628981445.6829175751583182.9628974434181106.575350475547

5.1200537152970400019.64192270962272.105472349955139.6134801555064820.5302536638241

5.1200537152970400019.64192270962272.105472349955139.6134801555064820.5302536638241

5.1200537152970400019.64192270962272.105472349955139.6134801555064820.5302536638241

5.1200537152970400019.64192270962272.105472349955139.6134801555064820.5302536638241

2605.91330532207400.663966102844522.971700468585284.5035169676231582.00213383587530.1061857487152095.602309099292333.45594463497

125.2363681428794.7588208289136416.11366724785636.335808679421365.395350402091313.2963137812525163.229796596131122.273973339984

125.2363681428794.7588208289136416.11366724785636.335808679421365.395350402091313.2963137812525163.229796596131122.273973339984

125.2363681428794.7588208289136416.11366724785636.335808679421365.395350402091313.2963137812525163.229796596131122.273973339984

120.429945464266011.63870423267922.0920122998089224.74502523707095.2733252791357330.47565835132944.8652862359792

59.21453427256570000000

59.21453427256570000000

61.2154111917002011.63870423267922.0920122998089224.74502523707095.2733252791357330.47565835132944.8652862359792

61.2154111917002011.63870423267922.0920122998089224.74502523707095.2733252791357330.47565835132944.8652862359792

4.339673780995270001.040510775509750.892281962434517014.9152347382429

4.339673780995270001.040510775509750.892281962434517014.9152347382429

4.339673780995270001.040510775509750.892281962434517014.9152347382429

17.009225824429348.496972892512135.731780586484421.255095440262430.494930664816542.914349895926623.78757443570715.93727935199633

038.185434720282715.36702102839749.7690055007826213.131475421633728.653093172345100

038.185434720282715.36702102839749.7690055007826213.131475421633728.653093172345100

17.009225824429310.311538172229420.36475955808711.486089939479717.363455243182714.261256723581523.78757443570715.93727935199633

17.009225824429310.311538172229420.36475955808711.486089939479717.363455243182714.261256723581523.78757443570715.93727935199633

59.008781677128600040.4334200702855.9299722973218944.559999364571581.6924634856584

19.38020332079230001.093349213328612.812779467518192.568601729049383.91816225057359

19.38020332079230001.093349213328612.812779467518192.568601729049383.91816225057359

39.628578356336300039.34007085695643.117192829803741.991397635522177.7743012350848

39.628578356336300039.34007085695643.117192829803741.991397635522177.7743012350848

99.000364635881512.166348522091849.154675710986215.464007186658223.968264457852628.15195431410861.896813584528780

99.000364635881512.166348522091849.154675710986215.464007186658223.968264457852628.15195431410861.896813584528780

99.000364635881512.166348522091849.154675710986215.464007186658223.968264457852628.15195431410861.896813584528780

38.044131427502125.13133061902418.01693541899516.6968416420097614.625291995150513.570087337254457.291375047579560.1892979583583

10.86649698086980005.95526380025794022.73485785711824.8983927412336

10.86649698086980005.95526380025794022.73485785711824.8983927412336

27.177634446632323.25082883985658.067720510414476.696841642009768.670028194892613.570087337254434.556517190461535.2909052171247

27.177634446632323.25082883985658.067720510414476.696841642009768.670028194892613.570087337254434.556517190461535.2909052171247

123.395274773466.302222178831556.645057079146911.19866650691755232.43593585229731.3265589247548286.785803000977190.391574465796

123.395274773466.302222178831556.645057079146911.19866650691755232.43593585229731.3265589247548286.785803000977190.391574465796

123.395274773466.302222178831556.645057079146911.19866650691755232.43593585229731.3265589247548286.785803000977190.391574465796

61.71388459408292.286100202125173.628537201952932.1740519978406520.64972483903116.2159339077476520.678051655240318.5852993964807

61.71388459408292.286100202125173.628537201952932.1740519978406520.64972483903116.2159339077476520.678051655240318.5852993964807

61.71388459408292.286100202125173.628537201952932.1740519978406520.64972483903116.2159339077476520.678051655240318.5852993964807

36.029045543183600013.12914366431584.5291549402506115.422127123698845.6662071863914

36.029045543183600013.12914366431584.5291549402506115.422127123698845.6662071863914

36.029045543183600013.12914366431584.5291549402506115.422127123698845.6662071863914

61.19834376273542.6497979615541714.01934828027272.5199239065880252.63545921607175.1215659678703174.943043484832336.5971656763047

59.35123577683712.6497979615541714.01934828027272.5199239065880238.46343903317945.1215659678703154.134118084938530.2487508905653

59.35123577683712.6497979615541714.01934828027272.5199239065880238.46343903317945.1215659678703154.134118084938530.2487508905653

1.847107985898300014.1720201828923020.80892539989386.34841478573947

1.847107985898300014.1720201828923020.80892539989386.34841478573947

143.8864553269441.281220992399821.612683200867974.6164678901461899.53680998912612.0285533368519125.938821831226192.877497804566

65.85142776551721.2812209923998203.7113523645145759.99948237404833.0128516472161278.417531354964281.4989058534249

36.6253113371806003.7113523645145723.53452905188023.0128516472161227.211898307104440.3427378028083

29.22611642833661.281220992399820036.4649533221681051.205633047859941.1561680506166

5.7224129759202201.61268320086797010.97636857302443.1375666391488219.340060077548324.0382241778109

5.7224129759202201.61268320086797010.97636857302443.1375666391488219.340060077548324.0382241778109

1011.75144327778193.589551330566236.338318430843131.048016061941568.034437979174232.44575254928649.069316182807901.468288191346

87.17755122604700035.201347641671912.538559218389517.751778095803177.3495910959004

87.17755122604700035.201347641671912.538559218389517.751778095803177.3495910959004

123.5705249138576.6336321382356320.53505575427230.9558332059471865.895212655636912.2236671824612107.85562189093572.8100628813938

97.13178064183545.025478892602757.772614561196550.9558332059471860.07396554524911.3959987414443107.85562189093570.4800988020748

26.43874427202171.6081532456328812.762441193075805.821247110387910.82766844101684502.32996407931901

70.5339898974595000.80931862693337978.328584878224712.298635669247575.83702599203117.082586483682

70.5339898974595000.80931862693337978.328584878224712.298635669247575.83702599203117.082586483682

45.60047840186430004.373396853314423.7503726233575915.411610374296336.5695143386866

02.933109693292684.655481693071678.368049199235697.041443990242093.0191678980488800

02.933109693292684.655481693071678.368049199235697.041443990242093.0191678980488800

46.0944688614211000.62290253870715218.54957382123364.0453457510373916.668344300465858.7022985025105

46.0944688614211000.62290253870715218.54957382123364.0453457510373916.668344300465858.7022985025105

299.962036871066171.707617353267167.762903222194116.719716415851236.488640809315151.088639130022257.490936049016370.056766496159

10.64473173636227.159103264549875.41097652922805012.190380222708512.229658543356438.974042040773712.5338847813012

112.235613081284001.4035019226566264.61418480292917.3137894406179972.3592838905585122.626939899112

19.358801344070531.029361913220249.98110388927624.522427353770727.281036405327330.90182559157860.8229812798956725.02152458646727

40.18129111352683.0283405274904812.81768985624930.80345399920197734.26938787825833.6480191450238929.780889612166960.5706241634563

1.5606580843418800016.46455285953671.28354998874273.5163745595542410.7278025256346

13.8150561785530000000

16.103423258877124.18858946917599.55313294744185.332148942546936.299873996597388.218410749200312.485355239936321.270395802321

46.601686510488000.88524272965966427.93387211697845.2699846843188328.218730372630452.7218319065868

46.601686510488000.88524272965966427.93387211697845.2699846843188328.218730372630452.7218319065868

384.68550135925820.631225087247835.508502039555123.2617161281574169.52919791984453.6443215818236333.337085671543347.26596564796

46.774411239120600021.18360946181653.5470996851913950.823758090406424.0232793156966

46.774411239120600021.18360946181653.5470996851913950.823758090406424.0232793156966

01.448336774017197.6627493705838406.95397263632604000

01.448336774017197.6627493705838406.95397263632604000

327.39422302925719.182888313230627.845752668971223.2617161281574141.39161582170150.0972218966322282.513327581136323.242686332264

250.4437799901485.405247487537462.4429755419089097.278747639813441.4607948981332152.294117137491258.000033193747

76.950443039108813.777640825693225.402777127062323.261716128157444.1128681818888.63642699849897130.21921044364565.2426531385173

34.96036677476261.967782452462187.287694971010942.807003845313258.881983097526552.02551770375437.7058051871481530.0960288812173

34.96036677476261.967782452462187.287694971010942.807003845313258.881983097526552.02551770375437.7058051871481530.0960288812173

34.96036677476261.967782452462187.287694971010942.807003845313258.881983097526552.02551770375437.7058051871481530.0960288812173

34.956758272534611.748272023876722.420866849314214.963110916312165.11741629737832.280063672323451.587054531553317.858527455736

34.956758272534611.748272023876722.420866849314214.963110916312165.11741629737832.280063672323451.587054531553317.858527455736

34.956758272534611.748272023876722.420866849314214.963110916312165.11741629737832.280063672323451.587054531553317.858527455736

22.403099062010600013.94830065602360.45718828170454421.91873475455487.64228218016636

22.403099062010600013.94830065602360.45718828170454421.91873475455487.64228218016636

4.053375857943493.7762302933889513.98529318323563.5911466199149222.902985518463910.192645632603320.092173525008510.2162452755696

4.0533758579434900021.76979766983185.33386328655320.092173525008510.2162452755696

03.7762302933889513.98529318323563.591146619914921.133187848632084.8587823460503100

13582.2469201088688.4315387588591761.58697742577612.697828016625285.845456201871653.821123687354256.358726890789831.2883700239

6155.76750321251208.469539721758736.366021813096178.2301028089172387.51643625857649.8834887606382082.789886282644720.31928819691

6155.76750321251208.469539721758736.366021813096178.2301028089172387.51643625857649.8834887606382082.789886282644720.31928819691

6155.76750321251208.469539721758736.366021813096178.2301028089172387.51643625857649.8834887606382082.789886282644720.31928819691

008.760018807081641.312149726507371.656197624923811.4202594549993200

008.760018807081641.312149726507371.656197624923811.4202594549993200

6155.76750321251203.945722390569722.383981212727167.1404705524392385.36659242798642.5367145428022082.789886282644720.31928819691

6155.76750321251203.945722390569722.383981212727167.1404705524392385.36659242798642.5367145428022082.789886282644720.31928819691

6346.17093025133467.462276469524923.362784408563411.563356910582536.08475966648921.5145363717471860.681012008614446.08306351839

6346.17093025133467.462276469524923.362784408563411.563356910582536.08475966648921.5145363717471860.681012008614446.08306351839

3738.55991295793311.609992091115579.798973073821283.0560064017791430.04644995992554.817024148233945.826079068872269.2194876965

1996.54753968357237.025384699219395.190075805091216.223742341656875.985253682723380.032806511106621.7407928234061440.91679866845

89.3268122254364191.341086886112202.711487807475168.116138359835139.423956970069186.93329197387740.607022375862696.7158874778116

183.6005008750882.7284114701029738.91999282618815.3813872200382772.793217531369421.199263086534392.7565925045678116.450357635545

270.15453325972611.06745465016934.7373580832649.8542313898542119.12029258836949.028509006674786.8668649930218213.259916314172

1332.6270628576457.9799633494747146.05433094102956.011798402454442.756795397122142.510985534436273.010449454266625.638092409432

621.68080648062720.409476719941272.65035775319433.3974283750305208.27207682716981.8293653952736137.843630207908356.329204413922

687.22074639819928.296193764093962.188494563616418.8344841675415214.10058465747956.5903045500453133.319734856929256.341104230909

946.39043802907785.5737377364365156.21664391979453.0756220715726324.946817045594107.893708677048228.432647466127608.900000256199

946.39043802907785.5737377364365156.21664391979453.0756220715726324.946817045594107.893708677048228.432647466127608.900000256199

946.39043802907782.593773255711156.21664391979450.2417140360487323.158335265325104.826311259861228.432647466127608.900000256199

693.52561033921943.111813634338553.393099626261253.1225248633983422.687022655434129.041086946679384.381741613198773.909087190999

693.52561033921943.111813634338553.393099626261253.1225248633983422.687022655434129.041086946679384.381741613198773.909087190999

693.52561033921943.111813634338553.393099626261253.1225248633983422.687022655434129.041086946679384.381741613198773.909087190999

35076.06833811071523.763316014164970.76585624931263.4773425849315320.084761694028.1903919084115145.772475225927248.0792360513

6236.89109600854267.700539619297899.368238174854158.3717947119412580.97888178926773.4532936076652101.885751851434929.81479570789

6236.89109600854267.700539619297899.368238174854158.3717947119412580.97888178926773.4532936076652101.885751851434929.81479570789

6236.89109600854267.700539619297899.368238174854158.3717947119412580.97888178926773.4532936076652101.885751851434929.81479570789

6236.89109600854267.700539619297899.368238174854158.3717947119412580.97888178926773.4532936076652101.885751851434929.81479570789

6236.89109600854267.700539619297899.368238174854158.3717947119412580.97888178926773.4532936076652101.885751851434929.81479570789

10219.3174522041542.0940577876541435.2677630233473.1853745523674775.650144472361234.419543778786936.766843774957883.37798444309

10219.3174522041542.0940577876541435.2677630233473.1853745523674775.650144472361234.419543778786936.766843774957883.37798444309

10219.3174522041542.0940577876541435.2677630233473.1853745523674775.650144472361234.419543778786936.766843774957883.37798444309

104.06396337691921.011960785137264.894719540861416.381672096472999.193989416638135.3893700569995139.28149087233993.0020490971232

104.06396337691921.011960785137264.894719540861416.381672096472999.193989416638135.3893700569995139.28149087233993.0020490971232

5953.00488811961374.687876159911839.559091194258304.3161459554162906.61953322109794.5481357502635600.784424733254843.29724781134

05.6188486895606614.863887333301200000

91.880131222181500061.0090126752268.108913780232632.94949779744578.987623550165

45.77140459637600023.60438787584163.0242485595201519.009677973726917.7296547043157

05.1437254547816310.88561160585881.630538998380486.174207322326241.7648812345212100

1.612392606474760006.18557786988117012.71528811728321.84723671481922

23.459947915989400016.90695470956513.8359975690963318.015398428401214.1474026663443

110.39598341103334.826079760223271.634777578100826.652349781311663.794330781114541.688475951662717.795649611783582.2543353991301

5.55891546232259.517641657827255.035521014955080.90511552563161519.80226493582371.9593783501623321.91873475455489.55285272520796

9.5166215795194900000021.8054246988442

87.42712410840183.0679816773481611.2053407627232026.36395483746938.2003460588125235.71242128112847.2701505540463

01.1103915267465116.44936864885331.055968113236891.999267132943741.7144560563920400

20.12710770840906.2308214578989703.217211478300271.2122416560347701.92155083553034

290.34643354117284.730634745757585.914056223081873.7138766875294631.539039104427167.7474042803883653.442510418751511.54959833094

19.56489799588373.601269816475183.81066455185791.7123807241679244.86070471870830.92673300345515526.588517805804414.9430098494873

01.340127704694078.508294128717211.2744442745962412.9849308634494.9489453174203115.2527277931181.72345281124886

48.6129807279120002.510290570512311.0763401250891776.66642649575228.99595996544248

7.083569460483780002.717450471962371.1651643101693504.86917250556717

26.26251435114421.8360804772973812.628452309159017.5593933494011.598991713432212.54212129885319.0072895191546

2.280023920093210004.37339685331442012.84300864524692.61210816704905

03.761003558334971.326561987810754.768888253327871.504824723721096.4522539756689600

03.331174580239539.399639227916151.055968113236891.33284475529582000

111.49060786792900031.44914591147458.0906915020747636.941687788575360.1078373720951

00009.38680300223584015.03571743833799.17423356231862

41.14374912812693.1725472192757416.7850700498503021.530569124009504.598335962494019.35244322747632

76.3281853865052001.7057946444595823.6836260364104032.878102131832100.304953614684

33.375669298385714.82661518372514.278737509817346.409055022536018.446663901666433.413203469787990.8744176098891539.78151143405602

182.05757240409318.400814085999352.397712182378224.6091175152766117.29245682858645.94081429333384.0122178273834129.79209834347

92.53913743571678.7485697891454210.914304596984314.698355335176298.874217400406229.844933432930866.0916081875374108.583746448677

4.229609590897569.6522442968538441.48340758539399.9107621801003815.35925146520616.095880860402317.471455239137911.3065165105118

107.88562115161411.30846047650647.946496674779212.564716222122346.8935192280258.842858679350993.5352797991217373.5735545224218

107.88562115161411.30846047650647.946496674779212.564716222122346.8935192280258.842858679350993.5352797991217373.5735545224218

846.75364643921220.243602752325494.102271078075423.2554705278574404.76679117890872.4682865641949304.84186034033708.782330249029

846.75364643921220.243602752325494.102271078075423.2554705278574404.76679117890872.4682865641949304.84186034033708.782330249029

375.873063940572007.12236294706596157.7750142694125.5733704016126157.258240831614364.490747177644

375.873063940572007.12236294706596157.7750142694125.5733704016126157.258240831614364.490747177644

336.14170623484630.864611009031256.170565132412234.2861690680933231.60432127827944.7463786645597122.201524799236288.990811769012

336.14170623484630.864611009031256.170565132412234.2861690680933231.60432127827944.7463786645597122.201524799236288.990811769012

4512.74007390385182.45704843447679.378839428884159.8977383401012002.37688610852553.9400984431631486.436797916783584.18121453086

4512.74007390385182.45704843447679.378839428884159.8977383401012002.37688610852553.9400984431631486.436797916783584.18121453086

2872.27159931352126.335690498363405.688956151408106.0350020887011141.41677274563349.744606519556829.204793456081891.30377671128

1275.5897801374333.7053704588467141.1294133778529.3625877943239564.2049177583167.502119477266428.866497413011867.594381918117

1275.5897801374333.7053704588467141.1294133778529.3625877943239564.2049177583167.502119477266428.866497413011867.594381918117

8.032377846933880005.1357320846261109.0490189353666412.2697190965974

8.032377846933880005.1357320846261109.0490189353666412.2697190965974

1413.658603386987.989877838188257.73656619064276.6724142943772518.83318429863162.97520803806354.387198240249840.859302163766

1404.6092061226587.989877838188257.73656619064276.6724142943772516.663437022567162.97520803806354.387198240249835.675583630707

9.049397264245940002.16974727606297005.1837185330586

16.8567922319010001.1331878486320803.0171537718820410.5584161699667

16.8567922319010001.1331878486320803.0171537718820410.5584161699667

1486.4712056416939.0606056549351224.14559403165428.2008025010748752.513584995633173.885317600171571.1023798176941518.87756488807

821.29340803274518.05404949561877.604767090619417.0486548295073425.53971188176493.6263252592557385.331799636056924.826828799778

821.29340803274518.05404949561877.604767090619417.0486548295073425.53971188176493.6263252592557385.331799636056924.826828799778

552.37652096059615.2011896709328107.3030577473368.85180219667386221.49301673568355.131060840394163.160183466488443.342783018832

552.37652096059615.2011896709328107.3030577473368.85180219667386221.49301673568355.131060840394163.160183466488443.342783018832

3835.22871022183.6316292119776402.12496971623474.04268515713151400.94349143859358.894472568161151.209629595092587.1170121091

3835.22871022183.6316292119776402.12496971623474.04268515713151400.94349143859358.894472568161151.209629595092587.1170121091

3835.22871022183.6316292119776402.12496971623474.04268515713151400.94349143859358.894472568161151.209629595092587.1170121091

1382.522160135327.87757820983158.41063759153132.8221364860697491.872380758757119.943478260366394.09202051008923.068571922988

337.67350371511703.274390484055933.3280858441117382.914262356903326.656207607820777.4190846417933178.733372127558

85.40744743324030.84588471807775512.98634367014730.80442552761697349.54906436645358.7070319671663253.465843419055472.2795421002753

726.98541986936519.560353565454585.466859514291823.0623205894766261.91382735971666.6300981051737216.019252914936527.84664257582

893.97404180615114.321026929250871.683940177664912.6532866287406291.49339507405569.6263107240216305.367401785858535.928126779272

893.97404180615114.321026929250871.683940177664912.6532866287406291.49339507405569.6263107240216305.367401785858535.928126779272

935.79033668421228.1174971988755139.48100397647121.6182163614436296.971063178434105.317306062832214.062443270829599.55834324864

149.4785576412436.8991759612027921.91148339815322.7719162972468137.562968454451516.196328210637640.0413802248073118.474687747656

298.6509992334536.5007538720817627.657963963395310.506527847622269.376627176703919.185113796885531.7630695537271136.974385143069

557.21730774894229.155497215220666.21890908078520.1769343358344190.78642648452647.4468530629282176.489165059057367.295491854799

557.21730774894229.155497215220666.21890908078520.1769343358344190.78642648452647.4468530629282176.489165059057367.295491854799

557.21730774894229.155497215220666.21890908078520.1769343358344190.78642648452647.4468530629282176.489165059057367.295491854799

557.21730774894229.155497215220666.21890908078520.1769343358344190.78642648452647.4468530629282176.489165059057367.295491854799

557.21730774894229.155497215220666.21890908078520.1769343358344190.78642648452647.4468530629282176.489165059057367.295491854799

2544.44684649595158.370229234269385.132758017842141.6241654646341019.4615288948296.619639623922682.411870442211836.03029205762

2544.44684649595158.370229234269385.132758017842141.6241654646341019.4615288948296.619639623922682.411870442211836.03029205762

44.15921139177149.0130585154619221.59200061665579.759927628041316.57092499679713.735779733772521.2793035848961916.5441564485218

44.15921139177149.0130585154619221.59200061665579.759927628041316.57092499679713.735779733772521.2793035848961916.5441564485218

44.15921139177149.0130585154619221.59200061665579.759927628041316.57092499679713.735779733772521.2793035848961916.5441564485218

2134.95347343021142.053775450476311.994872298398120.775495568746890.09664567526271.947998557075605.9311257032041684.19702321822

1941.7926184239131.959307025508305.29796430556115.975640508579833.175457305042247.461322850118534.6174586332941522.16323010658

567.36604932876232.9220935902496131.03176210026625.745340935306286.76284645230585.7213295999005222.240217496979546.137997792446

84.738569103472513.45282042019814.7450101871692029.66950160743652.307921614373894.215141298952834.96693909501554

221.15472572479933.221524971827849.216617475326237.7243366015517148.20632362713260.812055943603696.9615005444726262.236956010414

328.99296069205625.62908583887258.850358980109824.4647768643549110.28159462885745.168671330038348.0292037743502152.037099698559

366.99879913386213.41122233602937.0955068847094415.200749948518297.591192136232615.646464443375368.5325142151422183.598019254967

124.37204822562210.09446842496836.696907992838274.7998550601676650.965924569960619.379785326214564.3183261907963154.919966614147

124.37204822562210.09446842496836.696907992838274.7998550601676650.965924569960619.379785326214564.3183261907963154.919966614147

3483.53636162591136.984407763624418.884914528579123.4038192981751478.31163253537367.8109343632141218.573926879082896.24563738785

3483.53636162591136.984407763624418.884914528579123.4038192981751478.31163253537367.8109343632141218.573926879082896.24563738785

2905.0260789691176.1725372266227255.26505346265967.31797285673471146.96851496864290.150309030575935.6560634521222369.8678963158

1653.1162538169646.7775940752576182.11100335547238.9677577224095716.636191508588195.60171869266497.6420267215081290.56914181117

440.48403679052714.481968751615264.647812611033110.5208354451341175.38522900884957.4074411988481115.263612862331376.182472894867

176.101355432273.6152282266165516.10045538592082.5391599669436552.609119740794312.821318544712640.571988008844577.2270991114177

620.1523989824222.440107785091749.33068922255819.1128864586916305.96481184221584.3860576895393230.975645205078536.84941457792

1236.8469574477923.869298113053163.798958932483927.2992516093027428.52653379158494.5485903379148438.0140367306141079.29875450464

542.3726416163845.2467552235782317.672931282871712.2778458692388202.12381295447943.2780066124174176.362559018939520.5679820975

97.26253281350524.8529274156764323.54439753594914.6141745132631726.467554564589610.977921124756846.315042436014969.2752860556095

44.330591661559300014.17202018289233.0382765556314612.4853552399363101.574636571831

44.330591661559300014.17202018289233.0382765556314612.4853552399363101.574636571831

44.330591661559300014.17202018289233.0382765556314612.4853552399363101.574636571831

312.12049261041528.577583736246393.890654557652538.6031187010887168.79370771442441.118936927324161.73190748266222.297542018376

312.12049261041528.577583736246393.890654557652538.6031187010887168.79370771442441.118936927324161.73190748266222.297542018376

312.12049261041528.577583736246393.890654557652538.6031187010887168.79370771442441.118936927324161.73190748266222.297542018376

8588.044549154211246.812776584492221.026229747391175.960573861645157.029117027692043.287056363884795.950208699118703.08660677608

8588.044549154211246.812776584492221.026229747391175.960573861645157.029117027692043.287056363884795.950208699118703.08660677608

8588.044549154211246.812776584492221.026229747391175.960573861645157.029117027692043.287056363884795.950208699118703.08660677608

04.2982897809542422.741062648184305.159399052758024.4244027261729800

04.2982897809542422.741062648184305.159399052758024.4244027261729800

04.2982897809542422.741062648184305.159399052758024.4244027261729800

30.783223083558136.199335195805117.270516338573927.753826464961460.507931252941123.223183437683569.098524111402985.6948731230987

30.783223083558136.199335195805117.270516338573927.753826464961460.507931252941123.223183437683569.098524111402985.6948731230987

10.61247497352483.62459410285132.55689668116894.5959233943988847.56298451940285.409429087670531.268684544959219.6401307777003

4.3343028976029421.25521582029529.9225414101661810.10671893902335.8880017094499414.058148286042113.021030547260321.5175643067803

94.8893669521936001.769308274838434.866382926909712.208187655738824.757189333733867.9538202440097

34.347455201633500014.02295962600697.10129727499662.896749086505038.37552332748666

34.347455201633500014.02295962600697.10129727499662.896749086505038.37552332748666

108.19873215616536.769815236059970.070376984838836.897203407550565.377037931396539.851101388749452.385622566327660.0777129593318

108.19873215616536.769815236059970.070376984838836.897203407550565.377037931396539.851101388749452.385622566327660.0777129593318

108.19873215616536.769815236059970.070376984838836.897203407550565.377037931396539.851101388749452.385622566327660.0777129593318

3327.14607954218311.452206399147548.944642805413312.3101958436562121.28089219786697.9067431784342178.026321613063474.76543063457

30.004311287656310.469405823614.028416811964083.6204621025264710.50246645736274.2725333468817524.424946674164823.6002202553855

30.004311287656310.469405823614.028416811964083.6204621025264710.50246645736274.2725333468817524.424946674164823.6002202553855

2.8895352650686315.602616334032431.928339111750712.177904620666489.244364122670419.56096886394253.255257636815063.31039450873542

2.889535265068631.5649813464212613.24781367692886.3251781279457739.501957683894610.70668875440493.255257636815063.31039450873542

014.037634987611118.68052543482195.852726492720582.462440456998155.6107145974445600

666.307098781815131.04497907845229.469891669117138.882509177271553.246965134426206.41162217307525.465416807351707.197320079079

666.307098781815131.04497907845229.469891669117138.882509177271553.246965134426206.41162217307525.465416807351707.197320079079

290.73764239349358.197238132744671.688092223696566.3624810436211174.989898991011101.31526116564686.8647656557534237.388965864988

01.1601105503321803.30975080268278010.747336472905300

05.7509328674715624.661993318845231.28561909209312.118894761192624.165348832589400

61.783226025492624.388991437977618.458645844474320.220801467462146.507975285284433.086905569778924.687735341403379.4784082885508

44.054060193033400042.18139456338199.231686457495598.4302825979056848.2304491601898

19.98925080629672.263905054531727.1866173708582401.278070313297362.3303286203386907.63355811375066

40.982217184994600027.3942134811865030.779499868098266.8699690764557

390.51610156950828.238476963973347.784133067311126.7009080115259223.65415819143997.1915740611262172.006501297164499.668857497194

390.51610156950828.238476963973347.784133067311126.7009080115259223.65415819143997.1915740611262172.006501297164499.668857497194

340.4704431878621.757720383272333.584702115490422.7227916719271316.86799077171484.5221741590004258.348288474316244.905239432383

340.4704431878621.757720383272333.584702115490422.7227916719271316.86799077171484.5221741590004258.348288474316244.905239432383

1592.1222484204546.1417696830643130.46106780608241.843139216118747.366403145434184.6326094087671101.30788861691753.84878306372

1551.4076333391146.1417696830643125.66998955884438.2548980546334741.692807227621177.6416235477511097.127464581681750.41955388032

5.9865243440396204.791078247238813.5882411614845706.990985861016081.064016250221113.429229183408

83.316233202297119.738334318551311.07483017149634.519358791355444.587381124050134.875728696545422.938210789650370.3922949750696

83.316233202297119.738334318551311.07483017149634.519358791355444.587381124050134.875728696545422.938210789650370.3922949750696

930.757650635551277.125313031003394.492906759616208.659599814729575.317466036208291.167261205427617.137391180446912.20968628793

220.18879099424782.6155434845639102.3275036781863.913490312709133.06467614196184.5635740779476251.429493748942292.917829230512

220.18879099424782.6155434845639102.3275036781863.913490312709133.06467614196184.5635740779476251.429493748942292.917829230512

115.134780089785145.25727501299166.396951575939111.369261533856145.191973760411107.09909699078989.077088241111654.8485790022067

018.580645553548713.432155158286514.88484895380311.585820955309487.1244466776318400

115.134780089785125.611870461191139.44476739119796.4844125800526142.32808249180497.782651702245389.077088241111654.8485790022067

586.68673982538644.6350248182663124.54696373454326.7901161727261294.77282649940495.9398795243898267.672198255289557.154998046878

586.68673982538644.6350248182663124.54696373454326.7901161727261294.77282649940495.9398795243898267.672198255289557.154998046878

2337.70542193443359.110369961721859.697878517698326.6349891722111571.7217044783600.5777537636751160.8757856762761.05709262009

78.27707169157978.3035857592228118.299407290751312.7225631888672169.23730476934716.362929019146543.146256140042787.8467450063926

78.27707169157978.3035857592228118.299407290751312.7225631888672169.23730476934716.362929019146543.146256140042787.8467450063926

2161.67764223691348.564647465798841.398471226947313.9124259833441317.80332116296572.6752166726591098.761393690672588.20012096863

1877.83936519939347.768803709427837.654733012639311.1091111998141168.7961771039550.183264210349935.4154221356642114.21455521005

48.057150215698001.281811004507214.8735591927431015.203746650558260.699779251589

148.6064358176470.795843756371222.892908801436230.756837214265343104.49853282627317.5259418162092118.817483961128332.10864631015

36.01467570802550003.57315828015476001.42276529949906

36.01467570802550003.57315828015476001.42276529949906

05.781377370663672.039177931676035.498015796192056.939604924267537.9346726576821600

05.781377370663672.039177931676035.498015796192056.939604924267537.9346726576821600

05.781377370663672.039177931676035.498015796192056.939604924267537.9346726576821600

662.05250729273729.733169126539524.544890870558916.8739652912944248.51240294879674.4062907639114259.935249267534621.674233580549

662.05250729273729.733169126539524.544890870558916.8739652912944248.51240294879674.4062907639114259.935249267534621.674233580549

662.05250729273729.733169126539524.544890870558916.8739652912944248.51240294879674.4062907639114259.935249267534621.674233580549

404.75964846602851.102844919625182.807860566559566.5604703027588154.61703686577576.6698426952527150.89640130722275.062098648044

35.305451476182210.732481482109812.514924588224615.69079379528058.8237068907259714.75121411782738.8859735491438322.5582602606597

35.305451476182210.732481482109812.514924588224615.69079379528058.8237068907259714.75121411782738.8859735491438322.5582602606597

343.42495093991640.370363437515370.292935978334950.8696765074783139.74149432938161.9186285774254142.010427758076245.274652541281

343.42495093991640.370363437515370.292935978334950.8696765074783139.74149432938161.9186285774254142.010427758076245.274652541281

123.1384612086223.3794524727067910.3579232871344.7809771542619431.816567525260517.842216473341419.750096686966359.1908592247389

123.1384612086223.3794524727067910.3579232871344.7809771542619431.816567525260517.842216473341419.750096686966359.1908592247389

123.1384612086223.3794524727067910.3579232871344.7809771542619431.816567525260517.842216473341419.750096686966359.1908592247389

11259382.348097516309019.243172315316804.076029516452164.099758612692551.568612915778176.254562613012271.478466111514419.3588463

552.441183619383523.655912819577590.31437020629528.906574134939498.613097309224496.407547308892586.047048406762525.100923045196

552.441183619383523.655912819577590.31437020629528.906574134939498.613097309224496.407547308892586.047048406762525.100923045196

552.441183619383523.655912819577590.31437020629528.906574134939498.613097309224496.407547308892586.047048406762525.100923045196

552.441183619383523.655912819577590.31437020629528.906574134939498.613097309224496.407547308892586.047048406762525.100923045196

99.098241313069857.620251170027346.190816976289444.533146279087644.990612252961538.167818163532150.659809002572105.514491435455

030.607086599401220.886797148000522.91061252269888.8044142185302418.552567953227800

01.95131565369685.16193576846866.494866637900521.171118822644871.0042838824053800

99.098241313069825.061848916929320.142084059820315.127667118488335.015079211786418.6109663278988150.659809002572105.514491435455

63.3098705431175001.1732979035965426.65689510591648.8897721442549915.656239110396349.5333104270042

63.3098705431175001.1732979035965426.65689510591648.8897721442549915.656239110396349.5333104270042

324.11044076446449.560830845104515.962949293105466.524807405181379.778232070579414.814246343853376.059581805552289.42077210696

34.334477855521415.17754098689028.760018807081649.8411229488052917.909666473440712.782335094993912.893373385032226.2235172848845

42.34951246401512.571862727390825.534621015529427.2108308499807660.986039833457411.079268314184784.907789151696963.889767136043

08.2639703573925211.393310181256726.28109832560126.91477200901358.1560454984719200

18.59784217174074.797988078534321.015393126472426.3879552529144813.034437680466511.054112094408612.087537548467716.3896983030529

23.37145187116926.010407195773417.183098866126731.735747774230233.433994391588418.577674402623931.918962908673624.8947353575023

16.37523413932280000029.1065492688299.17169151921462

08.7615683661141223.40587060957779.843913272519283.5789263915632716.236904402421800

182.828142367578383.977493133009448.67063668706375.22413898113235.923326759275336.927906536749179.312575010699146.463149324961

8.5836194638803425.004358498004118.297386115346227.69732834823756.3682892055159714.748555882974204.91690949091586

8.5836194638803425.004358498004118.297386115346227.69732834823756.3682892055159714.748555882974204.91690949091586

8.5836194638803425.004358498004118.297386115346227.69732834823756.3682892055159714.748555882974204.91690949091586

8.5836194638803425.004358498004118.297386115346227.69732834823756.3682892055159714.748555882974204.91690949091586

8.5836194638803425.004358498004118.297386115346227.69732834823756.3682892055159714.748555882974204.91690949091586

8.583619463880345.502825265292463.6392408515162114.39106986181240.8256560431036083.5401747477121804.91690949091586

515.3588454530881091.922109857361294.392748911661101.10713031679845.933868082499882.467681746632572.782937072866547.826243601184

515.3588454530881091.922109857361294.392748911661101.10713031679845.933868082499882.467681746632572.782937072866547.826243601184

515.3588454530881091.922109857361294.392748911661101.10713031679845.933868082499882.467681746632572.782937072866547.826243601184

515.3588454530881091.922109857361294.392748911661101.10713031679845.933868082499882.467681746632572.782937072866547.826243601184

515.3588454530881091.922109857361294.392748911661101.10713031679845.933868082499882.467681746632572.782937072866547.826243601184

2.67745928231129000003.0163396451222118.4045786448961

010.841801947545319.157130605974422.63031836169928.197831758514079.8763532606603601.39895332795933

362.487811836757989.0166956631071074.14579844186933.543002162012663.871616571536736.948522518981480.182628400699346.383880349986

76.38080132312275.5519576337325611.74954903489522.6399202830922145.920666959801512.233358319047337.24472507121669.2208664267998

23.834732812589735.799094085500375.900621896214855.084267355192363.889360666245752.24613818148111.503026255037121.5078847906729

6.921179330559631.843337712385522.925777032404737.011962174853626.637882971038491.897421722489211.299529728530913.96462267251714

109.7954707223081.126484157568930022.85223841045999.7169074655030742.856058578451763.607231454851

109.7954707223081.126484157568930022.85223841045999.7169074655030742.856058578451763.607231454851

109.7954707223081.126484157568930022.85223841045999.7169074655030742.856058578451763.607231454851

109.7954707223081.126484157568930022.85223841045999.7169074655030742.856058578451763.607231454851

109.7954707223081.126484157568930022.85223841045999.7169074655030742.856058578451763.607231454851

109.7954707223081.126484157568930022.85223841045999.7169074655030742.856058578451763.607231454851

1198.83298691182597.565686249672827.104359547022609.973387963672081.675713862872337.02487903014791.5414473365611050.7063941388

1198.83298691182597.565686249672827.104359547022609.973387963672081.675713862872337.02487903014791.5414473365611050.7063941388

1198.83298691182597.565686249672827.104359547022609.973387963672081.675713862872337.02487903014791.5414473365611050.7063941388

1198.83298691182597.565686249672827.104359547022609.973387963672081.675713862872337.02487903014791.5414473365611050.7063941388

1198.83298691182597.565686249672827.104359547022609.973387963672081.675713862872337.02487903014791.5414473365611050.7063941388

1.7959573032118827.676886044417415.079687351223624.08514761443595.2367242058965125.187190804981300

017.272757082723513.707807207377719.16386575874351.727761719827921.4816286907091700

011.23769737912132.229583099995171.33586327578162.529193360952926.5066705754637400

1095.597647698712370.895605885122633.265474366592395.824365523121964.256389292165.06672097765709.425528457892938.922957187861

5.996775241889010000011.259624017750720.6106069071268

03.028340527490486.408844928124671.439956518050304.6757892447055600

017.164802350956514.050502387562227.41117227277418.552420513148197.1118177154040100

32.357866123966876.800493459467932.546080227127674.240910600244550.048882728841143.562656958664228.041025272351466.2996495107717

19.08204634662630001.076528456200470.92316864574955902.57192188755598

1079.073476969525937.791874243596777.252827584076571.384038316473417.462460336026026.917280138611226.816320556431172.39113070125

242.941994152934545.714456653437737.469116291764659.658746269244399.08772553285634.750474459167189.147296400477280.671709630864

120.050702178866410.035236292968596.488525365702519.685082263138264.362314216176480.98123672771165.0850965992861143.357360492392

040.784054439684446.169075761829642.060543360188914.045255961095540.370289004129200

040.784054439684446.169075761829642.060543360188914.045255961095540.370289004129200

040.784054439684446.169075761829642.060543360188914.045255961095540.370289004129200

120.050702178866369.251181853284550.319449603872477.624538902949250.31705825508440.61094772358265.0850965992861143.357360492392

120.050702178866369.251181853284550.319449603872477.624538902949250.31705825508440.61094772358265.0850965992861143.357360492392

0201.8983311243262.957565068216233.10308629469383.8177678256004233.31339216572500

92.66366998912522.073017912605236.865597712476128.337417355665991.557979020838839.392607072719856.7313502009349143.357360492392

27.387032189741421.168698289390319.348850001232622.876607929104518.315893740609330.19708921848658.353746398351210

122.891291974067123.869114665952127.559557928043128.950063828173130.040936026095139.049305397914124.06219980119137.314349138471

122.891291974067122.748046297602118.069537553705127.883942175385128.695275455845134.433462169167124.06219980119137.314349138471

122.891291974067122.748046297602118.069537553705127.883942175385128.695275455845134.433462169167124.06219980119137.314349138471

121.363317828664119.104574100465108.431235611017123.552822960937112.272139890192121.307157987415118.898099989908119.809121631546

1.527974145402783.643472197136999.638301942687464.3311192144481616.423135565653313.12630418175155.1640998112825317.5052275069256

011.810105694517313.421032998018411.02360017793324.6844752905794714.719932333541700

011.810105694517313.421032998018411.02360017793324.6844752905794714.719932333541700

011.810105694517313.421032998018411.02360017793324.6844752905794714.719932333541700

011.810105694517313.421032998018411.02360017793324.6844752905794714.719932333541700

818.444024527385290.270809676725941.66329954865807.740064324012947.06167732425287.635774574811037.66902415595891.719421070391

818.444024527385290.270809676725941.66329954865807.740064324012947.06167732425287.635774574811037.66902415595891.719421070391

818.444024527385290.270809676725941.66329954865807.740064324012947.06167732425287.635774574811037.66902415595891.719421070391

818.444024527385290.270809676725941.66329954865807.740064324012947.06167732425287.635774574811037.66902415595891.719421070391

96.79088582710141039.212513218931037.91999286002962.225994026068542.001416816544953.34038887024125.595327645266114.952746175879

74.4581396427394309.645688772505279.37994853269271.38354294495253.202450589906282.13329489398899.213836001097359.1005684985556

4.88259285194772158.668493902068109.812070652158162.7005018288584.300056315758899.61166504184059.5205041966684110.9622900125337

13.573910087718876.74342739256459.3817788851133104.45696252134539.274151616977682.38104916252695.7565825984369315.6726490022943

299.433672754057300.849117802922304.690328238977258.833921053002262.780546984519250.923569903978454.648429319095360.639729970482

64.03756068051591.5187921979654101.548120507909111.96281042656685.9578833506231101.50270640484268.195541310463596.3373868125436

014.385308536304229.74134170470528.01288448020235016.72756803047300

63.546473127759366.347181396189987.859469655499391.412120220174684.129210556630365.79403380502446.440720997553674.5795815379626

66.5645009588422200.310259969429187.123790705182254.502041670382161.839304266783231.82578764759764.678340500301969.4459941601846

25.0746793889761199.205087329189191.611512516687179.05216839661490.6255322355555158.19341048047723.53751705885310.6991950522329

2.334744494175451297.405156875361868.690196809791644.37893389114588.9306189691051471.360730686546.536930353425393.56639835074431

028.557760151355929.734148943176543.967425597788611.131996794259141.082294478192300

5.0755315090770858.568078416272859.976736294924358.30618338036428.315992834849338.078817442621716.20080394901872.9073899598459

0158.972139864157104.375748067004193.65098530229851.3882029426354136.84281729300200

30.2512243121349172.415695204525253.476726991351238.537865497991142.984877698779188.34689015116417.112745101792829.2591069042368

0323.218056348324337.84995728474345.488505270017169.095247992437350.78066219548700

013.37930774030647.078621754629513.63237523236142.294240972230528.853338651860500

246.530713154141469.163509601427572.372345304937387.005365304137403.615013249913464.963880481234240.530815997022238.177615747801

246.530713154141469.163509601427572.372345304937387.005365304137403.615013249913464.963880481234240.530815997022238.177615747801

246.530713154141469.163509601427572.372345304937387.005365304137403.615013249913464.963880481234240.530815997022238.177615747801

246.530713154141469.163509601427572.372345304937387.005365304137403.615013249913464.963880481234240.530815997022238.177615747801

246.530713154141469.163509601427572.372345304937387.005365304137403.615013249913464.963880481234240.530815997022238.177615747801

04.020383114082219.9263431501700900000

23.95064640681514.175210979742724.749050094779310.110332999076620.418047315170115.320671142226621.985357048641620.3252185642723

040.276929015623565.049776020465324.19126950324515.267130833388539.276629655526600

215.9114581691160.18107854660154.761832528111554.1976115908742166.63169868188674.2643267234261187.597811720872192.151624526233

012.07416029083599.5821564944776512.200019949047510.86980188784946.9909858610160800

013.13702651362072.316812485753984.164381291638425.256289175814515.6343626266405100

019.850047062080924.99377309631418.87714039499694.2102876298274238.985281886250900

09.71592585903212.337026486648.623739591434573.887463869612817.3340620190103700

05.747449099709067.819242139419690.7808214921822040.9855542204652233.380617575984300

019.29403716687217.499328349843919.396861087117217.865791400773819.292696993915100

2.08459329837094160.58369397541203.014968350814159.37673051124893.0694943829926167.6302687515714.497331430731482.38821318130199

050.915749930566845.285184548734928.754446891506815.903745913016834.359620659283600

012.7771079790019.0133526843031812.15086596053414.0587734462716.5759958327365700

2.571304509003790003.699084563155823.1721213378178717.380494519030211.7832544628116

21.793518855314253.505121776505364.710519857753538.232011107127935.557259068997722.762223627916221.298151339744.8207615765199

21.793518855314253.505121776505364.710519857753538.232011107127935.557259068997722.762223627916221.298151339744.8207615765199

21.793518855314253.505121776505364.710519857753538.232011107127935.557259068997722.762223627916221.298151339744.8207615765199

21.793518855314253.505121776505364.710519857753538.232011107127935.557259068997722.762223627916221.298151339744.8207615765199

21.793518855314253.505121776505364.710519857753538.232011107127935.557259068997722.762223627916221.298151339744.8207615765199

09.011873260551417.151899412544912.142543997871942.704322691904572.3190709941534800

011.155198758168314.941488467670412.18622462025696.092321283568617.9658406065286300

4.5246986321229727.241782552118441.004448776670321.00450649187824.017666008786468.068009797661055.734552697412591.29592963326465

210.141551989264940.072157372112958.256562091319960.765532451101570.815621472104855.288797915287149.984732461586233.441711255498

210.141551989264940.072157372112958.256562091319960.765532451101570.815621472104855.288797915287149.984732461586233.441711255498

210.141551989264940.072157372112958.256562091319960.765532451101570.815621472104855.288797915287149.984732461586233.441711255498

210.141551989264940.072157372112958.256562091319960.765532451101570.815621472104855.288797915287149.984732461586233.441711255498

210.141551989264940.072157372112958.256562091319960.765532451101570.815621472104855.288797915287149.984732461586233.441711255498

130.412588484614924.246531425529941.534802989131922.671332697613515.78695860969825.420704205954105.003812473934158.607232246227

06.0030955105197810.80040708856622.51332064808424.9393658578609916.245481679382300

66.94751554383589.822530436063745.9213520136217415.580879105403646.003203594156913.622612029950336.581404844483467.5129495483447

010.32541198303034.552408297653134.0913893686306.1998779899047800

010.32541198303034.552408297653134.0913893686306.1998779899047800

010.32541198303034.552408297653134.0913893686306.1998779899047800

010.32541198303034.552408297653134.0913893686306.1998779899047800

010.32541198303034.552408297653134.0913893686306.1998779899047800

010.32541198303034.552408297653134.0913893686306.1998779899047800

818.7960984089532585.627668510743003.987633463162538.099599612271745.331626565482557.18708344785764.061885862017752.518360596093

818.7960984089532585.627668510743003.987633463162538.099599612271745.331626565482557.18708344785764.061885862017752.518360596093

818.7960984089532585.627668510743003.987633463162538.099599612271745.331626565482557.18708344785764.061885862017752.518360596093

818.7960984089532585.627668510743003.987633463162538.099599612271745.331626565482557.18708344785764.061885862017752.518360596093

818.7960984089532585.627668510743003.987633463162538.099599612271745.331626565482557.18708344785764.061885862017752.518360596093

03.843662977199486.7785859816703217.05794644459584.613693383716312.6376247021415900

04.1149803638253113.062733927030513.044311987043913.17164228762937.0595249380848500

3.1895416587096242.941238682053971.552600474515278.492905011298937.209279268855232.50088077121013.5932352056647212.9643848950623

01.3879894084331411.74954903489525.279840566184411.666055944119785.714853521306781.957029888799530

024.84728580342624.269560301586827.264750464722836.707855555688229.51112883953500

63.101202545282437.243664357193941.141658172741841.236608448620944.888361494125743.847827525784553.31584129486327.1094469228874

045.24431146295533.144250262614737.234696530181320.887865568068929.555175300489500

28.47283950562411.47652987375596.623906838464426.9452936306855519.960221279368916.670669650297532.124485960201139.6179886719483

00001.23302818771861013.03537088927271.47290680785145

05.259749337220335.5655758586345710.83756747795751.052245859444078.1211076355412100

5.4045011439246713.400306801857832.76726659555314.555148901898312.239634284214428.79751644434129.132806147731150

13.85420132954592.605387939852166.892193567955294.955381090050183.029104024291023.9385000634423512.28036398325473.50104550138513

23.513422038496800011.93875159956922.2751075629846934.280485635085715.8459642361269

54.926548639635712.873296997663516.408090433925215.581160616895637.928380238531219.575345185906642.902416834511141.5315366284135

010.571201022679217.629495008784210.98030223381225.687469147268053.6271209408998203.31112302865373

5.060862342865862.695748215222752.8524916732115510.254488036057611.325328267542603.800936662639560

16.763831224497429.043049321878639.400710917237425.469216214781434.482293823839626.063344090986718.02715782685317.85678487843474

044.578953941440943.542446423435144.568065955733334.301151790701243.533737118189900

015.868672255416911.59321407892156.66759041543389010.138255943639700

08.531056851832969.027092551199972.704308582679823.413382909903938.7813602888372700

011.815229908520710.917148551226912.65704390587093.403007885861695.5130754843842800

12.812622224133613.781692935503524.487863661421214.268255476622415.800235893928424.615130479775514.434288740804413.0477988441865

011.968401269056820.372793462081916.781928618886316.70061515954457.599303406007511.816469731040450

17.13827605176891986.861503354562189.455468126141835.697124714671005.344689627421863.8992587547219.71882112359481.83038236523145

16.67674638696750006.664223776479134.5718828170454414.09061519935667.96071060433997

02.263905054531724.791078247238818.6117787875629504.6606572406773800

338.163434514105539.41277299147505.25686086775535.796521192389398.003191590435448.803240091171219.65697301554233.950411821155

120.975431178972348.59591713242309.823701591283360.160714039212234.748858016658289.33336555118692.4118496473994131.583974599515

120.975431178972348.59591713242309.823701591283360.160714039212234.748858016658289.33336555118692.4118496473994131.583974599515

120.975431178972348.59591713242309.823701591283360.160714039212234.748858016658289.33336555118692.4118496473994131.583974599515

120.975431178972348.59591713242309.823701591283360.160714039212234.748858016658289.33336555118692.4118496473994131.583974599515

31.372946083201245.375396704045556.907391031771658.057512225741532.553630007735357.979454399762212.279579781190827.0029834344431

12.16012757383050004.6649566435353805.4796836886386827.8624871151898

14.839477717216914.110262511148515.935403701571524.432625056819113.191222758967722.071521529825512.538259287563112.7506296967818

017.937093893597516.113263760595512.34924247811884.7098119958770915.193817294628100

3.8654710168467731.200676109015826.979604498264229.82151549839313.543640741080717.810054938785206.64271215991283

0102.64387522215673.3449346792563101.74679573256761.586265636649773.969169340463502.22899896921519

55.100735120936920.179838420141829.14489527387528.0095227420757552.12507076063357.1572653714629959.04160706460255.0961632239722

3.6366736669399661.173318715797541.800869841072553.696127512038428.484026964376362.37239793302883.072719825404880

217.188003335133190.81685585905195.433159276467175.635807153177163.254333573777159.469874539985127.245123368141102.36643722164

217.188003335133190.81685585905195.433159276467175.635807153177163.254333573777159.469874539985127.245123368141102.36643722164

217.188003335133190.81685585905195.433159276467175.635807153177163.254333573777159.469874539985127.245123368141102.36643722164

20.975511432714629.205370530309941.175210469914535.574912208958143.688633948378128.180207970622919.032001622544710.2617085412587

20.975511432714629.205370530309941.175210469914535.574912208958143.688633948378128.180207970622919.032001622544710.2617085412587

2.677459282311294.0377873699873210.68140821354114.799855060167661.211677050268933.117192829803717.9282871547740

04.0377873699873210.68140821354114.799855060167661.211677050268933.11719282980372.846588929162940

7.2960765442982849.416038891317348.820036915003241.066442478291537.976486431118346.298766811072800

7.2960765442982849.416038891317348.820036915003241.066442478291537.976486431118346.298766811072800

61.842934518337850.732185766159152.287785023592541.827880413809538.659248660345637.645745442015622.962484028581241.1303381224233

61.842934518337850.732185766159152.287785023592541.827880413809538.659248660345637.645745442015622.962484028581241.1303381224233

124.39602155747157.425473301276442.46871865441652.366716991949841.718287483665844.227961486469867.322350562240650.9743905579578

28.582567905498300010.09939067157153.0930908233876927.96333428738315.5110753012397

66.495887492338730.076284712015716.886243103870317.21808816819717.3708254658771216.579802005677400

29.317566159633623.465553200147221.936063781095519.387134325684920.439943473943413.744705346164239.359016274857625.1756277064941

35.503501408065495.5774709613771104.37933981838198.322266443958477.589859667912106.97771232038125.51467758429038.86453619448554

35.503501408065495.5774709613771104.37933981838198.322266443958477.589859667912106.97771232038125.51467758429038.86453619448554

35.503501408065495.5774709613771104.37933981838198.322266443958477.589859667912106.97771232038125.51467758429038.86453619448554

35.503501408065495.5774709613771104.37933981838198.322266443958477.589859667912106.97771232038125.51467758429038.86453619448554

35.503501408065495.5774709613771104.37933981838198.322266443958477.589859667912106.97771232038125.51467758429038.86453619448554

35.503501408065495.5774709613771104.37933981838198.322266443958477.589859667912106.97771232038125.51467758429038.86453619448554

4.5130370377102726.555299156704416.252217288710722.866080520142919.302679694352333.34543779985511.6947475325686715.5110753012397

4.5130370377102726.555299156704416.252217288710722.866080520142919.302679694352333.34543779985511.6947475325686715.5110753012397

4.5130370377102726.555299156704416.252217288710722.866080520142919.302679694352333.34543779985511.6947475325686715.5110753012397

4.5130370377102726.555299156704416.252217288710722.866080520142919.302679694352333.34543779985511.6947475325686715.5110753012397

4.5130370377102726.555299156704416.252217288710722.866080520142919.302679694352333.34543779985511.6947475325686715.5110753012397

4.513037037710270001.442770095938787.423417976130471.6947475325686715.5110753012397

010.061524630538911.046720880845815.202066671130210.773899506967414.781672453075900

044.204348533280443.913857827536734.409383598202717.741880189337127.32763445980378.9342668836500335.0391883418299

044.204348533280443.913857827536734.409383598202717.741880189337127.32763445980378.9342668836500335.0391883418299

044.204348533280443.913857827536734.409383598202717.741880189337127.32763445980378.9342668836500335.0391883418299

044.204348533280443.913857827536734.409383598202717.741880189337127.32763445980378.9342668836500335.0391883418299

044.204348533280443.913857827536734.409383598202717.741880189337127.32763445980378.9342668836500335.0391883418299

000000030.3954404892982

044.204348533280443.913857827536734.409383598202717.741880189337127.327634459803704.64374785253166

148.420905262962104.30946595606467.703892886823698.022576741826974.189299193967299.454314269104225.552909239978268.6403237821841

148.420905262962104.30946595606467.703892886823698.022576741826974.189299193967299.454314269104225.552909239978268.6403237821841

148.420905262962104.30946595606467.703892886823698.022576741826974.189299193967299.454314269104225.552909239978268.6403237821841

148.420905262962104.30946595606467.703892886823698.022576741826974.189299193967299.454314269104225.552909239978268.6403237821841

039.253678395364315.508121318716537.399073307507913.270526838203626.02345768314727.095993265863060

039.253678395364315.508121318716537.399073307507913.270526838203626.02345768314727.095993265863060

77.300165807031336.997054378358419.854707962800343.169673584971923.998161819091129.17586187902417.705805187148155.2242163340981

77.300165807031315.33535520011893.916516344965068.158086985944675.854335470309786.349837245896437.705805187148155.2242163340981

021.661699178239415.938191617835235.011586599027218.143826348781322.826024633127700

71.120739455931128.058733182341825.61177643077615.437890724076729.287045119978339.890924745207610.75111078696763.416107448086

71.120739455931121.396384021862718.56204700983888.0461139314185125.288510854090835.319041928162210.75111078696763.416107448086

06.662349160479087.049729420937137.391776792658213.998534265887474.5718828170454400

92.8212723101787317.814584664498379.265238755174342.652901741455146.440088945773306.002250206394145.733005562583131.541088889903

92.8212723101787317.814584664498379.265238755174342.652901741455146.440088945773306.002250206394145.733005562583131.541088889903

92.8212723101787317.814584664498379.265238755174342.652901741455146.440088945773306.002250206394145.733005562583131.541088889903

92.8212723101787317.814584664498379.265238755174342.652901741455146.440088945773306.002250206394145.733005562583131.541088889903

92.8212723101787317.814584664498379.265238755174342.652901741455146.440088945773306.002250206394145.733005562583131.541088889903

06.926204572775272.442975541908910.977886325732.771261372397257.1294212246005500

1.2800134288242655.391266925075177.273208742205640.272454032874316.081892647721537.062005410992400

020.482222081202611.670160190064810.488331935528515.129589114168814.596044804418700

07.0896614132515519.52346370307336.02150274283742013.067477967418700

05.903347357386527.808244611797444.210505767969853.543005045723081.5191382778157300

07.603768063590251.340981139852176.0259049940148302.6089548684226600

06.615098457213275.249798504953183.14543693304604013.61837434864600

016.910606108964832.585033884024723.46143458552612.675681371573623.55891986668600

01.2144907323797.710641554149981.154965123852840000

07.729797368511645.45282938636026.125781872368671.546394467470299.2826902500784300

17158.335677104719452.920169663717965.919576820419561.523277684317860.958329426218730.959275817313215.477132647816357.5839869349

17158.335677104719452.920169663717965.919576820419561.523277684317860.958329426218730.959275817313215.477132647816357.5839869349

11596.312071230813183.509405691112228.613561478213069.024303270511838.362576943712684.06959331759424.3432597108111322.6457013674

3803.44619531736948.720423474702837.386067253428959.3677360898721830.472454420661071.159887311583317.141249502144507.34478825461

3803.44619531736948.720423474702837.386067253428959.3677360898721830.472454420661071.159887311583317.141249502144507.34478825461

141.32531716905782.71589077739884.7813077355906100.16515808409462.0838751172567103.41042308711981.7547159779036122.189622184972

75.158894531504328.105607148038221.230905545254835.03022337257466.365220395735324.379112540183553.2505564240183102.341312657563

08.900084756365181.883515494143516.771093245183074.273242726902657.3289724548056500

13.254850895701852.190948697363846.227605185268865.617768430292352.370954301103843.90999697483695.9950324113529950.6522140769445

2.8895352650686328.583771692858945.757510018275727.33062270128321.071385727318129.551429013587400

142.84211474453970.572117804819862.270445071526869.9539712619171111.21222288474684.4013561095128197.952259457917256.337099827402

18.8839628205367006.522155993521924.939365857860991.411904987616977.736024031019341.96676379636635

134.605308199749157.815947341997190.256296416289169.796990968466118.914295176793173.61674967724290.8479640766064124.90181579561

91.712756745845360.733227945006265.088379360921368.449049602872351.037660869113660.3165770080721107.707781024016137.211330536464

327.84566803251333.100544127959134.408887018464328.1026220656902160.06201890385750.9474049322324270.416800256206269.905530482152

423.166752327981138.07663746947494.2691638161493125.284902882859197.302107545782125.200715045778347.408361214328577.657658435186

118.47449033308200045.01330644145395.42626568792864125.034346825219115.378812777731

1588.711351612883559.306808790763453.909925095673543.946359036022728.946056827593362.283021804011521.500002231871703.94304646988

328.30112771522213.353211813841217.750634603039176.59729395325229.227680343802206.081814449144269.486579217264316.162950138805

328.30112771522213.353211813841217.750634603039176.59729395325229.227680343802206.081814449144269.486579217264316.162950138805

209.66795628438144.45836942317298.4323534275517139.119931207589216.100382167428128.129837642115158.31349109721229.605707791533

209.66795628438144.45836942317298.4323534275517139.119931207589216.100382167428128.129837642115158.31349109721229.605707791533

1025.877733014893192.389639991463129.767565138223215.428433826772282.667578701383021.958708051591074.391580870371128.60013884431

35.8169640681074117.485837189059144.162929612313116.07436865752235.0341944637202112.1859438398125.775477704843342.7061541257358

85.134403600187545.892256636196342.865742263808145.907912030008765.66053075721736.669214965720370.928759701579153.0682600434582

115.547200157342345.334998023636355.530657386317419.677585446548206.094879478749310.447924670175109.85790172592120.571003538881

23.4607980342635294.321208505733257.749211059992294.796506372886168.600895093017269.69901760367437.501714929375546.1905639418725

761.935833767922284.836027045172259.43267396332303.26863882171769.975638050532230.88697878619798.921567260424858.594180020298

2.5377657545385379.045624625123861.732551533739333.839949357099329.826021525884556.018606810523220.01275781937615.81477991969181

33.503548020831436.624816264109627.707393067492939.812814450489544.049100897282739.25099404183329.9030428107928215.6550113661335

33.503548020831436.624816264109627.707393067492939.812814450489544.049100897282739.25099404183329.9030428107928215.6550113661335

33.503548020831436.624816264109627.707393067492939.812814450489544.049100897282739.25099404183329.9030428107928215.6550113661335

2068.432594297732836.182256760072432.715286387082682.253230499392082.721139436052630.735875205311233.323143046571716.53227516196

165.154766976052311.46691261034311.470452670206310.584963373918242.047292057438309.990956656695138.187508333254115.624468587368

91.9289728099785145.979139718434160.21269487122168.835837923975138.438149874968172.30592685363585.091050251563166.7256782302468

73.2257941660739124.268953324005129.682280759763115.04450734060192.7578862325282117.27545915352153.096458081690848.8987903571212

152.986488264712231.852571008391197.290561246379228.312547004254147.087055855409226.91687842518382.722991562388976.2444671884897

14.03091643134290000000

023.596113931207413.475489435477517.48376913623194.201458415241828.625221959931600

035.722448066234425.040426478497648.878693219940116.635106270724741.453635562260500

106.3629533558283.51369875012354.999293148042851.397786801552379.949694319347856.423461455327956.913289728598174.0154682192745

120.085019486563407.705627299353393.37562973578414.028340400203217.112892755486350.78626262078958.391555289148983.2184285244144

13.39607496658050003.6707855555688204.311882246797676.57737400752023

67.203530945387171.269403603719115.303109753817168.07254818730881.1861236475643147.23772249529136.083570501997142.0287309869014

23.078892244823862.168059752440169.576553554878463.594899215122447.98554591664671.097470834932414.951833824443724.4403044244473

0115.90213072717145.66991338901105.53646448773946.834629387849382.456584862754500

287.02677354711470.858725260784662.774840220373770.3751900264596160.41301778395283.1516041027775135.818837410753243.502852412619

287.02677354711470.858725260784662.774840220373770.3751900264596160.41301778395283.1516041027775135.818837410753243.502852412619

84.9630429348219.2454555760331630.68749664756658.2062480358695350.055756138952122.929165708552264.785067162218798.1088741794093

84.9630429348219.2454555760331630.68749664756658.2062480358695350.055756138952122.929165708552264.785067162218798.1088741794093

837.2156067304551343.816043812041128.422650396781247.96168580877953.8732531585111204.39020697826527.463714280243719.27289587107

486.452204526582429.599040869662412.48753364774446.905414791374443.716523230309443.288546977349343.523036837429321.32143879388

14.34383162985658.197812443558238.706246370207814.6833201176010814.72953702507537.7068096765699710.196187069544921.2244024106306

16.556804918177130.039294388417126.436380689111816.949188164720723.537562480411132.541025376557918.327212584298666.0071761371645

91.697771327252855.904573265943542.214485700823244.757530671737392.119888244650461.303429188444772.3387363706703148.05671723499

32.9226349588839377.215782079833290.344182624568296.048301190978164.78848649984308.12624619557437.414369340067129.7978076691535

048.678631773815844.446763021193140.056903280337414.144605236883923.399984597277400

3385.617810117165014.558351851024748.36874493534947.261240161274508.777707557864876.15935920273064.399659621912893.83720433099

620.202569528407832.499390605306703.421282530824775.36044115255715.308942671599821.10734451604655.379631233271538.238532975648

016.071561089274630.992792969724227.0689450452791.4427700959387818.939876717199800

78.4508270416922181.594091201709178.309274545649154.192968734516122.510954244834132.66428707004829.774811879592951.4421899714272

10.49795186229974.020383114082216.381220596537919.55833205947184.02730069945524.830053230505414.191986531726133.6755239953375

20.74011606501040005.683195910906042.436790333958221.668939194509256.78882934786354

010.59919184621676.280668029562166.854193025919422.03561744445186.1096979464152600

17.350649600065800017.62771371192646.1751987639497633.79861460667746.6843746215483

06.040990171418841.278448340584455.74490424299864.350736765991549.9491750422750100

15.669426135405721.56680351097829.8952375738054518.563797970066714.986380358531629.531470894180344.13168071386857.85385542844279

26.420021205153925.170322024019931.808523039864139.046580612943645.395633881183636.785622982714623.445334697206917.3679577825695

17.873404904035125.556956356973727.682165079792822.566174327274816.553617285220224.95915909186089.7160152359694114.3893089028311

022.538021042071915.210364236856520.184187813133113.088727273228821.644618945517600

24.88584247667630008.678989104251873.7212999673625612.743450438694633.6941704648807

010.9475220946839000000

036.72451513635534.479841467408515.516933393697612.504934005238633.981634169421700

0.8220931317519199.245831875489189.8725933699552111.820016493379328.473830501875912.579104514717216.853044238416615.2548364704991

47.272132812345355.88945222566430.422105334985154.510516841872953.923259551353673.805330477819124.823116075314410.6111111239413

41.16463419934820000.54454746811697031.34293782995664.87864560382703

18.003305758658120.639144365170313.287580131737712.821930416449324.67600247473654.7445399210901216.012062726541626.0532347051125

10.809002287849324.85698798452858.767429990505557.8795590175544208.528766168853788.1180499090943737.1499828202533

1111.066175574531053.010506486641032.854456214181142.549489269551135.966622522211096.33591911227843.420636044226968.328009884127

57.494165546227120.520035414275617.107343394807415.37489572872922.151177377260422.670470296426636.484874218127240.1702771487003

024.037651126780920.178004988526524.335642107018113.926832043882919.657899285332800

17.747213215860738.252296499977119.732859677447840.657114417906236.541733931968734.416966347120417.513633913719620.3320851921656

02.6802554093881302.548888549192483.217211478300275.5177896067789700

8.1067517158869912.678675198814811.328061136436514.8291942355357.5981449000911514.4670337289823.653122459092471.85749914101266

6.5990058933631454.368079984488937.938504684860848.524294128936725.141252139210438.9202577483912.630248170546571.33739938152911

5.338592593388986.136374226757044.8698788763052614.589033143404310.50962632786229.243537146144498.0190493004468630.580778541062

016.840938155655510.280855405533310.47168378959913.1099710956902614.668124038020700

46.473233148674448.294308706666869.731171310573944.728602223673778.825611293298643.164582379334940.041481351635160.5510639877327

020.819841126497118.175083663353513.364596433154313.744961538988221.430700704900400

3.997850161259330003.834210939892111.095999305456115.012832023667615.2671162275013

22.9920413459210009.9993518784851209.652523497155473.76297424197665

019.966673324983220.561710811066617.462224991830211.341408353549316.331897907220800

3.2976617149370769.050311675181346.159027599591765.531134613695154.235177387342338.977512044270920.43271883899173.77796435460201

013.1442951916578.8445853235245117.90885361714988.7176908740375713.519460089086900

190.5184521659572.1511274964646516.05150834481753.7883719335066292.849327547882629.323841281422764.1657160045998267.257730830511

0014.40439269160029.2900280016620609.474625574798100

2.2449466290148600020.09519784907550.61544576383303921.075706494764212.8596094377799

19.91807246514661.70829465653316.3266802495589607.475004710709510.7122369373735479.984828138426653.9685441588401

003.6020515289459717.80500979253432.043046705197983.5039977794873600

59.072789178461858.18824110853246.256300363009470.600152761888578.297315057655672.919297409145636.47746100895321.446419398602

222.82920736110920.7590400299426.244805592468818.3380791368222148.79106674251956.542438539342246.896094315607250.539197940908

10.742689390377811.60635487232787.815310896514016.0204516863279411.03334830606047.602565317937538.068245921921998.20490418116021

026.907266778002228.51658008328423.25735072678918.417214549938530.43227907915427.472295939052765.69914509174338

640.4887197069761500.452911353181598.645407307311519.750222847461324.396893738411475.45991308878842.572003659873582.681472659195

640.4887197069761500.452911353181598.645407307311519.750222847461324.396893738411475.45991308878842.572003659873582.681472659195

417.102936787836668.237968207568584.669536029484585.027568510333536.779705077285605.354843584035313.084634924461378.017379931959

27.554503640034160.326457950786593.222353312969879.790049138652849.718920732274559.397352221261031.7007519306005

04.214136517170506.6793163789080204.337780383642500

16.869541142886251.933747383629219.064333785673427.152046966222315.990986298893630.69310974649793.800936662639565.79797419738055

165.654978683801115.10040581060468.749877224550573.2031457936599146.37545648420190.551534185842195.3620990723031159.415812568032

42.563650655024381.020087596442770.502869180914561.780945927016573.329293848928961.05494310522942.405400472508627.8266481743792

15.5401942949055169.942239042038129.347473975376133.4115428640962.8691142867266133.97398748360633.9024531557932.748607828804

52.8855950889956.39570731018259.700723659432836.456550888832342.643943198889447.30136032650731.030598946407938.7995938043842

2985.193616673594254.07331989973996.753962676544427.60721886723619.458699954724152.421997203632172.832150978372665.77157152165

2002.092521573622905.755709852062665.499620028822881.045638440792467.101717262432807.00717580111481.380507363381907.55641057211

235.263960388271303.403939107013253.828916515862238.814541999179237.983220488107276.14893811964471.8919147210196180.514870581233

235.263960388271303.403939107013253.828916515862238.814541999179237.983220488107276.14893811964471.8919147210196180.514870581233

4.3396737809952717.03016217987636.93091375653937.474830464485813.653478846593236.7422429183956611.00010851994386.21468114093454

4.3396737809952717.03016217987636.93091375653937.474830464485813.653478846593236.7422429183956611.00010851994386.21468114093454

640.282393688437678.051401174415647.663655037186694.453584764728699.067162708978723.473009024676563.322395987015440.002500578273

205.755727944798127.149501865449115.736095782685135.741977891796219.226972618532179.926707523256212.85062948202156.061581403592

65.741765544789563.32423281503363.513954354856957.419405394329781.015373944684270.348615025152244.388553423274245.6519101396135

20.594707622324220.14569439705644.394668724612432.6449313902325.761827426621730.765947747656421.223655887701412.7269625726491

63.9823267383604127.616996451544123.27965082313126.67689398385883.5157310408541123.2038290776558.280166759561292.1218847798514

49.022039029896430.518612150480528.037960922666238.516544584104561.27091228492336.968216636282668.328334853922450.5334871683709

135.998568285754358.966208052135380.361720791758420.04287529144221.365347152795357.82118126334393.3975573033133145.141608358112

54.488345140717795.170851444933888.1394605752738139.96245836411193.253911429253985.210308699315845.358346597932865.4459546022819

71.5448503040432209.087544733316238.828158974475245.301438137787105.206194569714218.15453842153143.227781125112463.3859052005969

71.51363160678253.172435920074837.984523032438661.881951955440721.930949739739158.263714499181538.840297650312745.2458209045636

71.51363160678253.172435920074837.984523032438661.881951955440721.930949739739158.263714499181538.840297650312745.2458209045636

793.8004216182151301.732263297671168.541625182881252.864038850261149.11122902061141.90949797174618.629144442713906.650745912041

497.068913019561073.21005198992922.302518257404976.849834320836922.790856921607906.760047756482391.559772001624555.933064060167

47.25738876521137.2625368087592414.455201487412217.900295672614923.129722843008725.229603775940864.726710059669536.4611972536097

1.612392606474760001.5463944674702903.632939462080918.4723671481922

683.140964628724846.149599567889845.092972918282985.955186647356736.155482345237888.103669113092550.247908698461652.889844916373

504.144269037937690.685369403382684.966663275265808.607279436307607.992445339449702.754604129051488.397274062033513.303719845804

504.144269037937469.786821356732465.280140484362554.913460951273510.080448721856452.485362176506488.397274062033513.303719845804

178.996695590786148.935127987238149.269726334774174.686867565692121.445499439098179.58849263456461.8506346364276139.586125070569

178.996695590786148.935127987238149.269726334774174.686867565692121.445499439098179.58849263456461.8506346364276139.586125070569

268.957123333182495.105681575767475.195123963538554.51317236889398.586335878417443.792926341903103.450952775129101.710723110114

268.957123333182495.105681575767475.195123963538554.51317236889398.586335878417443.792926341903103.450952775129101.710723110114

268.957123333182495.105681575767475.195123963538554.51317236889398.586335878417443.792926341903103.450952775129101.710723110114

1428.577860024941169.807933393161100.341107934521262.159842405581264.149681074671149.06148761748832.2280489317211282.90590366218

1428.577860024941169.807933393161100.341107934521262.159842405581264.149681074671149.06148761748832.2280489317211282.90590366218

132.09232164185548.828184844304743.150214519892651.202824084242493.338427051121250.829793218130958.2262076856852143.591645819946

132.09232164185548.828184844304743.150214519892651.202824084242493.338427051121250.829793218130958.2262076856852143.591645819946

999.23644656948978.075928468175948.5732252069851080.5993874162971.446409611034965.525288538161622.05708853677814.595247094294

8.4259151692683570.464215646959986.566217482013100.422146864478.192267193050156.840127688738118.16316252312232.63267594789197

17.043511820269668.55781271121957.321372897761742.644501025915523.478865447702378.17061414079766.5576714576707616.6118170953029

71.431572840925147.360494507062466.823065401806559.698102580082350.091974383223939.795174285896579.238219079100980.2815400151003

96.619377353731168.365434925034260.3250310610668109.87753876454768.979437634864187.058769297618546.66135078712579.6719516904761

30.97472781284099.749779259237688.5972310011428412.362553520822119.1385650442722.555080672277234.0780177183719321.0334461555845

55.242229448992.3036477183352106.317039746937132.18873434426991.7921193754825109.10601291309327.925346495146649.4911992823037

15.0434567923676146.745643363073134.634082941016154.45161572929175.8431284896299124.0190660512748.4737376628433417.2345281124886

150.098960876976136.33530682387107.19961918619108.700853753137128.017740928285108.93791947121556.5735625567507146.291279333328

150.098960876976136.33530682387107.19961918619108.700853753137128.017740928285108.93791947121556.5735625567507146.291279333328

338.395562994662252.241432402948184.917242207107235.714872394684381.065896822512203.359718540906314.196918942824368.953140326765

338.395562994662252.241432402948184.917242207107235.714872394684381.065896822512203.359718540906314.196918942824368.953140326765

261.520372890044209.554880837578150.683828924262172.22344400716296.505628565901174.377058848453216.050425235817294.471845690488

179.98357170301290.908457434762142.326614127253366.9078538751087168.38067867426667.44189165824117.097262379905175.602675532589

81.5368011870316118.646423402816108.357214797008105.315590132051128.124949891635106.93516719021398.9531628559122118.869170157899

000012.0385977897687005.39273944164966

000012.0385977897687005.39273944164966

76.875190104618342.686551565369634.233413282844963.491428387524472.52167046684328.982659692453298.146493707006869.0885551946274

76.875190104618342.686551565369634.233413282844963.491428387524472.52167046684328.982659692453298.146493707006869.0885551946274

188.418332458914255.741324164292254.966932868613268.525455827435198.476115882102274.129156583429135.675724923518186.423093557097

188.418332458914255.741324164292254.966932868613268.525455827435198.476115882102274.129156583429135.675724923518186.423093557097

188.418332458914255.741324164292254.966932868613268.525455827435198.476115882102274.129156583429135.675724923518186.423093557097

188.418332458914255.741324164292254.966932868613268.525455827435198.476115882102274.129156583429135.675724923518186.423093557097

188.418332458914255.741324164292254.966932868613268.525455827435198.476115882102274.129156583429135.675724923518186.423093557097

14.290911282473633.160681288499718.923988066598828.385582063635936.785428913260231.019976915387712.186268361238220.0089272811643

3.9706539016589216.644393019814919.652935275090325.07548989496996.6314969086184234.32752541617218.946422348797860

8.6030834535943474.759831678421667.652642966680358.22494935506731.609622801888854.316656605781631.51033179196796.82346623229139

19.456204118128894.63346353079470.5379290751041114.81438904013134.18473728830686.321917932172915.34311432818836.68699690764557

100.2633206777336.542954646761478.199437485139542.025045473631972.069767939249764.866303633438151.9799147197541117.528804819099

34.674423180823504.425839098346174.9720471699494615.299076957925110.617336721310685.039410842158631.5170033898678

34.674423180823504.425839098346174.9720471699494615.299076957925110.617336721310685.039410842158631.5170033898678

34.674423180823504.425839098346174.9720471699494615.299076957925110.617336721310685.039410842158631.5170033898678

34.674423180823504.425839098346174.9720471699494615.299076957925110.617336721310685.039410842158631.5170033898678

34.674423180823504.425839098346174.9720471699494615.299076957925110.617336721310685.039410842158631.5170033898678

00001.442770095938783.7117089880652410.1684851954121.72345281124886

34.674423180823500013.85630686198634.7529474830670374.870925646746629.7935505786189

706.6559367423172754.929787207042430.066429976573301.548156842351600.485340586192453.724667674851048.43469802493581.060005671903

362.790801024693911.706825255202826.436258910772882.628937656175637.089160915197733.130362408787305.074194081522331.447440025192

362.790801024693911.706825255202826.436258910772882.628937656175637.089160915197733.130362408787305.074194081522331.447440025192

362.790801024693911.706825255202826.436258910772882.628937656175637.089160915197733.130362408787305.074194081522331.447440025192

362.790801024693911.706825255202826.436258910772882.628937656175637.089160915197733.130362408787305.074194081522331.447440025192

0000005.802018023264490

021.890554172655830.463566756701716.04824672385399.2086297055387413.084674685787200

14.486465666126713.413311252086824.517232298384529.868430523740823.191142366544326.577462601307300

1.0889666484027316.55776512890513.87168779571538.899798145006520.93611170104388913.739696878822900

021.090271611913119.904320614922119.18220408624042.3720118526451114.190335343173200

013.16351245417241.9898429817161214.30666475998364.514474171163271.9356761927006826.51459849341290

1.686954114288620008.089520191101821.387421086097620.90515164451763.86531613158704

03.720992882182463.9373488787148811.79538849892262.9776319001289610.213780761484500

014.020108028278814.549458947404411.740183019849916.504607958013316.767189559848900

094.185062981476189.117313489404493.322483645179232.767273869833383.82478161487780.9963061252070350

04.0377873699873213.908083611381608.002117186151062.0781285532024700

27.4317407822041153.031774281702160.583682552098157.20258419464143.598383346203137.38802449595668.951934841752673.995630411044

1.0889666484027322.983397229666827.081780708725126.107437908599817.96695077794619.09599205185782.453589711330761.24757404993388

016.80221415273629.147586195814456.20611845425079011.108543110725200

011.61444010734862.836098042905744.248147581987475.362019130500451.8392632022596600

04.114980363825317.257074403905866.522155993521926.585821143814675.6476199504678800

8.6198372675253936.298735082528719.064074147336147.420526305485424.78271307258649.554753018420912.485355239936311.2860707302035

014.03411512971292.284634534562962.053271331293943.8874638696128110.000993662286900

0142.913054577641108.004664309522124.73879877059754.80277164880479.5076716455340.8744176098891531.39895332795933

14.467433831429122.049203173966522.044391998803426.952328985474715.544973263230416.8179975055621.07570649476428.85884205713731

21.3543703735568.0226643710462424.917979890551112.241850274729824.423403719168714.809224988647929.785040258802617.4747305948926

301.7531070944991427.599578333691252.042014332931870.22031336296769.1175231601451322.79793521248717.53833581789238.409936696712

276.0709176585691403.849505139881225.443000526271819.71982394203751.9373336017821298.41563460255633.3703943604233.060339170596

276.0709176585691403.849505139881225.443000526271819.71982394203751.9373336017821298.41563460255633.3703943604233.060339170596

276.0709176585691403.849505139881225.443000526271819.71982394203751.9373336017821298.41563460255633.3703943604233.060339170596

03.5736738791841713.23512420022685.097777098384972.144807652200185.5177896067789700

009.490020374338432.8429910740993207.6930720479129900

01.1717699528480804.4573528397938906.0307499471076500

03.363205105049542.37250509358461003.4618824215608500

15.378984872785613.272611814348117.19653945264899.674575406751998.718459908736167.737886059842582.739841844319344.75301250788533

242.731493998546885.721363876487712.8240498777741295.41307508355490.776348699906878.730172571585612.579073869448216.486301793236

6.826738287062733.864898684255819.542451426130362.4503127489474716.09697164969082.652197214308127.690784124405181.95526225369754

01.850652544577522.937387258723799.6797077046714500.95247558688446300

4.51969343452107202.242611210657221.094589092392194.922724772369116.048514350917147.351158413452.909566560339111.47942409461185

087.261419121444183.211317993075192.422258881851549.170620334215981.480463035130700

087.261419121444183.211317993075192.422258881851549.170620334215981.480463035130700

087.261419121444183.211317993075192.422258881851549.170620334215981.480463035130700

087.261419121444183.211317993075192.422258881851549.170620334215981.480463035130700

087.261419121444183.211317993075192.422258881851549.170620334215981.480463035130700

035.818093122700635.283230030468936.999677433449127.426787035943934.80846212113100

051.443325998743547.928087962606255.422581448402321.74383329827246.672000913999800

171.530643722539195.623368351235242.403610612217185.502599346133136.983445015447197.171269052317216.376135286138121.073954472624

171.530643722539195.623368351235242.403610612217185.502599346133136.983445015447197.171269052317216.376135286138121.073954472624

171.530643722539195.623368351235242.403610612217185.502599346133136.983445015447197.171269052317216.376135286138121.073954472624

171.530643722539195.623368351235242.403610612217185.502599346133136.983445015447197.171269052317216.376135286138121.073954472624

171.530643722539195.623368351235242.403610612217185.502599346133136.983445015447197.171269052317216.376135286138121.073954472624

03.569115621685232.517760507477545.656972035197611.428047952102676.1230573442572700

4.73003341607669147.644104013438177.652529674256140.94437541393392.52591293396151.2246654866818.650463121784813.5474005422317

01.274219784791082.696617811287432.423533374642039.176963888922063.9348171786046800

11.80867880002030001.617904038220364.1622632582927924.706088307157117.3939225921417

48.151591797176615.858596541504524.241920462399911.52055763755728.554415545925886.2327672084407375.958012309118233.6041552075198

105.422993183612172.086135396472145.804518426276125.667026224676188.569643473905151.815908549523133.428953728031112.610530836532

105.422993183612172.086135396472145.804518426276125.667026224676188.569643473905151.815908549523133.428953728031112.610530836532

105.422993183612172.086135396472145.804518426276125.667026224676188.569643473905151.815908549523133.428953728031112.610530836532

105.422993183612172.086135396472145.804518426276125.667026224676188.569643473905151.815908549523133.428953728031112.610530836532

105.422993183612172.086135396472145.804518426276125.667026224676188.569643473905151.815908549523133.428953728031112.610530836532

04.484273473399394.74501018716920013.847529686243400

61.396382722018346.962372274414842.845070247357935.473240553508187.577241549233355.167191849279977.066492930604673.2050133450493

05.687371234555314.51354627566.76077145669958010.244920336976800

025.38037775420625.177605074775426.399202830922118.088607393300522.859414085227100

2.334744494175457.461831059736573.947848475724785.32207929071396.717537566690955.7605723494772300

015.545481374451217.545993225443513.797983346295311.19589594448496.400635943863600

85.1366397106886465.545873304559576.497234279656490.125661194689343.715310282806470.17038014090352.8599962983289102.110901228869

85.1366397106886465.545873304559576.497234279656490.125661194689343.715310282806470.17038014090352.8599962983289102.110901228869

85.1366397106886465.545873304559576.497234279656490.125661194689343.715310282806470.17038014090352.8599962983289102.110901228869

85.1366397106886465.545873304559576.497234279656490.125661194689343.715310282806470.17038014090352.8599962983289102.110901228869

85.1366397106886465.545873304559576.497234279656490.125661194689343.715310282806470.17038014090352.8599962983289102.110901228869

85.1366397106886433.296406252539534.508765244637455.539218372295312.371516693955447.35077637281352.859996298328985.857783745008

04.2875733592175614.51414880781177.995040227820597.823388527627496.8865432011913900

027.96189369280327.474320227206826.591402594573423.520405061222715.9330605668981016.2531174838608

019.620510472608322.358365153781214.352964645938211.775618711836926.410391030505200

019.620510472608322.358365153781214.352964645938211.775618711836926.410391030505200

019.620510472608322.358365153781214.352964645938211.775618711836926.410391030505200

019.620510472608322.358365153781214.352964645938211.775618711836926.410391030505200

019.620510472608322.358365153781214.352964645938211.775618711836926.410391030505200

019.620510472608322.358365153781214.352964645938211.775618711836926.410391030505200

418.839808821451446.503628057285502.863827739637427.788526822353473.365068642206403.714009953502485.339902274375403.155365486231

418.839808821451446.503628057285502.863827739637427.788526822353473.365068642206403.714009953502485.339902274375403.155365486231

418.839808821451446.503628057285502.863827739637427.788526822353473.365068642206403.714009953502485.339902274375403.155365486231

418.839808821451446.503628057285502.863827739637427.788526822353473.365068642206403.714009953502485.339902274375403.155365486231

418.839808821451446.503628057285502.863827739637427.788526822353473.365068642206403.714009953502485.339902274375403.155365486231

4.946492572405620002.033153016552951.743514633619029.5529594571909410.5243292735342

384.543635740843237.474866740585305.696954889764243.948876302655299.801361038361231.324943428345439.16055852862359.900181588185

10.514958463705795.3174925408659106.66342128393388.879360873756697.624911466569793.60953094407528.570060365324487.77557779958787

0003.801485207652790000

02.1895044189367910.42565618589294.1643812916384203.380617575984300

046.127439183934323.804072252984235.203931673950619.378989057642919.76624882216600

143.549432580006304.624365396668376.777550178996346.125958719656274.419861836733344.842276147167162.479751408585162.230931847448

143.549432580006304.624365396668376.777550178996346.125958719656274.419861836733344.842276147167162.479751408585162.230931847448

143.549432580006304.624365396668376.777550178996346.125958719656274.419861836733344.842276147167162.479751408585162.230931847448

143.549432580006304.624365396668376.777550178996346.125958719656274.419861836733344.842276147167162.479751408585162.230931847448

143.549432580006304.624365396668376.777550178996346.125958719656274.419861836733344.842276147167162.479751408585162.230931847448

44.5754146303401181.512757480967228.316811904417217.945980045931143.916315197358201.02258092158328.61459126871236.5233099095006

28.70587492838663.129962692842529.935860257696621.4882772065754826.35040973474611.6108983080193646.712057673641356.6384983980908

61.95944375375140.281281327643870017.21926095201242.3162735623149381.302906357365251.221267024788

220.309175619371106.36025911026785.581714331720785.0969552239988151.90593493723577.8997426799243129.532765943181104.180362639452

220.309175619371106.36025911026785.581714331720785.0969552239988151.90593493723577.8997426799243129.532765943181104.180362639452

220.309175619371106.36025911026785.581714331720785.0969552239988151.90593493723577.8997426799243129.532765943181104.180362639452

220.309175619371106.36025911026785.581714331720785.0969552239988151.90593493723577.8997426799243129.532765943181104.180362639452

32.32138143402513.50476181178191.905332275928955.993332534587727.158804341984173.7069320138206303.26726233272584

32.32138143402513.50476181178191.905332275928955.993332534587727.158804341984173.7069320138206303.26726233272584

04.8078808374591212.71858400684536.85834960143543012.372363293550800

04.8078808374591212.71858400684536.85834960143543012.372363293550800

015.810057852804220.10933610247959.113149470400527.668421879784192.4244833120695500

015.810057852804220.10933610247959.113149470400527.668421879784192.4244833120695500

182.5020223475277.9104432026599815.056615907973313.786947741195462.92285422890321.167976201675108.91159024614490.8574658591386

182.5020223475277.9104432026599815.056615907973313.786947741195462.92285422890321.167976201675108.91159024614490.8574658591386

03.284256628405181.158406242876996.246571937457642.62814458790726000

03.284256628405181.158406242876996.246571937457642.62814458790726000

03.284256628405181.158406242876996.246571937457642.62814458790726000

03.284256628405181.158406242876996.246571937457642.62814458790726000

03.284256628405181.158406242876996.246571937457642.62814458790726000

40.666656148547913.37194290426112.844271236112966.3259612288299813.146681250345517.796836129159205.48114500626685

40.666656148547913.37194290426112.844271236112966.3259612288299813.146681250345517.796836129159205.48114500626685

40.666656148547913.37194290426112.844271236112966.3259612288299813.146681250345517.796836129159205.48114500626685

40.666656148547913.37194290426112.844271236112966.3259612288299813.146681250345517.796836129159205.48114500626685

40.666656148547913.37194290426112.844271236112966.3259612288299813.146681250345517.796836129159205.48114500626685

010.75191795351092.844271236112963.834351074001374.839724471679355.533691017749500

40.66665614854790005.162042187518651.4755564419767505.48114500626685

31.1415616159079322.309990897538334.646209822816332.944892317395120.801467700369260.49528788281524.519106619604529.9209500590455

31.1415616159079322.309990897538334.646209822816332.944892317395120.801467700369260.49528788281524.519106619604529.9209500590455

31.1415616159079322.309990897538334.646209822816332.944892317395120.801467700369260.49528788281524.519106619604529.9209500590455

31.1415616159079322.309990897538334.646209822816332.944892317395120.801467700369260.49528788281524.519106619604529.9209500590455

10.7295243298504160.93922067343152.294457408868168.64761239962748.9845179266282126.74810861606404.91690949091586

10.7295243298504160.93922067343152.294457408868168.64761239962748.9845179266282126.74810861606404.91690949091586

20.4120372860575158.705830559916178.121914761386155.42714776657970.2175360673855131.00404957652324.519106619604525.0040405681296

20.4120372860575158.705830559916178.121914761386155.42714776657970.2175360673855131.00404957652324.519106619604525.0040405681296

44.7263041258926128.139719093403145.48487246917786.537955509155885.4098703053651121.03974053913941.435342312003243.2726775721376

44.7263041258926128.139719093403145.48487246917786.537955509155885.4098703053651121.03974053913941.435342312003243.2726775721376

44.7263041258926128.139719093403145.48487246917786.537955509155885.4098703053651121.03974053913941.435342312003243.2726775721376

44.7263041258926128.139719093403145.48487246917786.537955509155885.4098703053651121.03974053913941.435342312003243.2726775721376

44.7263041258926128.139719093403145.48487246917786.537955509155885.4098703053651121.03974053913941.435342312003243.2726775721376

014.56865757160824.439912816352914.679453239332911.904840914172726.264940987960100

35.051948487044194.424759709332278.202754723007161.577158766006345.759309622377273.381772951477834.42772922637830.8673011508161

01.110391526746517.049729420937131.055968113236893.998534265887473.4289121127840700

032.520432300576256.178375399891343.479790501641827.051386513374741.429403630898700

032.520432300576256.178375399891343.479790501641827.051386513374741.429403630898700

032.520432300576256.178375399891343.479790501641827.051386513374741.429403630898700

032.520432300576256.178375399891343.479790501641827.051386513374741.429403630898700

032.520432300576256.178375399891343.479790501641827.051386513374741.429403630898700

032.520432300576256.178375399891343.479790501641827.051386513374741.429403630898700

612.5449058996195831.02151131284995.186492185636124.92408042012921.798367274435439.8333110139762.475477661973670.178626648456

612.5449058996195831.02151131284995.186492185636124.92408042012921.798367274435439.8333110139762.475477661973670.178626648456

612.5449058996195831.02151131284995.186492185636124.92408042012921.798367274435439.8333110139762.475477661973670.178626648456

612.5449058996195831.02151131284995.186492185636124.92408042012921.798367274435439.8333110139762.475477661973670.178626648456

612.5449058996195831.02151131284995.186492185636124.92408042012921.798367274435439.8333110139762.475477661973670.178626648456

04.338273871939884.590521483400917.219875006782431.301848365637787.8147299314613700

5.2426897324299156.747144140794630.301533627340975.207563427501921.846027538523660.419436397373513.78124041454044.00418976505722

011.25707271943025.104976477230334.5879993885464806.6213475281347500

095.650542750534280.686987719343899.905294289407250.978879470258386.410687016268400

4.451454454875468.25794159678178.315710966287186.226430987593841.807365904925569.794892526508140.99343887858373.08369385075783

05.486640485100415.805659523124693.478483196545030000

010.79547317670227.767757417514067.391776792658213.628299611638627.1118177154039900

01.564981346421261.65597670961615.953108826301891.878506030953858.0544915400968200

05.6630948486556928.203500595868410.25154795831945.9839707312939214.652618621490100

024.46973920052515.992441741940730.114646192311317.277617198279222.224430360637500

23.518488494441431.39193397518726.660591034990935.052786195767748.213417240700231.714069035038221.677869537471824.4944941671999

08.832659871847275.6077393121090711.75964489741084.240869675941278.1826311782347100

043.872485443491928.269312403378222.38684967801038.9573189282889934.907234874527400

028.833891801893722.933914211666228.11043302026025.2387213643980119.910456601077400

504.4678394777794384.443311473793678.272917689444549.906480542692112.297106097044022.15368798348579.247203611595565.152240406261

029.129909178670515.72797637607922.23306925220064.7126224816127824.086111479389600

05.4519955513845211.538000340603617.741110796731816.534211388939815.942543402612300

03.9355649049243512.49319137887597.485343587501973.543005045723086.0765531112629100

050.07262799582736.765078338848342.792753125519814.942551325132144.482054430091300

04.2785728553535413.58204750822754.068867959261381.283933021156539.9092414268530600

24.996254482728462.986359680551952.581225598488757.94782180718236.011746647281452.631473067749637.564347458314932.5272474014434

24.996254482728462.986359680551952.581225598488757.94782180718236.011746647281452.631473067749637.564347458314932.5272474014434

24.996254482728462.986359680551952.581225598488757.94782180718236.011746647281452.631473067749637.564347458314932.5272474014434

24.996254482728462.986359680551952.581225598488757.94782180718236.011746647281452.631473067749637.564347458314932.5272474014434

24.996254482728462.986359680551952.581225598488757.94782180718236.011746647281452.631473067749637.564347458314932.5272474014434

24.996254482728417.361999258393118.946070883533816.398205718652424.995084378287119.39427896164823.059302400153732.5272474014434

00000014.50504505816120

07.261730053809388.537734592830425.371187288782770.9685031093845085.813726419599300

00.992264768581996.2997582059438112.26720403887961.191052760051592.0427561522968900

36.9319891909684193.427505248629191.064775802722231.532759114456121.884520970706237.94208353180797.017925008230831.8809319417825

36.9319891909684193.427505248629191.064775802722231.532759114456121.884520970706237.94208353180797.017925008230831.8809319417825

36.9319891909684193.427505248629191.064775802722231.532759114456121.884520970706237.94208353180797.017925008230831.8809319417825

36.9319891909684193.427505248629191.064775802722231.532759114456121.884520970706237.94208353180797.017925008230831.8809319417825

36.9319891909684193.427505248629191.064775802722231.532759114456121.884520970706237.94208353180797.017925008230831.8809319417825

31.26889947556413.919028917928862.0734498296873911.180838846037612.34841464465256.1770843208242376.324165663181823.8821318130199

011.49039411154229.894824988811796.732508324344898.656620575632688.115816288888823.389495065137320

028.990222022625236.677646311632332.36399568677376.8083151013759629.84080271125600

02.0276714836240710.727849118817415.4263167846782.4338904227141120.871638947381300

08.9195384935375612.134780150793418.17650030981524.588481944461037.8696343572093400

27.2616974365598106.608199027696120.818882953539127.65947132522138.053109311146783.85443244719490.63471239636741610.8806623704106

27.2616974365598106.608199027696120.818882953539127.65947132522138.053109311146783.85443244719490.63471239636741610.8806623704106

27.2616974365598106.608199027696120.818882953539127.65947132522138.053109311146783.85443244719490.63471239636741610.8806623704106

27.2616974365598106.608199027696120.818882953539127.65947132522138.053109311146783.85443244719490.63471239636741610.8806623704106

27.2616974365598106.608199027696120.818882953539127.65947132522138.053109311146783.85443244719490.63471239636741610.8806623704106

063.265789278160578.112303313484779.363329198262322.905104991049365.884194953953900

3.4377740266062243.342409749535242.706579640054648.29614212695915.148004320097417.9702374932410.6347123963674160.64546302197351

1541.392420176645778.938951203075518.033803769425855.322393337243622.731119445395503.106649972161489.482594726591717.57834252434

046.473379633411853.489205746271150.398478131760415.658595726552436.087501606573700

046.473379633411853.489205746271150.398478131760415.658595726552436.087501606573700

046.473379633411853.489205746271150.398478131760415.658595726552436.087501606573700

046.473379633411853.489205746271150.398478131760415.658595726552436.087501606573700

046.473379633411853.489205746271150.398478131760415.658595726552436.087501606573700

1541.392420176645688.440983766255445.067429650125782.070192703473589.653124378285451.265035941691489.482594726591717.57834252434

482.9550484239292301.679795466572021.575306278882170.445097182351293.080854433762130.80020211749668.808142207194572.421298476077

482.9550484239292301.679795466572021.575306278882170.445097182351293.080854433762130.80020211749668.808142207194572.421298476077

157.45975483708487.249546671594414.444613432407502.647691238308297.011078720396414.575098454742188.867325420965182.510852716781

027.125826947040537.344084505766933.460351615814115.469002613084331.11343300204200

18.376199415631123.79410414456815.0355210149550815.83952169855331.15947555348857.9442483853412402.21607188322968

0.76000797336440513.966643422358516.706390033991614.43706404816058.746793706628868.750869454501011.712401152699592.61210816704905

039.208545525179717.508774157072116.134941349280313.082505961207214.596540469068600

031.926993576009615.232986469990433.556577592133314.66878944571745.838001957700700

60.751571614741965.220768433315770.238365045091160.675066440697285.810413595654871.3162917607685116.25406278658980.6834253491451

5.1653639251669330.637881469929229.074669996801744.736757446267514.181946146206539.25080738159952.9095665603391111.8353927568948

4.2143402421939511.811952354988217.728552856653627.292555766570427.363705108276910.731254033229918.99096152019184.82815661201848

112.16546445515994.740833577117806.382114192619828.05729084031552.631565621318873.999056268099183.452708740676102.22577262056

015.47180610727379.355091174703299.458671725202442.6530559110153813.650645377908100

48.4514158809733451.031014005357366.473133487517350.406718935951272.170179553588386.54469963170918.528633036650822.9541779157374

09.711085123426255.9658549582239520.101490829423310.524555623204325.232717165351300

028.102176174458423.902775433568439.33114109605312.041818733098628.813053710624500

0207.157419208646165.228033303214126.71617358842683.7193111920191172.5171406744492.93554483319930

03.5153098585442402.228676419896945.626078364062779.6491999153722500

2.6233084204218724.285234569893521.808498745646743.682621081530713.002783395631929.48938058396874.191162056013162.00810717947315

020.150526177053516.583911849039513.327278925596409.7306965362791300

50.305393513054700041.19006603884074.68339215404653129.38298030973172.1809166101978

125.94340473346224.840479638818834.64121786572246.306444176589292.859860752728952.3977437135637269.783715093659237.658133815633

125.94340473346224.840479638818834.64121786572246.306444176589292.859860752728952.3977437135637269.783715093659237.658133815633

19.974193968027297.27034724886222.712633127336255.007631609151144.818529765921241.69189679945620.011520172015625.9066467651641

13.34139710957455.605579468862430.033640830459350.145376821974824.424916750742145.816992995597415.029989545980411.4634232702495

6.6327968584529811.10391526746511.46869362936192.6399202830922114.84304386579441.42871338032674.9815306260351812.6647668705408

031.402451891074522.305527850961327.762218522285714.846996214047835.846991262959200

06.700638523470355.672196085811495.0977770983849712.86884591320111.3794474016947400

12.160127573830518.01925729501946.7771257089469811.48034995656328.7143921925640840.69817321085209.28749570506329

012.40040860545876.777125708946987.472760129218362.2352857432093920.789903363702700

05.6188486895606604.0075898273448018.797048329117500

20.622981828803431.643413456670128.750169730430117.01409818889218.9154270392738446.82624357958071.660510208678395.06590674821635

20.622981828803431.643413456670128.750169730430117.01409818889218.9154270392738446.82624357958071.660510208678395.06590674821635

154.621108950958859.832538194982886.11464608454941.423360589361457.627641825611836.662489983217170.378041176054114.249137769171

154.621108950958859.832538194982886.11464608454941.423360589361457.627641825611836.662489983217170.378041176054114.249137769171

9.74345701358027719.086077684293721.632998566757800.525125675702369.953331791797717.7479079935887.11397531507480

9.74345701358027719.086077684293721.632998566757800.525125675702369.953331791797717.7479079935887.11397531507480

144.877651937378122.030807547964149.79671080705129.29264935327178.1928752531183105.839277234617163.26406586098114.249137769171

020.788805062642441.728047484841428.76509197729980.96850310938450819.367753476374100

144.877651937378101.242002485321108.068663322209100.52755737597177.224372143733786.471523758243163.26406586098114.249137769171

903.8162628017562419.521432437332438.662610622172569.488450592991782.907602052378.5147687234650.2964113433391030.90790627909

903.8162628017562419.521432437332438.662610622172569.488450592991782.907602052378.5147687234650.2964113433391030.90790627909

900.4998643725292402.783842410782422.210698780882549.463912411031771.979429034722365.92724720115647.8056460303211015.71018603444

06.758904945413567.1518994125449124.103619976059310.141210094642213.914425964920900

58.27578524968463.4175522751048316.13410453720670.50000744933426513.4252845763923.3517404718474826.093731850660345.9952168250754

11.813034978370214.920926844157922.300712980542216.481928106444710.565237582998218.54377792607255.2721199125539126.3481623022822

00002.813039182031382.4122999788430619.825991235275711.7610498375676

032.781444935646537.459562531947950.891100194021114.380376053223834.666764748342800

78.21840835462062.091320364275945.532298872932734.9720471699494630.22912309413964.0682008117777250.920013106672259.2043575311503

07.94939388466253020.15939125270423.180652256955955.4550874521564800

04.3996645399399.310963386143376.2760368994267604.528751847073300

047.214874518887738.896798136210554.357111658456630.328858331844345.88247243844501.43497787717716

14.167138920967526.548016597845110.211786576432219.38010631045571.5506781086544220.54367042595713.192048750663318.11528750927862

15.182587028597612.491904675898303.959880424638323.235808076440735.673561227077691.900468331319785.79797419738055

016.543347301627218.425906251193412.048901597941211.793530180468712.690430363098600

07.9845763872967316.983886917317125.16225905047964.352003577302412.789530679234700

0.95062886570661574.085889511486289.660450851170565.595259587115221.367484052055269.979261773827300

018.00180202452685.0380642073868735.24693565849792.5445218055647510.91017490431300

2.9184306177193221.929081855945116.66909766350873.983592283468491.67603232701877.1863427513438519.72686127909930

1.8354909545404511.87791472310615.953524846099735.350615908894666.7535565348769813.537689837766114.47463615866822.10282921624075

032.563827755537.8583748545152126.3906531882349.8316901361233218.032043698993900

13.387296411556512.95456781204275.8747745174476114.07957484315852.2214079254930417.1445605639204015.3371488707467

13.80338805678050003.782397278542214.8653482681395513.328960323715727.1094469228874

04.185321908506093.796008149735389.0975714371177803.077228819165200

019.746926972069213.27965757110028.541104093923220.247456467845214.044747542805300

4.3886174702546286.654500838201334.899313538305306.513860076765212.136731852097288.2752476529510.010423852257.54172583569051

49.558255772592282.233243770548122.346662269025108.33304905485693.3669557691504113.42095884848259.462739187303363.0848764872224

050.453296160973930.890730301715634.44029568852622.846861555330320.888177190331400

1.2633898778005727.279195292648417.937131464146819.86853982917139.3141642247149729.28831451168174.269883393744440

29.603598187871200030.9943310742897.671133550232325.703563715967444.0482582964997

015.48318750054233.415209531311376.13870771444407013.855839758842900

190.0617539974338.822073635092914.712579648886731.652902662647128.90659142286957.834201980060377.412314081445229.978667385072

1.118172650467170001.072403826100092.7588948033894811.33727659718358.9672372324749

021.041047775259219.003300602535527.139542466050813.495304437859546.570786434502400

8.737816220716512.05211477345098.317096507847185.9798643715886602.6968971673582600

02.809424344780348.918332399980699.3510429304712105.7837071781899900

017.831581576576414.514148807811716.95760558315714.81809757358324.002384789488500

2.993262172019817.488666546257613.1080906420554.8508535201819410.11488195305035.250521672700620.23267823497366.85845836681597

19.182173074211412.07479169651285.70027626445415.432249853201617.902608678021715.898944045470738.589321750976843.952092350722

15.95943829964830002.37201185264511015.788919102857214.8089074465768

021.198383692433427.70882954218616.60185161987414.967775326851528.238099752339400

09.099793975288496.018061700799988.6537874645754404.6833921540465300

04.4842734733993933.215071310184610.66121652787240000

50.669066828321966.906194457406761.727362070287480.290962189993970.209408114690970.762900562175414.004871090638448.1518032903483

11.297150778268324.815722561050525.805890265632328.48207571482989.501104356558325.76402770981804.31419155331972

21.504225604247617.149824955118718.361296550399616.53009360583428.29104252104887.6203549599417607.03894411331114

10.06355385420450000000

012.09907748483239.3109633861433716.736098398471415.84324897804476.7931277706099600

0132.279493877095161.712438878074158.60470761770584.0099711219841127.02084797699100

45.764593234158914.18849037692999.975740782799717.271365845091421.574646118034622.12127364086732.962621674587412.0331563890794

07.127840804879367.542286935063740012.577668885321500

036.497606018802516.37232076483728.64152669377822.534307795546514.509270793616900

04.558631126623265.6024559700023113.70549908418257.647469907435056.7615536574873301.82704833542229

04.090916151171364.328781223382443.890408838241162.455240338702846.3164170498653900

17.000566705161183.6774137463458130.126913676608126.98233433392195.110766518682693.96004501237429.5761462519899616.2305750185572

019.142289210003216.875304522654223.31262179562214.2410113688806618.881929497778300

05.050878424911946.235320245955214.80332065948907020.796290070314900

13.4489890217480000006.16313079045675

010.680908971561710.04306691316047.064929519513465.172707026505229.4703286924512600

010.680908971561710.04306691316047.064929519513465.172707026505229.4703286924512600

23.1931247604911133.808343861388102.909847833266118.69693064071753.03853325873786.056817486167228.873290193329136.4259519882358

23.1931247604911133.808343861388102.909847833266118.69693064071753.03853325873786.056817486167228.873290193329136.4259519882358

23.1931247604911133.808343861388102.909847833266118.69693064071753.03853325873786.056817486167228.873290193329136.4259519882358

23.1931247604911133.808343861388102.909847833266118.69693064071753.03853325873786.056817486167228.873290193329136.4259519882358

23.1931247604911133.808343861388102.909847833266118.69693064071753.03853325873786.056817486167228.873290193329136.4259519882358

013.789786374075811.909627018321811.13829379715621.2780703132973610.280950006615300

015.37465190879795.422868785336264.8736989841702405.2752494042831800

044.804834257306826.754384382488743.599562557522910.443502195175114.363255728292700

15.09533078130680002.8954903304702509.069821277746815.764652506591

017.172334076428611.476303708502311.17361608192527.5941154662203913.024549885768900

05.979031297865851.581670062389745.685982148198615.382642281002376.1544576383303900

216.630867383348200.106593612354218.462353090087248.279983980661207.184961637222231.768340111964120.656524385178217.212608138907

216.630867383348200.106593612354218.462353090087248.279983980661207.184961637222231.768340111964120.656524385178217.212608138907

216.630867383348200.106593612354218.462353090087248.279983980661207.184961637222231.768340111964120.656524385178217.212608138907

216.630867383348200.106593612354218.462353090087248.279983980661207.184961637222231.768340111964120.656524385178217.212608138907

216.630867383348200.106593612354218.462353090087248.279983980661207.184961637222231.768340111964120.656524385178217.212608138907

216.630867383348200.106593612354218.462353090087248.279983980661207.184961637222231.768340111964120.656524385178217.212608138907

5538.235994435926452.561447556736535.620758635556237.192651097316091.786143353646199.158591519055651.666116805955563.15163765684

2276.558697632422739.559865639512722.548827541152709.29671703622714.532702422152802.417196278772482.188482989342147.20157610983

2276.558697632422739.559865639512722.548827541152709.29671703622714.532702422152802.417196278772482.188482989342147.20157610983

2276.558697632422739.559865639512722.548827541152709.29671703622714.532702422152802.417196278772482.188482989342147.20157610983

2276.558697632422739.559865639512722.548827541152709.29671703622714.532702422152802.417196278772482.188482989342147.20157610983

08.04076622816445.6721960858114909.65163443490079000

85.179710769300980.011228013557653.383716371595663.287763230749218.945728137899646.80795714042529.2909783386479920.9486718540171

34.2898276914342.832172720041723.43243698973936.2845065848510925.96250368198539.4689758278840815.512761845821524.9910284514543

14.010232033655456.149092892760950.861822822600863.84863456436821.317124812387662.55734333126812.961991183047953.01216076920973

2095.212907541912531.824638875392541.086659527352511.417584035912589.853779873742614.836258136242427.120807113242052.95557293078

13.34139710957439.819364075871955.734683955305145.763881211974327.572266449734460.36722179056213.75749738649519.55285272520796

34.524622486541220.882602833721612.377311788758318.694347408352421.2296650315038.3794400523902223.544447122085435.7412893791618

3228.785165618543623.729026468783731.64550861663451.601278472363309.823594442453339.024078506933142.361673295513369.73945515615

1562.508558005722429.627747056712697.697618713842321.626680097392029.024145964422236.100246526611746.831559218881459.63670433289

119.217854827396121.949234437177104.411381414016114.26716027386493.077695440065992.391147056383742.1303752917069119.106315917965

77.557854380035986.130456077708165.8205370196871.570334204972376.278441122010759.595834871564229.7701865203414105.640392575954

77.557854380035986.130456077708165.8205370196871.570334204972376.278441122010759.595834871564229.7701865203414105.640392575954

41.6600004473635.818778359468738.590844394335942.696826068891716.799254318055232.795312184819512.360188771365513.4659233420104

41.6600004473635.818778359468738.590844394335942.696826068891716.799254318055232.795312184819512.360188771365513.4659233420104

892.8828698664421129.485279384381202.083746030551064.764614261521078.042725577111112.772096650051115.55898945511862.477703569868

892.8828698664421129.485279384381202.083746030551064.764614261521078.042725577111112.772096650051115.55898945511862.477703569868

035.922656438949728.797491414274450.049527187532717.102679851903763.880389745663300

3.9616705217909218.48618860462623.530085891420915.141731780502211.717105924710717.82374276780145.950787716168729.07737136784467

026.809796128659334.786887302621117.269178885000825.594538976110950.091726799363900

15.36016114589110000008.79868014163891

107.94981324141518.721612188416334.046391491078633.592706850238758.611777843658918.4313579931161133.36569952307569.5246322278594

25.852918721739134.843176877817330.665523243982524.306280557744627.987968163865742.461805149400321.095506682858357.6098141046061

684.16915161238947.2916193319471024.66834135195880.782633553148896.62150748703881.763166539982934.272010052484690.931503713453

55.58915462322537.046575564328621.019756266098936.230778654694438.333833266041833.875021582600420.874985480528426.5357020144666

536.5105446560731138.824580247211369.097730923781116.80896378251835.1066754000371008.34780430323589.142194472059471.229218612763

130.566438971013155.78701722277173.330189116399139.089156167126116.904373238156119.16313787042277.148212892981977.3900881345923

025.317192453726429.839076023906724.418210018809111.108102502070722.069178359075200

52.270217593039685.146411210103468.254483003456348.308696698047941.677791533274443.363173849329636.309267160678213.5286064605546

405.944105685059983.0375630244451195.76754180738977.719807615381718.202302161882889.184666432808511.993981579077393.83913047817

405.944105685059983.0375630244451195.76754180738977.719807615381718.202302161882889.184666432808511.993981579077393.83913047817

1601.255018981061011.37486489778867.837247197469950.9338112626071205.90015686597959.0696382883391354.022952426771824.25908388184

1601.255018981061011.37486489778867.837247197469950.9338112626071205.90015686597959.0696382883391354.022952426771824.25908388184

1601.255018981061011.37486489778867.837247197469950.9338112626071205.90015686597959.0696382883391354.022952426771824.25908388184

012.65205729994824.6602778623983415.76337015192562.787439752661954.286140140980091.467772416599650

02.767741491000227.321677440142429.870325094053984.152780394838634.2734216242412700

1065.01304606146348.413466454219298.775928731139290.089081423315759.991026524591345.545800904405990.4437913585041282.18841761411

03.666387116615856.983222539607528.368049199235691.760360997560529.8122956686588100

013.108185270179520.76950191862636.908606150039796.0931380658596317.231690464560700

09.640608604310813.825434569500783.438035717515430.7232490920209893.1010833061354600

024.181859915812915.840132772969826.829412062240810.366570318967518.96484724107744.059024954547170.825555173783405

35.27773274166225.877179473834434.77344796142519.034521095110227.544993535698734.7486153421521.677869537471880.8318307517598

04.151612236500329.518180672185137.896260075243170.8305560789677244.9856585616148200

52.90747941529521.571923493181131.521917895379631.260377625338517.497035504690636.878125720664729.801933635024343.0789473138374

16.82303363597666.960484612019549.750285992335244.976618686886646.039124944077348.854084305641818.128276864669428.8867579979584

136.15291735144184.617477281122652.123034098850978.897625515186975.022289240461862.36820354260371.2253807887293112.42205631239

85.831294989940300035.714586066294716.1354370513982111.362128628489119.524435934041

033.13923280165231.342483173892656.441395074010318.676692496884922.57048394055381.596024375331660

9.859562897700390003.782397278542210011.2956028845364

12.230533606102410.668467609917512.043101967772115.943047984164716.06962148408043.1375666391488228.191590192996514.7032810417627

015.19848402234292.9205670244210613.52000514814363.1273452358896514.738096406554400

494.4536529268091616.465144571071659.671121189551573.410099078521189.696109304341382.27989542389460.364805554514524.098324549284

102.534750617817145.04625551589192.804347538634161.499052278738162.248224595656127.651522146076153.409734537229151.117318533893

102.534750617817145.04625551589192.804347538634161.499052278738162.248224595656127.651522146076153.409734537229151.117318533893

102.534750617817145.04625551589192.804347538634161.499052278738162.248224595656127.651522146076153.409734537229151.117318533893

102.534750617817145.04625551589192.804347538634161.499052278738162.248224595656127.651522146076153.409734537229151.117318533893

13.519080890543400024.42137719306883.7317338883915115.125307569086415.6480739287797

89.0156697272733145.04625551589192.804347538634161.499052278738137.826847402587123.919788257684138.284426968142135.469244605113

3.2070666128783749.24177378272945.505243460295458.73476446122852.505548680076537.339782438790.9903042810792824.28501750814755

3.2070666128783749.24177378272945.505243460295458.73476446122852.505548680076537.339782438790.9903042810792824.28501750814755

3.2070666128783749.24177378272945.505243460295458.73476446122852.505548680076537.339782438790.9903042810792824.28501750814755

3.2070666128783749.24177378272945.505243460295458.73476446122852.505548680076537.339782438790.9903042810792824.28501750814755

3.2070666128783749.24177378272945.505243460295458.73476446122852.505548680076537.339782438790.9903042810792824.28501750814755

365.4376951340821031.621855006951084.476153012231024.43793096507837.236133970741970.210181174943283.151389746771337.369762249203

365.4376951340821031.621855006951084.476153012231024.43793096507837.236133970741970.210181174943283.151389746771337.369762249203

365.4376951340821031.621855006951084.476153012231024.43793096507837.236133970741970.210181174943283.151389746771337.369762249203

365.4376951340821031.621855006951084.476153012231024.43793096507837.236133970741970.210181174943283.151389746771337.369762249203

91.413427526658142.878923522000936.723035759018837.074956548341655.040321813764353.322081863312674.3566494944103126.356994001425

17.10435526868460002.0505303927628108.4302825979056813.4719717919599

231.275127713448945.2367895760721012.42791376964955.684559430046745.97050993769895.819820437979175.422449140651179.861825696581

5.0317769271022737.130065563887227.614561929415125.614848086458615.366211191088316.611953756485724.942008513803811.529305013182

20.61300769818920003.501671648834470.70595249380848506.14966574605456

78.903555117429946.760894506226629.236044914550554.6254487497023106.01239309260386.152465311283662.4952797861434109.97578955825

78.903555117429946.760894506226629.236044914550554.6254487497023106.01239309260386.152465311283662.4952797861434109.97578955825

78.903555117429946.760894506226629.236044914550554.6254487497023106.01239309260386.152465311283662.4952797861434109.97578955825

78.903555117429946.760894506226629.236044914550554.6254487497023106.01239309260386.152465311283662.4952797861434109.97578955825

78.903555117429946.760894506226629.236044914550554.6254487497023106.01239309260386.152465311283662.4952797861434109.97578955825

6.1974395356973811.731876414116510.355350417463212.441149679185820.148112313487115.573557698474218.40665949465512.2637974174967

6.141264397277325.448663291576315.820346841486727.223691329720440.123406867824840.763892833428410.200070182092219.9752823193151

65.2074415948183001.2084648707343128.59987043039410.464254949095827.771693898822871.5162475817681

27.5892997834618255.761870538109302.197236017252206.138661248343171.529337728395194.1634835605817.690324273005518.273182096225

27.5892997834618255.761870538109302.197236017252206.138661248343171.529337728395194.1634835605817.690324273005518.273182096225

27.5892997834618255.761870538109302.197236017252206.138661248343171.529337728395194.1634835605817.690324273005518.273182096225

27.5892997834618255.761870538109302.197236017252206.138661248343171.529337728395194.1634835605817.690324273005518.273182096225

27.5892997834618255.761870538109302.197236017252206.138661248343171.529337728395194.1634835605817.690324273005518.273182096225

0171.302646394421179.355281137579118.47357081369276.0982744789121106.1489463570461.178426599707240

31.07277693324795.9610959975022815.744751134949125.687701983977335.484027053973223.246080905837334.970780721900927.7431262611761

31.07277693324795.9610959975022815.744751134949125.687701983977335.484027053973223.246080905837334.970780721900927.7431262611761

31.07277693324795.9610959975022815.744751134949125.687701983977335.484027053973223.246080905837334.970780721900927.7431262611761

31.07277693324795.9610959975022815.744751134949125.687701983977335.484027053973223.246080905837334.970780721900927.7431262611761

31.07277693324795.9610959975022815.744751134949125.687701983977335.484027053973223.246080905837334.970780721900927.7431262611761

01.702060004501959.0051288223649314.56773528480082.0430467051979810.511993338462100

18.908142030294128.444958705028225.136510243702231.645157206790520.502943038748126.512961691320300.941830550372616

18.908142030294128.444958705028225.136510243702231.645157206790520.502943038748126.512961691320300.941830550372616

18.908142030294128.444958705028225.136510243702231.645157206790520.502943038748126.512961691320300.941830550372616

18.908142030294128.444958705028225.136510243702231.645157206790520.502943038748126.512961691320300.941830550372616

18.908142030294128.444958705028225.136510243702231.645157206790520.502943038748126.512961691320300.941830550372616

08.9000847563651811.3010929648615.0783199338873203.6644862274028300

18.90814203029416.949139025003024.902129729790723.67141231423427.649467194604223.8552473283741400.941830550372616

190.120658708471257.848240644921251.340368810751268.043201793827228.861291889253207.724815362143163.692571158096318.240138079782

190.120658708471257.848240644921251.340368810751268.043201793827228.861291889253207.724815362143163.692571158096318.240138079782

190.120658708471257.848240644921251.340368810751268.043201793827228.861291889253207.724815362143163.692571158096318.240138079782

190.120658708471257.848240644921251.340368810751268.043201793827228.861291889253207.724815362143163.692571158096318.240138079782

190.120658708471257.848240644921251.340368810751268.043201793827228.861291889253207.724815362143163.692571158096318.240138079782

38.65317274338424.237162966231925.728206109820217.0480523720543.138208993615524.486871625748540.63585656743364.8508599375922

03.194276994750246.760014513227383.0377164901335016.439989581841400

07.603768063590254.0229434195565201.521181514196327.8268646052679900

09.280884402657434.9102592981651612.13575294317025.570097484818378.3590394789263500

06.477283906021323.426951801844444.6198604954113701.6668322770478100

62.53779895112823.4291503031877610.88561160585884.0763474959512238.711299878077315.841910128916627.398418443193471.6463954390598

030.460562614061121.713419392032247.534884234070211.285144826869926.206703966755700

28.208450390644449.783940268936746.166838106116750.590765571971822.873215324384735.714197636142319.408712868360314.8183932551932

011.515171388482402.7376951083919305.9265147628366700

010.013960394585116.65120139301120.406745746602401.472538944140400

224.585212186922514.316095951539513.030906741316540.846190466914478.526881272893520.895081279728249.249425964607217.496016053125

224.585212186922514.316095951539513.030906741316540.846190466914478.526881272893520.895081279728249.249425964607217.496016053125

224.585212186922514.316095951539513.030906741316540.846190466914478.526881272893520.895081279728249.249425964607217.496016053125

224.585212186922514.316095951539513.030906741316540.846190466914478.526881272893520.895081279728249.249425964607217.496016053125

224.585212186922514.316095951539513.030906741316540.846190466914478.526881272893520.895081279728249.249425964607217.496016053125

010.73865489682486.493171835073667.2945165717021612.89001177818994.7373127873990500

10.3612921339147000007.781799321143710

010.999161349847506.276036899426762.640541496340796.7931277706099600

05.722263082620069.08247348709694.081349149320475.15148586402687.3626947207019900

17.873691069299640.378549581282345.617331491392681.244784868590232.534148447540248.538878058872911.33727659718357.68620334212134

000010.03616170322352.8688109310145611.788960923764526.641421942811

53.790357113418778.268276979854172.469158903659272.530079515673564.257797747602264.8851602883022114.49319225679785.0663955183033

022.485428416616914.09945884187428.71173693420435.997801398831228.5722802819601800

042.071264372315244.043894942707148.521836143744744.052949493654757.587295560193100

12.97080274541920000010.22874288545894.45799793843039

07.101996567008157.51493999186191.125651288222061.420798977726513.6551855009373400

05.381128168079257.592016299470746.8231785778383301.8463372914991100

03.379452472706795.959916177120766.427631993615826.760806729761428.1167484795371700

012.397361223499212.99802116164547.336386932840594.456964946052920.054446639400300

011.17996948162585.0700108849205316.707440695734311.50263281967639.8639937491048700

5.14564569642187109.364500487458108.692238805807107.929506670769113.750152111543133.4257505173327.18463669626021.18563774958255

023.566249105668422.77538993162179.29133954384412.3455089269172318.147812001142400

20140.6218785130397.862939378729536.59020669430486.337864591925985.655131496228449.108623707719797.530252918520159.6222911776

4449.380676870857223.632322120676869.438882528626976.942435899766215.807841563046849.742463766774280.811664787024350.03321029012

4449.380676870857223.632322120676869.438882528626976.942435899766215.807841563046849.742463766774280.811664787024350.03321029012

12.2881289167129512.686605056398362.843730689667373.63147082513273.914255135967301.256053848927.383152759428981.75973602832778

015.02257947481967.0311335657535713.15482082032842.699974906226286.0679537186588400

05.248474419026925.553645363760767.843364442370442.699974906226284.6306851683900700

12.2881289167129497.664025581579355.812597123914360.476650004802271.21428022974295.1881001302627.383152759428981.75973602832778

12.2881289167129484.588387042321342.231356766103346.66024478488268.598416691309291.4494109107157.383152759428981.75973602832778

013.07563853925813.581240357810713.81640521992192.615863538431073.7386892195464900

013.95107302835362.108893416519651.89532738273287010.257429397217300

013.95107302835362.108893416519651.89532738273287010.257429397217300

013.95107302835362.108893416519651.89532738273287010.257429397217300

193.380939882077193.365672117281139.993841060524148.06494460212155.246200852155178.062690102763139.087071132933155.212989688152

026.06768739888094.3287812233824411.23830556872514.6649566435353826.405375480165900

026.06768739888094.3287812233824411.23830556872514.6649566435353826.405375480165900

193.380939882077167.2979847184135.665059837142136.826639033395150.58124420862151.657314622597139.087071132933155.212989688152

027.173994624504814.447584345489219.542310910391311.137710633572717.326418976665900

01.2403309607274926.862107678629114.73132096471292.0430467051979810.838163344531400

156.3442375840088.0062565018632612.70766676735454.758654587548276.481204680059130.171044991021289.2423451071809135.902509234405

018.5310374000084.902129729790724.405694777081041.8536251563716811.126933346120500

14.355363586144721.351878086547813.130316438109113.098994697355419.768054852921712.33215654199419.645721946596.3015564165186

13.701552195865320.492572595787618.669263830434623.534779728001716.971558073930223.888678722965223.454778000837611.2943094455253

8.9797865160594210.762256336158502.274392859279441.435371274933973.692674582998226.744226078324551.71461459170399

12.4497658984229252.5528000468324.890707673972328.400146766739148.704363192164343.80544344847910.779705616994110.9622900125337

12.4497658984229252.5528000468324.890707673972328.400146766739148.704363192164343.80544344847910.779705616994110.9622900125337

6.0706825810036512.572550119296425.825273250936627.763746690941111.077811406444324.998310012514500

063.2638657510199103.702116831159114.03432029490945.1626180102089124.56673939633600

084.098412867362279.86222763129678.397772143188131.823605730610282.408878387199700

6.3790833174192765.169723664046782.015322287660466.40403625601948.276743854903189.37095902154110.779705616994110.9622900125337

06.477283906021322.284634534562964.1065426625878802.2224430360637500

64.828835160208415.95521927885649.23171910092312.03039519827828.612545213766521.102500050476276.238889280187631.7255035231457

64.828835160208415.95521927885649.23171910092312.03039519827828.612545213766521.102500050476276.238889280187631.7255035231457

52.59589245120539.628569882277458.275360458547818.5923594807625726.936512886747818.311525074954370.332643388241725.7192188755599

12.23294270900316.326649396578970.9563586423751923.438035717515431.67603232701872.790974975521935.906245891945896.00628464758584

1041.4279832803554.550429183498463.646243031606479.120209068965755.660050706267645.6446260778091104.526097894081072.91144449364

1041.4279832803534.126795942681449.194297718685458.669226621356751.911424831997633.0875342950831104.526097894081072.91144449364

10.37664219633530007.46393062965661016.07373882000684.45799793843039

1031.05134108397534.126795942681449.194297718685458.669226621356744.447494202341633.0875342950831088.452359074071068.45344655521

020.423633240817214.451945312921120.45098244760943.748625874269512.557091782725900

020.423633240817214.451945312921120.45098244760943.748625874269512.557091782725900

00016.53423756252493.6828605080542614.211938362197200

00016.53423756252493.6828605080542614.211938362197200

00016.53423756252493.6828605080542614.211938362197200

3094.592173699235590.154263866425471.930123865715527.36155203794791.445959717875242.894294419582909.758685393973043.27886354064

16.302049308259816.376360551361510.607006680467514.579642501380911.838902854089511.363346476563516.047580510367424.8765784947233

16.302049308259816.376360551361510.607006680467514.579642501380911.838902854089511.363346476563516.047580510367424.8765784947233

12.656459311537819.22036295710289.2017886828111811.01903193055610.34351227724956.910420625910493.35490838079926.82346623229139

010.51231322452641.011231679232781.817650030981521.147120486115264.9185214732558400

12.65645931153788.70804973257648.19055700357849.201381899574519.196391791134211.991899152654653.35490838079926.82346623229139

14.674218701041935.246524571502528.520287985501121.730528930622347.980024141207530.452875512425938.350289673153917.777105322195

3.814941983946800023.7821319082196032.233433462580513.1117586424423

10.859276717095117.610115619495511.56596233122515.592029172013418.432681156178719.73451888941956.116856210573434.66534667975273

04.726666634123665.0014972243135101.89119863927113.2435655120930500

010.18508186196278.660227952444321.9682861087794016.781367186563900

010.18508186196278.660227952444321.9682861087794016.781367186563900

12.353145471827819.862673833557322.845956939342128.798077591646218.90055390216112.0967623849536.9583284935094514.1523744077155

09.797572294822164.146899659374785.5904194230187902.017007125167100

12.353145471827810.065101538735118.699057279967323.207658168627418.90055390216110.07975525978596.9583284935094514.1523744077155

48.6814299202778167.490694731491124.827163055706160.21609484439191.0273855942539141.38087869163752.253028810066841.3322990142572

041.243113850584846.998196139580951.289879785791720.944703297505826.12504466883100

13.38729641155658.6792389807737.3471125478749316.50769010767349.302939011853747.7427047708027315.08169822561113.06742977414934

08.4250290162127810.486495490189713.43058337787713.5655719568423324.988514726643700

26.039458732809430.605166455950715.421283108316.631497783480924.954619797949331.830979254150237.171330584455725.8303708581223

0.81293331969897378.538146427969344.574075769760862.356443789567429.946349888680249.701801188999100

91.2579149570593209.526047554675238.623234065391198.752016894111156.437614012072172.469205904071152.837376852505116.379110343462

02.1491448904771212.50758445650135.109523128565580000

13.265593716906108.219541506276117.725135837866100.7825867303245.052816944039382.24566634426539.0215976658655410.6384041712543

11.902435945916432.763709077322621.025832288061620.288574179937524.187649906220719.831479952391213.452722050476427.2274764132444

57.137644135588755.735752400151964.259730579071561.710851210902777.512724173012853.9986267177092116.243626772878.5132297589635

033.182510379126831.480559267029636.100192464333413.829911684957421.435778395005800

033.182510379126831.480559267029636.100192464333413.829911684957421.435778395005800

102.175763096551258.511036985407251.259621517009310.223283994361167.880431491111217.5620716802643.509100210256259.4893787366153

102.175763096551258.511036985407251.259621517009310.223283994361167.880431491111217.5620716802643.509100210256259.4893787366153

9.5462683757173841.888927359057746.895489291762865.684807998814424.98533162174940.610058988010110.75451938891716.2495298202295

9.5462683757173841.888927359057746.895489291762865.684807998814424.98533162174940.610058988010110.75451938891716.2495298202295

39.8222643123426477.279932095352512.029449256637511.245802468158321.200910837541484.63014915282237.212605780849714.908128856163

8.5607298119766619.506731537485819.719915120066727.083882850521311.319061314616722.171810044871519.28848658400824.45799793843039

31.261534500366457.773200557867492.30953413657484.161919617636309.881849522924462.4583391079517.924119196841510.4501309177326

258.172072757076139.446069120464135.793021317944142.234549943948195.347872854751159.445284968653258.673227871872283.927144453669

258.172072757076123.764363131909114.524426108342126.99426008041183.487319193999138.427367266414258.673227871872283.927144453669

32.156354584705429.185390488696529.434239282959119.683236748317422.121204761124936.117030822536329.763796813505919.0911884264986

011.0086026900626.353833383677221.903461835019283.60382916667976.1808716196107700

32.156354584705418.176787798634523.080405899281917.779774913298118.517375594445229.936159202925529.763796813505919.0911884264986

9.1609929704193663.559051941850847.671481548802555.65525593774443.86975760256757.834961972628310.320480489812813.4939399481637

9.1609929704193663.559051941850847.671481548802555.65525593774443.86975760256757.834961972628310.320480489812813.4939399481637

15.643295353991456.322634331927834.643655959833746.132027937640633.164233848941750.700128879953721.32868937943217.92233661744961

15.643295353991456.322634331927834.643655959833746.132027937640633.164233848941750.700128879953721.32868937943217.92233661744961

039.596980859451158.193521163396137.656221396560623.76487346706730.569074967744800

039.596980859451158.193521163396137.656221396560623.76487346706730.569074967744800

192.0842443450451283.105557929971309.056192208881285.00739518521949.7246663094561169.84019438458209.637700631446248.914644958549

062.498101901570338.863587116162852.922702373301241.008581991679171.806657826902400

04.462817619459664.7223067891444810.61020592247596.6961100146440910.33595517250700

09.2340992516052314.089196887511510.31478959586877.861610196899875.5267343148456700

01.880501779167485.9695289451483600000

08.855021036079799.369893534156932.807003845313252.362003363815391.0127588518771500

11.9786331324321.761569568799728.049731259193645.133399267828510.877182117340225.337708798993117.175127979315311.2281664494049

63.814660040114517.71713849412213.94957527752775.4034398702443724.862051163696722.815144618207366.363580772633664.3640638198253

5.73699502573144451.205059600618461.326124093848469.661266384251383.180706011562463.0397013575184.0095246502234320.3871856940414

36.1431098646565572.503593122847612.389668572771591.038665115457354.586102387763450.59157386987423.781397344072942.9185395021569

3.297661714937070000014.86015915563045.66694653190303

039.196672276618928.754954927815223.193429461917319.46635164469337.253632495672100

47.07146157611796.758904945413562.681962279704345.6241779944138429.11508704590815.4142826839308955.680656836167278.19472190392

02.91477775770963.0842566216599911.08766518898733.49871748265154000

14.776863887186400010.6290151371692020.80892539989388.46455304765261

08.728746226296127.916808440624584.743386177106874.490332598055445.1341999549707900

131.356994056761140.022147976488153.316432408044130.95014463723107.848492281404129.78416104044963.078034667689949.8734310460414

115.60573682288199.8971623097056111.96721452635991.768729053286481.929857347861194.000027133595948.845090021875242.4908247620812

15.75125723388050006.502905825999541.0390642766012314.23294464581487.38260628396025

040.124985666782541.349217881685239.181415583944119.415729107543734.745069630251500

012.272748453514104.668490605889395.89257681288687.5797004598384700

012.272748453514104.668490605889395.89257681288687.5797004598384700

714.387351714145653.744391895672558.243242562215604.552987637988707.784403225171554.179749702979747.162648873022715.124505413658

7.9957003225186621.457079988506613.214674987424712.940953355336213.75525946672225.1641915220877631.5269472497029.16026973650079

13.012111034417339.596980859451133.053920020809132.216989417057411.28873536524831.248387744805816.753173060806210.6480842478433

497.518289062138356.878576825159320.554374441232309.128741595677444.023178856777307.355500056587435.393102495807435.101790768864

74.6184704119615116.457858121571100.230203633207128.216563564331131.970930862636115.43873909639195.2242091993868116.225028363133

121.24278088310957.480593885728658.496478940299763.829879023302480.864565214831173.9279542630844168.26521686732143.989332297317

03.085587040297492.7985693353436611.73741494486374.232852909068035.8985066590425300

03.085587040297492.7985693353436611.73741494486374.232852909068035.8985066590425300

15.226594527231249.49820094676446.879273578498827.23674839000410.566608770361521.834255414666700

15.226594527231249.49820094676446.879273578498827.23674839000410.566608770361521.834255414666700

1222.6097531443902.036919201029990.631549062318932.1610581883991178.581523142891161.615316994541072.55425160351247.80750803398

27.329785048876600012.16945211357061.605510688260116.494031169815519.0098574297617

03.355139864989482.662667587044610.7976737546034021.0068251748637510.360741635750400

15.0953307813068000000.9447730497652910.96077541776517

41.817264147704827.087162287810322.57383368669915.205733007447916.437381082051624.110723016102225.112071038793432.8423874538438

20.12710770840911.400019674597526.318989838165223.653685736506215.946644894108313.868044545037802.88232625329551

40.16960329823300016.26018917408935.0321197386047935.656122140905539.8951516089352

12.750425028870828.219504405850840.861489000336215.536855678835739.818438285028230.57628635603629.5761462519899616.2305750185572

65.460126004919117.390733784839849.00603242306923.598809066450651.299321152140245.265028486981786.036155111336553.1210034719507

7.9479476927855116.655872901197712.20145476700667.310548476255373.075795589144227.6376437027230310.0063789096887.26847489961476

3.7415777150247725.306597586315913.021190746185313.1570982266456034.56515161992524.215141298952830

09.496108531904563.3494189556488710.03408614387991.266504066118210.860807597053600

014.812034246695921.119771129734318.26560455076170.96184673062585222.644791456665600

40.833790125055711.860678919451624.329798670346714.810457231513521.55977139444559.158325770450909.78351004211689

04.286437878984717.257074403905864.891616995141463.087103661163133.5297624690424200

1.726882022319122.75955290670737.300015672568033.936449179522121.656197624923814.260778364997951.945449830285930

027.692316529910737.607297581714923.735485994569238.026291923047732.336386081185600

010.30639649134885.45282938636023.67546912342119014.587084678694700

09.68230673841255.122640755698240.7673124698261116.403404053309227.474791110913377.980121876658290

6.2915936019321525.596461314189818.547689707486424.178466451270223.745386799816123.418966323963619.308623238073614.9959745227294

09.268067943244233.9478484757247819.42981328355872.2391791888969834.380558784181600

484.89163367127140.679290985123929.474877482450233.881755829806284.653216228668118.166166757829405.855669642581551.488779780968

09.783869396507738.627291249398571.550722404054173.914648931638085.0354653404521400

029.139525203960232.059754170215438.705876173891712.152177725924139.46818795807400

01.689726236353391.787974853136236.4276319936158206.9572129824604400

352.18003047997898.4803555016791106.42994017201684.7689584953167252.028476782949161.829097226405344.784728092383408.282527896797

12.0200339381873116.229866341773128.235387259709118.11793587456887.9327766767995133.07871480296834.04527864243521.3067530412065

3.9042550069823710.21368612271316.087084932981812.52798290014418.797322463219296.7413204467284829.689256105667710.0640421686974

04.513204270001951.59187438537294.29199942799509013.936868587444900

015.37465190879795.422868785336260010.550498808566400

039.89227663985126.059262964762331.378926143870812.96366898835126.252232386297700

11.673179321781225.854689400218648.450148717879347.442047068191134.044190471270232.82684484746293.151255795383278.37028648929241

2.79275657198024.462817619459668.264036881002853.1830617767427601.1484394636118900

4426.011815203275602.784621838485061.863892189385344.641820033254947.259290933724920.879733735763303.937660679974529.16232359271

4426.011815203275602.784621838485061.863892189385344.641820033254947.259290933724920.879733735763303.937660679974529.16232359271

265.184246586595250.03713179801186.802695181332174.450136149395187.429256785867172.267310616256.153266323126171.79457645441

265.184246586595218.484754772706155.721862713671146.006228132973181.028827780494138.283757305258245.984781127714168.347670831912

78.635491644103741.053543673738220.077154600867936.983358927552364.631252847074425.0295237439894132.42568914210291.0174579096202

118.3636908580280.759218615411185.061848383292953.433880911475677.782040716449672.741379183644256.835642547512947.6805235856081

51.48304989994896.671992483556350.582859729509755.588988293944932.024011063506940.512854377624945.450957278613624.5837825884677

031.552377025304531.080832467661328.44390801642256.400429005373133.983553310741210.1684851954123.44690562249771

06.7006385234703511.3443921716233.823332823788711.608605739150135.5177896067789700

05.8417257774555512.8778982115245.555406357738942.921684745429275.0109362817303700

019.01001272437866.858542084514319.06516883489481.870138520793723.454827422231810.1684851954123.44690562249771

281.540619245699778.251885051564758.937332955036766.076365623068527.157880620498596.751824683128329.097755091123244.479358260019

281.540619245699778.251885051564758.937332955036766.076365623068527.157880620498596.751824683128329.097755091123244.479358260019

25.443083534786674.397560853078863.024314518215764.606977847811333.894734165850582.498973123125521.346715579465523.1942810889154

02.045458075585682.164390611691220.972602209560288014.738306449685900

19.889165544957811.224594483784512.535545750577513.347868273081814.78538872673578.6792929431173870.877688879471318.1358052432669

045.348720917799738.649159937003239.84842679623132.249298912289820.87920387632200

014.597083306513913.875064271446623.116458457676509.2104641897771900

015.91622065065113.8553207770749915.62242299106221.457798951104816.908581148290300

29.199569935654145.520375281164240.455199613837643.853586634122338.823236243304840.723747537582257.582533824334924.3034832320362

207.008800230301514.770756060003563.944961555471549.686324316601397.275152000787387.285485863788179.290816807851178.8457886958

022.42571259323088.33825191320873.464895371558524.3394945521259511.832570989973100

032.005402829752412.095124006509811.55680272536344.3327770682991113.995198561466400

26.367533520033126.486390616383101.258262268497133.51297076518779.8661493028061118.43932260200320.144058013594116.2911494225245

26.367533520033126.486390616383101.258262268497133.51297076518779.8661493028061118.43932260200320.144058013594116.2911494225245

26.367533520033126.486390616383101.258262268497133.51297076518779.8661493028061118.43932260200320.144058013594116.2911494225245

02.989515648932919.490020374338432.8429910740993206.1544576383303900

02.989515648932919.490020374338432.8429910740993206.1544576383303900

02.989515648932919.490020374338432.8429910740993206.1544576383303900

38.2279459656346106.53229449636688.484980893065179.0138064573113113.27234933685897.268685546017539.613828145367565.1490507546352

38.2279459656346106.53229449636688.484980893065179.0138064573113113.27234933685897.268685546017539.613828145367565.1490507546352

38.2279459656346106.53229449636688.484980893065179.0138064573113113.27234933685897.268685546017539.613828145367565.1490507546352

166.002286340124508.822053534663473.765724359304501.799540836913326.627578112471470.112345164315190.566798119516121.319720962622

17.1106436345922133.16632481849496.1586267810809158.99063581268858.4923390965626113.04916681221225.00393481454466.74319015897032

092.168211494009869.785865259711297.722428343061540.343134796697374.705590751334400

14.46952995339837.402610178310095.8747745174476121.11936226473777.997068531774945.5243584039298925.00393481454466.74319015897032

2.6411136811939533.595503146174420.497987003922140.148845204888410.152135768090432.819217656947400

118.441622977057375.02035481803377.607097578223342.204672588858253.226232485989353.075497863689137.21259244992379.1916782979166

13.008907336328259.905181142058455.007120793657957.013473602912244.385204206860255.380585238656226.24581752631185.97193613373754

28.257953813688830.762488036284920.963170467102526.483354426928535.947409756269211.93767081233261.475376791806333.3744551847403

21.44626273199934.063824845986624.718143036033850.076320926156727.680883225799836.604507719756519.68768181280526.30848764872224

088.8986069935914115.84786489103969.769916207664538.600732079020883.810385047803400

55.728499095041416.349420759407415.06805965837114.564033332385539.421215407276720.888675223863229.803716318999733.5367993307165

069.752314749016860.095248868937250.244929380387823.872617962348856.616681899339100

1907.114591007652586.85808988442419.260806890112589.881420397382317.286735445882299.451697858991469.883652564691845.17530036436

18.682782750629160.954493394628238.432249241224201.92038058485272.9995963267691204.54486336696355.44553070966748.28730385990263

18.682782750629160.954493394628238.432249241224201.92038058485272.9995963267691204.54486336696355.44553070966748.28730385990263

114.29643277964536.18035265661347.635607081076540.866620120601956.090971922035943.149450435897478.580337090326732.5523232922729

18.773530289422516.51754860255568.0458868391130218.532245364916520.940389416674519.10350159907437.690784124405187.82104901479016

78.84615610325519.662804054057439.589720241963522.334374755685421.822134952403224.045948836823161.495809499683816.7705636731428

014.453443426659219.372190350922318.32671932064025.7830041035562724.795852055256700

014.453443426659219.372190350922318.32671932064025.7830041035562724.795852055256700

41.495815817628241.027274663645623.599535076201129.292015257607240.835419069114853.846062548669542.673419190407949.2083351198486

021.40768493595497.7358884683802113.40595313175245.8137245536683218.063766153629500

30.22774393454216.503089817997512.77938998616096.1843550854909220.9727903380048.2930786800515129.979171263059636.2990746803784

013.11649990969323.084256621659999.70170704036388021.002086690802400

972.77777462013431.594743258898826.480339067483821.8411129785907535.018860723987115.227418066036473.3996034172841030.74503245166

972.77777462013431.594743258898826.480339067483821.8411129785907535.018860723987115.227418066036473.3996034172841030.74503245166

688.4487228790732003.588155207641734.745762539151903.345819806771406.491924036281560.26378795072695.755466264241630.865567358523

03.13416963194584.642966957337622.980555158329926.771711256744915.8070285781020600

08.799329079878033.491611269803764.184024599617852.64054149634079000

042.575387191638231.773719093024837.298361696598924.355985762058629.879611788884500

4.2092749294028714.231011694105421.49234625399723.524127957077426.904807591926812.933624455447415.806779871073112.8596094377799

236.7303565494091267.839594898721132.376728374571240.93835786283856.34067352012987.601390317702337.34309311306230.204000958796

55.396282339708740.452794344185928.746380818314521.957784324040633.255919081310742.299322531184530.649135069168458.4029998040573

016.287979651600623.144992069896814.443653782513113.20270748170397.9253157323782800

93.0171138116883202.116138712645165.592610661746161.063836738402101.113411151208146.08305595358485.518354861113474.4346694078365

036.415629540642634.568815119042130.18846832023422.55068560870629.291119865920500

04.379008837873563.475218728630978.328762583276853.942216881860881.126872525328100

2.74675822844172105.833712511201105.88044387215774.402819466130958.848738966678861.79069569303863.868012015509672.36011655563961

31.26889947556410009.996335664718683.3543705451148425.526476810428628.5547228199151

019.05380012821845.7604792933727411.217093187302313.069139234807312.141284134760700

04.7979880785343208.213085325175784.607364586207782.9632573814183500

210.036432417174218.345530485592161.210288061564247.364660066562180.967134125448200.19016164275151.274895505155170.205293416268

43.573790472892600022.35291725027372.5002484155717228.540019211659929.0234240783229

04.020383114082212.12707353217935.734999235683083.6193629130878000

11.469814654792100017.1116313561997017.228699807073624.8207308799072

014.144273019271121.887300958593314.78355358531642.392285458223271.2246114688514511.24037679720762.85769098617331

01.189705207228418.8121617761714001.2246114688514500

01.0138357418120318.23734350198951.928289598084753.650835634071182.0871638947381300

01.0138357418120318.23734350198951.928289598084753.650835634071182.0871638947381300

5.7224129759202221.223013127385127.15376856841127.462918325767511.081461463617230.08854245613323.2233433462580516.3896983030529

5.7224129759202221.223013127385127.15376856841127.462918325767511.081461463617230.08854245613323.2233433462580516.3896983030529

40.5513306734086112.917879440478145.333997724104132.814311386733122.037977782525113.43405839218481.770362602308258.0281815360615

6.209426846211320001.985087933419315.106890380742244.6635605860754818.9702039933208

5.165363925166936.529102177269497.895696951449587.096105720951886.073525853070147.7827201653704327.640882323221611.8353927568948

23.419112763777112.955678552659311.36068459800349.2500199167320916.074125192813311.216067658639515.10271160755996.27442780335969

07.320169585616520.436681060418915.40746812788158.638808599139592.7583512858947300

5.7574271382532486.1129291249323105.640935114232101.06071762116789.266430204082286.570028901537634.363208085451220.9481569824862

11.1588125580671137.77916108056890.9648217683366190.17136703576953.516174790271136.7578468152912.40683099314427.41560634204104

020.47186631705916.912731146004148.653854757308913.79775908651318.4515439399607400

11.158812558067146.90793511450429.281819065431969.871761922800812.855197880303445.865438710124212.40683099314427.41560634204104

76.0799599566505142.088944906915143.173609508497103.362823888814127.361710291343116.75873339635249.1565749042801101.781150345284

76.0799599566505142.088944906915143.173609508497103.362823888814127.361710291343116.75873339635249.1565749042801101.781150345284

2.5231388625815421.940865151711516.749207163680114.599356101789124.648796391318619.28516004663334.7374786933475615.416707568059

3.6480382721491314.0112017542629.987829205375588.808272527333736.242809625907669.71594411581788.2195255329580412.5381192018355

63.423381449210363.387898384963655.574164160950454.562724530804974.497271664152759.419706429269131.328740732517971.3496580540398

10.5849815150441141.635383443541160.384401044239147.999647771602102.57558995897119.8031033242398.70197163371154.5593160733947

10.5849815150441141.635383443541160.384401044239147.999647771602102.57558995897119.8031033242398.70197163371154.5593160733947

10.5849815150441141.635383443541160.384401044239147.999647771602102.57558995897119.8031033242398.70197163371154.5593160733947

49.6027979520327280.903437743341182.660423144989222.940276203416147.171802099121219.80295948454591.185071861972105.214690036581

49.6027979520327280.903437743341182.660423144989222.940276203416147.171802099121219.80295948454591.185071861972105.214690036581

23.538796605619699.710210083965160.28758552827768.536017082628557.768369691591552.438850780114175.017319536106739.7984159400486

090.903140511330974.321237753980586.216876521183244.8377853922552100.92774799159600

1391.39774822143433.463277296593338.532527946812408.348199305571763.620108702501453.199856977195722.1878172130611602.7737345376

1391.39774822143433.463277296593338.532527946812408.348199305571763.620108702501453.199856977195722.1878172130611602.7737345376

1391.39774822143433.463277296593338.532527946812408.348199305571763.620108702501453.199856977195722.1878172130611602.7737345376

22.800239200932173.842258733266846.597951902028757.075957287642762.410111902979468.594076203790613.356728991056818.8071788027532

22.800239200932173.842258733266846.597951902028757.075957287642762.410111902979468.594076203790613.356728991056818.8071788027532

22.800239200932173.842258733266846.597951902028757.075957287642762.410111902979468.594076203790613.356728991056818.8071788027532

778.8084616824761099.30983165194989.4967304968571068.37339195948793.5811025314291016.31078476768893.196802095274748.352609024193

778.8084616824761099.30983165194989.4967304968571068.37339195948793.5811025314291016.31078476768893.196802095274748.352609024193

212.33764295034200094.848952353609726.3149069120639198.797826843636216.605381559948

212.33764295034200094.848952353609726.3149069120639198.797826843636216.605381559948

212.33764295034200094.848952353609726.3149069120639198.797826843636216.605381559948

435.074060494846931.632725340808829.838488080065909.468303479298540.536432687938822.12471846915450.764956337034345.507753862606

29.640310961211849.502853875237634.706939518219245.889760308855522.208790776077525.4453775651452.5686017290493823.5089735034415

29.640310961211849.502853875237634.706939518219245.889760308855522.208790776077525.4453775651452.5686017290493823.5089735034415

20.042077902077553.84786739235646.879246101500946.472731573752220.217932697004640.074161705954634.969517187479815.4872355636401

17.208650117883953.84786739235646.879246101500946.47273157375229.3481308091552435.413504465277234.96951718747988.99500555621717

2.8334277841935100010.86980188784944.6606572406773806.49223000742288

014.462237044631814.361755339878828.27840256361685.570097484818379.7509917146809500

014.462237044631814.361755339878828.27840256361685.570097484818379.7509917146809500

227.934222775086503.87913274468421.307546604832472.682341116891265.556990976797412.819919514696210.364912361164174.631501992227

171.439084169711172.538501436877151.430471253781164.928962378818114.25217004217158.068284874963156.05905989434150.672205539356

4.5959537286918447.005097123629542.451184634591740.160543046052225.872052986450836.0577053478095.177653879028675.26535189578391

51.8991848766833271.136540564356227.425890716459255.040761893167125.432767948177218.69392929192449.128198587795118.693944557088

101.13759956749228.699494853382117.262725963019519.557040458578674.157963075414640.8637174491588141.65118258531883.0117438352168

101.13759956749228.699494853382117.262725963019519.557040458578674.157963075414640.8637174491588141.65118258531883.0117438352168

56.31984928897796.372242751488916.651468376083420.90313694362431.973493886761626.060623879207247.396413847202846.0586364018426

03.643472197136995.140427702766641.154965123852841.457798951104818.7508694545010100

16.424068411407406.3805108622929417.46205528595943.635031150806789.486272006748315.6931778583259117.3688231367418

0210.36223294296197.114130769372204.62388462891689.3089706221984216.12442749642513.81432862682022.80966256623764

018.27218246084056.747300200216459.231910575784023.998534265887472.8405189099986500

0190.702061073686172.634287486228188.79217334540183.6443804121912210.42648182577313.81432862682022.80966256623764

131.396758237288167.67710631113159.658242416792158.905088480183158.195717489881167.87115938647243.634018914604186.239473601639

131.396758237288167.67710631113159.658242416792158.905088480183158.195717489881167.87115938647243.634018914604186.239473601639

029.710892422933820.394499273555720.545170232078614.641592976321527.55404785437600.877558649297318

07.3858621914813325.678878659974524.08180674531185.0660162644728128.238099752339400

109.2714719657718.979948189736925.821683344130128.363794669502556.630403905243523.4441897943841218.677609528164.842249351263

016.33760781391371.571595730782161.41244142534878.913929892105836.1152572712072700

22.125286271518386.62641715170277.96690108392376.288790082765867.760489292253573.629792569908423.738701900239820.5196656010785

140.280349413444208.93697085409209.047830845365222.218087226913167.3790608851207.420733428855144.463220883452137.177723039017

140.280349413444208.93697085409209.047830845365222.218087226913167.3790608851207.420733428855144.463220883452137.177723039017

140.280349413444208.93697085409209.047830845365222.218087226913167.3790608851207.420733428855144.463220883452137.177723039017

113.02215480102565.741187740064685.270762098426479.869986402117255.968512995165977.6211560837632105.38678802184882.3662729763489

29.883712207583455.557547335957671.311248257464171.872927638844429.665205261107970.170167398923532.796239443673230.2819187694501

83.138442593441510.18364040410713.95951384096237.9970587632728926.30330773405797.450988684839772.590548578174752.0843542068988

046.638716435280740.636158137878639.051967221213321.106882908241733.929444456245600

046.638716435280740.636158137878639.051967221213321.106882908241733.929444456245600

27.25819461241996.557066678744383.1409106090598103.29613360358390.303664981692695.870132888846539.076432861603854.8114500626685

27.25819461241996.557066678744383.1409106090598103.29613360358390.303664981692695.870132888846539.076432861603854.8114500626685

175.70862071177998.8079192835911103.06321246253786.5763410481643185.674034618272122.103263369183177.861472141238168.45235528775

175.70862071177998.8079192835911103.06321246253786.5763410481643185.674034618272122.103263369183177.861472141238168.45235528775

175.70862071177998.8079192835911103.06321246253786.5763410481643185.674034618272122.103263369183177.861472141238168.45235528775

175.70862071177998.8079192835911103.06321246253786.5763410481643185.674034618272122.103263369183177.861472141238168.45235528775

175.70862071177962.575696868688445.473779694231345.737523588906173.69395526865172.8916185680329177.861472141238168.45235528775

028.061618888345832.07338688940934.91662240282599.4884111127175632.39098246351200

06.2274183547504813.17901939225645.922195056432372.4916682369031712.820264872723800

264.335006838023926.99833339539991.52410719295975.637687467462622.976888828267950.769099609748234.737548873102251.251575501072

264.335006838023926.99833339539991.52410719295975.637687467462622.976888828267950.769099609748234.737548873102251.251575501072

264.335006838023926.99833339539991.52410719295975.637687467462622.976888828267950.769099609748234.737548873102251.251575501072

1.48899521312218.8867668834540811.461098567590110.9658227143836.500617628120385.6587338467931310.06472514239760

1.48899521312218.8867668834540811.461098567590110.9658227143836.500617628120385.6587338467931310.06472514239760

262.846011624901918.111566511936980.06300862536964.671864753079616.476271200146945.110365762955224.672823730705251.251575501072

18.2401913607457410.405857147389393.431518908562420.728907647709250.861655313788354.9284519016258.562005763497948.70702722349684

83.541148200185391.652957345094436.979906349687418.217978878495225.646709748299455.87337309040385.533681369509592.3698934593722

15.991191421143622.603758018709514.887509403406613.36492975642243.8769832703741313.706412324233212.87043143950722.3797142020091

037.462338805831243.472180563083436.78130311605348.6001824758327155.448131142227700

118.6372020793054.4543483918284731.805779181776214.603376756838188.15396665007515.276732606409686.9982068513546109.353629883254

14.717947511774100020.26843231329173.0002980986860524.44600266267730.4806001286

11.11783092464516.565709413587119.419741099908616.60240761752078.7292894986849413.92660980734216.26249564415857.96071060433997

012.895852228239319.250814026251927.03422529355815.7316873435929720.109574805882800

198.873427085711161.759856685702143.335401035932170.16282415613798.7448516490784103.15006055855124.0490428021550.2116812987574

198.873427085711161.759856685702143.335401035932170.16282415613798.7448516490784103.15006055855124.0490428021550.2116812987574

198.873427085711161.759856685702143.335401035932170.16282415613798.7448516490784103.15006055855124.0490428021550.2116812987574

198.873427085711161.759856685702143.335401035932170.16282415613798.7448516490784103.15006055855124.0490428021550.2116812987574

198.873427085711161.759856685702143.335401035932170.16282415613798.7448516490784103.15006055855124.0490428021550.2116812987574

13.684667222791964.811841037170278.1532895760447101.61358672784980.270262898460770.768934682618517.081249824759821.7739718176894

13.684667222791964.811841037170278.1532895760447101.61358672784980.270262898460770.768934682618517.081249824759821.7739718176894

13.684667222791964.811841037170278.1532895760447101.61358672784980.270262898460770.768934682618517.081249824759821.7739718176894

13.684667222791964.811841037170278.1532895760447101.61358672784980.270262898460770.768934682618517.081249824759821.7739718176894

13.684667222791964.811841037170278.1532895760447101.61358672784980.270262898460770.768934682618517.081249824759821.7739718176894

1.065120663401210008.172186820791930.87599944487184316.79903028633767.3215294609258

1.065120663401210008.172186820791930.87599944487184316.79903028633767.3215294609258

1.065120663401210008.172186820791930.87599944487184316.79903028633767.3215294609258

1.065120663401210008.172186820791930.87599944487184316.79903028633767.3215294609258

1.065120663401210008.172186820791930.87599944487184316.79903028633767.3215294609258

95.7067925478622570.847472601551629.706673907641685.837054811241433.381296738962553.38509673552842.470174272079562.9660980276055

95.7067925478622570.847472601551629.706673907641685.837054811241433.381296738962553.38509673552842.470174272079562.9660980276055

95.7067925478622570.847472601551629.706673907641685.837054811241433.381296738962553.38509673552842.470174272079562.9660980276055

95.7067925478622570.847472601551629.706673907641685.837054811241433.381296738962553.38509673552842.470174272079562.9660980276055

95.7067925478622570.847472601551629.706673907641685.837054811241433.381296738962553.38509673552842.470174272079562.9660980276055

1497.17650606876978.2119665692251122.89875067081946.5915645058161115.99496775497984.3462513361632100.38293661031899.34258879351

1497.17650606876978.2119665692251122.89875067081946.5915645058161115.99496775497984.3462513361632100.38293661031899.34258879351

134.765884209876112.758170956755111.005385475267112.020512681907137.465559177203126.11909483040376.7901251605151143.726026829376

012.13825258005093.380007256613697.290519576320422.300526563935263.9455974996419400

012.13825258005093.380007256613697.290519576320422.300526563935263.9455974996419400

048.471434880369537.828954212433852.781558380387338.411158493237566.164139914013400

032.928852172482821.797439244046932.680392470003618.544926164202331.845528587695800

08.974069451076357.34346814680955.234324699234562.4416337112100217.415523446396100

06.568513256810358.6880468215774314.866841211149117.424598617825216.903087879921500

017.255936051715510.398899940229721.102527149133628.096719154380717.50523411215900

017.255936051715510.398899940229721.102527149133628.096719154380717.50523411215900

134.76588420987634.892547444618859.397524065989430.845907576065668.657154965649138.504123304588576.7901251605151143.726026829376

71.80265805499900019.992671329437420.954462911458236.531224590924753.071404028933

09.965052163109743.163340124779474.7383184568321704.1029717588869200

62.96322615487720.67983154698766116.46875565985312.5860443589474733.45712344926264.1986678932049840.258900569590490.6546228004428

37.681382975696833.32620694095228.320306786838140.428470663241218.035668733249130.02743791192551.92269603110130

37.681382975696833.32620694095228.320306786838140.428470663241218.035668733249130.02743791192551.92269603110130

1.2010002542054814.393964235602915.230896897086424.639255975527310.366570318967519.755049209455600

36.48038272149132.727277434114242.885854148921622.593605892160761.636826892468564.210944699910251.92269603110130

1300.40898373553832.127588671518983.573058408707794.142581160668940.50106851508808.1977312692611990.35763719791707.8522983381

1300.40898373553832.127588671518983.573058408707794.142581160668940.50106851508808.1977312692611990.35763719791707.8522983381

1300.40898373553832.127588671518983.573058408707794.142581160668940.50106851508808.1977312692611990.35763719791707.8522983381

292.404797216987814.073074964276804.15958747807884.482915958287509.99368008881710.484356137966444.153563809649348.258202736611

292.404797216987814.073074964276804.15958747807884.482915958287509.99368008881710.484356137966444.153563809649348.258202736611

146.951289633217702.129245458143656.324569196017764.59237040317380.30980179013590.565410924781187.645862950596152.539589139387

8.28461958688985348.71558399971294.305812171906343.196248039451173.309014232709257.0765229053940.3807974911632.5659162619323

011.80669471477316.2465956894379611.228015381252910.6290151371692000

033.509291883625829.726510069298742.302663791922413.014770914521934.758256558718600

4.7071461576117957.880525191707176.189866798025466.541945972774641.966388268037154.68806011187522.5388495854696713.3673309711934

3.5774734292780640.008767107288853.74694875519232.811611037830136.493314290646732.46548168579937.841947905690319.1985852907389

0167.841277208402103.054670828547159.70801635623658.8563147844847108.69198442926800

138.666670046328349.307593765962349.834039506441404.60972867279205.5276433542330.525630637972143.206040504889119.973672877455

23.807124489600486.75733544918368.049265107632986.781834011125142.636484152071360.373555284244337.182512884555511.144994846076

26.784008732103665.780603595985883.068069908293494.7048759747545.210965525395272.390783642985225.11081368151569.17423356231862

05.2995959231083611.21547862421817.559771719764066.36130451391195.4550874521564800

1.1627213616411654.396949918336742.341492235489940.273298514203231.390598029518943.72355872655640.6549422735424742.6641421942811

03.643472197136996.4255346284583212.704616362381302.5002484155717200

43.281536540813237.897063122826553.549904121604650.115764890645921.002282555393642.337332227204229.526450943635556.8969580855837

031.756244264093840.961673901000648.55438161912019.5144578466148955.681728828866900

43.631278922169342.867911625314633.759556172294631.617639021335239.704124407369741.084978028573950.731320721639840.0933441891952

02.878792847120583.0461793794172710.95078043356773.455523439655851.4816286907091700

145.453507583769111.943829506132147.835018282053119.890545555117129.683878298679119.918945213186256.507700859053195.718613597224

145.453507583769111.943829506132147.835018282053119.890545555117129.683878298679119.918945213186256.507700859053195.718613597224

125.40133414053590.445692754534690.871479911120592.7509474975434111.3485764609481.7022702534826225.488308862533166.038586440786

15.71787054563158.8059618233144220.635627715480412.9767203889394.8615688560492114.209299031767919.625990267667626.3696326477019

4.334302897602940006.92815343099314011.39340172885273.31039450873542

06.0566810549809919.2265347843745.759826072201190000

2020.078663009411743.0590872431737.488796548081627.676210840021565.007543816961756.733909992811579.619830765011656.88114158338

2020.078663009411743.0590872431737.488796548081627.676210840021565.007543816961756.733909992811579.619830765011656.88114158338

726.444098787554576.05691718769547.383927504794537.317047893294461.644441520868564.898354577738414.238245624898636.512310440517

44.823420321285184.033690958934130.589773197039152.38533284454467.9724984379474159.99371030151761.11932612997560.9211255611134

023.960504675891927.467210973350938.56324822299839.4523341424574631.646300415451100

44.823420321285160.073186283042103.122562223688113.82208462154658.5201642954899128.34740988606661.11932612997560.9211255611134

8.75529185315795116.24008330960687.3408412774596114.69176249253545.23503119505697.6613161757252040.31865782551

8.75529185315795116.24008330960687.3408412774596114.69176249253545.23503119505697.6613161757252040.31865782551

22.9006038902377018.18088113820637.0027359088340910.535213089706711.560957267026439.118124705497427.195920437793

12.28812891671290008.838865219330212.5265668199461731.147675703841121.1168323399334

10.6124749735248018.18088113820637.002735908834091.69634787037659.03439044708027.970449001656276.07908809785961

65.2132012879765101.63131225852193.872611582982890.003192025612460.862701249397895.761582166646575.411242007804565.232088262649

8.6544683864393631.731466667004329.120032062633931.484037520936611.721193583336830.397519804828919.34006007754835.61932513247528

56.558732901537158.99847744285147.428473343387145.535602436788345.848597094153748.585473517745256.071181930256259.6127631301737

010.901368148665217.324106176961812.98355206788753.2929105719073216.778588844072400

43.6290041082625122.4090057124293.5633598285773100.50556822563768.3608347389908108.97499806472772.621057073383345.9757217532054

21.22903750756543.822659354373242.696617811287438.4823668112471113.21404027782568.1032465359104740.143341383589519.3538041896202

1.47395485743460.599315099299841.010190803283542.626248060315934.688139144237549.571607390438301.68863558273878

20.926011743263157.987031258746849.856551214006449.396953354073920.458655316927751.30014413837832.477715689793824.9332819808465

521.09413191091451.7428249482095123.83646048052972.7284563961316203.18997852325890.9457906020958162.745152361979390.312917279025

131.95042527512313.848132312971418.935056997311116.250872163610733.71836122169817.059557021841436.832903610976423.5928017227136

389.14370663579137.8946926352381104.90140348321856.477584232521169.4716173015673.8862335802544125.912248751003366.720115556311

1274.680750350911139.081274692181181.197945095171079.233915617151098.041115602421179.916540087891159.27160919621020.36883114286

11.286748245323321.276887756012328.437385796361930.658249715191925.263231138076746.34122435998955.449409193121343.69447342963844

11.286748245323321.276887756012328.437385796361930.658249715191925.263231138076746.34122435998955.449409193121343.69447342963844

5.4244457476060923.547298843930830.714863848812226.20698632815613.9725377612904831.432052437031217.37123848009490

5.4244457476060923.547298843930830.714863848812226.20698632815613.9725377612904831.432052437031217.37123848009490

351.153830292614297.685870233128303.991325469853295.609525114391273.910617972296302.924976226083261.281637196699309.337600650416

09.570912040240478.28606256565374.964626204024163.133179835210347.1648909819368600

58.504578586615538.795706027778541.051470525208930.423377561946934.391203239490840.426936688242716.800508181906935.2594974125913

0144.068126878664111.792611579621133.15486262433332.408993204872106.64534609350700

292.649251705998101.174854605747136.273790338057113.815680560914200.107692403154134.345058914121244.481129014792274.078103237824

02.149144890477124.548212529636844.087618502852473.869549289568534.4244027261729800

53.2520058671273130.76436790697692.968808494904664.461495521686948.857422528830789.62208142478655.64839553109935.7106894269477

19.861541703923146.841267700936222.568292309469215.78130204084610.62217369043536.742829509518.26561229546236.50124699354431

33.390464163204383.923100206040370.400516185435448.68019348084138.235248838395852.879251915286137.382783235636729.2094424334034

726.857654457743469.811519172504491.649582190784462.80054974055574.151383903641485.7748385045672.960725010479570.465191541359

177.966124907999.4688432489991614.195845626409928.305835986979964.010687100836325.4101764457429107.6851317136761.9139188734501

52.336581546021736.388696784820521.930801450829633.024834730828837.928396073489150.288494455273866.856418662351173.4777938895663

103.273539555658160.755782270371181.876974435656148.835023239762134.861576105093161.555616289377138.16419268037132.609437739374

74.519756157576613.583430327190331.142008607052415.070612878235142.148934254118725.611207817760838.709047595383514.359897205521

10.5910788546265104.41493286612793.807170524452698.858559148225660.645160640494484.47446557664523.21477651875014.04455458123723

239.51939784472585.940836257448871.514154270475868.2522986647131162.4975859291891.8884049467164220.250588194778231.06427469552

05.795061104085346.4240137918598712.071777483867811.26214384948195.6810378199972700

7.0939244999806612.100731168510733.309755128296527.336287833997713.038615060827315.813224620944226.043167760383818.044548004228

05.9599289614509319.63334132541426.309455626260785.323264166744525.3317733407236800

05.9599289614509319.63334132541426.309455626260785.323264166744525.3317733407236800

48.248093232904754.85597755562476.655813306690759.873010913261259.354180135647256.698847660234941.02730093384329.8205845096829

21.30241326802426.529102177269497.8956969514495811.531171796546810.99976346078596.384283954225992.39986146947684.8810196406172

1.2471925716749248.326875378354668.760116355241248.341839116714423.344634667310845.723142928206900

25.698487393205600025.00978200755054.5914207778020238.627439464366224.9395648690657

3.5374916578415958.170162398805235.384143855438545.110067934721617.201917641976749.163588498744212.95197962769146.07908809785961

3.5374916578415958.170162398805235.384143855438545.110067934721617.201917641976749.163588498744212.95197962769146.07908809785961

32.870473802804523.442085188461942.829485149526539.516656944653342.159970859639738.268535194900135.167564110063422.6924016141087

1.376618215905341.911329677186624.044926716931155.4529500929445811.88243673353353.0995965510706718.61024648971639.46273147308335

04.50593663027574.76793294169664.285087995743892.704322691904571.1595354970767400

31.493855586899217.024818880999634.016625490898829.778618855964827.573211434201634.009403146752716.557317620347113.2296701410254

5618.4906715060510279.263973405910094.77074700210695.83975520588795.184590729469589.589958949516230.049638818465809.40947539888

734.6485441974932051.797028737051903.744081460362089.684278216631334.57987427661805.27967674213790.956012718953640.406700691878

7.11812345785199120.541916641869123.506609454048112.92661942960454.9039712216355116.04502885997510.69206573392920

7.11812345785199120.541916641869123.506609454048112.92661942960454.9039712216355116.04502885997510.69206573392920

7.1181234578519924.55286281190879.9590303277563113.79589452965139.234194049903869.4988525059213210.69206573392920

070.515612326896985.852987062707375.82851788041829.719744986038874.042162518504800

025.473441503063527.694592063584223.302207019534615.950032185692932.504013835548900

3.9966058879442861.466066384244436.144683950737441.631211424782530.769932433298645.726318075374619.340060077548317.2742217035351

3.9966058879442861.466066384244436.144683950737441.631211424782530.769932433298645.726318075374619.340060077548317.2742217035351

1.5441431839784721.543717092379416.256552199695112.38273559340513.656907149306912.893431120478408.84523400482218

2.4524627039658139.922349291865119.888131751042329.248475831377517.113025283991732.832886954896219.34006007754838.42898769871292

23.662950954480972.257261855539779.9846256548434109.99451804037262.400960743423664.69067751214454.442986774571914.51824115381459

23.662950954480972.257261855539779.9846256548434109.99451804037262.400960743423664.69067751214454.442986774571914.51824115381459

23.662950954480972.257261855539779.9846256548434109.99451804037262.400960743423664.69067751214454.442986774571914.51824115381459

49.766453700083453.257702059053352.457532608744653.3772015797676113.31049812286355.122265273580264.152567720960959.9562038243852

49.766453700083453.257702059053352.457532608744653.3772015797676113.31049812286355.122265273580264.152567720960959.9562038243852

11.33371113677400010.869801887849406.384097501326623.24611500371144

15.296692998291143.016080829726933.982815517587540.171227674956872.446012589616437.841268475087418.870433894649820.3823390205872

23.136049565018310.241621229326418.474717091157113.205973904810929.994683645397417.280996798492838.898036324984536.3277498000866

553.206229526093983.1703006734841044.622024084641103.26671263614621.0007973764281009.0770183576553.444251820312422.362288833791

74.3995977900635148.859864114684160.439196282038140.4876822363787.9687216848231160.68211142047722.042412117785138.9120235557614

74.3995977900635131.140245232433149.231378503875125.79501115260385.0531237826135158.71470283117422.042412117785138.9120235557614

017.71961888225111.207817778163314.69267108376732.915597902209631.9674085893023400

023.376569690356314.351964794617420.20260955446486.1924203232770742.494698914340600

023.376569690356314.351964794617420.20260955446486.1924203232770742.494698914340600

468.85743644835800.334675022227855.811514727716922.417029592604504.575089569636786.807401940237497.77650797679375.851405155705

36.170234093117933.226489297581744.100920448234926.54916985303730.133009645364545.833416336419242.813444945711730.2480549600589

11.41994589542348.187707312074058.876387132167639.354831865192339.735561690856462.5344133007534418.583275117992110.1758648594607

14.395846096373319.860275042948828.901419144948314.994362804818314.066370638713320.190363966767423.589669691000614.9932666090708

58.0665961139905207.793501621668200.415913823857197.018880861072103.208632254091176.83146168011458.109717547818832.0691763463068

120.22023768526176.460089273411235.668921748637250.600341922235137.320874727231240.293350873127170.071960342049116.030832785271

44.3305916615593002.8070038453132517.715025228615412.153106222525841.617850799787616.9291060953053

3.43344778555214108.44618891937452.244923631418953.909537267937719.128213020811165.676317188846300

146.39640902708223.570799064845617.0367252623246.695238824922367.770165714537545.5607421021513111.68539947267117.853122185256

19.243038535161340.381411774040552.744838272375528.630345211761115.047002991582232.43035599266625.9075793073530815.9848531656866

0157.188723946421183.453192981765232.71470735116153.0756697949386111.3536868967271.611671673129030

012.316222899845217.09145699364738.81239078337389.8568843417977115.837093494642100

47.9491237842756223.576762014265199.182335990921222.66031236056198.986295549391160.926284276741108.0582206427101.1539119159

47.9491237842756223.576762014265199.182335990921222.66031236056198.986295549391160.926284276741108.0582206427101.1539119159

016.945813641717713.420311250847212.54958748669637.093317043966357.4168442931724600

027.49754995384416.0729787859174815.68989058939014.496777177111419.7638388096820400

18.538651512669919.552938705142851.074178396330439.500378008299385.529258769471931.826276917644893.937187102153876.2024309172223

024.590125083222826.01991040818616.12751300216337.1246610555813323.56597779331600

11.8295649359473118.47382872891291.3432928099925112.51666264689263.919090837860175.179867782766914.121033540546624.9514809986775

17.58090733565854.3996645399393.1036544620477911.15739893231435.281082992681567.5479197451221600

47.576754088462818.42304761781214.2877514872157812.143377284231628.498874086494826.433144956597430.825859948930733.5187757585963

37.79708443330740001.78278597842117008.51846739827462

37.79708443330740001.78278597842117008.51846739827462

07.391858723769721.152993129592529.3260735234472510.385685237716814.690017263180300

07.391858723769721.152993129592529.3260735234472510.385685237716814.690017263180300

9.7796696551554411.03118889404243.134758357623262.8173037607843116.330402870356811.743127693417230.825859948930725.0003083603217

9.7796696551554411.03118889404243.134758357623262.8173037607843116.330402870356811.743127693417230.825859948930725.0003083603217

4863.891515440438082.329757771848076.76852479838434.954728458267397.538429985267647.955487524595386.368289807065114.42428159986

4863.891515440438082.329757771848076.76852479838434.954728458267397.538429985267647.955487524595386.368289807065114.42428159986

104.929992973084152.61928634331152.492792209727155.78144211620572.9522357054708133.735288951088130.94305183051121.261964820161

21.020148550270166.097124735660553.87942657849570.634488991683127.690659808022966.126109132599113.151240852732920.9362445582077

83.909844422814333.589884585445424.452956920608130.745059829490128.510467610771121.3862929036474117.79181097777789.7950164703854

108.776495955005145.414099221087129.004928383852111.023317444222152.312921666631120.216786384326135.900103114862135.391615258039

22.221552926796893.858204694446953.207955655996560.158688153680829.309245925662267.258181481114315.02045275058326.78882934786354

11.570870290517114.008776808595639.644681070149924.059873349543651.856776341614610.790082576135518.82886906228275.89162723140578

22.95987476965171.899741471489395.422868785336264.8736989841702414.89762001532457.5793813279930943.441990545251736.9431548633154

52.024197968039800037.72530155206897.3050736315834658.608790756744185.7680038154542

303.29260908136623.610488818729646.404125598986144.9026021752053210.23505960659165.5074407362569244.187598466438223.072353470189

303.29260908136623.610488818729646.404125598986144.9026021752053210.23505960659165.5074407362569244.187598466438223.072353470189

1820.013179671591992.818224073351870.936263225311942.128702448172055.611525292351940.201459463011928.931499083821793.75452419802

050.35642039571821.795466740263848.610180956485336.523335258446844.0832415329900

9.4651803817923824.114398006905427.632370018368131.252705951309416.778452343386129.50840182602997.108778839315060

07.03061971708848016.71507314922711.406519591015694.8245999576861400

35.311971886995400012.94898997137941.7083547892874424.570823657241141.6449985351594

17.173176213951138.428212497200662.900896255700933.85742319305831.18059255483741.962854800196726.819044772556718.3155197715127

42.7095687109572487.922709056993429.13704881213491.081335732584271.59502079174383.52240078779447.060812855367631.9041074538492

7.2960765442982825.756968388845617.52320283205531.405453433033127.91882027636325.31742695784518.08295617250773.3434984538228

01.1601105503321809.929252408048321.39252437120459000

17.024178603362747.908795927417943.481848653751746.046580979346615.837495417626810.724096253243213.699209221596713.0024939870886

18.788717806719231.615155930396720.814311738703843.975875834295687.465471781608135.088096689774848.884477999247823.7407651430205

36.738394743499616.283626261513917.592262672699719.770588024467628.238417602621133.331962423917348.477760560951439.421790463824

5.142609018007628.36930380533421.324409658507334.971224708032516.234167355082723.88870875173272.896749086505031.47290680785145

100.753475538734128.793605300398157.645799296378202.633765072338158.442491901868146.85754431754173.0945246117061122.129808435127

06.314412163703579.545088190823957.291694708424927.0380390366684711.142306285255800

0002.491610154828619.43474377344236000

098.715787061733394.831622185044691.925980832783148.1090799527503106.6453252663100

1499.7712611751799.576195673189726.306519292868629.1963134090611183.58843861641815.0267768254541604.625093650451484.17507649063

10.2401074305941000008.652132139955820

10.2401074305941000008.652132139955820

1290.289966107914177.400644567434420.77413266114547.395906721873596.314099078213779.447249878371582.04268088091431.55668336622

44.9766796497003109.08390796354767.462877604628784.373622731963649.432443618846656.828612555099312.5249912883178.09338911441228

010.999161349847511.63870423267924.1840245996178502.2643759235366500

070.152326567417475.350887196819376.636298454575423.74132247505885.863361549498800

52.4691166949817222.554819516664141.435367081046154.925048284463230.708244203647169.531118280429186.126209452848117.253925039121

1192.844169763233714.413967432624064.881451152644188.211343749043277.315625974933415.988670284041373.997736673491293.47223224574

303.752598448551970.044558195196889.178256377333964.238728528218611.570375243328874.81520284054370.027014430026408.438983132755

14.263006778327591.989461447037472.952543755331381.973237943925842.851293950133678.66142629477389.8881510170923818.8543145892262

023.92327714277654.1093150424766915.139947681686713.005905989981821.025276333817700

09.802351598380622.4544251642478313.3306505428577.723615447115989.2175585776945200

7.7207159198923774.027454409968788.668330246050483.025509248460683.144403067909580.972596625252716.533895519776927.4704836898725

64.96456175865531.048690293104722.828842074309927.729138592835630.951053547934924.7032631216243113.5141085205283.1044627149906

20.0111550695281118.71217023388690.879973980215135.47956070698660.9106266929145136.56143516172118.78218871281046.78882934786354

01.267294677265049.386867978965210.84662898922673.042363028392646.5223871710566600

05.2335161734436613.53688691800899.401012878443094.187991001677645.3870689053714900

4.3129516518019510.903631144625630.551595550575213.054807142406235.699962560632319.43357334332628.0980547122739213.1763485864937

027.169741362242425.097091572571533.428332198290319.045063519694636.987502803576300

028.032806393415425.260440732782627.849937373871123.917734173722438.852258758701400

183.04828806459466.585603591356448.123526288956643.88967132165667.18938348439733.1504295939261189.881655623837259.044544204308

011.97677045335958.01128753753618.94200623650521.4888159500644812.004658609934800

029.641928115511732.448654217576316.77757457301179.6644766676258614.063221300347500

013.2705328806294.0120411338666516.2258514960796.826765819807893.9028267950387900

059.143629823477963.068606720681490.573978483115415.744080078896953.72071341325500

789.398040632705193.46228159986153.232932325819193.302254799832494.716663183432277.930528079913805.094501229695824.318786766893

675.00715294544757.381001156543860.567984611920958.1474213271378414.113107895888152.394800736477718.143026751323699.2773112835

48.041538596097821.310922620280119.946115357526726.020188862885426.069278478183526.080331078029320.997810403483753.6174271280769

55.5403468033103114.77035782303672.7188323563719109.13464460980837.256659611080697.677441836556628.610634493053759.0407207485654

04.6870798113923115.023241802121620.804744575152805.047163348591300

04.6870798113923115.023241802121620.804744575152805.047163348591300

66.7546463439025102.856794299926134.842259814956119.04627939826358.0070455010707134.38663906522783.403366092600261.0716001859912

28.922603988220227.674464036101918.58008429720719.999159567231216.319359858326420.301201739709230.407452520957623.0033520709824

14.574626121929452.598749355082589.210362780760280.643230264445418.071033643352871.755123932081736.937200448125715.720957678147

23.25741623375290007.914836464610171.2340557732384816.05871312351722.3472904368618

014.5738887885488.4116089681636113.85958148623417.9516306423898624.547893534704100

014.5738887885488.4116089681636113.85958148623417.9516306423898624.547893534704100

014.5738887885488.4116089681636113.85958148623417.9516306423898624.547893534704100

014.5738887885488.4116089681636113.85958148623417.9516306423898624.547893534704100

014.5738887885488.4116089681636113.85958148623417.9516306423898624.547893534704100

014.5738887885488.4116089681636113.85958148623417.9516306423898624.547893534704100

68.9265351753839106.133344211485126.75827818083103.056319761473144.600416434097108.87457428991429.698890109010150.740557047362
[truncated: 2,962,396 more chars]
