# Supplementary figures and images for: Analysis of Antibiotic Resistance Genes in Water Reservoirs and Related Wastewater from Animal Farms in Central China
Source: Microorganisms. 2024 Feb 16;12(2):396. doi: 10.3390/microorganisms12020396 (PMC10893252; doi:10.3390/microorganisms12020396)

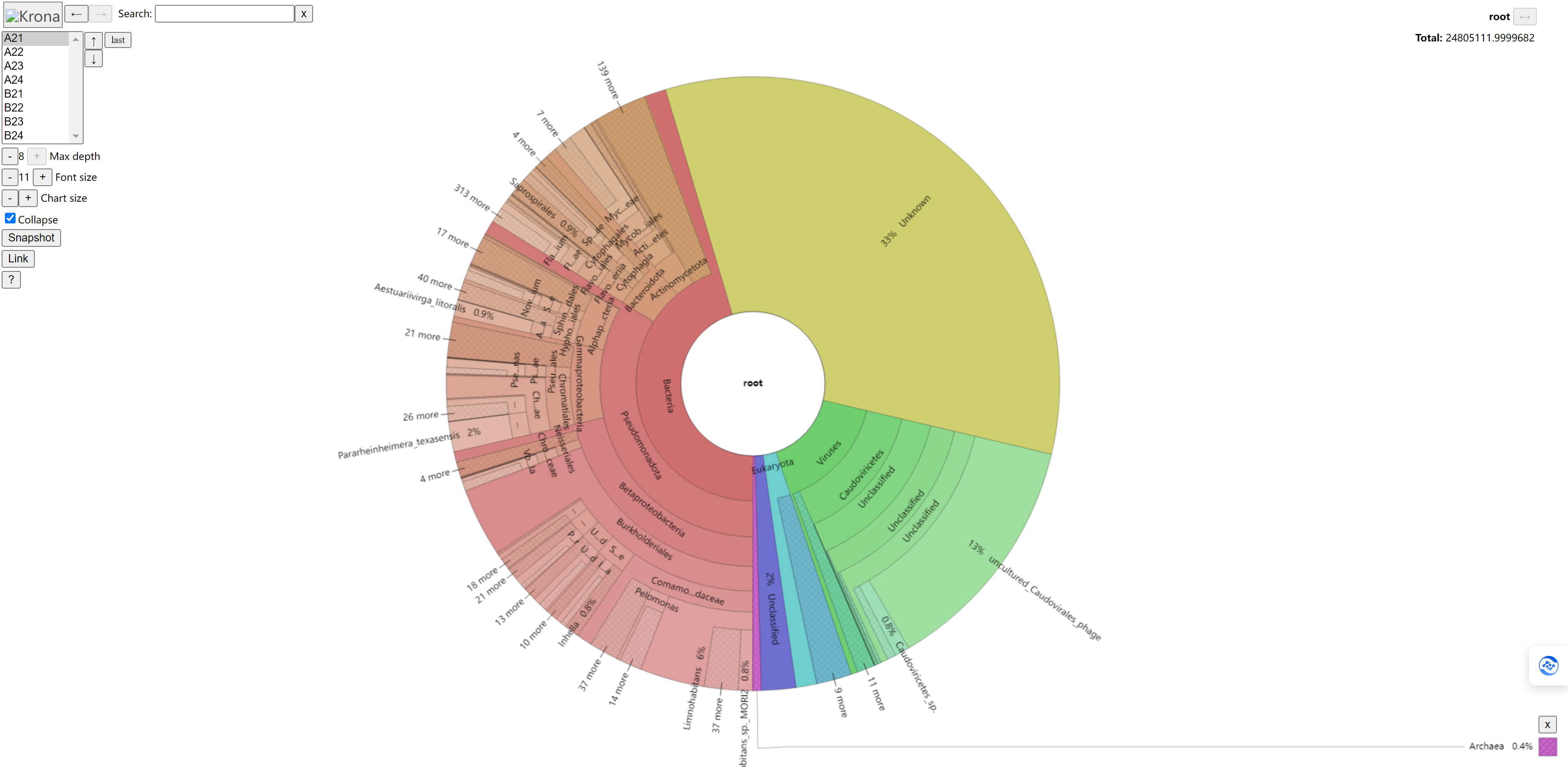

Supplement: Supplementary file 1 [file microorganisms-12-00396-s001.zip › 20240131123145.png]

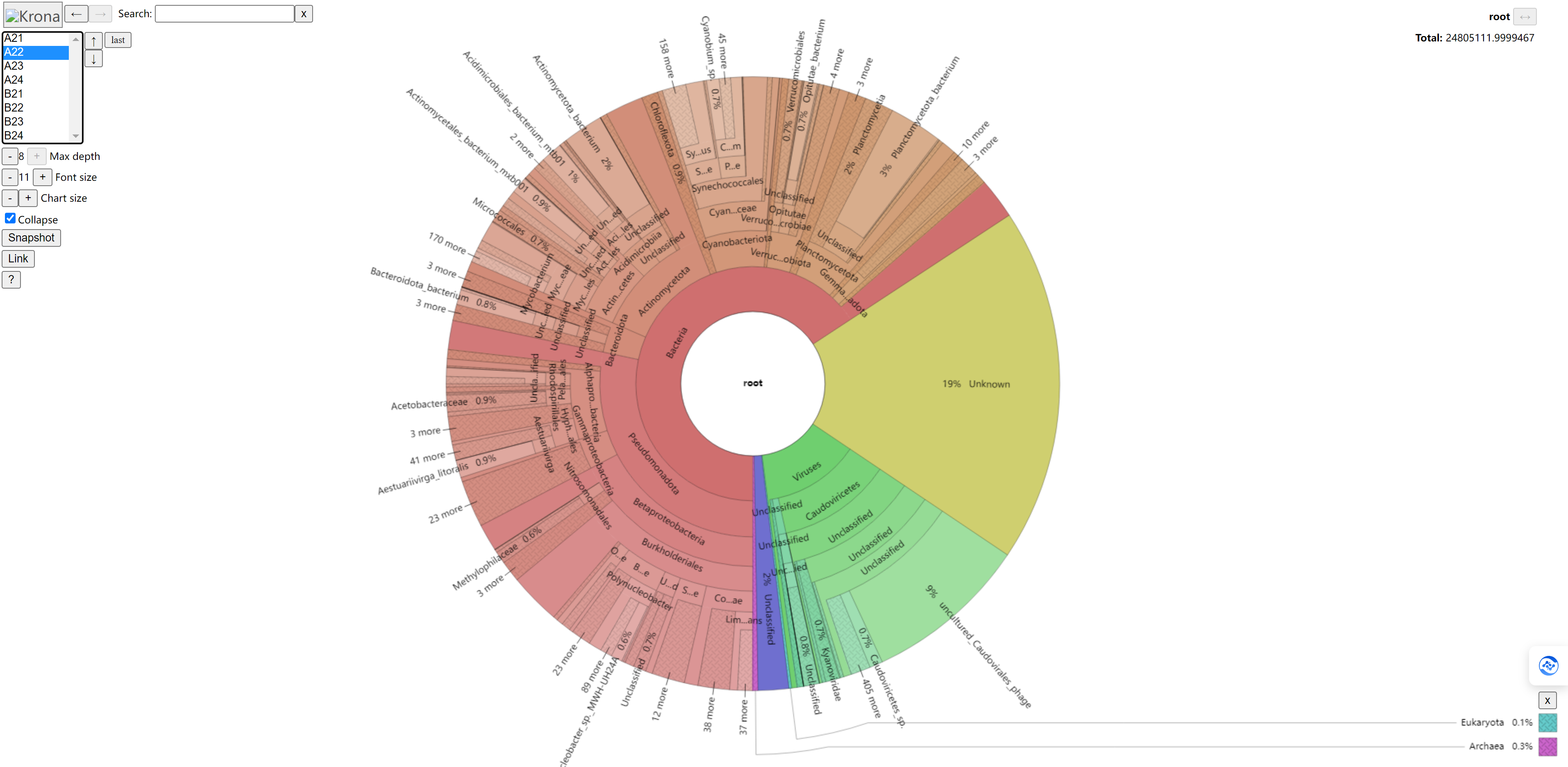

Supplement: Supplementary file 1 [file microorganisms-12-00396-s001.zip › 20240131123156.png]

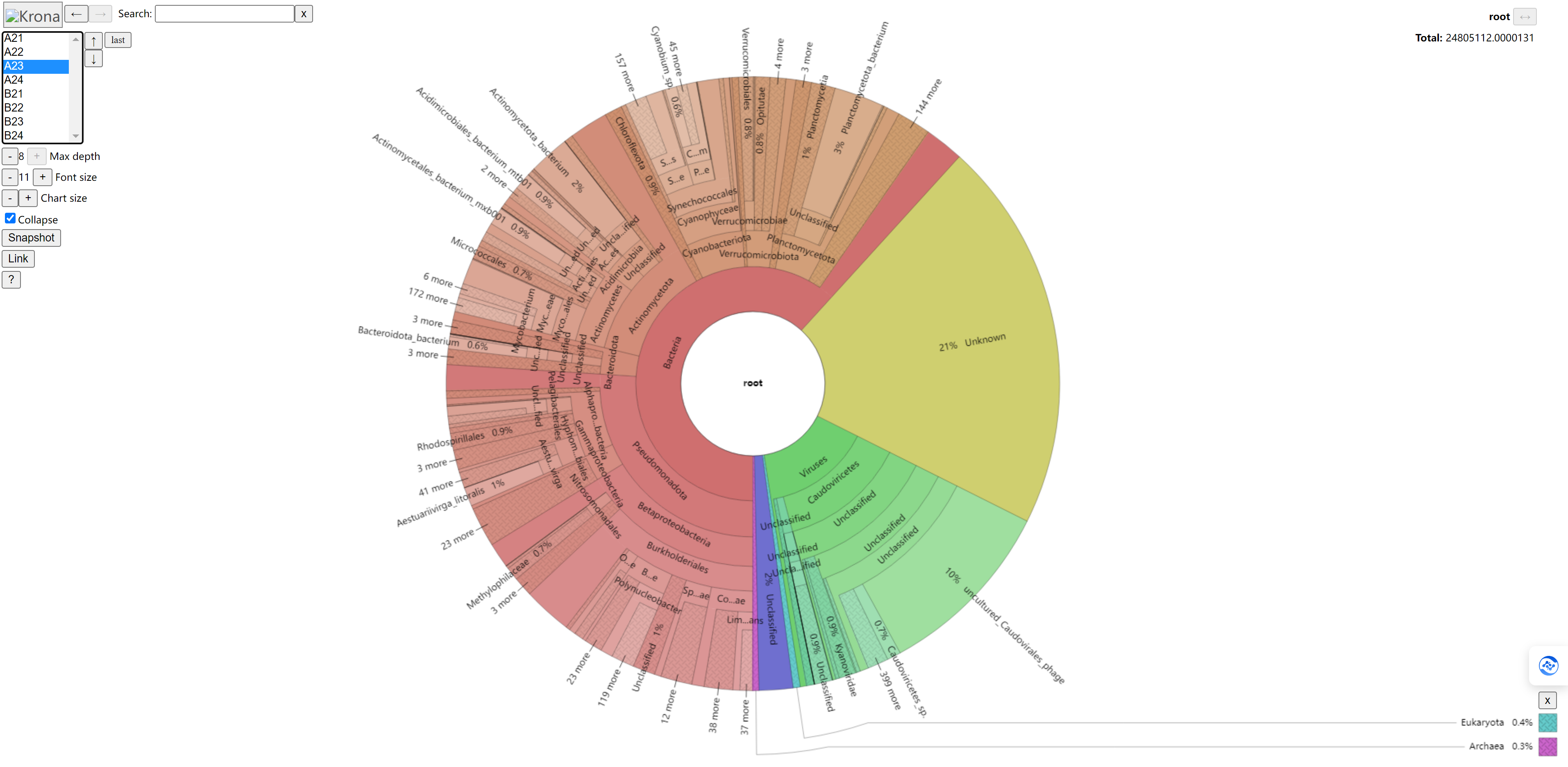

Supplement: Supplementary file 1 [file microorganisms-12-00396-s001.zip › 20240131123203.png]

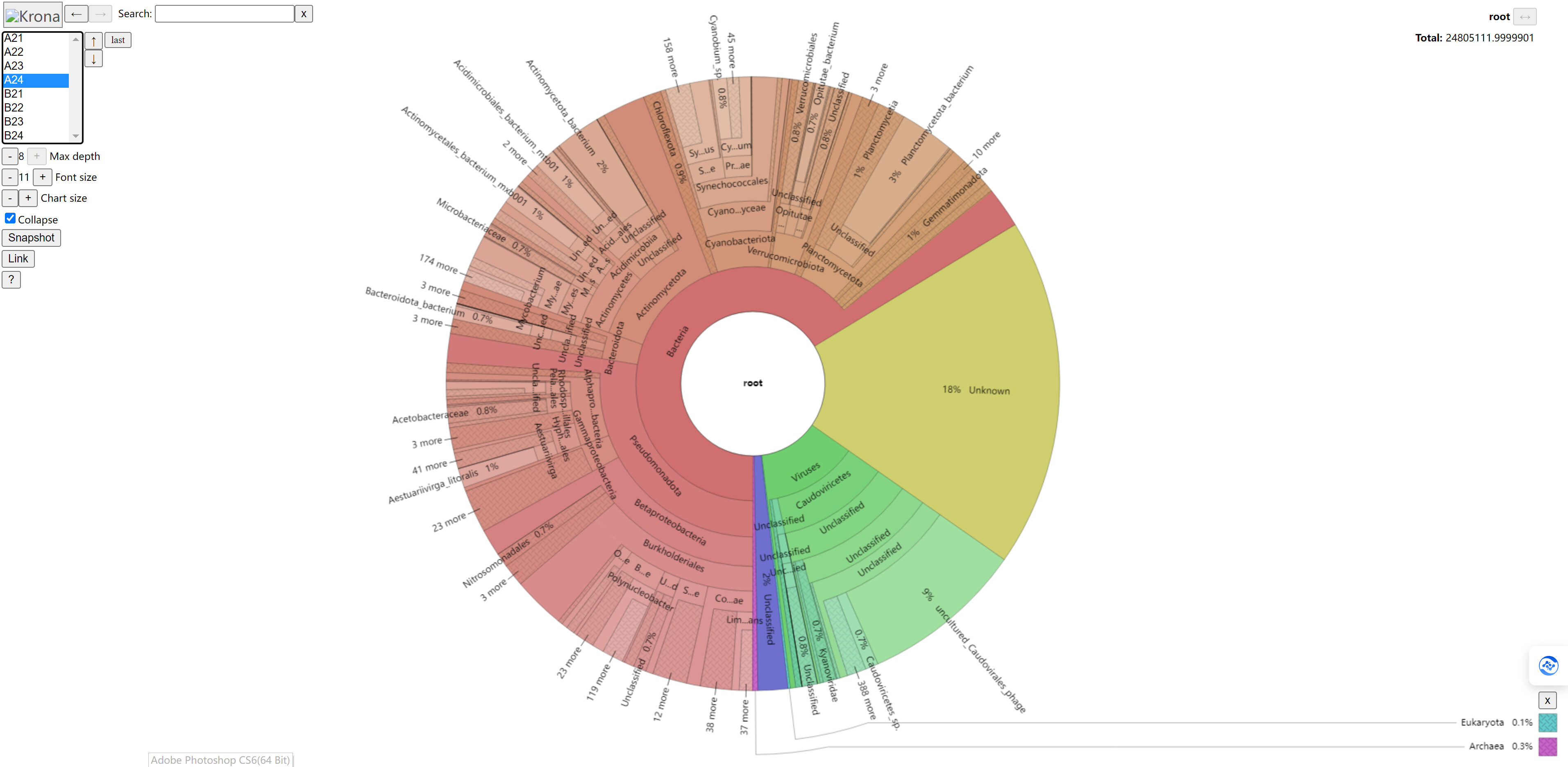

Supplement: Supplementary file 1 [file microorganisms-12-00396-s001.zip › 20240131123209.png]

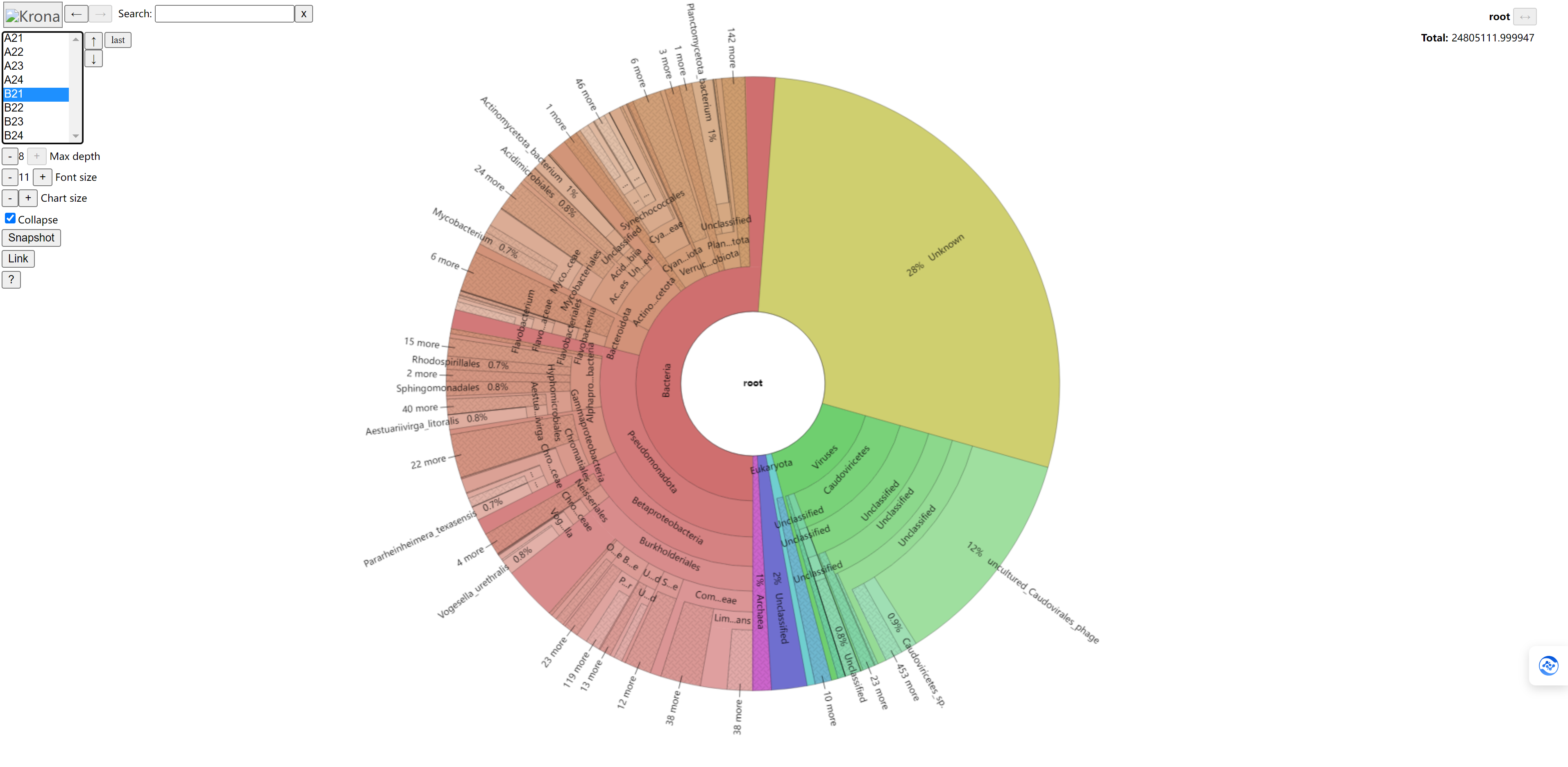

Supplement: Supplementary file 1 [file microorganisms-12-00396-s001.zip › 20240131123221.png]

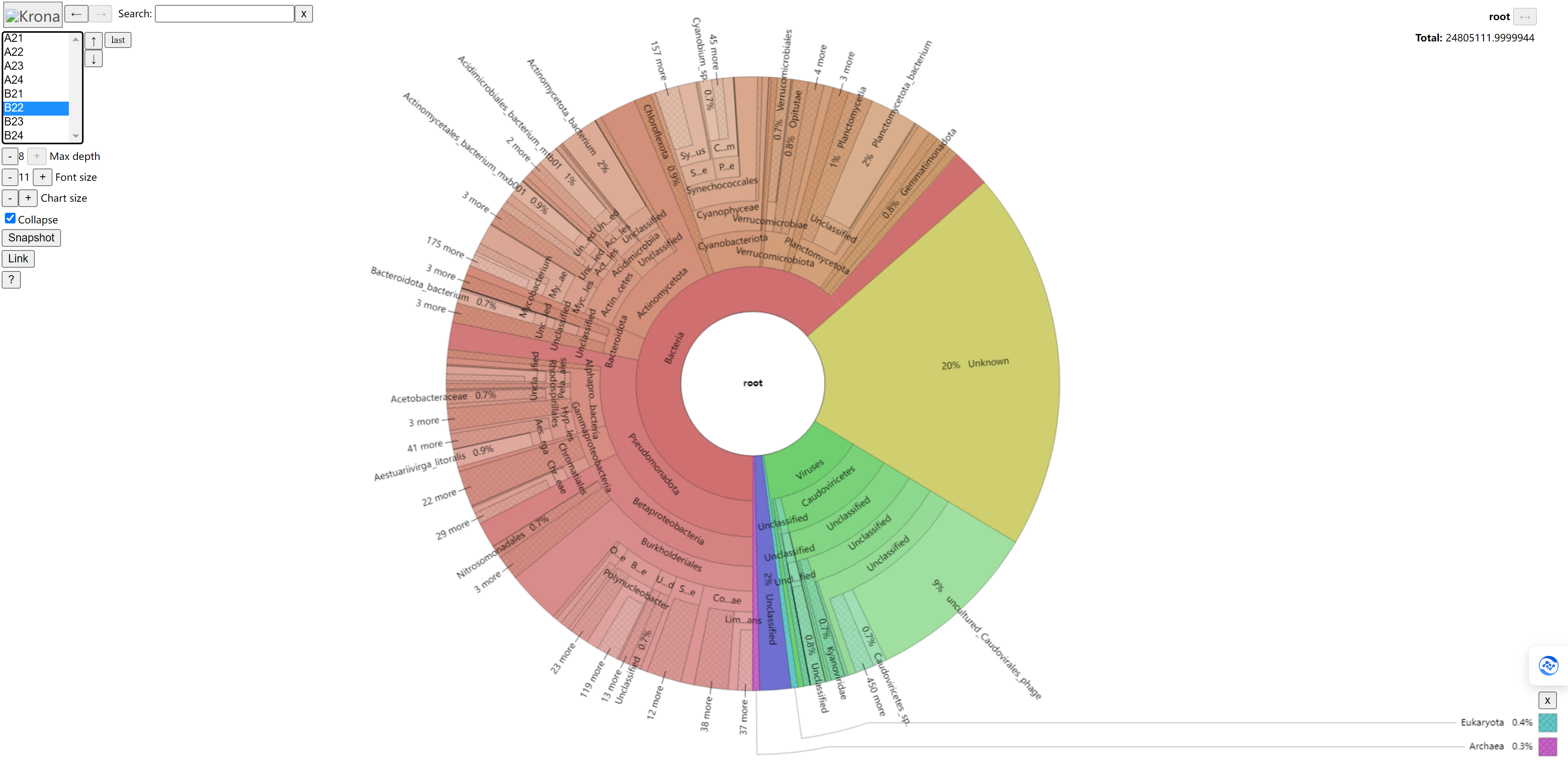

Supplement: Supplementary file 1 [file microorganisms-12-00396-s001.zip › 20240131123229.png]

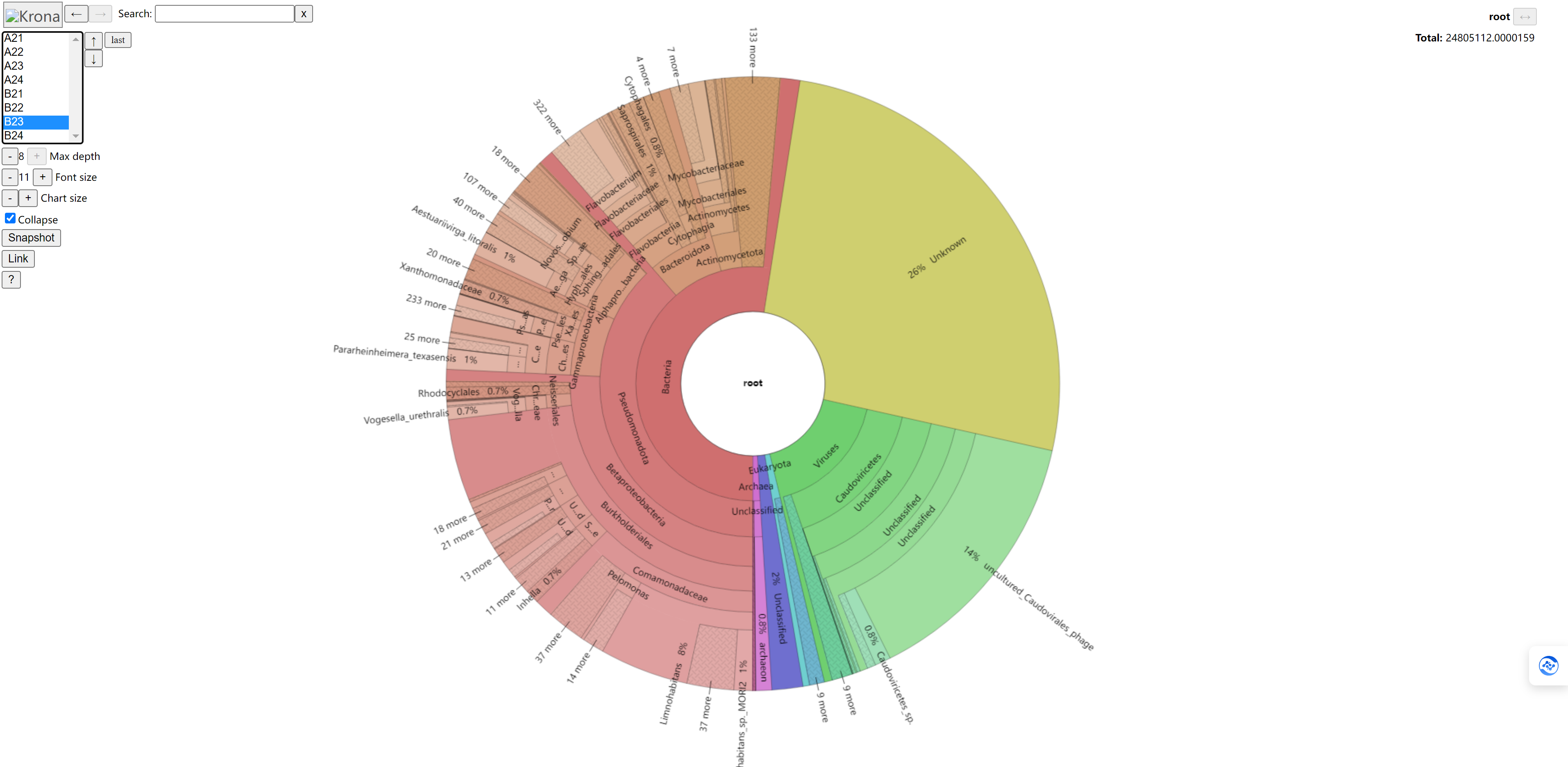

Supplement: Supplementary file 1 [file microorganisms-12-00396-s001.zip › 20240131123235.png]

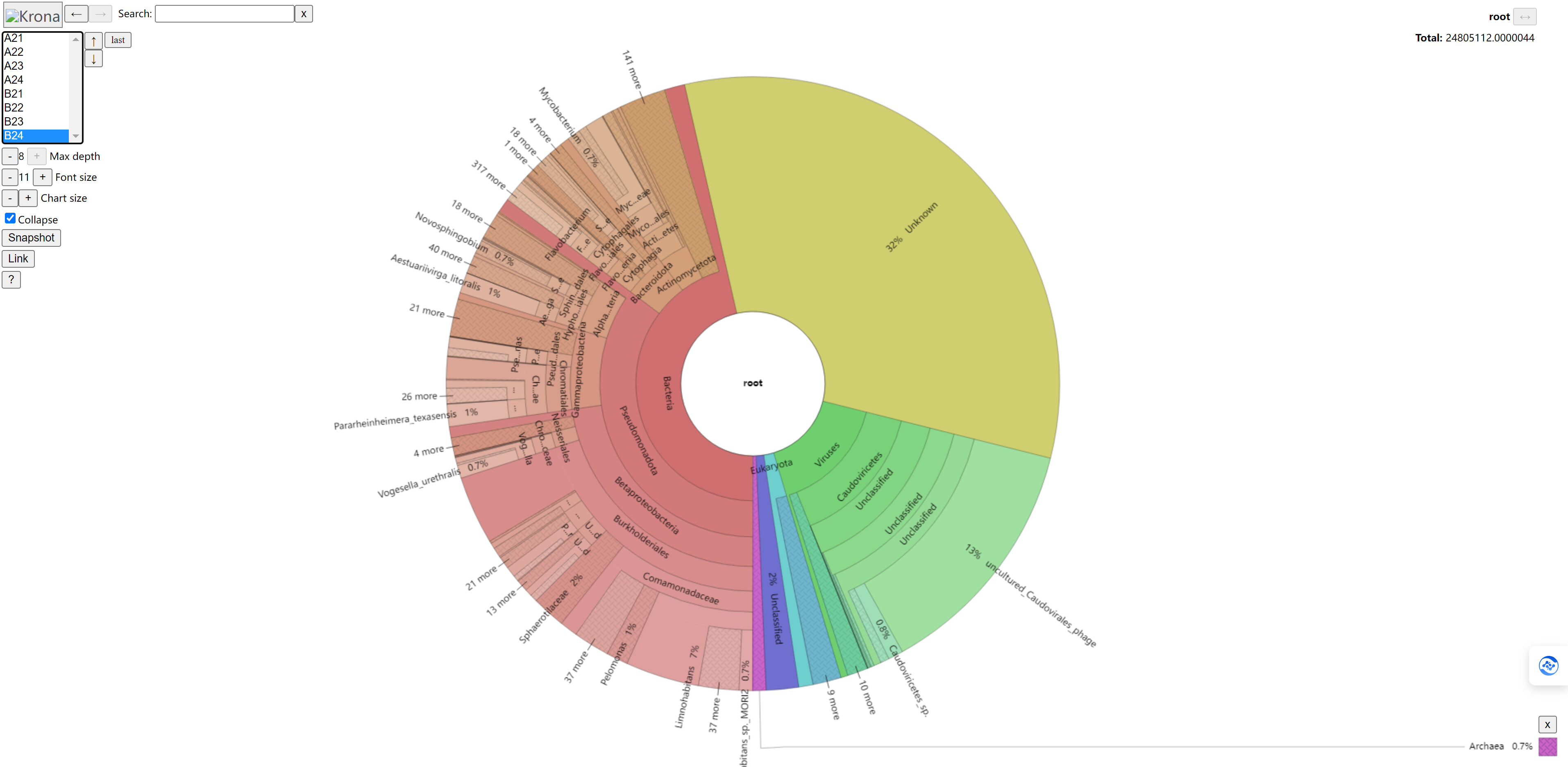

Supplement: Supplementary file 1 [file microorganisms-12-00396-s001.zip › 20240131123239.png]

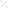

Supplement: Supplementary file 1 [file microorganisms-12-00396-s001.zip › Krona_geneset_origin_group.list.result/img/hidden.png]

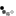

Supplement: Supplementary file 1 [file microorganisms-12-00396-s001.zip › Krona_geneset_origin_group.list.result/img/loading.gif]
